# 27th Annual Computational Neuroscience Meeting (CNS*2018): Part One

**DOI:** 10.1186/s12868-018-0452-x

**Published:** 2018-10-29

## K1 Probabilistic models of sensorimotor control and decision making

### Daniel Wolpert^1^, Mortimer B. Zuckerman^2^

#### ^1^University of Cambridge, Department of Neuroscience, UK; ^2^Columbia University, Mind Brain Behavior Institute, New York, NY, United States

##### **Correspondence**: Daniel Wolpert (wolpert@eng.cam.ac.uk)

*BMC Neuroscience* 2018, **19(Suppl 2):**K1

The effortless ease with which humans move our arms, our eyes, even our lips when we speak masks the true complexity of the control processes involved. This is evident when we try to build machines to perform human control tasks. I will review our work on how humans learn to make skilled movements covering probabilistic models of learning, including Bayesian and structural learning as well as the role of context in activating motor memories. I will also review our work showing the intimate interactions between decision making and sensorimotor control processes. This includes the bidirectional flow of information between elements of decision formations such as accumulated evidence and motor processes such as reflex gains. Taken together these studies show that probabilistic models play a fundamental role in human sensorimotor control.

## K2 The Bayesian brain: from predictive coding to decision making

### Rajesh Rao

#### University of Washington, Department of Computer Science & Engineering, Seattle, WA, United States

##### **Correspondence**: Rajesh Rao (raocs@washington.edu)

*BMC Neuroscience* 2018, **19(Suppl 2):**K2

How can the structure of brain circuits inform large-scale theories of brain function? We explore this question in the context of Bayesian models of perception and action, which prescribe optimal ways of combining sensory information with prior knowledge and rewards to enact behaviors. I will briefly review two Bayesian models, deep predictive coding and partially observable Markov decision processes (POMDPs) and illustrate how circuit structure can provide important clues to systems-level computation.

## K3 Coordination, modulation and functional implications of brain rhythms

### Nancy Kopell

#### Boston University, Department of Mathematics & Statistics, Boston, MA, United States

##### **Correspondence**: Nancy Kopell (nk@bu.edu)

*BMC Neuroscience* 2018, **19(Suppl 2):**K3

The neuroscience community is just beginning to understand how brain rhythms take part in cognition and how flexible are the kinds of computations that can be made with rhythms. In this talk, I will discuss some case studies demonstrating this enormous flexibility and important functional implications. Each of the case studies is about some form of coordination. Examples include the interaction of multiple intrinsic time scales in a cortical rhythm in response to a periodic input; the ability of a slow rhythm in the striatum to modulate two other rhythms in different phases of its period; and the ability of a parietal rhythm to guide the formation, manipulation and termination of a kind of working memory.

## K4 Differential resilience to perturbation of circuits with similar performance

### Eve Marder

#### Brandeis University, School of Life Sciences, Waltham, MA, United States

##### **Correspondence**: Eve Marder (marder@brandeis.edu)

*BMC Neuroscience* 2018, **19(Suppl 2):**K4

Experimental work on the crustacean stomatogastric ganglion (STG) has revealed a 2–6 fold variability in many of the parameters that are important for circuit dynamics. At the same time, a large body of theoretical work shows that similar network performance can arise from diverse underlying parameter sets. Together, these lines of evidence suggest that each individual animal, at any moment in its life-time, has found a different solution to producing “good enough” motor patterns for healthy performance in the world. This poses the question of the extent to which animals with different sets of underlying circuit parameters can respond reliably and robustly to environmental perturbations and neuromodulation. Consequently, we study the effects of temperature, pH, hi K+, and neuromodulation on the pyloric rhythm of crabs. While all animals respond remarkably well to large environmental perturbations, extreme perturbations that produce system “crashes” reveal the underlying parameter differences in the population. Moreover, models of homeostatic regulation of intrinsic excitability give insight into the kinds of mechanisms that could give rise to the highly variable solutions to stable circuit performance.

## F1 Predictive computations in the primary visual cortex

### Jan Homann, Michael Berry, Sue-Ann Koay, Alistair M. Glidden, David W. Tank

#### Princeton University, Department of Neuroscience, Princeton, NJ, United States

##### **Correspondence**: Jan Homann (jhomann@princeton.edu)

*BMC Neuroscience* 2018, **19(Suppl 2):**F1

Predictions about the future are important for an animal in order to interact with its environment. Therefore, predictive computation might be a core operation carried out by neocortical microcircuits. We explored whether the primary visual cortex can perform such computations by presenting repeated temporal sequences of static images with occasional unpredictable disruptions. Simultaneous recordings of 150–250 neurons were performed using two-photon Ca++ imaging of layer 2/3 neurons labeled with GCaMP6f in awake mice, who were head-fixed but free to run on a styrofoam ball. In our visual stimuli, each spatial frame consisted of either an oriented grating or a random superposition of Gabor filters. We found that most of the neurons (~ 98%) showed a strong reduction in activity over a few repeats of the temporal sequence. When we presented a frame that violated the temporal sequence, these neurons responded transiently. In contrast, a small fraction (~ 2%) had activity that ramped up over several repeats, before reaching a steady, sequence-modulated response. This partitioning of the neural population into ‘transient’ and ‘sustained’ responses was observed for all temporal sequences tested. At the same time, the identity of which neurons were transient versus sustained depended on the temporal sequence.

These features—adaptation to a repeated temporal sequence and a transient response to a sequence violation—are hallmarks of predictive coding. After a few repeats, the temporal sequence becomes predictable and can be efficiently represented by a small subset of the neural population. The unpredictable frame then elicits an ‘error’ signal because it encodes a potentially important novelty. In order to explore whether neural novelty signals could be useful to the animal, we performed behavioral experiments with matched visual stimuli that demonstrated that mice could easily learn to lick in response to a violation of an ongoing temporal sequence.

## F2 Response to deep brain stimulation in essential tremor: predictions beyond noisy data with a Wilson-Cowan model

### Benoit Duchet^1^, Gihan Weerasinghe^1^, Christian Bick^2^, Hayriye Cagnan^1^, Rafal Bogacz^1^

#### ^1^University of Oxford, Nuffield Department of Clinical Neurosciences, Oxford, United Kingdom; ^2^University of Oxford, Mathematical Institute, Oxford, United Kingdom

##### **Correspondence**: Benoit Duchet (benoit.duchet@ndcn.ox.ac.uk)

*BMC Neuroscience* 2018, **19(Suppl 2):**F2

Thalamic deep brain stimulation (DBS) is a therapy option for Essential tremor (ET), the most common movement disorder. Clinically available DBS delivers constant, high frequency electrical stimulation and could be improved in terms of efficacy, reduction of side effects, and decrease in power usage. Given phased locked stimulation data, we propose a method to study the effects of stimulation along both the tremor oscillation phase axis and the tremor oscillation amplitude axis, with the goal of better informing stimulation strategies. Because of noise in tremor recordings and experimental limitations, the amplitude axis is especially difficult to access by direct data analysis in the phasic paradigm. We show that a Wilson-Cowan model can be fitted to data, and thanks to isochronal and isostable coordinates, we obtain response curves and surfaces for the noiseless model. The noiseless 2D phase response curves and amplitude response curves show good agreement with the response curves obtained directly from experimental data (Fig. 1). The 3D response surfaces give us the ability to make predictions beyond what the noise level of the data can let us see. In that sense, our method can be seen as a way of de-noising the experimental response to stimulation. Although mathematically inspired by a canonical neuroscience model, our model includes the various neural populations thought to be involved in the generation of ET, and allows for the stimulation of the most common target for ET DBS, the ventral intermediate nucleus of the thalamus. Our model predicts that only certain phases are conducive to amplitude reduction through stimulation, the best of which being the phase that brings the system closer to the fixed point, where there are no pathological oscillations. This particular phase is amplitude dependent, but in general the optimal stimulation phase occurs during the descending part of the oscillations, slightly before the trough. Moreover, the response to stimulation is linearly dependent on stimulation magnitude. We also find that the best phase to stimulate corresponds to the maximum positive slope of the PRC. Finally, we report that the effects of stimulation are reduced as the amplitude of the oscillations increases, and therefore predict that phasic stimulation will be less effective when delivered at higher oscillation amplitudes.

**Fig. 1:**
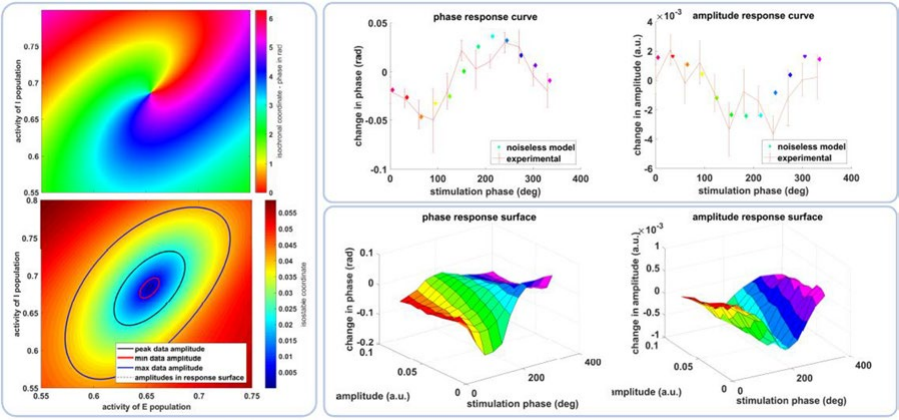
Response curves and surfaces from isochronal and isostable coordinates for “patient 1”. The model response curves agree with experimental data

## F3 A molecular odorant transduction model and combinatorial encoding in the Drosophila Antennae

### Aurel A. Lazar, Chung-Heng Yeh

#### Columbia University, Department of Electrical Engineering, New York, NY, United States

##### **Correspondence**: Chung-Heng Yeh (chyeh@ee.columbia.edu)

*BMC Neuroscience* 2018, **19(Suppl 2):**F3

A key functionality of olfactory sensory neurons (OSNs) in the *Drosophila* antennae is to jointly encode both odorant identity and odorant concentration. The identity of an odorant is combinatorially encoded by the set of responding OSN groups expressing the same receptor type, and the size of OSN set varies as the concentration changes. The temporal response of an OSN simultaneously represents the information of odorant concentration and concentration gradient. These two aspects of olfactory coding, *identity* and *concentration*, originate in the odorant transduction process. However, detailed molecular models of the odorant transduction process are scarce for fruit flies. To address these challenges we advance a comprehensive model of fruit fly OSNs as a cascade consisting of an odorant transduction process (OTP) and a biophysical spike generator (BSG). We model identity and concentration in OTP by an odorant-receptor binding rate tensor modulated by the odorant concentration profile and an odorant-receptor dissociation rate tensor, and quantitatively describe the ligand binding/dissociation process. To biologically validate our modeling approach, we first propose an algorithm for estimating the affinity and the dissociation rate of an odorant-receptor pair. We then apply the algorithm to electrophysiology recordings and estimate the affinity and dissociation rate for three odorant-receptor pairs, (*acetone*, *Or 59b*), (*methyl butyrate*, *Or 59b*) and (*butyraldehyde*, *Or 7a*). Second, we evaluate the temporal response of the OSN model with a multitude of stimuli, including step, ramp and parabolic odorant waveforms for all three odorant-receptor pairs. The output of the model closely reproduces the temporal responses of OSNs obtained from in vivo electrophysiology recordings for all three odorant-receptor pairs across all three types of stimuli (Fig. 1). Lastly, we evaluate the model at the OSN antennae population level. We first empirically estimate the odorant-receptor affinity using the spike count records in the DoOR database for 24 receptor types in response to 110 odorants. With estimated affinity values, we simulate the temporal response of the OSN population to staircase odorant waveforms. The output of simulated OSN population demonstrates that the odorant identity is encoded in the set of odorant-activated OSN groups expressing the same receptor type, and, more importantly, the size of the set expands or reduces as the odorant concentration increases or decreases. The fruit fly OSN model presented here provides a theoretical foundation for understanding the neural code of both odorant identity and odorant concentration. It advances the state-of-the-art in a number of ways. First, it models on the molecular level the combinatorial complexity of the transformation taking place in *Drosophila* antennae OSNs. The resulting *concentration*-*dependent combinatorial code* determines the complexity of the input space driving olfactory processing in the downstream neuropils, such as odorant recognition and olfactory associative learning. Second, the model is biologically validated using multiple electrophysiology recordings. Third, the OSN model demonstrates that the currently available data for odorant-receptor responses only enables the estimation of the affinity of the odorant-receptor pair. Our model calls for new experiments for massively identifying the odorant-receptor dissociation rates of relevance to flies.

**Fig. 1:**
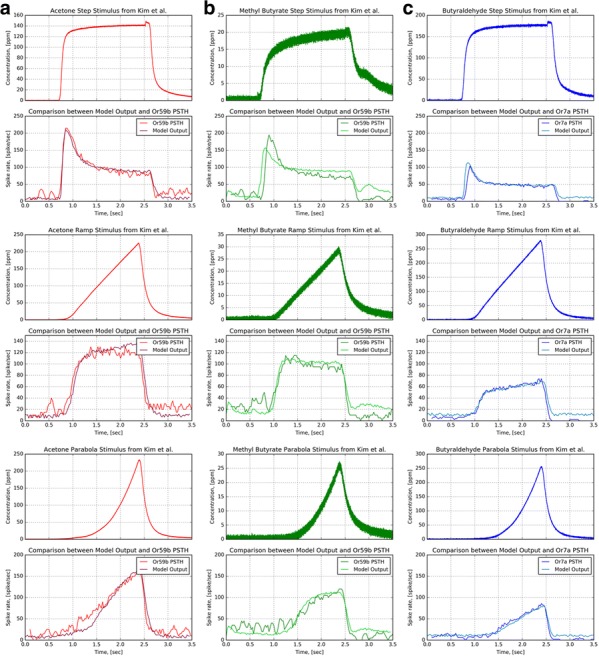
Characterization of the fruit fly OSN model with multiple odorants and receptor types. Three odorant-receptor pairs are tested. (**a**) (Or59b, acetone) (**b**) (Or59b, methyl butyrate). (**c**) (Or7a, butyraldehyde). (Odd rows) Stimuli. (Even rows) PSTH from the model output and experimental recordings

## O1 Generative model of visual cortex with short- and long-range recurrent interactions

### Federica Cappareli, Klaus Pawelzik, David Rotermund, Udo Ernst

#### University of Bremen, Institute for Theoretical Physics, Bremen, Germany

##### **Correspondence**: Federica Cappareli (federica@neuro.uni-bremen.de)

*BMC Neuroscience* 2018, **19(Suppl 2):**O1

In V1, neuronal responses are sensitive to context: responses to stimuli presented within the classical receptive field are modulated by stimuli in the surround. Recently, sparse coding models [1] have been successful in explaining part of these modulatory effects [2]: Their dynamics implements an inference process to seek an optimal (w.r.t. accuracy and sparseness) representation of a visual input in terms of fundamental features. This is achieved through a competition between similarly tuned neurons with overlapping input fields, which also mediates contextual modulation. However, this connection scheme implies that neurons with non-overlapping input fields do not interact. Therefore, the proposed mechanism does not provide a satisfactory explanation of the mechanisms behind these phenomena, since contextual effects are usually caused by surround stimuli positioned far from the cRF (e.g. Mizobe et al.) 21 report collinear modulation for distance center-surround up to 12 deg). To overcome this limitation, we propose an extension of the classical framework [2] by defining a new generative model for visual scenes that includes dependencies among different features in spatially well-separated locations. To perform inference in this model, we also derive a dynamical system that can be mapped to a neural circuit and a lateral connection scheme for optimally processing local and contextual information.

The result can be interpreted as a neural network where units are linked by short range horizontal connections within the same hypercolumn and by long range connections between different hypercolumns (Fig. 1b). Each hypercolumn contains units that receive input from a localized region of the visual field and builds a sparse representation of its input as if it was presented in isolation. In parallel, these local representations are combined by providing contextual information to each other. In our simulations connections are learned from natural images. Long-range connections reflect the co-occurrence of features in different visual field locations: this predicts a connectivity structure linking neurons with similar orientation and spatial frequency preferences, which is similar to the typical patterns found for long-ranging (3–4 mm) horizontal axons in visual cortex [3]. Subjected to contextual stimuli typically used in empirical studies, our model replicates several hallmark effects of contextual processing. Hereby local and long-range interactions act hand-in-hand, for example in realizing two different origins of near and far surround suppression, respectively [4]. In summary, our model provides a novel framework for contextual processing in the visual system proposing a well-defined functional role for horizontal axons.

**Fig. 1:**
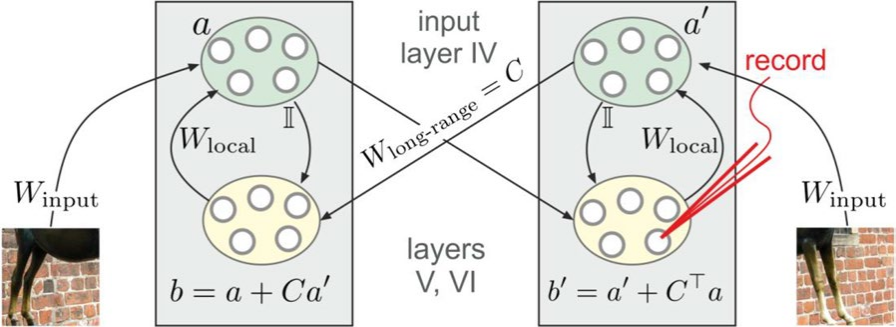
(A) Example of stimuli from a natural scene (top) and dictionary of fundamental features (bottom) (B) Scheme of the generative model (C) Network architecture to perform inference in the generative model

**Acknowledgements**

This work has been supported by the Creative Unit I-See of the University of Bremen and the BMBF, Bernstein Award Udo Ernst, Grant No. 01GQ1106.

**References**

1. Olshausen BA, Field DJ. Sparse coding with an overcomplete basis set: A strategy employed by V1? 1997
2. Zhu M, Rozell CJ. Visual nonclassical receptive field effects emerge from sparse coding in a dynamical system. 2013
3. Florencia Iacaruso M., Gasler I.T. Hofer SB. Synaptic organization of visual space in primary visual cortex
4. Angelucci A, Bijanzadeh M, Nurminen L, Federer F, Merlin, Bressloff PC. Circuits and Mechanisms for Surround Modulation in Visual Cortex. 2017

## O2 Info in a bottleneck: exploring the compression of visual information in the retina

### Gabrielle Gutierrez^1^, Eric Shea-Brown^1^, Fred Rieke^2^

#### ^1^University of Washington, Department of Applied Mathematics, Seattle, WA, United States; ^2^University of Washington, Departments of Physiology & Biophysics, Seattle, WA, United States

##### **Correspondence:** Gabrielle Gutierrez (ellag9@uw.edu)

*BMC Neuroscience* 2018, **19(Suppl 2):**O2

The retina is organized in convergent and divergent layers that compress and expand signals before passing visual information along to the brain. Receptive fields anatomically correspond to the collection of inputs that converge upon a single retinal output cell. This subunit circuit structure produces an information bottleneck because information is compressed along the pathway to an output neuron. We wondered whether the structure of the retina combined with its adaptation properties serve to preserve information given this bottleneck.

A remarkable property of the retina is its ability to adapt its processing to environmental conditions. Adaptation to background luminance shifts the nonlinear response filters of the subunits over a timescale of about a minute. This has the effect of adjusting the linearity of responses in a manner that is dependent on the luminance environment. Another feature of the retina is the diversity of cell types present at the output layer. Within types, there are ON and OFF versions of cell types which have sensitivities that are complementary but not symmetrical. Having complementary cell types combined with adaptation mechanisms may allow the retina to leverage these redundancies under certain conditions while having the flexibility to adapt to an efficient or predictive code in other conditions. We want to know whether the retina adapts its processing to maximize visual information transmission by adjusting the subunit response functions in the circuit. To quantify the amount of information that is preserved in the signals exiting the retina under this kind of set up, we estimate the mutual information between a naturalistic stimulus set and the output from our model retina circuit. We use a binless estimator to account for the fact that the input signals and the outputs are continuous. Consistent with past studies, our preliminary results indicate that the optimal thresholds for the nonlinear subunits depend on the amount of input noise given a naturalistic distribution of stimulus contrasts. Our work builds on past studies by incorporating the known subunit structure into the circuit which produces information compression. Under circumstances where subunits receive independent inputs, rather than correlated inputs, the circuit is optimal when ON and OFF subunits redundantly encode the most prevalent stimuli for a broad range of subunit noise levels. Our preliminary results suggest novel ways in which adaptation mechanisms, along with the particular bottleneck structure of the retina, enable the retina to adapt the computations it produces in different contexts.

## O3 Structural and dynamical properties of local cortical networks result from robust associative learning

### Danke Zhang, Chi Zhang, Armen Stepanyants

#### Northeastern University, Department of Physics, Boston, MA, United States

##### **Correspondence**: Armen Stepanyants (a.stepanyants@neu.edu)

*BMC Neuroscience* 2018, **19(Suppl 2):**O3

Many ubiquitous features characterize the structure and dynamics of local cortical networks. At the level of pair-wise connectivity, it is known that the probabilities of excitatory connections are generally lower than those for inhibitory, and the majority of reported probabilities lies in the 0.10–0.19 range if the presynaptic cell is excitatory and 0.25–0.56 range if it is inhibitory. It is also known that the distributions of connection weights have stereotypic shapes with the majority of measured coefficients of variation (CV) of unitary postsynaptic potentials in the 0.85–1.1 range for excitatory connections and slightly lower values for inhibitory, 0.78–0.96. At the level of connectivity within 3-neuron clusters, several overrepresented connectivity motifs have been discovered. Information becomes scarce as one considers larger clusters of neurons, but even here deviations from random connectivity have been reported for clusters of 3–8 neurons. Similarly, many universal features characterize activity of neurons in local cortical networks. For example, individual neurons exhibit highly irregular spiking activity, resembling Poisson processes with close to one CV in inter-spike-intervals. Spike trains of nearby neurons are only marginally correlated, 0.04–0.15, and, at the network level, spiking activity can be described as sustained, irregular, and asynchronous. In this study, we pursue a hypothesis that associative learning alone is sufficient to explain these network features. To test this hypothesis, we trained recurrent networks of excitatory and inhibitory McCulloch and Pitts neurons [1, 2] on memory sequences of varying lengths and compared network properties to those observed experimentally. Learning in the network is mediated by changing connection weights in the presence of biologically inspired constraints. (1) Input connection weights of each neuron are sign-constrained to be non-negative if the presynaptic neuron is excitatory and non-positive if it is inhibitory. (2) Input weights of each neuron are homeostatically constrained to have a predefined*l*1-norm. (3) Each neuron must attempt to learn its associations robustly, so that they can be recalled correctly in the presence of a given level of postsynaptic noise. We explore structural and dynamical properties of associative networks in the space of these constraints, and show that there is a unique region of parameters that is consistent with all of the above-described experimental observations. In this region, local cortical circuits are loaded with associative memories close to their capacity and memories can be successfully retrieved even in the presence of noise comparable to the baseline variations in the postsynaptic potential, which provides an independent validation of the theory in terms of the hypothesized network function. Confluence of these results suggests that many structural and dynamical properties of local cortical networks are simply a byproduct of associative learning.

**Acknowledgements**

This work is supported by Air Force grant FA9550-15-1-0398 and NSF grant IIS-1526642.

**References**

1. Chapeton J, Fares T, LaSota D, Stepanyants A. Efficient associative memory storage in cortical circuits of inhibitory and excitatory neurons. *PNAS* 2012, 109, E3614-3622.
2. Chapeton J, Gala R, Stepanyants A. Effects of homeostatic constraints on associative memory storage and synaptic connectivity of cortical circuits. *Front Comput Neurosci* 2015, 9, 74.

## O4 Reduced models of an attractor neural network’s response to conflicting external inputs

### Kathryn Hedrick

#### Southern Methodist University, Department of Mathematics, Dallas, TX, United States

##### **Correspondence**: Kathryn Hedrick (khedrick@smu.edu)

*BMC Neuroscience* 2018, **19(Suppl 2):**O4

The theory of attractor neural networks has been influential in our understanding of the neural processes underlying spatial, declarative, and episodic memory. Many theoretical studies focus on the inherent properties of an attractor, such as its structure and capacity. Relatively little is known about how an attractor neural network responds to external inputs, which often carry conflicting information about a stimulus. In this talk I will present analytical results concerning the behavior of an attractor neural network’s response to conflicting external inputs. My focus is on analyzing the emergent properties of the megamap model, a quasi-continuous attractor network in which place cells are flexibly recombined to represent a large spatial environment (Hedrick and Zhang 2016). In this model, the system shows a sharp transition from the winner-take-all mode, which is characteristic of standard continuous attractor neural networks, to a combinatorial mode in which the equilibrium activity pattern combines embedded attractor states in response to conflicting external inputs. I derive a numerical test for determining the operational mode of the system a priori. I then derive a linear transformation from the full model to a reduced 2-unit model that has similar qualitative behavior. The analysis of the reduced model and explicit expressions relating the parameters of the reduced model to the megamap elucidate the conditions under which the combinatorial mode emerges and the dynamics in each mode given the relative strength of the attractor network and the relative strength of the two conflicting inputs. Although my focus on a particular attractor network model, I describe a set of conditions under which the reduced model can be applied to more general attractor neural networks. The reduced 2-unit model captures the amplitude of each activity bump but not its radius. I extend this reduced model to examine the spatial effects on the system’s behavior by approximating the activity bump and recurrent connections using two-dimensional Gaussian tuning curves. Analysis of this reduced model reveals that these spatial effects underlie the nonlinearities observed in the full megamap model but not in the reduced 2-unit model. I compare these results to numerical simulations and electrophysiological data from an experiment in which hippocampal place cells resolve conflicting external inputs from the medial entorhinal cortex (MEC) and lateral entorhinal cortex (LEC) when local and global cues are rotated in opposite directions (Knierim and Neunuebel 2016). In this experiment, place cells in the CA3 (which are believed to form attractor neural networks) coherently follow the noisy inputs from the LEC rather than the much stronger spatial inputs from the MEC. The reduced model predicts that this surprising response is due to three factors: (1) CA3 place cells are initially driven by the LEC input only, (2) the attractor network acts in the WTA mode, and (3) connections from MEC to CA3 are governed by fast Hebbian synaptic plasticity. To bridge the gap between the idealistic theory and the noisy electrophysiological data, I run numerical simulations using the conductance-based integrate and fire model and unsupervised Hebbian plasticity. The noise in the model leads to the partial remapping observed experimentally.

**Reference**

1. Knierim JJ, Neuneubel JP. Tracking the flow of hippocampal computation: Pattern separation, pattern completion, and attractor dynamics. *Neurobiol Learn Mem* 2016, 129, 38–49.

## O5 Topologies of repetitive functional network motifs vary dynamically with age in the developing human brain: Evidence from very high-dimensional invasive brain signals

### Caterina Stamoulis^1^, Phillip Pearl^2^

#### ^1^Harvard Medical School, Faculty of Medicine, Boston, MA, United States; ^2^Harvard Medical School, Department of Neurology, Boston, MA, United States

##### **Correspondence**: Caterina Stamoulis (caterina.stamoulis@childrens.harvard.edu)

*BMC Neuroscience* 2018, **19(Suppl 2):**O5

Throughout the course of the day, or even an hour, functional brain networks are continuously recruited to process thousands of inputs from the outside world and respond to the demands of countless behaviors and cognitive processes. Across scales of organization, these networks’ small-world and scale-free topologies facilitate optimally efficient neural information processing. However, the building blocks of these networks (modules or motifs), their emergence, re-organization during development and time-dependent stereotypy remain poorly understood. Unrelated theoretical work has shown that specific network patterns emerge as a result of a dynamic system’s propensity towards a stable configuration. There is also growing evidence from both animal and human studies that a relatively small number of such modules are combined (in potentially infinite ways) to give rise to the observed functional network topologies. In this study, we investigated the organization, size and stereotypy of functional network motifs in the developing human brain, using very high-dimensional invasive human electrophysiological signals, collected continuously over long periods of time (typically several days) from a relatively large number of children and young adults (n = 39, age < 1 to ~ 23 years) with intracerebral electrode grids covering different parts of the brain. All patients had recordings from a relatively large number (> 70) of electrodes. Information theoretic and contraction theoretic measures were used to estimate functional connectivity, identify sub-network patterns (motifs) that occurred repetitively over time and independently of the area of the brain being spatially sampled, and characterize their stability (using an eigenvalue analysis).

A relatively small number of functionally active nodes were estimated, which formed stable patterns that occurred repetitively across temporal scales and brain regions. The size of these patterns (number of activated nodes) changed with age, with progressively smaller sub-graphs (3–4 nodes) emerging as a function of neural maturation. Across ages, identified motifs were consistently correlated with network stability. These results indicate the although stable functional network motifs may be in place early in life to process multi-modal sensory information, re-organization of the brain’s neural circuitry as a function of neural maturation may lead to increasingly parsimonious modules to facilitate increasingly efficient neural information processing. These modules may also constitute a network-level biomarker of neural maturation at the macroscale sampled by invasive human recordings.

## O6 Revealing principles of cortical computation using the Allen Brain Observatory: A large, standardized calcium imaging dataset from the mouse visual cortex

### Michael A. Buice^1^, Saskia E.J. de Vries^1^, Gabriel Ocker^1^, Michael Oliver^1^, Peter Ledochowitsch^1^, Daniel Millman^1^, Eric Shea-Brown^2^, Christof Koch^1^, Jianghong Shi^2^, R Clay Reid^1^

#### ^1^Allen Institute for Brain Science, Modelling, Analysis and Theory, Seattle, WA, United States; ^2^University of Washington, Department of Applied Mathematics, Seattle, WA, United States

##### **Correspondence**: Saskia E.J. de Vries (saskiad@alleninstitute.org)

*BMC Neuroscience* 2018, **19(Suppl 2):**O6

A prominent question of sensory processing is how information is represented and transformed by the neural circuit through multiple layers and across multiple areas in order to create perceptions and ultimately guide behavior. In order to facilitate uncovering these principles, we have created the Allen Brain Observatory. This is a public dataset of neural responses collected from visual areas of awake mouse cortex using 2-photon calcium imaging. We systematically recorded responses from over 50,0 neurons in over 5 experiments, using a high-throughput imaging pipeline. Data were collected from 6 cortical areas and 4 cortical layers. GCaMP6f was transgenically expressed, driven by 13 different Cre lines which limit expression to specific subsets of excitatory (10 Cre lines) or inhibitory cells (3 Cre lines). Visual responses were imaged in response to an array of both artificial and natural stimuli, including drifting gratings, static gratings, locally sparse noise, natural scenes and natural movies while the mouse was awake and free to run on a running disc. Several metrics were computed to describe the visual responses of the neurons, including orientation and direction selectivity, image selectivity, lifetime sparseness, and receptive field areas. Surveying these metrics across areas, layers and Cre-defined cell populations, several patterns emerge. Layer 4 exhibited clear differences across areas and cell populations, but these differences were reduced in the other layers. This pattern is consistent with layer 4 predominately carrying feedforward thalamocortical input, while layers 2/3, 5 and 6 represent higher order responses. One of the most striking results in this dataset is the small numbers of responsive cells and the remarkable variability of the responses of these cells. Only 57% of cells in the Brain Observatory dataset respond to any of the visual stimuli presented. Further, even responsive cells show large trial-to-trial variability. We fit these neurons to a simple wavelet pyramid model with simple (linear-nonlinear) and complex components (the “energy” model). Roughly 15% of neurons in the dataset show significantly predictable responses to visual stimuli via this model, with relatively low explainable variance. All cells also show some degree of “complex” behavior, i.e. there are no purely “simple” cells according to this model. We compare the representations in each layer and area to responses generated by standard Convolutional Neural Networks, a model derived from the canonical understanding of the cat visual system. We find that the mouse cortex are most similar to early middle areas of ConvNets, rather than the initial Gabor-like layer thought to describe responses in V1 of cats. Finally, we examine the correlation structure of population activity, showing that correlations in neural responses have an impact on information transmission in an area and layer dependent fashion. Furthermore, we show that the “noise” and “signal” correlations are positively correlated throughout the mouse visual system, providing strong evidence against certain types of theories that exhibit “explaining away”, i.e. theories in which neurons with similar mean tuning properties will functionally inhibit one another, such as the sparse coding model of Olshausen and Field and some probabilistic coding models. This dataset provides a testbed for theories of cortical computations and will be a valuable resource for the community.

## O7 Characterization of the brain’s dynamical repertoire in the psychedelic state

### Louis-David Lord^1^, Paul Expert^2^, Robin Carhart-Harris^3^, Morten Kringelbach^1^, Joana Cabral^4^

#### ^1^University of Oxford, Department of Psychiatry, Oxford, United Kingdom; ^2^Imperial College London, Centre for Mathematics of Precision Healthcare, London, United Kingdom; ^3^Imperial College London, Psychedelic Research Group, London, United Kingdom; ^4^University of Minho, Life and Health Sciences Research Institute (ICVS), School of Medicine, Braga, Portugal

##### **Correspondence**: Louis-David Lord (louis-david.lord@hertford.ox.ac.uk)

*BMC Neuroscience* 2018, **19(Suppl 2):**O7

Brain activity can be understood as the exploration of a dynamical landscape of activity configurations over both space and time. This dynamical landscape may be defined in terms of spontaneous transitions within a repertoire of discrete metastable states of functional connectivity (FC), or “FC states”, which underlie different mental processes. It however remains unclear how the brain’s dynamical landscape might be disrupted in altered states of consciousness, such as the psychedelic state. The present study investigates changes in the brain’s dynamical repertoire in a rare fMRI dataset consisting of healthy participants intravenously injected with the psychedelic compound psilocybin; the active compound in magic mushrooms. We employed a data-driven approach to study brain dynamics in the psychedelic state, which focuses on the dominant FC pattern captured by the leading eigenvector of dynamic FC matrices, and enables the identification of recurrent FC patterns (“FC-states”), and their transition profiles over time. We found that a FC state closely corresponding to the fronto-parietal control system was strongly destabilized by the drug, while transitions toward a globally synchronized FC state were enhanced. These differences between brain state trajectories in normal waking consciousness and the psychedelic state suggest that psilocybin induces an alternative type of unconstrained functional integration at the expense of locally segregated activity specific networks supporting executive function. These results provide a mechanistic perspective on the acute psychological effects of psychedelics, and further raise the possibility that mapping the brain’s dynamical landscape may help guide pharmacological interventions in neuropsychiatric disorders.

## O8 Understanding the bispectrum as a measure of cross-frequency coupling

### Christopher Kovach

#### University of Iowa, Caltech, Iowa City, IA, United States

##### **Correspondence**: Christopher Kovach (christopher-kovach@uiowa.edu)

*BMC Neuroscience* 2018, **19(Suppl 2):**O8

Interest in the origin and significance of cross-frequency coupling in electrophysiological signals has grown rapidly over the last several years, with particular emphasis on phase-amplitude coupling (PAC). Much of this recent attention has focused on measures of PAC obtained from filtered analytic signals through the comparison of phase and analytic envelope. As use of these measures has increased, so has an appreciation of their ambiguities, attested by an expanding cautionary literature on the topic. Meanwhile, “classical’’ statistically motivated measures of cross-frequency coupling derived from spectral representations of higher moments have remained at the periphery of the latest surge of attention, due in large part to a common perception that such measures are comparatively difficult to interpret and that they relate to a form of cross-frequency coupling distinct from PAC. Recently, we have shown that common PAC measures are, in fact, fundamentally normalized bispectral estimators which yield smoothed estimates of the true signal bispectrum [1]. Differences between the measures relate to properties of the respective smoothing kernels. In light of this observation, classical bispectral estimators can claim a number of advantages over recently introduced PAC measures, including more favorable bias properties and freedom from the constraints on range and resolution that are inherent in PAC measures. Interpretation of the bispectrum is commonly explained in terms of ``quadratic’’ phase coupling between spectrally narrow signal components; in demonstrating the relationship to PAC measures, we develop an alternative approach to interpretion through a decomposition of the signal into spectrally broad transient components. The relationship between PAC measures and the bispectrum can be understood by considering the case of a low-frequency transient, corresponding to the ``slow’’ oscillation (SO), accompanied by a transiently windowed high-frequency ``fast’’ oscillation (FO). As detailed in Figures 1 and 2 of reference [1], windowing of the FO at the scale of the SO implies that the bispectrum contains a straightforward representation of the spectrum of the SO and the power spectrum of the FO, from which both might be directly recovered to good approximation. Moreover, within the range of the FO, the phase bispectrum encodes the relative delay between the SO and the FO modulating window. With these insights we develop guidelines for the evaluation of PAC from bispectral statistics. This framework addresses a number of the recently identified limitations and ambiguities of PAC measures. Finally, some extensions of this framework towards the blind recovery of recurring transient signal features are briefly considered. The feasibility of this application is demonstrated through the identification of auditory evoked responses in human intracranial recordings from both controlled stimuli (click trains) and uncontrolled ecologically meaningful stimuli (a video soundtrack) with no foreknowledge of the stimulus.

**Reference**

1. Kovach CK, Ova H, Kawasaki H. The bispectrum and its relationship to phase-amplitude coupling. *Neuroimage* 2018, 173, 518–539

## O9 Spinal interneurons and locomotor speed and gait control in quadrupeds

### Ilya Rybak, Simon Danner, Natalia Shevtsova

#### Drexel University College of Medicine, Department of Neurobiology and Anatomy, Philadelphia, PA, United States

##### **Correspondence**: Ilya Rybak (rybak@drexel.edu)

*BMC Neuroscience* 2018, **19(Suppl 2):**O9

To effectively move in a complex and dynamic environment, limbed animals should vary locomotor speed and adapt gaits to the desired speed and the environment. With increasing locomotor speed, quadrupedal animals, including mice, switch locomotor gait from walk to trot and then to gallop and bound. Centrally, the locomotor gaits are controlled by interactions between four central pattern generators (CPGs) located on the left and right sides of the lumbar and cervical enlargements of the cord, and each producing rhythmic activity controlling one limb. The activity of these CPGs are coordinated by commissural interneurons (CINs), projecting across the midline to the contralateral side of the cord, and by long propriospinal neurons (LPNs) that connect the cervical and lumbar CPG circuits in both directions. We use computational modeling to investigate how the CIN and LPN connections between the cervical and lumbar, left and right CPGs can be organized and what roles different CIN and LPN pathways play in the control and speed-dependent expression of different gaits. Our model contains four rhythm generators (RGs) with left–right cervical and lumbar CIN interactions and homolateral and diagonal ascending and descending LPN interactions. These interactions are organized via several interneuronal pathways mediated by genetically identified neuron types and are based on their suggested functions and connectivity. Supraspinal (brainstem) drives excite all RGs, thereby controlling oscillation frequency, and inhibit some CINs and LPNs, which allows the model to reproduce the speed-dependent gait transitions observed in the intact mice [1]. The model reproduces the experimentally observed loss of particular gaits after selective removal of genetically identified neurons (V2a, V0 V, or all V0) and the speed-dependent disruption of hind limb coordination after deletion of ascending (cervical-to- lumbar) LPNs [2]. The model suggests that (1) V0Dand V0VCINs together secure left–right alternation, whereas V3 CINs promote left–right synchronization, and that (2) V0DLPNs support diagonal alternation, whereas V0VLPNs promote diagonal synchronization. Thus, V0DCINs and LPNs together stabilize walk and V0VCINs and LPNs stabilize trot. The transition from trot to gallop and bound occurs when the activity of V3 CINs overcomes the activity of (brainstem-drive inhibited) V0VCINs and diagonal LPNs. Our simulations have also shown that external inputs to CINs and LPNs, other than supraspinal drives controlling locomotor frequency, can induce gait changes independent of speed. These inputs may represent activities of sensory afferents, which is consistent with multiple experimental data showing that CINs and LPNs receive direct and indirect inputs from sensory afferents. Based on the results of these simulations we suggest that CINs and LPNs represent the main neural targets for different local/intraspinal, supraspinal, and sensory inputs to control interlimb coordination and adjust locomotor gait to various internal and external conditions. The model proposes a series of testable predictions, including the anticipated effects of the deletion of particular identified types of CINs and LPNs, and can be used as a test bed for simulating various spinal cord perturbations and injuries.

References

1. Bellardita C, Kiehn O. Phenotypic Characterization of Speed-Associated Gait Changes in Mice Reveals Modular Organization of Locomotor Networks. Current Biology 2015, 25:1426–1436
2. Ruder L, Takeoka A, Arber S. Long-Distance Descending Spinal Neurons Ensure Quadrupedal Locomotor Stability. Neuron 2016, 92:1063–1078.

## O10 A simplified model of network bursts in the pre-Botzinger complex

### Yury Sokolov, Jonathan Rubin

#### University of Pittsburgh, Department of Mathemathics, Pittsburgh, PA, United States

##### **Correspondence**: Yury Sokolov (ysokolov@pitt.edu)

*BMC Neuroscience* 2018, **19(Suppl 2):**O10

Network (population) bursts are a signature neuronal activity in a critical brainstem region for respiratory rhythm generation, the pre-Botzinger complex (pre-BotC). During the initiation of a network burst, the pre-BotC shows a consistent pattern of dynamic transitions. Starting with mostly silent neurons, the pre-BotC transitions to an intermediate state with a positive fraction of firing neurons that may include tonically spiking and bursting neurons. When a sufficient number of neurons becomes engaged in firing, the pre-BotC network finally undergoes a transition to a population burst, characterized by a high fraction of simultaneously bursting neurons.

Over the last few decades several models of population bursts in the pre-BotC have been proposed, including conductance-based models featuring various ionic currents, such as INaP and ICAN. While the main objective of these models was to identify the bio-physical driving sources underlying network burst initiation, the role of the synaptic connection patterns in shaping neuronal activity has been relatively overlooked. The main reason for this omission is that the models are too complicated for a full analytical treatment and, due to computational limitations, it is difficult to gain full insight into the influence of connectivity. To overcome these obstacles, we propose a simplified model, which is based on a bootstrap percolation process, and is defined as follows. For a given graph, every node has three possible states: inactive, weakly- active, and fully-active, which correspond to silence, tonic spiking and bursting, respectively. We initialize all nodes to the weakly-active state with probability p1 and to the fully-active state with probability p2, independently of other nodes. As the process evolves, an inactive node will transit to the weakly-active state if the amount of activity among its neighbors exceeds a threshold k1, and if the amount is greater than k2, it will transit to the fully-active state. Similarly, a weakly-active node becomes fully-active if the amount of activity among its neighbors exceeds k2. Nodes cannot reduce their activity levels, and those nodes that are fully-active will not change their states until the end of a trial. We analyze this process analytically and computationally on various random graph models and address three questions. First, we determine values p1 and p2 as functions of k1 and k2 for which the network reaches a population burst at the end of a trial. Our findings suggest possible reasons why the network may fail to generate a population burst after the deletion of a fixed fraction of arbitrary nodes in the network, which is consistent with laser ablation of rhythmogenic pre-BotC (Dbx1) neurons in experiments. Second, we investigate how structural features of different graph models affect the duration of the process. Lastly, we describe how using nodal measures we may identify nodes that, when activated initially, are particularly well suited to ignite a population burst. This result shows that local properties of graphs are good descriptors of the spread of bursting activity and also addresses the extent to which successive population bursts may feature similar or different initiation mechanisms.

## O11 Traveling waves in single cortical regions: mechanisms and emerging computational principles

### Lyle Muller, Terrence Sejnowski

#### Salk Institute for Biological Studies, Computational Neurobiology Laboratory (CNL), La Jolla, CA, United States

##### **Correspondence**: Lyle Muller (lmuller@salk.edu)

*BMC Neuroscience* 2018, **19(Suppl 2):**O11

With new multichannel recording technologies, neuroscientists can now record from single cortical regions with high spatial and temporal resolution. Early recordings during anesthesia found spontaneous and stimulus-evoked waves traveling across single cortical regions. For a long time, however, these waves were thought to disappear in awake animals and during high-input regimes. By introducing new signal processing methods for moment-by-moment detection and characterization of spatiotemporal patterns under noise, our recent work has found that small visual stimuli evoke waves traveling out from the point of thalamocortical input to primary visual cortex in the awake monkey [1]. Further, using a measure of directed information transfer across recording sites in V1 of anesthetized monkey, another group has found that traveling waves can influence intracortical dynamics during viewing of natural stimuli [2]. These results indicate that traveling waves can play a role in organizing neural activity during natural sensory processing. Their overall computational role in sensory cortex, however, remains poorly understood. Here, we introduce a spiking model that captures a general network-level mechanism for traveling waves in cortex. We study networks in the self-sustained activity regime [3], where conductance-based networks of neurons can create an internally generated noise [4] consistent with the irregular-asynchronous (IA) background activity state in cortex [5]. We find that a microscopic property—the axonal conduction velocity—profoundly controls the spatiotemporal structure of the spontaneous background state. While previous work has generally considered the time delays from intraregional recurrent fibers to be negligible, these can range up to tens of milliseconds over a few millimeters of the cortical surface, and their inclusion shapes self-sustained activity patterns into spontaneous traveling waves matching those observed in recordings from cortex. By studying networks from 104to 106neurons through a range of connectivity regimes, from very sparse (1 synapses/cell) to that found in cortex (10,0 synapses/cell, [6]), we identify spatiotemporal patterns ranging from*dense waves*, where the fraction of individual neurons participating in a passing wave is nearly unity, to*sparse waves*, where this fraction becomes very low. The sparse wave regime offers a unique operating mode, where many waves can coexist while weakly interacting during their propagation across the network. Finally, in collaboration with the laboratory of John Reynolds (Salk Institute), we show how spontaneous, sparse traveling waves can affect visual processing in the awake marmoset, leading to dynamic shifts in perceptual thresholds.

**References**

1. Muller L, Reynaud A, Chavane F, Destexhe A. The stimulus-evoked population response in visual cortex of awake monkey is a propagating wave. *Nature Communications* 2014, 5.
2. Besserve M, Lowe SC, Logothetis NK, et al. Shifts of Gamma Phase across Primary Visual Cortical Sites Reflect Dynamic Stimulus-Modulated Information Transfer. *PLoS Biology* 2015, 13.
3. Kumar A, Schrader S, Aertsen A, Rotter S. The High-Conductance State of Cortical Networks. *Neural Computation* 28, 20(1), 1–43.
4. Destexhe, C. Neuronal computations with stochastic network states. *Science* 26, 314.
5. Brunel. Dynamics of Sparsely Connected Networks of Excitatory and Inhibitory Spiking Neurons. *Journal of Computational Neuroscience* 20, 8.
6. Braitenberg, S. Cortex: *Statistics and Geometry of Neuronal Connectivity*. Springer Press, 1998.

## O12 Excitable dynamics of NREM sleep: a unifying model for neocortex and hippocampus

### Daniel Levenstein^1^, György Buzsáki^1^, John Rinzel^2^

#### ^1^New York University, Neuroscience Institute, New York, NY, United States; ^2^New York University, Center for Neural Science & Courant Institute of Mathematical Sciences, New York, NY, United States

##### **Correspondence**: Daniel Levenstein (dl2820@nyu.edu)

*BMC Neuroscience* 2018, **19(Suppl 2):**O12

During non-rapid eye movement (NREM) sleep, the neocortex continuously alternates between states of neuronal spiking (UP states) and inactivity (DOWN states). Similarly, the hippocampus also shows continuous alternations between brief periods of neuronal activity (SPW-Rs) and relative inactivity. While the durations of active/inactive states are dramatically different in the two regions, the hippocampus and neocortex are both cortical tissue and are under similar neuromodulatory influence during NREM. Thus, it prompts one to wonder whether the neocortical UP/DOWN states and hippocampal SPW-Rs might be explained by similar mechanisms. Furthermore, the mechanisms by which alternation dynamics in the two regions interact to support NREM function are unclear. To address these questions, we used an idealized firing rate model of UP/DOWN alternations with four distinct dynamical regimes, which are distinguished by the stability or transience of UP/DOWN states and encompass those seen in previous studies. By directly matching model dynamics with experimental observations in naturally-sleeping rats, we found that the alternation dynamics observed in neocortex and hippocampus during NREM reflect two distinct regimes of excitable activity that show characteristically asymmetric durations of UP/DOWN states. Specifically, we find that the neocortical dynamics reflect a stable UP state interrupted by transient DOWN states (slow waves), while the hippocampal dynamics reflect a stable DOWN state with transient UP states (sharp waves).We further considered the effects of including an inhibitory population in the model. We find that under conditions of balanced excitation and inhibition, neocortical UP- > DOWN transitions can be evoked by excitatory input and are followed by a high frequency oscillation at the DOWN- > UP transition, as is observed in vivo. We propose that during NREM sleep, hippocampal and neocortical populations are in excitable states, from which small fluctuations can evoke the transient events that support NREM function. The excitable dynamics we describe suggest a mechanism by which the two structures could show a form of communication through“stochastic synchronization” of spontaneous population events during NREM sleep.

## O13 Biological mechanisms for learning: A computational model of olfactory learning in the Manduca sexta moth

### Charles Delahunt^1^, Jeffrey Riffell^2^, J. Nathan Kutz^1^

#### ^1^University of Washington, Deparment of Applied Mathematics, Seattle, WA, United States; ^2^University of Washington, Department of Biology, Seattle, WA, United States

##### **Correspondence**: Charles Delahunt (delahunt@uw.edu)

*BMC Neuroscience* 2018, **19(Suppl 2):**O13

The moth olfactory network, which includes the antennal lobe (AL), mushroom body (MB), and ancillary structures, is a relatively simple biological neural system that is capable of learning. Its structural features include motifs that are widespread in biological neural systems, such as a cascade of networks, large dimension shifts from stage to stage, sparsity, noise, and randomness. Learning is enabled by a neuromodulatory reward mechanism of octopamine stimulation of the AL, whose increased activity induces rewiring of the MB through Hebbian plasticity. The goal of this work is to analyze how these various components interact to enable learning. To this end, we build a computational model of the moth olfactory network, including the dynamics of octopamine stimulation, which is closely aligned with the known biophysics of the AL-MB and with in vivo AL firing rate data of moths during learning. To our knowledge this is the first full, end-to-end neural network model that demonstrates learning behavior while also closely matching the structure and behavior of a particular biological system. The model is able to robustly learn new odors, and provides a valuable tool for examining the role of octopamine in learning. This octopamine mechanism during learning is of particular interest, since how it promotes the construction of new codes in the MB is not understood. Specifically, our experiments elucidate key biological mechanisms for fast learning from noisy data that rely on an interaction between cascaded networks, sparsity, Hebbian plasticity, and neuromodulatory stimulation by octopamine.

## O14 Modeling of TRP channel mediated noxious cold sensation in Drosophila sensory neurons

### Natalia Maksymchuk, Atit Patel, Nathaniel Himmel, Daniel Cox, Gennady Cymbalyuk

#### Georgia State University, Neuroscience Institute, Atlanta, GA, United States

##### **Correspondence**: Natalia Maksymchuk (nmaksymchuk1@gsu.edu)

*BMC Neuroscience* 2018, **19(Suppl 2):**O14

Intracellular Ca2+ concentration usually correlates with the neuronal pattern and behavioral response. However, noxious cold sensation in Drosophila presents a paradox with these associations. Pkd2 and Trpm channels are required to trigger nociceptive full body contraction (CT) under acute cold [1].*Trpm* mutants exhibit an increase in [Ca2+] levels above control and display reduction of CT behavior, whereas*Pkd2*mutants showed reductions in [Ca2+] level and inhibition of behavior [1]. We developed a Hodgkin-Huxley-type model of the cold sensitive CIII neurons to investigate interaction of Pkd2, Trpm and SK currents and to explain the experimental paradox. Our main mechanism assumes that the mutation of *Trpm* is homeostatically accompanied by a compensatory increase of the total Pkd2 current conductance, which leads to an amplified rise of [Ca2+] under noxious cold temperatures. This higher [Ca2+] activates stronger SK current which hyperpolarizes the membrane potential and suppresses spiking. This leads to inhibition of the stereotyped CT behavior under noxious cold stimuli. This model prediction is supported by the experiments, which showed 2-fold increase of*Pkd2* mRNA levels in Trpm mutants relative to control, while no change in *Trpm* mRNA levels was observed in*Pkd2*mutants.

Basic models of the CIII neuron describing responses of Control, *Trpm* and *Pkd2* mutants show transitions from silence at room temperature to spiking activity below 18 °C, but have distinct features. Models of Control and *Trpm* mutants reach a maximum spike frequency near 14.5 °C, while *Pkd2* mutants exhibited a maximum frequency at 6 °C and had a smaller frequency compared to Control and *Trpm* mutants. The decrease of maximum frequency in *Pkd2* mutants as well as absence of spiking activity for most of the temperature range in *Trpm* mutants may explain the inhibition of CT behavior under noxious cold. The [Ca2+] responses of the three models describing control, *Trpm* and *Pkd2* mutants are in agreement with the corresponding experimental data [1]. [Ca2+] signal of CIII neurons under noxious cold is the strongest in *Trpm* mutants and the weakest in *Pkd2* mutants. Thus, the model and experimental results suggest that cold-evoked CT behavior is tuned to an optimal Ca2+ level which does not always functionally represent level of neuronal excitation. Also, the basic model currently exhibits a wide spectrum of qualitatively different activity regimes. Depending on the parameter set, the model could show different regimes which are associated with different levels of [Ca2+] and could be arranged into an alternative scheme of the temperature coding following the sequence of transitions between regimes: small amplitude spiking, period doubling cascade, bursting, large amplitude spiking, and rest state along with the temperature going down. These two coding schemes provide robust and generic mechanisms of coding modality-specific activity patterns by coordinated modality-specific activation of two TRP currents.

**Acknowledgements**

This research was supported by NIH grant NS086082 and a GSU Brains and Behavior Seed Grant (DNC), N.H. is a Brains and Behavior and Honeycutt Fellow; A.A.P. is a 2CI Neurogenomics and Honeycutt Fellow.

**Reference**

1. Turner HN, Armengol K, Patel AA, et al. The TRP Channels Pkd2, NompC, and Trpm Act in Cold-Sensing Neurons to Mediate Unique Aversive Behaviors to Noxious Cold in Drosophila. *Current Biology* 2016, 26(23), 3116–3128.

## O15 A geometric attractor mechanism for the self-organization of entorhinal grid modules

### Louis Kang^1^, Vijay Balasubramanian^2^

#### ^1^University of California, Berkeley, Redwood Center for Theoretical Neuroscience, Berkeley, CA, United States; ^2^University of Pennsylvania, Computational Neuroscience Initiative, Philadelphia, PA, United States

##### **Correspondence**: Louis Kang (louis.kang@berkeley.edu)

*BMC Neuroscience* 2018, **19(Suppl 2):**O15

The grid system of the mammalian medial entorhinal cortex (mEC) exhibits striking modularity. Rat grid cell recordings reveal that spatial grid scales cluster around discrete values separated by constant ratios reported in the range 1.3–1.8. Although this modular organization has been shown to be a robust and efficient encoding of spatial location, its origin is unknown. We present the first proposed mechanism through which geometric sequences of grid scales arise naturally. A series of continuous attractor networks along the longitudinal mEC axis that would otherwise generate a smooth distribution of grid scales forms modules separated by discrete jumps in scale when excitatory connections are introduced (Fig. 1). Moreover, constant scale ratios between successive modules arise through robust geometric relationships between commensurate triangular grids, whose lattice constants are separated by [sqrt(1.7)] or other ratios, or between grids containing local lattice modulations called discommensurations. These relationships persist in single neuron spatial rate maps due to faithful path integration and are unaffected by perturbations to model parameters. We speculate on how excitatory connections between attractor networks can be realized by the known architecture of the mEC and suggest analyses and experiments that test our model.

**Fig. 1:**
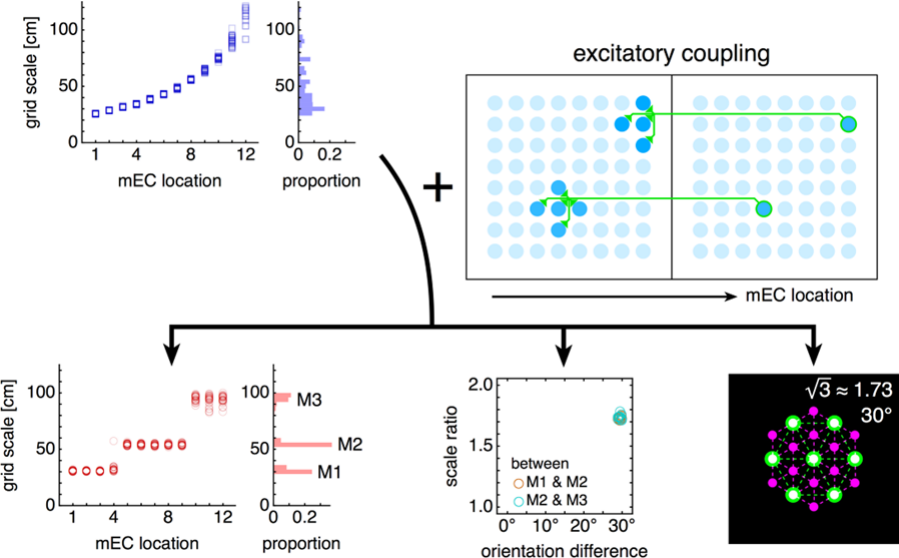
Grid cells with smoothly distributed scales self-organize into discrete modules when excitatory connections along the medial entorhinal cortex (mEC) are added. Adjacent modules have fixed scale ratios and orientation differences due to robust geometric relationships between commensurate triangular lattices

## O16 Simulating in vivo context-dependent recruitment of CA1 hippocampal interneuron specific 3 (IS3) interneurons

### Alexandre Guet-McCreight, Frances Skinner

#### ^1^Krembil Research Institute, Division of Fundamental Neurobiology, Toronto, Canada

##### **Correspondence**: Alexandre Guet-McCreight (agmccrei@gmail.com)

*BMC Neuroscience* 2018, **19(Suppl 2):**O16

Obtaining recordings from individual cells during behaviour is technically challenging, especially for the diverse interneuron subtypes that tend to be smaller, less accessible, and less identifiable relative to excitatory cells. As such, it is difficult to determine inhibitory cell contributions but it is clear that consideration of interneuron subtypes is critical to understanding brain function and behavior [3]. To address this, we use computational approaches. We focus on the hippocampal CA1 interneuron specific 3 (IS3) cell, a cell type that has not yet been recorded from in vivo. Notably, though IS3 cells represent a small fraction of interneurons in CA1 hippocampus, they possess unique circuitry properties in that they only inhibit other inhibitory neurons, such as Oriens Lacunosum Moleculare (OLM) interneurons. In vitro, photo-activation of IS3 cells at theta frequencies has been shown to elicit theta-timed spiking in OLM cells [4]. To explore the potential contributions of IS3 cells during in vivo contexts, we use multi-compartment IS3 cell models to generate predictions of input populations that could either enhance or dampen IS3 cell activities during behavior. We have developed data-driven multi-compartment models of IS3 cells with active dendritic properties [1], determined realistic synaptic parameters along the dendritic morphology of the models [2], and estimated numbers of active synapses and presynaptic spike rates to generate in vivo-like states for IS3 cell models. Here, we consider context-dependent recruitment of IS3 cells during simulated states of theta rhythms and sharp-wave associated ripples (SWRs). During these states, we use our models to predict the contributions of different presynaptic inhibitory and excitatory input populations.

Our results show that excitatory theta-timed inputs from CA3 and entorhinal cortex can modulate the timing of IS3 cell spiking during theta rhythms. Moreover, depending on their relative contributions, the timing of the IS3 cell model’s spiking can occur anywhere between the rising phase and peak of the theta cycle. As well, we show that inhibitory inputs can dampen spike recruitment of IS3 cells regardless of phase, though less so for inhibitory inputs that are the most antiphase relative to excitatory inputs. For our simulated SWR context, we show that transiently bursting CA3 inputs alone are sufficient to recruit the IS3 cell model to spike. We also show that the presence of feedforward inhibition on the proximal dendrites of the model can sufficiently dampen IS3 cell spiking during a SWR context. In summary, we have simulated in vivo-like contexts where IS3 cell spike recruitment can be either enhanced or dampened. Our results highlight possible IS3 cell spiking scenarios and thus their potential contributions to brain function and behavior.

**References**

1. Guet-McCreight A, et al. Using A Semi-Automated Strategy To Develop Multi-Compartment Models That Predict Biophysical Properties Of Interneuron Specific 3 (IS3) Cells In Hippocampus. *eNeuro*. 2016, 3(4). pii: ENEURO.87-16.2016.
2. Guet-McCreight A, et al. F10Research 2017, 6:1552 (poster).
3. Kepecs A, Fishell G. Interneuron cell types are fit to function. *Nature* 2014, 505(7483):318–26.
4. Tyan L, et al. Dendritic inhibition provided by interneuron-specific cells controls the firing rate and timing of the hippocampal feedback inhibitory circuitry. *J Neurosci*. 2014, 34(13):4534–47.

## O17 Quantitative simplification of detailed microcircuit demonstrates the limitations to common point-neuron assumptions

### Christian A Rössert^1^, Giuseppe Chindemi^1^, Andrew Davison^2^, Dimitri Rodarie^1^, Nicolas Perez Nieves^3^, Christian Pozzorini^1^, Csaba Eroe^1^, James King^1^, Taylor Newton^1^, Max Nolte^1^, Srikanth Ramaswamy^1^, Michael Reimann^1^, Willem Wybo^1^, Marc-Oliver Gewaltig^1^, Wulfram Gerstner^1^, Henry Markram^1^, Idan Segev^4^, Eilif Muller^1^

#### ^1^École Polytechnique Fédérale de Lausanne, Blue Brain Project, Lausanne, Switzerland; ^2^CNRS, Unité de Neuroscience, Information et Complexité, Gif sur Yvette, France; ^3^Imperial College London, Department of Physics, London, United Kingdom; ^4^Hebrew University of Jerusalem, Department of Neurobiology, Jerusalem, Israel

##### **Correspondence**: Eilif Muller (eilif.mueller@epfl.ch)

*BMC Neuroscience* 2018, **19(Suppl 2):**O17

A first-draft detailed simulation of a piece of the rat neocortex has recently been reported by an international collaboration [1]. This work integrated the current state of experimental knowledge on the detailed 3D anatomy and physiology of the various neuron types, and their synaptic properties and connectivity, and was shown to reproduce findings from a range of in vivo experiments reported in the literature without parameter tuning. On the other hand, for large-scale network simulations, point-neuron models are typically used for describing and analyzing network dynamics and functions. The properties and connectivity structure of point neuron models generally are not constrained by biological data and thus use ad hoc simplifying assumptions. This makes some of the mathematically tractable models somewhat disconnected from experimental neuroscience. To bridge the gap between these two extremes (the detailed and the oversimplified), we aimed to derive point-neuron network models from data-driven detailed network models in an automated, repeatable and quantitatively verifiable manner. The simplification occurs in a modular workflow, in an in vivo-like state. First, synapses are displaced from dendrites to the soma while correcting for dendritic filtering using low-pass filters for the synaptic current numerically calibrated for each dendritic compartment. Next, point-neuron models for each neuron in the microcircuit are fitted to their respective morphologically detailed counterparts. Here, generalized integrate-and-fire point neuron models are used, leveraging a recently published fitting toolbox [2]. The fits are constrained by currents and voltages computed in the morphologically detailed reference neurons with soma-displaced synapses, as described above. Benchmarking the simplified network model to the detailed microcircuit model for a range of simulated in vivo and in vitro protocols, we found good agreement for both quantitative and qualitative aspects. Our automated approach not only makes it possible to continuously update the simplified circuit as the detailed network integrates new data, but the modularity of the simplification process also makes it applicable to other point neuron and synapse models, network models, and simulators. In addition to providing an extensive assessment of validity for carefully reduced point neuron network models, our approach is fundamentally important and informative, in particular in cases when network functionalities are lost during the simplification pipeline. By taking the simplification further to evaluate common simplifying assumptions, we further illustrate the contributions of specific synaptic and cellular dynamics to the overall response of the detailed network, revealing limitations for several common approaches.

**References**

1. Markram H, Muller E, Ramaswamy S, et al. Reconstruction and Simulation of Neocortical Microcircuitry. *Cell* 2015, 163(2), 456–492.
2. Pozzorini C, Mensi S, Hagens O, et al. Automated High-Throughput Characterization of Single Neurons by Means of Simplified Spiking Models.*PLOS Computational Biology* 2015, 11(6)

## O18 A novel synaptic plasticity rule for detailed model neurons with realistic dendrites

### Christian Ebner^1^, Claudia Clopath^2^, Peter Jedlicka^3^, Hermann Cuntz^4^

#### ^1^Ernst Strüngmann Institute, Frankfurt, Germany; ^2^Imperial College London, Department of Bioengineering, London, United Kingdom; ^3^Justus Liebig University, Faculty of Medicine, Giessen, Germany; ^4^Frankfurt Institute for Advanced Studies (FIAS) & Ernst Strüngmann Institute (ESI), Computational Neuroanatomy, Frankfurt/Main, Germany

##### **Correspondence**: Peter Jedlicka (peter.jedlicka@informatik.med.uni-giessen.de)

*BMC Neuroscience* 2018, **19(Suppl 2):**O18

Numerous experiments have been conducted in the past in order to monitor the complex interactions that drive activity-dependent long-term plasticity of synapses. Spike timing, firing rate and synaptic location have been found to be important factors that dynamically contribute to the outcomes of plasticity induction protocols. While several theoretical models that implement plasticity rules already exist, they have not yet been used in depth to study plasticity in neuron models with detailed morphology. Here, we extend previous phenomenological voltage-based plasticity rules by developing a new framework based on three signaling pathways. We apply it to a L5 pyramidal cell model with active dendritic properties and realistic propagation of voltage. We show that our novel rule not only reconciles outcomes of several experiments but also predicts spatiotemporal patterns of plasticity that are characteristic for individual stimulation protocols and their impact on local processes at the synapse, including protocols inducing local plasticity in tuft dendrites. Due to this focus on local voltage signals, our framework can explain synaptic plasticity in the absence of postsynaptic action potentials, as suggested in recent studies. We thereby link experimental results that would intuitively seem to require entirely different rules, showing that a unifying rule might explain the vast majority of experiments in cortical pyramidal cells if key biophysical pathways are taken into account. Ultimately, we can now study how the cell-type specific electrotonic properties can explain differences in emerging plasticity by incorporating our plasticity rule in a variety of existing detailed compartmental models such as models of hippocampal pyramidal or granule cells. To summarize, a simple plasticity rule that utilizes pre- and postsynaptic plasticity pathways can explain experimental results with a large variety of induction protocols when the plasticity rule is incorporated in the compartmentalized structure of a detailed dendritic model.

## O19 Assisted construction of hybrid circuits: making easy the implementation and automation of interactions between living and model neurons

### Manuel Reyes-Sanchez, Irene Elices Ocon, Rodrigo Amaducci, Francisco B Rodriguez, Pablo Varona

#### Universidad Autónoma Madrid, Ingeniería Informática, Madrid, Spain

##### **Correspondence**: Irene Elices Ocon (irene.elices@uam.es)

*BMC Neuroscience* 2018, **19(Suppl 2):**O19

Closed-loop interactions with the nervous system are a powerful approach to characterize neural dynamics and control network functions [1, 2]. In particular, neuron models can interact with living neurons in hybrid circuits once proper adaptation is achieved in both directions [3, 4]. Such adaptations are not easy to accomplish in a manual trial-and-error process, and are better determined with closed-loop protocols based on real-time event detection [5] and well-defined interaction goals and performance measurements. This work presents a set of algorithms for the assisted construction of hybrid circuits. These algorithms have been implemented in RTHybrid, an open-source cross-platform real-time model library [6]. Our real-time algorithms for assisted construction of hybrid circuits are based in a general closed-loop paradigm designed to be modular and effective. The algorithms perform as a function of their online measured input parameters the following tasks: (1) temporal and amplitude scaling, (2) drift compensation, (3) synaptic tuning/calibration, (4) model turning/calibration, (5) automatic activity control, (6) automatic mapping of the dynamics. The temporal and amplitude scales are evaluated and matched online to create compatible working regimes between the model and living neurons [4]. All protocols use three steps: event detection, activity and connection characterization and target performance evaluation. The events detected online include: spikes, bursts, hyperpolarization intervals, voltage ranges, temporal structures, phases, etc. The interaction characterization measures include event timings, instantaneous periods, synchronization levels, target phases, and working/dynamic range assessments. When the interaction goal is not fulfilled, the target evaluator algorithm changes in an informed and automatic manner the parameters of the hybrid circuit. Our algorithms have been validated in a hybrid circuit to study the presence of dynamical invariants in CPGs. In conclusion, hybrid circuits require experiment-specific adaptations to work properly, and the parameters of the implementation must be evaluated dynamically on each preparation and even adapted during the same experiment. These algorithms can also be used to automatically map the parameter space to achieve a given goal, and in general to control/explore/unveil bifurcations and circuit dynamics.

**Acknowledgements**

We acknowledge support from MINECO/FEDER DPI2015-65833-P, TIN2014-54580-R, TIN2017-84452-R (http://www.mineco.gob.es/) and ONRG grant N62909-14-1-N279.

**References**

1. Chamorro P, Muñiz C, Levi R, Arroyo D, Rodríguez FB, Varona P. *PLoS ONE* 2012, 7(7).
2. Elices I, Varona P. 2015, 170, 55–62.
3. Ambroise M, Buccelli S, Grassia F, Pirog A, Bornat Y, Chiappalone M, et al. *Artif*. *Life Robot* 2017, 22, 398–403.
4. Reyes-Sanchez M, Elices I, Amaducci R, Muñiz C, Rodríguez FB, Varona P. *BMC Neuroscience* 2017, 18 (Suppl 1):P281 (CNS 2017).
5. Varona P, Guardeño DA, Nowotny T, Rodríguez FB. 2017. Online event detection requirements in closed-loop neuroscience. In *Closed Loop Neuroscience* (pp. 81–91).
6. Amaducci R, Muñiz C, Reyes-Sanchez M, Rodríguez FB, Varona P. *BMC Neuroscience 2017*, 18 (Suppl 2):P104 (CNS 2017).
7. Elices I, Arroyo D, Levi R, Rodríguez FB, Varona P. *BMC Neuroscience 2017*, 18 (Suppl 1):P282 (CNS 2017).

## O20 Deciphering the evolutionary route to the first neurons

### Oltman De Wiljes^1^, Ronald Van Elburg^2^, Fred Keijzer^1^

#### ^1^University of Groningen, Theoretical Philosophy, Groningen, Netherlands; ^2^University of Groningen, Faculty of Science and Engineering, Groningen, Netherlands

##### **Correspondence**: Oltman De Wiljes (otdewiljes@gmail.com)

*BMC Neuroscience* 2018, **19(Suppl 2):**O20

We do not yet know how the very first nervous systems and their constituting neurons evolved within the animal kingdom. One important difficulty comes from the lack of examples of intermediate neuronal stages within currently existing animals. Such examples would bridge the gap between non-neuronal and neuronal configurations. However, on the one hand there are basic animals like sponges and placozoa who do not have neurons or a nervous system. On the other hand, even the most basic forms of animals with nervous systems, such as jellyfish (cnidarians) and comb-jellies (ctenophores) already exhibit a nervous system built from complete neurons. So far it is unknown how the three fundamental ingredients of modern neurons—electrical signaling, synapses, and neuronal elongations—came together in the first neurons and why this happened. Compared to modern animals, very little is known about the earliest possessors of nervous systems. Essentially modern nervous systems complete with eyes and a central nerve cord are known from the beginning of the Cambrian period, so the very origin of nervous systems must predate that period. However, Precambrian animal fossils are enigmatic and difficult to interpret, providing insufficient information about the behavioural and neuronal makeup of these organisms. Molecular phylogenetic studies do provide important clues concerning the cellular building blocks present to these animals but do not allow a clear view of the organization of the animals living in these times. Computational neuroscience provides an important additional instrument to enhance our understanding of the neuronal and behavioural mechanisms that were potentially present in very early animals. Modelling very basic animal configurations, using primitive features such as cell-to-cell signalling that can be assumed to have been present at this stage, provides a way to assess the behavioural capacities of such configurations. Such modelling also allows a step by step investigation of potential evolutionary sequences of various proto-neuronal features and the behavioural effects they induce. All in all, these models provide rigorous thought experiments that enable a systematic investigation of various (proto-)neuronal features on coordination in a simple body (Fig. 1).

**Fig. 1:**
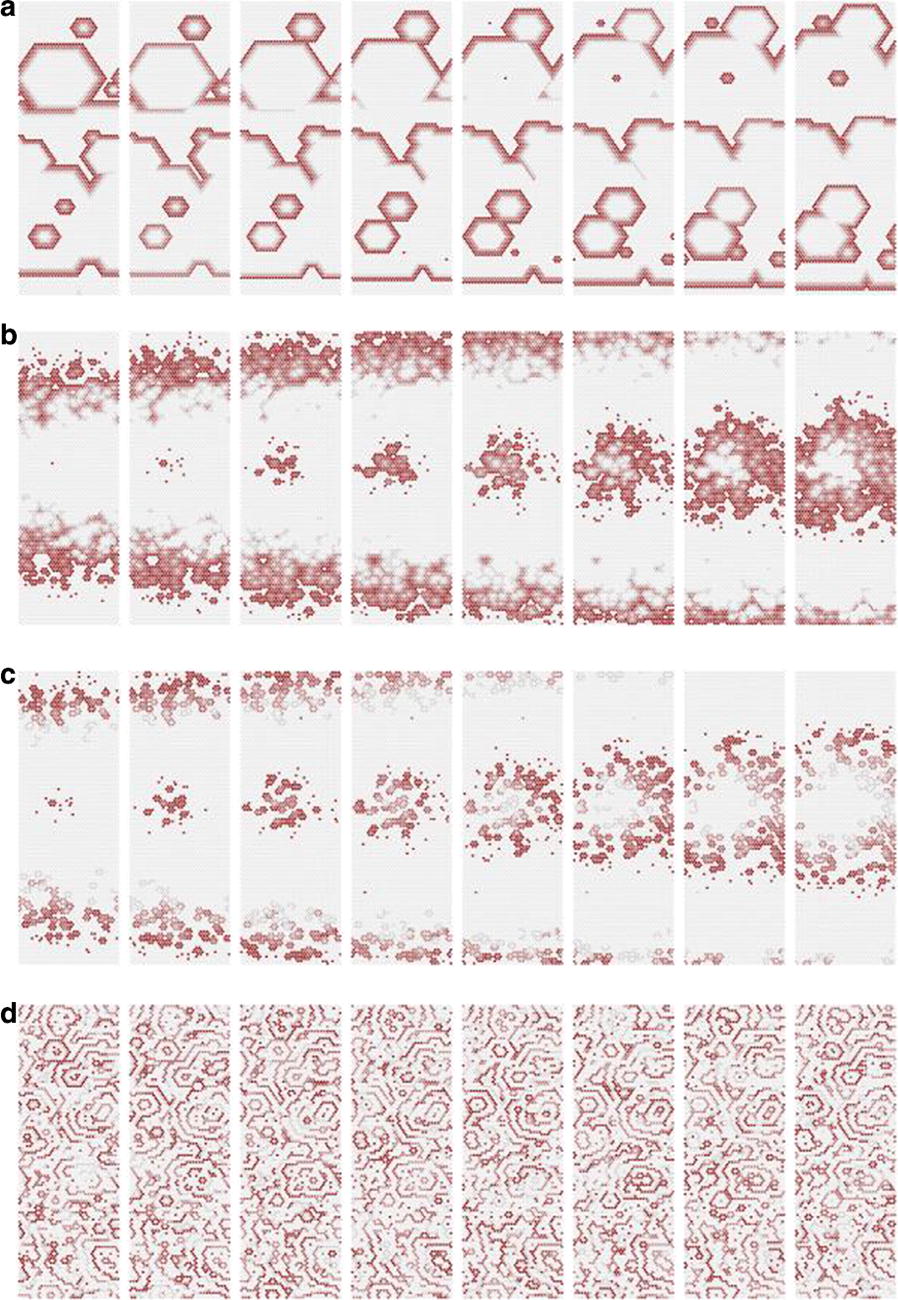
Various degrees of emergent coordination on a larger (32 cells in circumference, 128 in length) worm-shaped body. Four different experiments, showing 8 frames each: A, lacking elongations; B, 10% of cells exhibiting elongations; C, same as B, but no nearest-neighbour connections between cells lacking elongations; D, same as B, but with very low transmission speed

## O21 Community models as the ultimate objective (and success) of computational neuroscience: exempli gratia: The cerebellar Purkinje cell

### James Bower

#### Southern Oregon University, Department of Biology, Ashland, OR, United States

##### **Correspondence**: James Bower (bowerj@sou.edu)

*BMC Neuroscience* 2018, **19(Suppl 2):**O21

On its main web page, the Organization for Computational Neuroscience (OCNS) defines Computational Neuroscience as “the study of brain function in terms of the information processing properties of the structures that make up the (sic) nervous system”. As nervous systems ARE information processing structures, this definition begs the question how the field of Computational Neuroscience distinguishes itself from neuroscience as a whole? The definition of Computational Neuroscience provided by OCNS makes an effort to addresses this conundrum by further defining CNS as “an interdisciplinary science that links the diverse fields of neuroscience, cognitive science and psychology with electrical engineering, computer science, mathematics and physics.” In this presentation, I will propose that THE key concept underlying Computational Neuroscience is, in fact, the question of ‘linkage’. More specifically, I will propose that ‘linkage’ should not be an abstract ideal, but instead, specifically requires the development of computation tools and devices as well as an attitude towards science that supports the development of “community models’ defined as actual mathematical models shared and developed collaboratively across the community of those interested in a particular neuronal feature or component. While one can argue that standards for academic advancement and the current publication process favor isolated models developed by individual research groups which therefore, continue to dominate computational neuroscience, I will suggest that only shared community models can truly support scientific communication, coordination and collaboration. Further, of necessity, to be effective I will assert that these community models must be ‘realistic’, reflecting the actual physical and physiological structure of the components of the nervous systems being studied. Not only do community models of this type provide a basis for real collaboration, they also, in effect, represent the current state of our understanding of neuronal structure/function relationships mathematically. In this presentation, these assertions will be considered with respect to the development over the last 40 years of a model of the cerebellar Purkinje cell as one of the first computational models used across multiple laboratories as well as the historical context provided by the emergence of ‘realistic’ community models in Physics in the 16th century (Fig. 1). In a companion submission, I will consider, with several of my long-term colleagues, how the development of shared simulation platforms when combined with a new approach to scientific publication can drive the development and use of community models.

**Fig. 1:**
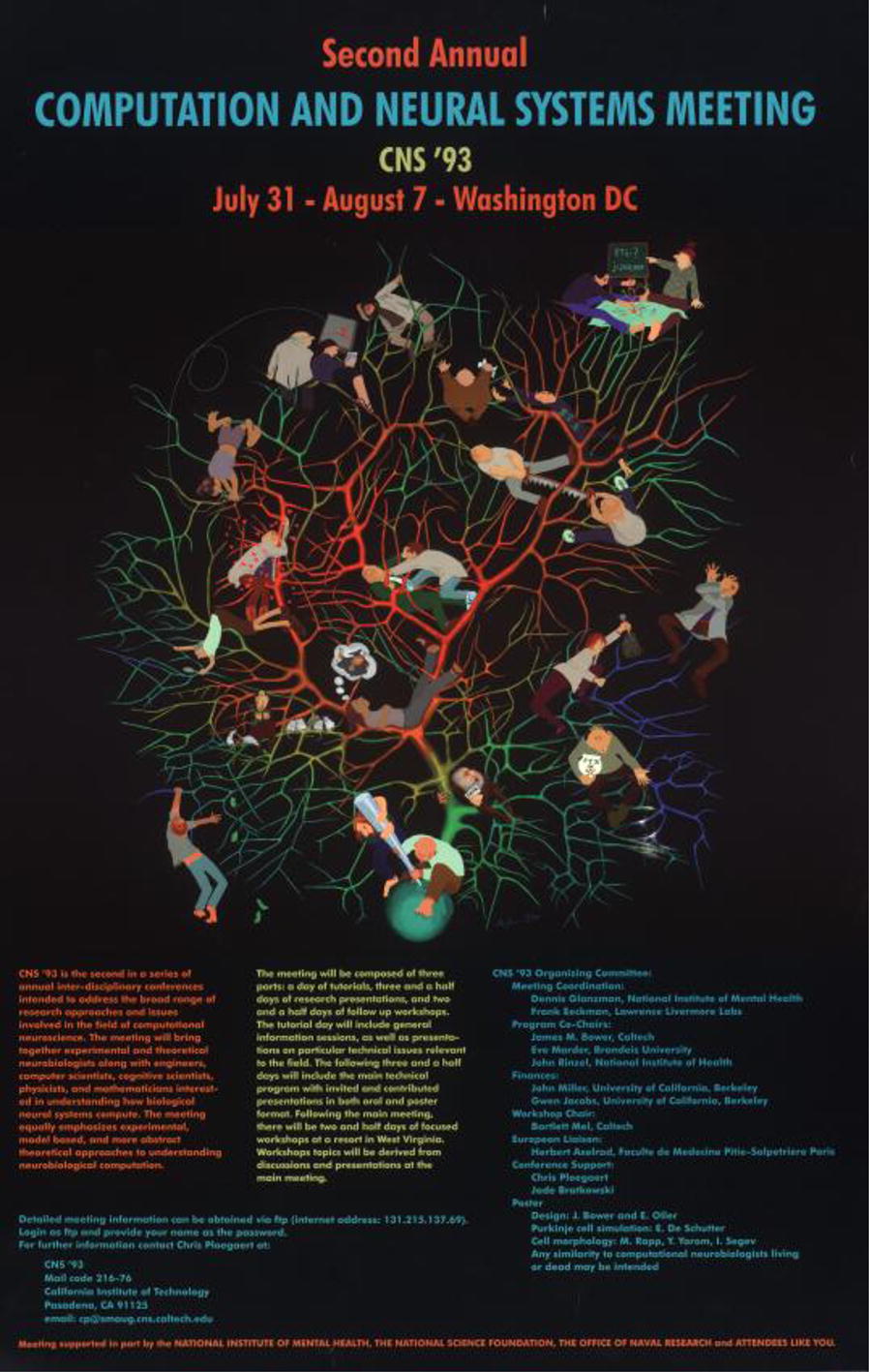
The first poster created for the CNS meeting, intended to represent the initial somewhat disorganized state of the field

## P1 MRI2MRI: A fully convolutional deep artificial network algorithm that accurately transforms between brain MRI contrasts

### Ariel Rokem^1^, Sa Xiao^2^, Yue Wu^2^, Aaron Lee^2^

#### ^1^University of Washington, eScience Institute, Seattle, WA, United States; ^2^University of Washington, Department of Ophthalmology, Seattle, WA, United States

##### **Correspondence**: Ariel Rokem (arokem@gmail.com)

*BMC Neuroscience* 2018, **19(Suppl 2):**P1

Many types of measurements of brain signals rely on the generation of images of brain tissue. For example, magnetic resonance imaging (MRI) measurements can non-invasively image brain tissue in vivo at millimeter resolution. One of the strengths of MRI is that it uses many different kinds of pulse sequences to generate images that emphasize different contrasts between parts of the brain that have different physical and physiological properties, leading to a wealth of different applications in scientific research and in clinical practice. But while different brain MRI contrasts represent different tissue properties and are sensitive to different artifacts, the relationship between different contrasts is complex and nonlinear. We developed a deep fully convolutional artificial neural network that learns the mapping between different MRI contrasts (Fig. 1). Using a publicly available dataset, we demonstrate that this algorithm accurately transforms between T1- and T2-weighted images, proton density images, time-of-flight angiograms, and diffusion MRI images. We demonstrate that these transformed images can be used to improve spatial registration between MR images of different contrasts.The successful transformation between image types also suggests that the physical and physiological relationships between brain tissue properties indexed by the different contrasts could be elucidated in future work.

**Fig. 1:**
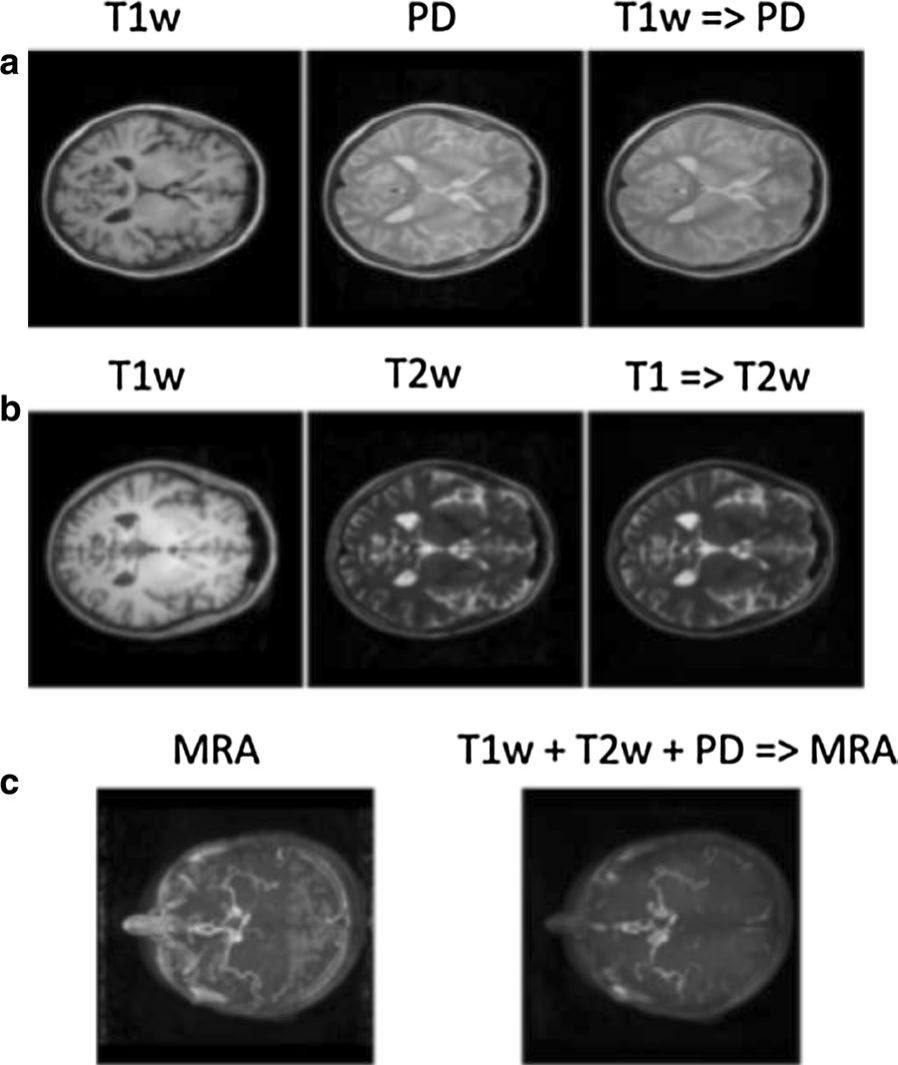
MRI2MRI learns the mapping between different imaging contrasts. (A) Synthesis of PD from T1w: a horizontal slice in one individual brain not shown to the algorithm during training, demonstrating that the algorithm generalizes outside of the training set. Left: input T1w; center: ground truth PD; right: synthesized PD. (B) Synthesis of T2w from T1w: input T1w (left), ground truth T2w (center) and synthesized T2w (right). (C) A many-to-one mapping: combination of T1w, T2w and PD used to synthesize MRA of an individual brain not used during training. The maximum intensity projection along the axial dimension for ground truth (left) and synthesized MRA (right)

## P2 Closing the loop between neural network simulators and the OpenAI Gym

### Philipp Weidel^1^, Jakob Jordan^2^, Abigail Morrison^1^

#### ^1^Jülich Research Centre, Institute for Advanced Simulation (IAS-6), Juelich, Germany; ^2^University of Bern, Department of Physiology, Bern, Switzerland

##### **Correspondence**: Philipp Weidel (p.weidel@fz-juelich.de)

*BMC Neuroscience* 2018, **19(Suppl 2):**P2

Since the enormous breakthroughs in machine learning over the last decade, functional neural network models are of growing interest for many researchers in the field of computational neuroscience. One major branch of research is concerned with biologically plausible implementations of reinforcement learning, with a variety of different models developed over the recent years. However, most studies in this area are conducted with custom simulation scripts and manually implemented tasks. This makes it hard for other researchers to reproduce and build upon previous work and nearly impossible to compare the performance of different learning architectures.

In this work, we present a novel approach to solve this problem, connecting benchmark tools from the field of machine learning and state-of-the-art neural network simulators from computational neuroscience. This toolchain enables researchers in both fields to make use of well-tested high-performance simulation software supporting biologically plausible neuron, synapse and network models and allows them to evaluate and compare their approach on the basis of a curated set of standardized environments of varying complexity. We demonstrate the functionality of the toolchain by implementing a neuronal actor-critic architecture for reinforcement learning in the NEST simulator [1], successfully training it on two different environments from the OpenAI Gym [2] and comparing its performance to a previously known model of reinforcement learning in the basal banglia [3] and a standard Q-learning algorithm [4].

**Acknowledgements**

We acknowledge partial support by the German Federal Ministry of Education through our German-Japanese Computational Neuroscience Project (BMBF Grant 01GQ1343), EuroSPIN, the Helmholtz Alliance through the Initiative and Networking Fund of the Helmholtz Association and the Helmholtz Portfolio theme “Supercomputing and Modeling for the Human Brain” and the European Union Seventh Framework Programme (FP7/2007–2013) under grant agreement no. 604102 (HBP). All network simulations carried out with NEST (http://www.nest-simulator.org).

**References**

1. Gewaltig, MO, Diesmann M. NEST (NEural Simulation Tool). *Scholarpedia* 2007, 2(4), 1430
2. Brockman G, Cheung V, Pettersson L, et al. (2016). *OpenAI Gym*. ArXiv e-prints
3. Jitsev J, Morrison A, Tittgemeyer M. “Learning from positive and negative rewards in a spiking neural network model of basal ganglia.” *Neural Networks* 2012, (IJCNN).
4. Tesauro G. Temporal difference learning and TD-Gammon. *Communications of the ACM* 1995, 38(3), 58–68.

## P3 Reproducing polychronization: a guide to maximizing the reproducibility of spiking network models

### Robin Pauli^1^, Philipp Weidel^1^, Susanne Kunkel^2^, Abigail Morrison^1^

#### ^1^Jülich Research Centre, Institute for Advanced Simulation (IAS-6), Juelich, Germany; ^2^Norwegian University of Life Sciences, Faculty of Science and Technology, Ås, Norway

##### **Correspondence**: Philipp Weidel (p.weidel@fz-juelich.de)

*BMC Neuroscience* 2018, **19(Suppl 2):**P3

Any modeler who has attempted to reproduce a spiking neural network model from its description in a paper has discovered what a painful endeavor this is. Even when all parameters appear to have been specified, which is rare, typically the initial attempt to reproduce the network does not yield results that are recognizably akin to those in the original publication. Causes include inaccurately reported or hidden parameters (e.g. wrong unit or the existence of an initialization distribution), differences in implementation of model dynamics, and ambiguities in the text description of the network experiment. The very fact that adequate reproduction often cannot be achieved until a series of such causes have been tracked down and resolved is in itself disconcerting, as it reveals unreported model dependencies on specific implementation choices that either were not clear to the original authors, or that they chose not to disclose. In either case, such dependencies diminish the credibility of the model’s claims about the behavior of the target system. To demonstrate these issues, we provide a worked example of reproducing a seminal study [1] for which, unusually, source code was provided at time of publication. Despite this seemingly optimal starting position, reproducing the results was time consuming and frustrating. From this process, we derive a guideline of best practices that would substantially reduce the investment in reproducing such a study. We propose that these guidelines can be used by authors and reviewers to assess and improve the reproducibility of future network models.

**Acknowledgements**

We acknowledge the Initiative and Networking Fund of the Helmholtz Association, the Helmholtz Association through the Helmholtz Portfolio Theme”Supercomputing and Modeling for the Human Brain”, the German Research Foundation (DFG; KFO 219, TP9) and the European Union’s Horizon 2020 research and innovation programme (HBP SGA1, grant no. 720270 and no. 754304). We thank P. Quaglio, G. Trensch and R. Gutzen for fruitful discussions.

**Reference**

1. Izhikevich EM. Polychronization: Computation with spikes. *Neural Computation* 2006, 18, 245–282.

## P4 Localization of coherent activity based on multi-electrode local field potentials

### Robin Pauli, Tom Tetzlaff, Abigail Morrison

#### Jülich Research Centre, Institute for Advanced Simulation (IAS-6), Juelich, Germany

##### **Correspondence**: Robin Pauli (r.pauli@fz-juelich.de)

*BMC Neuroscience* 2018, **19(Suppl 2):**P4

Deep brain stimulation (DBS) of the subthalamic nucleus (STN) can suppress pathological oscillations and alleviate motor deficits in Parkinson’s disease. The efficacy and the extent of side effects of DBS depend critically on the positioning of the stimulation electrode. In particular, with the increased use of directional DBS, it is becoming increasingly difficult to find optimal stimulation parameters. A major challenge during the positioning of DBS electrodes is the detection of hotspots associated with the generation of pathological coherent activity. Here, we develop and test a method for localizing confined regions of coherent activity based on the local field potential (LFP) recorded with multi-contact electrodes. Our approach involves two steps, the identification of coherent sources by independent-component analysis of the multi-channel recordings in Fourier space, and the localization of identified sources by means of current-source-density analysis. We benchmark this technique for a range of source sizes and source-electrode distances based on synthetic ground-truth data generated by a simple LFP model. In this context, sources of coherent activity can be reliably localized even if the source center is not contained in the volume covered by the electrode grid. The proposed method permits a continuous tracking of source positions, and may therefore provide a tool to study the spatio-temporal organization of pathological activity in STN. Moreover, it could serve as an intra-operative guide for the positioning of DBS electrodes, and thereby improve and speed up both the implantation process and the adjustment of stimulus parameters.

**Acknowledgements**

Funded by the Initiative and Networking Fund of the Helmholtz Association, the German Research Foundation (DFG; KFO 219, TP9) and the European Union’s Horizon 2020 research and innovation programme (HBP SGA1, grant no. 720270).

## P5 Exploring the role of striatal D1-MSNs and D2-MSNs in action selection using a robotic framework

### Jyotika Bahuguna, Philipp Weidel, Abigail Morrison

#### Jülich Research Centre, Institute for Advanced Simulation (IAS-6), Juelich, Germany

##### **Correspondence**: Jyotika Bahuguna (j.bahuguna@fz-juelich.de)

*BMC Neuroscience* 2018, **19(Suppl 2):**P5

The classical firing rate model of basal ganglia suggests that the “Go” pathway facilitates a movement, whereas the “No-go” pathway suppresses a movement. Strong evidence for this hypothesis was provided by the demonstration that selective optogenetic stimulation of D1-MSNs in mice leads to increased ambulation, whereas optogenetic stimulation of D2-MSNs leads to freezing [1]. However, it has also been shown that D1- and D2-MSNs co-activate in freely moving mice during action initiation [2], which suggests a co-operative rather than an antagonistic role for these pathways. In order to systematically investigate the individual and interactive roles of D1- and D2-MSNs in action selection, it is necessary to be able to both record D1- and D2-MSNs in the same animal, and selectively record and manipulate the action encoding neurons. Because this is beyond present experimental techniques, we investigate this issue with the help of a hybrid spiking neuronal network/virtual robot model. The advantage of this approach is that D1- and D2-MSNs can be observed/manipulated on the single channel and population levels whilst the effect of these manipulations can be observed as the trajectories of the robot, thereby bridging the gap between striatal recordings and behavioral expression. We first demonstrate that our model can reproduce the main features of several key motor studies employing optogenetic manipulation, such as freezing, increased ambulation [1] and ipsilateral turning [3]. We then test the hypothesis that D1- and D2- MSNs are competitive within a channel but cooperative on a population level. Our results show that in opposition to our original hypothesis, D1- and D2-MSNs co-operate within a channel and compete between channels. In this co-operative tandem, D1-MSNs drive the action execution while D2-MSNs suppress the competing actions. Although the co-operation between D1- and D2-MSNs within a channel is facilitated by distance dependent connectivity, an external stimulation to both populations is required in order to exhibit a concurrent activation on population level as observed in experiments [2]. We also show that D2-D2 connectivity is crucial for the competition between the channels. Furthermore, we show that individual pairs of D1- and D2-MSNs compete or co-operate depending on the distance between their originating channels and stimulation paradigms.

**Acknowledgements**

This work was inspired by the debate”Direct and indirect pathways: Friends or Foes?” at IBAGS 2017. Funded by German Research Foundation (DFG; grant DI 1721/3-1 [KFO219-TP9]), the Helmholtz “Supercomputing and Modeling for the Human Brain” (SMHB) Initiative and Networking Fund of the Helmholtz Association and Ger-Jpn Comput Neurosci Project, German Federal Ministry for Education and Research (BMBF Grant 01GQ1343).

**References**

1. Kravitz AV, Freeze BS, Parker PR, et al. Regulation of parkinsonian motor behaviours by optogenetic control of basal ganglia circuitry. *Nature* 2010, 466(7306), 622–626.
2. Cui G, Sang Beom J, Jin X, et al. Concurrent activation of striatal direct and indirect pathways during action initiation. *Nature* 2013, 494(7436), 238-42.3. Tecuapetla et al., Nature Communications 2014, 5:4315.
3. Tecuapetla F, Matias S, Dugue GP, et al. Balanced activity in basal ganglia projection pathways is critical for contraversive movements *Nature Communications* 2014, 5:4315.

## P6 Calcium imaging spike deconvolution with minimal parameter tuning and limiting assumptions

### Nathan Lee^1^, Kameron Decker Harris^2^, Aleksandr Aravkin^1^

#### ^1^University of Washington, Department of Applied Mathematics, Seattle, WA, United States; ^2^University of Washington, Department of Computer Science, Seattle, WA, United States

##### **Correspondence**: Nathan Lee (nlee41@uw.edu)

*BMC Neuroscience* 2018, **19(Suppl 2):**P6

Spike train recovery from fluorescent calcium imaging presents multiple challenges, including contamination of the signals, parameter tuning, and nonlinear dynamics occurring on different time scales. We present an unsupervised algorithm for spike deconvolution with an emphasis on minimizing parameter tuning and pre-estimation. Our optimization problem (Fig. 1A) minimizes the number of spike times, also known as the zero-norm. This results in a hard-thresholding that helps to debias autoregressive (AR) coefficients from the constraining of the residual errors of the fluorescent signal. Three constraints are enforced to retain biological relevance: (1) spikes must be nonnegative, (2) residual error between calcium levels and measured fluorescence must be less than a noise level which can be estimated directly from the data, and (3) the calcium signaling follows an order *p* AR model [1]. Rather than fix the AR coefficients to obtain a convex program, we optimize them, the calcium levels, and neural activity simultaneously, yielding a nonconvex problem. In each step of the algorithm, first we project out AR coefficients from the nonconvex portion of the objective to obtain a closed form update. Then, using these updated AR coefficients, a step is taken in the direction of the negative proximal gradient. We benchmark the algorithm on both simulated (Fig. 1B) and real data. Current methods for nonlinear deconvolution require sequential processing, performing separate steps to obtain AR parameters and neural activity. For example, some methods use Markov chain Monte Carlo methods to initialize, and impose strong assumptions, such as that spiking follows a Poisson process. Our approach requires fewer assumptions about the data and its structure, and is robust to initialization. We simultaneously solve for the AR coefficients, calcium levels, and the neural spiking activity. Our ongoing work is focused on inferring a decaying fluorescence or calcium baseline. Rather than assuming a fixed baseline, we propose a smoothly varying model that can account for a decaying baseline, and also incorporate this into the overall inverse problem. This approach can be used to predict a nonlinear decrease in baseline fluorescence intensity that occurs over the course of imaging, such as that due to photobleaching. The dynamic background model can also be used to better resolve calcium dynamics that occur on slower time scales. In summary, we present a spike deconvolution algorithm for calcium imaging that limits parameter tuning, requires minimal estimation of parameters beforehand, and is not sensitive to initial values for accurate recovery of spikes. Further, we require fewer assumptions about the underlying processes behind the neural activity and less information about the structure of the data.

**Fig. 1:**
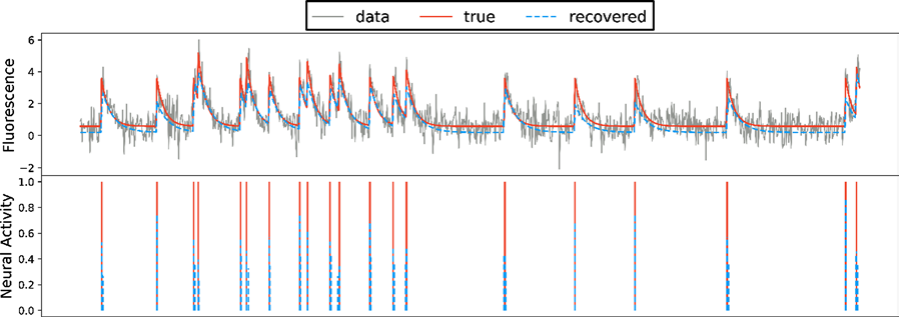
The top panel gives the simulated, true, and computed fluorescence traces. The bottom panel shows the true and computed spikes. We recover all spikes, with few false positives

**Reference**

1. Pnevmatikakis EA, Soundry D, Gao Y, et al.: Simultaneous Denoising, Deconvolution, and Demixing of Calcium Imaging Data. *Neuron* 2016, 89:285–299.

## P7 Applying exact robust PCA to analyze mouse brain activity data

### Roman Levin, Merav Stern, Eric Shea-Brown, Aleksandr Aravkin

#### University of Washington, Department of Applied Mathematics, Seattle, WA, United States

##### **Correspondence**: Roman Levin (rilevin@uw.edu)

*BMC Neuroscience* 2018, **19(Suppl 2):**P7

Principal component analysis (PCA) is a fundamental data decomposition technique which is used to reduce the dimensionality of the data and understand the underlying structure in it. In the presence of an additional structure or features (e.g. sparse outliers), it is beneficial to use a structured decomposition method to analyze the data. A key example is robust PCA (RPCA), which separates the data into low-dimensional and sparse components; a famous use case is the background/foreground separation to separate moving objects from their surroundings.

We apply RPCA to analyze the data of mouse brain activity provided by the Allen Institute for Brain Science. The data is in the form of videos recorded using wide-field imaging of the calcium activity in the cortex dorsal surface. The purpose of our work is to separate the calcium activity generated by the spiking rate from the background activity. As an additional result, we develop a framework which allows to automatically mask the video following the contour of the brain and eliminate the blood vessels, tissue, and background from the data. This reduces noise caused by the blood vessel activity, reflections from the tissue and the skull as well as mouse movements and other limitations of the experimental setup and thus gives an efficient way to preprocess the data to improve the quality of analysis with other methods. We use an *exact* RPCA formulation, which decomposes the data *D* (a certain matricization of the original video downsampled in time) into a low-rank component *LR* (*L* and *R* are low-rank matrices) and a set of sparse residuals *D*-*LR* by solving the matrix factorization problem corresponding to the exact RPCA. Our objective function is the sum of the absolute values of the elements of the residual. The input videos contain an hour of the recordings from the experiment with *100* Hz frequency and *64* × *64* frames; that is, our input data is of the shape *360000* × *64* × *64*. Thus, we obtain a large-scale structured nonsmooth nonconvex optimization problem. Using a novel relaxation approach, we propose an iterative algorithm based on partial minimization to solve this problem. Our algorithm uses only SVD and thresholding steps, and therefore can be readily implemented by analysts who already use PCA to analyze large-scale data. We reconstruct a video from the output of the algorithm to visualize the results. The results, shown in Fig. 1, separate the physical brain structure (contours and vessels) in the center panel of Fig. 1 from a sparse signal distributed throughout the brain (shown using binary thresholding in the right panel of Fig. 1). Our ongoing work seeks to characterize whether and how this signal carries the underlying spiking activity.

**Fig. 1:**
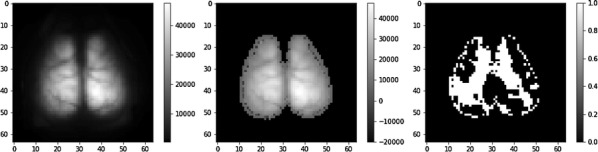
The original snapshots (left) are decomposed into low rank LR (center) and residual D-LR (right). Residuals correspond to brain activity at a particular time moment, shown using a binary image to highlight the structure

## P8 A theory of dendritic buckets

### Hermann Cuntz^1^, Alexander Bird^2^

#### ^1^Frankfurt Institute for Advanced Studies (FIAS) & Ernst Strüngmann Institute (ESI), Computational Neuroanatomy, Frankfurt/Main, Germany; ^2^Frankfurt Institute for Advanced Studies (FIAS), Computational Neuroanatomy, Frankfurt am Main, Germany

##### **Correspondence**: Hermann Cuntz (hermann.neuro@gmail.com)

*BMC Neuroscience* 2018, **19(Suppl 2):**P8

While the electrotonic properties of biophysically realistic neuronal models are most often probed with current injections in the soma or single synaptic inputs this is far from being a natural stimulus for real neurons. In this project we use analytical methods based on cable theory in combination with detailed passive and active compartmental modelling to study the responses of neurons to randomly occurring synaptic inputs in time and dendritic location. We find that under these uniform conditions dendrites behave very similarly to point neurons. The voltage responses throughout the dendritic tree average out to a constant voltage level similar to a bucket that is filled by multiple faucets. Analytically, the voltage integral over the total dendritic length is the same regardless of the location of synaptic inputs. In passive numerical simulations the individual voltage profiles then average out. The local voltage throughout the dendrite would in principle allow decoding of the percentage of synapses active at any given time, which could be very important for synaptic plasticity rules that correlate synaptic activity with the overall activity in the cell. In simple active somatic spiking models voltages are transformed into number of spikes in a manner that further allows decoding of the percentage of active synapses from the current firing rate of a neuron. Overall, while well distributed random synaptic events are also probably not a natural input to the neuron, our calculations serve as a reference point for comparison of the behaviour of neurons in more realistic biophysical neural network models.

## P9 Predictive information as an organization principle for both sensory and cortical circuitry

### Siwei Wang^1^, Idan Segev^1^, Stephanie Palmer^2^, Oren Amsalem^1^, Alexander Borst^3^

#### ^1^Hebrew University of Jerusalem, Department of Neurobiology, Jerusalem, Israel; ^2^University of Chicago, Department of Organismal Biology and Anatomy & Department of Physics, Chicago, IL, United States; ^3^Max Plack Institute, Department of Neurobiology, Munich, Germany

##### **Correspondence**: Siwei Wang (siwei.wang@mail.huji.ac.il)

*BMC Neuroscience* 2018, **19(Suppl 2):**P9

Although efficient prediction is essential to survival, little is known about what mechanisms allow predictions to be instantiated in neural systems. It has recently been shown that optimal encoding of predictive information is at work in the early visual system of vertebrates [5], but it remains open whether and how this theory generalizes to other systems. Specifically, can predictive information serve as a candidate principle to understand neuronal circuit organization, relating structure to function [1]? Here, we show that the encoding of predictive information indeed sheds light on the role of some specific features of neural circuits. We explore how this principle governs circuit organization in both the fly visual system and in mammalian cortex, using biophysically realistic reconstructions of these networks. First, we compute the maximal amount of predictive information guiding evasive maneuvers [2] that the fly motion discrimination system can encode. By comparing this encoded predictive information with the theoretical optimum obtained via an information bottleneck (IB) calculation, we show that the optimal encoding of the predictive information: (1) is present in the fly vertical motion sensitive system (Vertical Sensitive (VS) cells), and (2) is dependent on the presence of strong gap junction connections between VS cells (Fig. 1). Gap junctions (GJ) in this circuit are strikingly strong (3–4 orders of magnitude stronger than those in cortex) and support efficient prediction by passing information between triplets of VS cells without the delays incurred by chemical synapses. More intriguingly, we further show that the presence of these GJs helps the downstream readout scheme by preferentially encoding predictive features that are informative about the long-range future stimulus, thereby enabling successful, brief evasive maneuvers (no more than 40 ms from start to finish). Secondly, we test the idea that efficient prediction is also an organizing principle in cortex. Here, we evaluate how prediction is encoded by exploring how single neurons allocate their firing variability to encode prediction error in different layers of the blue brain column: a dense computer-generated neocortical network of ~ 0.3 mm^3^ composed of ~ 31,000 cells and ~ 36 million synapses [3]. Prediction error, i.e., the expected uncertainty of encoding the future stimulus given the past, was shown to account for up to 50% of neuronal response variability in auditory cortex [4]. With this detailed model circuit, we show that prediction errors can indeed account for firing rate variability of a substantial fraction of pyramidal neurons. We further show that it most strongly correlates with the firing variability of pyramidal neurons in layers 4 and 5, suggesting that signals sent to subcortical areas, such as basal ganglia circuits, may preferentially represent predictive information about the stimulus. This supports the notion that cortical prediction is used to generate reward signals and guide learning. Therefore, our results demonstrate that the efficient coding of predictive information may be a general design principle in a wide variety of neural systems, constraining circuit connectivity in areas downstream of the sensory periphery.

**Fig. 1:**
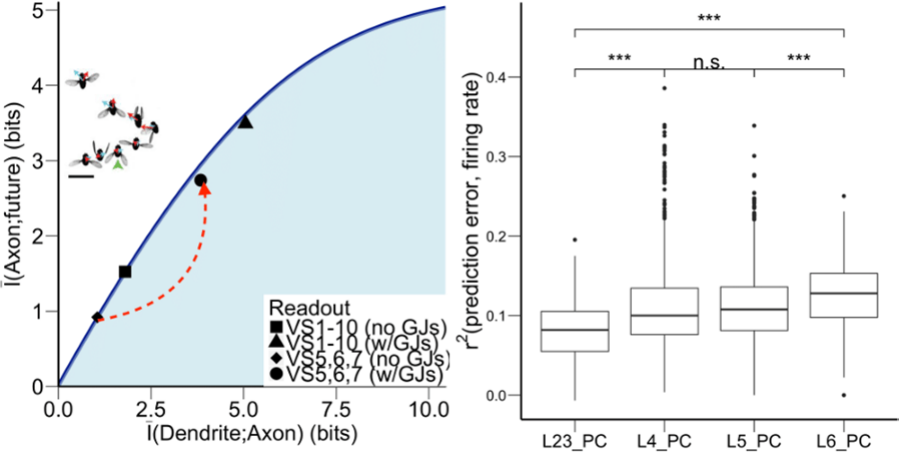
(left) the presence of axonal gap junctions tremendously help the encoding of predictive information, up to 200% compared to when gap junctions are missing. (right) The prediction capability in the cortical circuit (the blue brain column) improves mainly for pyramidal neurons in L4 and L5

**References**

1. Fairhall A, Shea-Brown E, Barreiro A. Information theoretic approaches to understanding circuit function. *Curr Opin Neurobiol*. 2012 Aug;22(4):653–9.
2. Muijres FT, Elzinga MJ, Melis JM, Dickinson MH. Flies evade looming targets by executing rapid visually directed banked turns. *Science* 2014, 11;344(6180):172–7. 10.1126/science.1248955.
3. Markram H, Muller E, Ramaswamy S, et al. Reconstruction and Simulation of Neocortical Microcircuitry. *Cell* 2015 8;163(2):456–92. 10.1016/j.cell.2015.09.029.
4. Rubin J, Ulanovsky N, Nelken I, Tishby N. The Representation of Prediction Error in Auditory Cortex. *PLoS Comput Biol*. 2016 4;12(8):e1005058. 10.1371/journal.pcbi.1005058. eCollection 2016 Aug.
5. Palmer SE, Marre O, Berry MJ, Bialek W. Predictive information in a sensory population. *Proc Natl Acad Sci U S A*. 2015 2;112(22):6908–13. 10.1073/pnas.1506855112. Epub 2015 May 18.

## P10 Distinct roles of anterior cingulate cortex and basolateral amygdala in reinforcement learning under perceptual uncertainty

### Alexandra Stolyarova^1^, Megan Peters^2^, Hakwan Lau^1^, Alicia Izquierdo^1^

#### ^1^University of California, Los Angeles, Department of Psychology, Los Angeles, CA, United States; ^2^University of California, Riverside, Bioengineering, Riverside, CA, United States

##### **Correspondence**: Alexandra Stolyarova (astolyarova@psych.ucla.edu)

*BMC Neuroscience* 2018, **19(Suppl 2):**P10

The incremental trial-by-trial refinement of behavior can be captured by reinforcement learning [1] models which map stimuli to actions using reward prediction errors (RPEs). Most tasks assessing the neural underpinnings of RL have used clearly discriminable and unambiguous cues, leaving open the question of how the brain copes with perceptual uncertainty when learning by trial and error. The subjective sense of certainty, or confidence, that accompanies perceptual decisions can substitute for RPEs in the absence of external feedback [2] and affect neural activity in canonical RL circuits [3]. In the current study, we trained rats to discriminate between horizontally (H)- and vertically (V)-oriented visual stimuli (sinusoidal gratings) either embedded in noise or compounded with orthogonal gratings. Animals indicated their decision based on a stimulus–response rule: H → left and V → right. Following discrimination, ratsexpressed their confidence by time wagering [4]: they could wait a self-timed delay in anticipation of reward or initiate a new trial. In general, rats’ expressed confidence increased with accuracy and was higher for correct than error choices. Yet confidence computations overly relied on perceptual information congruent with the decision (i.e., rats waited longer when the contrast of the grating favoring the choice increased, even in the absence of performance increases), while decisions themselves weighed congruent and incongruent evidence equally, consistent with previous studies in primates [5.6]. This allowed us to identify two stimulus conditions for each animal that produced matched decision accuracy and reinforcement history, but different subjective confidence levels. Rats were then randomly assigned to a low- (LC) or high-confidence (HC) group and performed a reversal learning task, which required remapping of the stimulus–response contingency for the LC or HC stimuli, respectively. The key finding is that subjective certainty potentiated learning: reversal learning was faster in the HC group. Motivated by recent work implicating the rat anterior cingulate cortex (ACC) and basolateral amygdala (BLA) in learning under uncertainty [7], we chemogenetically silenced projection neurons in these regions. Inhibition of the ACC decreased metacognitive sensitivity (i.e., the trial-by-trial correspondence between accuracy and confidence; [8], rendering confidence reports invariant to the strength of the evidence and thereby attenuating the benefit of certainty on learning. In contrast, BLA silencing slowed reversal learning, but left confidence reports intact. Finally, we extended the standard RL model to allow confidence to directly influence value updating. Fitting this model to rat behavior revealed that only BLA inhibition decreased the learning rate. Conversely, ACC inhibition attenuated the impact of confidence on value computations and decreased the inverse temperature parameter in the decision rule that maps action values to choice probabilities, indicating a decreased reliance on the learned information. Thus, the ACC may aid in estimating the reliability of perceptual and value information to guide action selection, whereas the BLA appears to play a more general role in potentiating learning when environmental conditions significantly change.

**References**

1. Sutton RS, Barto AG. *Reinforcement learning: An introduction* (*Vol*. *1*). MIT press Cambridge, 1998.
2. Guggenmos M, Wilbertz G, Hebart MN, Sterzer P. Mesolimbic confidence signals guide perceptual learning in the absence of external feedback. *Elife* 2016, 5, 10.7554/elife.13388.
3. Hebart MN, Schriever Y, Donner TH, Haynes JD. The Relationship between Perceptual Decision Variables and Confidence in the Human Brain. *Cereb Cortex* 2016, 26, 118–130, 10.1093/cercor/bhu181.
4. Lak A, Stauffer WR, Schultz W. Dopamine prediction error responses integrate subjective value from different reward dimensions. *Proc Natl Acad Sci U S A* 2014, 111, 2343–2348, 10.1073/pnas.1321596111
5. Zylberberg A, Barttfeld P, Sigman M. The construction of confidence in a perceptual decision. *Front Integr Neurosci* 2012, 6, 79, 10.3389/fnint.2012.00079.
6. Maniscalco B, Peters MA, Lau H. Heuristic use of perceptual evidence leads to dissociation between performance and metacognitive sensitivity. *Atten Percept Psychophys* 2016, 78, 923–937, 10.3758/s13414-016-1059-x
7. Winstanley CA, Floresco S. B. Deciphering Decision Making: Variation in Animal Models of Effort- and Uncertainty-Based Choice Reveals Distinct Neural Circuitries Underlying Core Cognitive Processes. *J Neurosci* 2016, 36, 12069–12079, 10.1523/jneurosci.1713-16.2016.
8. Maniscalco B, Lau H. A signal detection theoretic approach for estimating metacognitive sensitivity from confidence ratings. *Conscious Cogn*, 2012, 21(1), 422–430. 10.1016/j.concog.2011.09.021

## P11 Efficient search with Lévy flights emerges from stochastic optimization

### Lukasz Kusmierz, Taro Toyoizumi, Alireza Gourdarzi

#### RIKEN Brain Science Institute, Neural Computation and Adaptation, Wako, Japan

##### **Correspondence**: Lukasz Kusmierz (nalewkoz@gmail.com)

*BMC Neuroscience* 2018, **19(Suppl 2):**P11

A growing body of evidence shows that many organisms commonly exhibit Lévy flights (LFs) during their search behavior. For example, trajectories of T cells [1], fruit flies [2], wandering albatrosses [4], human saccades [3], and free word association involve power- law distributions of displacement steps, summarizing frequent nearby explorations and infrequent jumps to distant locations. Although there are multiple putative explanations as to why LFs might emerge from case specific search constraints, a general theory explaining this behavior is lacking. We show that Newton’s optimization method with noisy measurements generically leads to heavy tails of the step-size distribution. The resulting stochastic process is a LF with the tail index α = 1. Additionally, the magnitude of large jumps in our model strongly depends on the local curvature of the optimized function, with rarer jumps close to targets. This suggests that noisy Newton’s optimization method may be an efficient way of combining global random exploration with lo- cally optimal exploitation. We thus examine the circumstances under which the heavy-tailed steps can be advantageous for the search. Since search patterns of many organisms resemble those of LFs, our results suggest that they may be employing second order derivatives. We further discuss implications of our results for models of learning. Plasticity rules are often derived assuming the steepest descent method. We argue that even approximate and very noisy second order optimization should be more efficient.

**References**

1. Harris DT, Kranz DM. Adoptive T Cell Therapies: A Comparison of T Cell Receptors and Chimeric Antigen Receptors. *Trends Pharmacol*. *Sci*. 2016, 37(3), 220–230.
2. Reynolds AM, Frye MA. Free-Flight Odor Tracking in Drosophila Is Consistent with an Optimal Intermittent Scale-Free Search. *PLOS One* 2007
3. Brockmann D, Geisel T. The ecology of gaze shifts. *Neurocomputing* 2000, 32–33.
4. Viswanathan M, Childers TL. Processing of Numerical and Verbal Product Information. *Journal of Consumer Psychology* 1996

## P12 A multi-scale data-based network model of lateral inhibition in mouse olfactory bulb

### Daniel Zavitz^1^, Isaac Youngstrom^2^, Matt Wachowiak^2^, Alla Borisyuk^1^

#### ^1^University of Utah, Department of Mathematics, Salt Lake City, UT, United States; ^2^University of Utah, Department of Neurobiology & Anatomy, Salt Lake City, UT, United States

##### **Correspondence**: Daniel Zavitz (zavitz@math.utah.edu)

*BMC Neuroscience* 2018, **19(Suppl 2):**P12

In this study, we investigate a multiscale model of inter-glomerular connectivity of the mouse olfactory bulb. Each node in the network represents a glomerulus comprised of many neurons. We specify probabilistic wiring rules for outgoing connections of individual cells, based on tracing data, and study the emergent properties of the resulting network of nodes. An important parameter in the wiring rules, unknown from experiments, is connection selectivity. It is determined by the size of each node’s “target set”—the set of nodes where all outgoing connections must land. We investigate graph theoretic properties of these networks such as weighted degree distributions, clustering coefficients, centrality etc. We find that these properties differ significantly from well-studied network models (random, small-world, scale-free, etc.). Finally, we add minimal but biologically realistic nonlinear firing rate dynamics to the networks to study the effect of network structure on the processing of sensory data. Using both experimentally-derived and artificial stimuli, we find that in these networks, regardless of connection selectivity, lateral inhibition mediates the sparsening of neural code and the decorrelation of representations of similar stimuli.

## P13 Assessing phase-locking and entrainment in oscillatory networks using one-dimensional maps

### Casey Diekman, Amitabha Bose

#### New Jersey Institute of Technology, Department of Mathematical Sciences, Newark, NJ, United States

##### **Correspondence**: Amitabha Bose (bose@njit.edu)

*BMC Neuroscience* 2018, **19(Suppl 2):**P13

A central feature of many oscillatory networks is their ability to display phase-locked solutions where the constituent elements fall into a well defined pattern in which the phase difference between pairs of oscillators can be determined. The period of the locked network often depends on a combination of intrinsic and synaptic properties. Many such networks contain an identifiable pacemaker. In these cases, the phase-locking of the other network elements is often referred to as entrainment because the period of the pacemaker determines the overall network period. In this poster, we consider entrainment that arises in circadian systems. Such networks are subject to an external, pacemaking 24 h light–dark drive in which the intensity and total hours of light within the 24 h cycle are important parameters. The large amplitude, long-lasting light input necessitates a different approach to considering phase locking than in networks that primarily are connected throughout weaker, shorter-lasting synaptic inputs. We recently developed a new computational tool, a 1-dim entrainment map, to assess whether and at what phase a circadian oscillator entrains to periodic light–dark (LD) forcing. We have applied the map to a variety of circadian oscillators ranging from the Novak-Tyson model for protein-mRNA interactions to the Kronauer model of the human circadian rhythm. The map*F* (*x*)is defined by choosing a Poincare section in the phase space of the oscillator through which a trajectory is known to pass. We choose a photoperiod that dictates how many hours of light to subject the oscillator to within a 24-hour cycle. Beginning with a trajectory lying on the Poincare section and letting*x*denote the number of hours of light that have occurred since the lights last turned on, we evolve a trajectory from the Poincare section until it returns back to the section. The map*F* (*x*)measures the new number of hours of light that have occurred since the most recent onset of the lights. We show that the 1-dim map*F* (*x*)has several properties including that it is piecewise increasing, has at most one discontinuity and is periodic, *F* (*0*)= *F* (*24*). A stable fixed fixed point of the map corresponds to a stable LD-entrained periodic solution of the circadian oscillator to the 24 h period. Using the entrainment map, we systematically investigate how various intrinsic properties of the circadian oscillator interact with properties of the LD forcing to produce stable circadian rhythms. For example, we show that when the endogenous period of an oscillator is too fast or two slow, the map undergoes a saddle-node bifurcation destroying the stable fixed point and entrainment is lost. In this poster, we focus on how to use the map to study the reentrainment process due to jet lag after long-distance travel. We show that the east–west asymmetry of jet lag depends on the direction and length of travel, the photoperiod and an individual’s endogenous clock; see Fig. 1. Further, we show that individuals can experience jet lag after purely north–south travel due to changes in the photoperiod between departure and arrival cities. A goal of our work is to use the entrainment map on models of the suprachiasmatic nucleus in relation to circadian regulation of sleep–wake cycling. The mathematical and computational methods used to study these problems should be of wide interest to members of the CNS community.

**Fig. 1:**
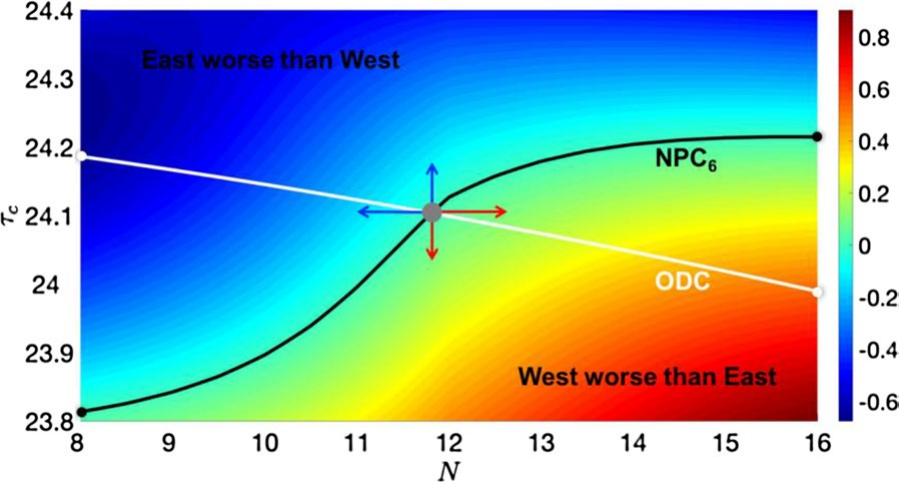
The difference in reentrainment times in travel 6 time zones west versus east as a function of photoperiod (N) and endogenous period (tau_c) computed using 1-dim maps. Reentrainment along the ODC curve is orthodromic. The NPC6 curve indicates parameter pairs for which there is no asymmetry in reentrainment times

## P14 Functional role of 5-HT1A receptors in serotonergic modulation of active exhalation

### William Barnett^1^, Yaroslav Molkov^1^, Lucas Koolen^2^, Adrian Newman-Tancredi^3^, Mark Varney^3^, Ana Abdala^2^

#### ^1^Georgia State University, Department of Mathematics & Statistics, Atlanta, GA, United States; ^2^University of Bristol, School of Physiology, Pharmacology & Neuroscience, Biomedical Sciences Faculty, Bristol, United Kingdom; ^3^Neurolixis Inc, Dana Point, CA, United States

##### **Correspondence**: William Barnett (wbarnett2@gsu.edu)

*BMC Neuroscience* 2018, **19(Suppl 2):**P14

In healthy humans, lung ventilation is tightly controlled to maintain physiological levels of CO2. During restful breathing, exhalation is largely passive; the lungs deflate as the diaphragm relaxes. In exercise, hypoxia or hypercapnia, active exhalation is engaged to increase lung ventilation; the abdominal and thoracic muscles contract during the final half of exhalation. This activity is quantifiable in vivo via the abdominal nerve (AbN). Active exhalation is thought to originate from late-expiratory (late-E) neurons located within the parafacial respiratory group (pFRG). However, the mechanisms by which this expiratory oscillator is recruited and interacts with the respiratory central pattern generator (CPG) are not fully understood. It has been proposed that active exhalation emerges during hypercapnia when late-E neurons receive excitatory drive from putative central chemoreceptors in the retrotrapezoid nucleus (RTN), overcoming inhibition from the respiratory CPG. The Kölliker-Fuse (KF) is thought to modulate the strength of inhibitory inputs from the respiratory CPG to late-E neurons. Both RTN and KF receive inputs from 5-HT neurons located in the medullary raphe, some of which are chemosensitive. Systemic administration of 5-HT1Areceptor (5-HT1AR) antagonist promoted irregular breathing and apneas in rodents. This effect was recapitulated by focal application of antagonist into the KF. Conversely, systemic administration of 5-HT1AR agonists ameliorated breathing irregularity and apneas in C57BL/6 and *Mecp2* deficient mice, and focal administration into the KF corrected apneas in the latter. Since deficits of inhibitory input to the KF were shown to contribute to apneas in *Mecp2* deficient mice, we propose that 5-HT1AR agonists inhibit KF and CPG neuron sub-populations that provide inhibitory drive to late-E neurons, disinhibiting the latter. Here, we combined experimental approach with computational simulations of the respiratory CPG to test hypotheses that 5-HT1AR activation promotes active exhalation in the absence of lung inflation feedback. For this, we determined the effects a biased, highly selective, and efficacious 5-HT1AR agonist, NLX-101 (aka F15599), on resting respiratory motor outputs of decerebrate rats under cardio-pulmonary bypass. NLX-101 increased respiratory rate in a concentration-dependent fashion, differing significantly from baseline at ≥ 0.1 μM. Notably, at [NLX-101] ≥ 0.1 μM, late-E bursts emerged in the AbN under eucapnia. Simulations of 5-HT1AR agonist-induced active exhalation, that best fitted the data, required the following testable assumptions: (1) 5-HT1AR activation inhibited KF subpopulations that drive post-inspiratory neurons in the Bötzinger complex (BC); (2) 5-HT1AR directly inhibited post-inspiratory neurons in the BC; leading to (3) disinhibition of late-E neurons and emergence of active exhalation. In summary, 5-HT1AR agonism evokes active exhalation and increases respiratory frequency in a manner resembling the hypercapnic response. The data indicates that 5-HT1AR may contribute to the emergence of active exhalation in response to hypercapnia. Our modeling results suggest that this respiratory response may be mediated by suppression of the KF and post-inspiratory neurons in the BC, disinhibiting late-E neurons. Future experimental verification of these predictions would provide a mechanistic basis for the indication of 5-HT1AR agonists to treat respiratory depression.

## P15 Analyzing how Na+/K+ pump influences the robust bursting activity of half-center oscillator (HCO) models

### Ronald Calabrese, Anca Doloc-Mihu

#### Emory University, Department of Biology, Atlanta, GA, United States

##### **Correspondence**: Anca Doloc-Mihu (adolocm@gmail.com)

*BMC Neuroscience* 2018, **19(Suppl 2):**P15

Robustness in bursting activity type of central pattern generating networks (CPGs) is achieved by the coordinated regulation of many membrane and synaptic current parameters. CPG neurons depend upon a Na+/K+ pump to maintain the ionic gradients that establish the resting potential and thus support other ionic currents. The Na+/K+ pump produces an outward net current proportional to its activity. However, how the Na+/K+ pump and its current are directly involved in the mechanisms that allow multiple parameters to interact, thus producing and maintaining rhythmic single cell and network activity, is not yet fully understood.

We use a half-center oscillator (HCO) mathematical model that includes a Na+/K+ pump to replicate the rhythmic alternating bursting of mutually inhibitory interneurons of the leech heartbeat CPG under a variety of experimental conditions. This HCO model consists of a pair of reciprocally inhibitory model neurons, each represented as a single isopotential electrical compartment with Hodgkin and Huxley type intrinsic membrane and synaptic conductances. The model has eight currents with voltage-dependent conductances (including two types of inhibitory synaptic currents, spike mediated and graded) and a Na+/K+ pump current, which tracks changes in intracellular Na+ concentrations that occur as a result of the Na+ fluxes carried by ionic currents. The Na+/K+ pump exchanges two K+ ions for three Na+ ions. Its current has a sigmoidal dependence on intracellular Na+ concentrations. Na+ currents include the fast spiking current (INa) and a persistent Na+ current (IP). Both the hyperpolarization-activated cation (Ih) and leak currents have Na+ and K+ components. We build a large parametric space of this HCO model and its corresponding isolated neuron models by varying a set of 9 key parameters (the maximal conductances of the persistent Na+ (IP), slow Ca2+, leak, hyperpolarization-activated (Ih), and persistent K+ currents, across of 50, 75, 100, 125, and 150 percent of their canonical values [3], the leak reversal potential across − 66.25, − 62.5, − 58.75, − 55, and − 51.25 mV, the half-activation of the Na+/K+ pump across − 2, − 1, 0, 1, and 2 mV, the maximum Na+/K+ pump current across 0.38, 0.41, 0.44, 0.47, and 0.5 nA, and the slope coefficient across 90, 95, 100, 105, and 110 percent of its canonical value) in all combinations possible (a brute-force approach). Then, we systematically explored this parameter space and analyzed its 1.65 million of simulated instances each having canonical synaptic interactions. For each simulated HCO model we computed its bursting characteristics, which we recorded into a row of a SQL database table called PumpHCO-db (similar to our previous work). This study reports on the results of our ongoing investigation on how realistic activity of HCOs is affected by the Na+/K+ pump. We use this PumpHCO-db database and follow our methodology described in previous work to analyze how the Na+/K+ pump influences the robust realistic bursting activity of HCO models. We are particularly interested in parameter variations corresponding to known neuromodulations such as the modulation of Ih and maximal Na+/K+ pump current by myomodulin. Our study here is preliminary to a full investigation of the role of the Na+/K+ pump in the robust maintenance of functional bursting activity.

## P16 Experimental directory structure (Exdir): An alternative to HDF5 without introducing a new file format

### Svenn-Arne Dragly^1^, Milad Hobbi Mobarhan^2^, Mikkel Lepperød^2^, Simen Tennøe^3^, Gaute Einevoll^4^, Marianne Fyhn^2^, Torkel Hafting^5^, Anders Malthe-Sørensen^1^

#### ^1^University of Oslo, Department of Physics, Oslo, Norway; ^2^University of Oslo, Department of Biosciences, Oslo, Norway; ^3^University of Oslo, Department of Informatics, Oslo, Norway; ^4^Norwegian University of Life Sciences, Faculty of Science and Technology, Aas, Norway; ^5^University of Oslo, Institute of Basic Medical Sciences, Oslo, Norway

##### **Correspondence**: Gaute Einevoll (gaute.einevoll@nmbu.no)

*BMC Neuroscience* 2018, **19(Suppl 2):**P16

There is an increased focus in the scientific community on data sharing and reproducible research. Open formats with publicly available specifications facilitate data sharing and reproducible research. Hierarchical Data Format 5 (HDF5) is a popular open format widely used in neuroscience, often as a foundation for other, more specialized formats. However, certain drawbacks related to HDF5’s complex specification have initiated a discussion for an improved replacement. Here, we propose a novel alternative, the Experimental Directory Structure (Exdir), an open specification for data storage in experimental pipelines which aims to improve drawbacks associated with HDF5 while retaining its advantages. Exdir is not a file format in itself, but a specification for organizing files in a directory structure. Within the Exdir structure, data is stored using established open source data formats. While HDF5 stores data and metadata in an internal hierarchy in a single binary file, Exdir uses file system folders to represent the hierarchy, where metadata is stored in human-readable YAML files, and data is stored in the NumPy binary format. The idea of such a solution is already present in the scientific community, but no formal standard has been introduced, making it unnecessarily hard to share data and develop common tools. Exdir facilitates improved data storage, data sharing, reproducible research, and novel insight from interdisciplinary collaboration. We invite the scientific community to join the development of Exdir to create an open specification that will serve as a foundation for open access to and exchange of data.

## P17 A mathematical framework for modeling large scale extracellular electrodiffusion surrounding morphologically detailed neurons

### Gaute Einevoll^1^, Geir Halnes^1^, Andreas Solbrå^2^, Aslak Wigdahl Bergersen^3^, Jonas van den Brink^3^, Anders Malthe-Sørensen^2^

#### ^1^Norwegian University of Life Sciences, Faculty of Science and Technology, Aas, Norway; ^2^University of Oslo, Department of Physics, Oslo, Norway; ^3^Simula Research Laboratory, Fornebu, Norway

##### **Correspondence**: Geir Halnes (geih@nmbu.no)

*BMC Neuroscience* 2018, **19(Suppl 2):**P17

Many pathological conditions, such as seizures, stroke, and spreading depression, are linked to abnormal extracellular ion concentrations in the brain. Ions move due to both diffusion and electrical migration, and to investigate the role of ion-concentration dynamics under pathological conditions, one must simultaneously keep track of both the ion concentrations and the electric potential in the relevant regions of the brain. This remains challenging experimentally, which makes computational modeling an attractive tool. Previous electrodiffusive models of extracellular ion-concentration dynamics have required extensive computing power, and have therefore been limited to either phenomena on very small spatiotemporal scales (micrometers and milliseconds, see e.g. [1]), or to simplified and idealized 1-dimensional (1-D) transport processes on a larger scale. We have previously introduced the Kirchhoff-Nernst-Planck framework, an efficient framework for modeling electrodiffusion in 1-D [2, 3]. In this study, we introduce a 3-dimensional version of this framework. We use it to model the electrodiffusion of ions surrounding a morphologically detailed pyramidal neuron, with a focus on highlighting the intricate interplay between extracellular ion dynamics and the extracellular potential.

The simulation covered a 1 cubic millimeter cylinder of tissue for over a minute, and was performed in less than a day on a standard desktop computer, demonstrating the framework´s efficiency. We envision that this framework will be useful to elucidate mechanisms behind pathologies, such as for example spreading depression propagation. A preprint of this work is available at bioRxiv [4].

**References**

1. Pods J, Schönke J, Bastian P. Electrodiffusion models of neurons and extracellular space using the Poisson-Nernst-Planck equations–numerical simulation of the intra- and extracellular potential for an axon model. *Biophysical Journal* 2013 105(1), 242–54.
2. Halnes G, Østby I, Pettersen KH, et al. Electrodiffusive Model for Astrocytic and Neuronal Ion Concentration Dynamics. *PLoS Comput*. *Biol* 2013, 9(12):e1003386.
3. Halnes G, Mäki-Marttunen T, Keller D, et al. Effect of Ionic Diffusion on Extracellular Potentials in Neural Tissue. *PLoS Comput*. *Biol* 2016, 12(11):e1005193.
4. Solbrå A, Wigdahl Bergersen A, van den Brink J, et al. Amplification and Suppression of Distinct Brain-wide Activity Patterns by Catecholamines. *bioRxiv* 2018, 261107.

## P19 Modeling the percieved perils of sodium channel anticonvulsants in Dravet syndrome

### Andrew Knox

#### University of Wisconsin, Department of Neurology, Madison, WI, United States

##### **Correspondence**: Andrew Knox (atknox@gmail.com)

*BMC Neuroscience* 2018, **19(Suppl 2):**P19

**Objective:** NaV1.1 sodium channel mutations are a well-known cause of epilepsy syndromes, some severe (such as Dravet syndrome) and some more benign (genetic epilepsy with febrile seizures plus). The conventional wisdom is that the many anticonvulsant medications that act on sodium channels should be avoided, although this is only supported in the medical literature by a few case reports or retrospective reviews. In this study, we use a computational model to predict the effects of carbamazepine in patients Dravet syndrome secondary to truncation mutations.

**Methods:** A thalamocortical model described by Destexhe was modified to incorporate sodium channels with slow and fast inactivation. Truncation mutation was then simulated by reducing interneuron sodium channel conductance by 50%. Effects of carbamazepine and oxcarbazepine were then simulated by increasing the fast inactivation time of sodium channels in cortical neurons, while effects of eslicarbazepine and lamotrigine were simulated by increasing slow inactivation time.

**Results:** Introduction of truncation mutation into the model reduced the amplitude of sodium currents from interneurons, decreasing the number of action potentials from this population of neurons and leading to periods of prolonged bursting from pyramidal neurons akin to tonic seizures. Simulation of carbamazepine and oxcarbazepine reduced spiking rates in both populations, decreasing incidence of seizures. Simulation of eslicarbazepine and lamotrigine also decreased action potentials in both populations but did not prevent seizures.

**Discussion:** This study provides mechanistic evidence that sodium channel anticonvulsants can be beneficial in Dravet syndrome, although effects may be difficult to predict. This model could be validated with patients who have known sodium channel electrophysiology and clinical data documenting efficacy of sodium channel drugs. If validated, the model then could be used to predict the potential benefit of sodium channel anticonvulsants in a given patient with a known sodium channel mutation. This represents a prime application for computer modeling to aid in personalized medicine for patients with epilepsy.

## P20 Spatial modeling of AMPA receptor trafficking and sorting at the Endosome

### Erik De Schutter, Sarah Nagasawa, Iain Hepburn, Andrew R. Gallimore

#### Okinawa Institute of Science and Technology, Computational Neuroscience Unit, Onna-Son, Japan

##### **Correspondence**: Erik De Schutter (erik@oist.jp)

*BMC Neuroscience* 2018, **19(Suppl 2):**P20

AMPA receptors (AMPAR) are constitutively trafficked from the neuronal plasma membrane to the endosome, where they are sorted either to degradation at the lysosome or returned to the membrane via recycling vesicles. AMPAR trafficking is controlled by a family of proteins known as the Rab GTPases, which coordinate the sorting of AMPAR-containing vesicles through the endosomal system. The network of Rab proteins can be manipulated in response to synaptic activity, or the induction of plasticity, to increase or decrease trafficking rates, or to redirect the movement of AMPARs towards either degradation or recycling1. For example, studies in cerebellar and hippocampal cells have revealed the critical importance of Rab7 activation in the regulation of long term depression, by augmenting the Rab7-dependent degradation pathway2. Although many molecular models of AMPAR trafficking in synaptic plasticity have been developed, these have almost exclusively considered trafficking only at the plasma membrane, with the crucial subcellular trafficking pathways being neglected3. This is largely because the modeling tools for detailed spatial simulation of vesicular and endosomal trafficking have not been available. Although spatial modeling has advanced in recent years, with voxel-based molecular simulators such as STEPS (steps.sourceforge.net) incorporating spatial effects—diffusion and probabilistic interactions between molecules within realistic neuronal mesh structures 4—there has been no explicit account of molecule size or excluded volume effects. This approach has the advantage of computational performance and accuracy for small molecules and ions, but these simplifying assumptions break down for complex structures of large size such as vesicles and a new modeling approach is required. We have developed Vesicle objects within STEPS as spherical structures of user-defined size, which occupy a unique excluded volume and sweep a path through the tetrahedral mesh as they diffuse throughout the cytosol. Hybrid modeling allows us to retain normal reaction–diffusion mechanics in the system for other biochemical species, such as kinases, receptors, and calcium. The incorporation of phenomena such as endocytosis, exocytosis, and the fusion and budding of vesicles to and from intracellular membranes allow us to simulate the complete AMPAR vesicular cycle. Our preliminary models using this vesicle modeling technology have been successful in replicating recent experimental studies revealing the essential role of specific Rab proteins in the expression of long term depression at the parallel fiber-Purkinje cell synapse. It is expected that this new methodology will enable us to model synaptic plasticity and other subcellular processes at levels of detail that have, until now, remained beyond the reach of modeling technologies. We envisage that this will open up entirely new avenues of modeling research in all areas of neuroscience and cell biology in which the regulation of protein trafficking plays a role.

**References**

1. Fernandez-Monreal M, Brown TC, Royo M, Esteban JA. The Balance between Receptor Recycling and Trafficking toward Lysosomes Determines Synaptic Strength during Long-Term Depression.*J*. *of Neurosci*. 2012, 32, 13200–13205.
2. Kim T, Yamamoto Y, Tanaka-Yamamoto K. Timely regulated sorting from early to late endosomes is required to maintain cerebellar long-term depression. *Nat*. *Comm*.2017, 8, 16.
3. Gallimore AR, Kim T, Tanaka-Yamamoto K, De Schutter E. Switching On Depression and Potentiation in the Cerebellum. *Cell Rep* 2018,.22, 722–733.
4. Hepburn I, Chen W, Wils S, De Schutter E. STEPS: efficient simulation of stochastic reaction–diffusion models in realistic morphologies.*BMC Syst*. *Biol*. 2012, 6, 36.

## P21 Neural representation of perceptual texture dimensions in macaque area V4

### Taekjun Kim, Wyeth Bair, Anitha Pasupathy

#### University of Washington, Department of Biological Structure, Seattle, WA, United States

##### **Correspondence**: Taekjun Kim (taekjunkim1223@gmail.com)

*BMC Neuroscience* 2018, **19(Suppl 2):**P21

Visual texture-the structure of a surface which underlies the perception of roughness or smoothness, fineness or coarseness-is thought to be processed along the ventral visual pathway in the primate. In most past studies, texture was typically defined as a spatially homogeneous pattern composed of separated elements such as lines or forms. The neural correlates of texture perception were tested by comparing responses to arrays of oriented line segments with and without the presence of differently oriented line segments in the surround. A simple, low-level mechanism (e.g., orientation-tuned suppression) might be sufficient to explain this discrimination. More recently, researchers have probed the neural representation of more naturalistic texture images. These studies demonstrate that, while V1 responses to texture can be explained on the basis of local orientation and spatial frequency information, responses in V2 and V4 require the inclusion of higher order summary statistics, for e.g. correlations between spatially neighboring filters, correlations between filters with neighboring orientations, etc. However, because of the high dimensionality of these statistics, it is still unclear how these statistics relate to the perceptual quality of texture. Specifically, we still do not know whether there are neurons in the brain that encode the perceptual qualities of smoothness, roughness, fineness, etc. In this study, we focus on four basic texture dimensions, which have been suggested to be crucial for human visual texture perception: Coarseness, Directionality, Regularity, and Contrast. We devised simple statistics to quantify the degrees of the attributes in a given texture image, and then examined whether responses of neurons in macaque area V4 to a variety of natural texture images could be described by selectivity for these perceptually relevant texture features. Our results indicate that many V4 neurons (about 30% of total recorded units) have strong texture selectivity for one or more of the four basic texture features. Textures classified based on neural population activity were in strong agreement with human perception. Interestingly, when we tested neural representation of shape information (e.g., curvature of object boundary) in the same neural population, neurons with strong texture selectivity were rarely overlapped with those having strong shape selectivity (about 40% of total recorded units). These experimental findings suggest that texture and shape encodings are provided by different population of V4 neurons and that texture selective V4 neurons extract key psychophysical measures of texture by computing simple summary statistics.

## P22 Object encoding in macaque inferior temporal cortex under partial occlusion

### Tomoyuki Namima, Anitha Pasupathy

#### University of Washington, Department of Biological Structure, Seattle, WA, United States

##### **Correspondence**: Tomoyuki Namima (namima@uw.edu)

*BMC Neuroscience* 2018, **19(Suppl 2):**P22

Occlusions, which are everywhere in natural scenes, make object recognition a challenging problem. Primate inferior temporal (IT) cortex, the final stage of form processing along the ventral visual pathway, is likely important (Kourtzi and Kanwisher 2001; Lerner et al. 2002; Hegde et al. 2008; Kovacs et al. 1995) but the specific role of IT neurons in representing and recognizing occluded objects is largely unknown. In present study, we examined how IT neurons encode information about occluding and occluded objects and how these signals might subserve shape discrimination. Monkeys were trained to report whether two stimuli presented in sequence were the same or different. The first stimulus in the sequence was unoccluded, while the second was partially occluded with a set of randomly positioned dots of variable diameter. As animals performed this sequential shape discrimination task, we recorded single-unit responses in IT cortex. We found that the responses of IT neurons were predominantly modulated by two factors—the shape of the occluded object and the total area of the occluding dots. Consistent with Kovacs et al. (1995), we found that many IT neurons maintained their shape preference under occlusion. But to our surprise, some neurons responded best to the occluded stimuli while others responded best to the unoccluded stimulus. For some neurons shape selectivity also increased under occlusion. Overall the color of the stimuli and the shape of the occluders played a minimal role in dictating the responses of IT neurons. Our simulation results suggest that IT responses can be modeled on the basis of two signals—one that reflects the shape of the occluded stimulus and a second that reflects the area of the occluding dots. Multiplicative modulation of the signal that reflects the shape of the occluded stimulus by the occluder area followed by an additive modulation by the occluder area can recapitulate the responses and shape selectivity of ITneurons that respond best to occluded and unoccluded stimuli. Thus our results imply that, under the partial occlusion, shape selectivity of some IT neurons seems to be enhanced by taking advantage of signal about occluders and those IT neurons might be involved in stable object perception under the partial occlusion.

## P23 The impact of propagation delay in a Linsker-type network

### Catherine Davey, David Grayden, Anthony Burkitt

#### University of Melbourne, Department of Biomedical Engineering, Melbourne, Australia

##### **Correspondence**: Catherine Davey (cedavey@unimelb.edu.au)

*BMC Neuroscience* 2018, **19(Suppl 2):**P23

Neural plasticity describes the process by which synaptic weights change in response to inputs and is a primary mechanism by which the brain learns. Learning begins prior to birth, with most mammals being born with some functionality in hearing, movement and vision. Linsker’s [1, 2, 3] seminal three-part paper series provided a compelling model of how learning can occur due to spontaneous activity in the absence of environmental input. He showed that structure in synaptic connection densities can evoke temporal correlation in neural activity that, through Hebbian plasticity, induces the emergence of spatial opponent cells in early layers of cortical processing.

While Linsker considered the spatial aspect of synaptic connectivity distributions, the spike propagation delay was assumed to be uniform among all neurons in a lamina, and hence had negligible impact. We address here the question of how spike propagation delay, due to the time taken for an action potential traverses along the axon from a presynaptic neuron to a postsynaptic neuron, affects the resulting pattern of synaptic connectivity. For myelinated axons, propagation delay is primarily a function of distance and axon diameter. Given the importance of motion perception in everyday life, an understanding of the impact of temporal delays in visual processing and its resulting effect upon subsequent neural learning is an important goal that the current work seeks to address. A three-layer, feed-forward network of Poisson neurons with Gaussian synaptic connection densities is used, as in Linsker’s analysis [1]. An expression for covariance between neurons that incorporates both distance-dependent propagation delay and an arbitrary post-synaptic potential (PSP) function is derived. We show that adding temporal delay destroys the structure of the lag-zero covariance and thus inhibits the development of simple cells, which is incongruent with the way in which neural systems are expected to behave. A more plausible simulation would model a presynaptic neuron as impacting a postsynaptic neuron over a finite time. This highlights the importance of the time course of the PSP function. We show the role that the duration of the PSP plays in determining the resulting network structure. We further calculate receptive field size as a function of delay, homeostatic equilibrium, and synaptic connection parameters. The results show the conditions under which the spatial resolution of the developing spatial opponent cells is optimised, and find that these conditions accord with experimental observations.

**Acknowledgements**

This research was supported under the Australian Research Council Discovery Projects funding scheme (project number DP140102947).

**References**

1. Linsker R. From basic network principles to neural architecture: Emergence of spatial-opponent cells. *Proceedings of the National Academy of Sciences of the United States of America* 1986, 83, 7508–7512.
2. Linsker R. From Basic Network Principles to Neural Architecture: Emergence of Orientation-Selective Cells. *Proceedings of the National Academy of Sciences of the United States of America* 1986, 83, 8390–8394.
3. Linsker, R. From Basic Network Principles to Neural Architecture: Emergence of Orientation Columns. *Proceedings of the National Academy of Sciences of the United States of America* 1986, 83, 8779–8783.

## P24 A biologically plausible neural model of visual pathways based on efficient coding

### Yanbo Lian^1^, Hamish Meffin^2^, David Grayden^1^, Tatiana Kameneva^3^, Anthony Burkitt^1^

#### ^1^University of Melbourne, Department of Biomedical Engineering, Melbourne, Australia; ^2^National Vision Research Institute, Carlton, Australia; ^3^University of Melbourne, Electrical and Electronic Engineering, Parkville, Vic, Australia

##### **Correspondence**: Yanbo Lian (yanbol@student.unimelb.edu.au)

*BMC Neuroscience* 2018, **19(Suppl 2):**P24

Sparse coding (or efficient coding) is successful in generating Gabor-like features using natural input images, which suggests that the visual system employs a small number of neurons to represent visual stimuli. Models based on efficient coding have been proposed to account for some physiological phenomena in the primary visual cortex (V1), such as diverse shapes of V1 simple cell receptive fields and visual non-classical receptive field effects (such as end-stopping effects). Though some models based on efficient coding were built from the perspective of biological plausibility, they did not respect some biological constraints, such as Dale’s law (the sign of synaptic connections cannot change through learning) and local learning. In addition, phase-reversed cortico-thalamic feedback, a phenomenon observed in cat cortex, cannot be explained by current biologically plausible models. In this study, we propose a two-layer model of visual pathways from the lateral geniculate nucleus (LGN) to V1 based on efficient coding using rate-based neurons. The first layer has separate channels for on-centered and off-centered LGN cells and the second layer represents V1 simple cells. There are feedforward and feedback connections between two layers and they are initially different. Both feedforward and feedback connections consist of excitatory connections and separate inhibitory connections. The learning rule of updating connections between LGN and V1 is local because it only depends on the pre-synaptic and post-synaptic firing rates. The sign of excitatory or inhibitory connections is not allowed to change during learning. 12-pixel by 12-pixel image patches sampled from ten 512-pixel by 512-pixel pre-whitened natural images are used as the input stimuli to the model. In our simulation, the learning rule is applied after every 100 input patches are displayed to accelerate the learning process. Simulations demonstrate several interesting points. First, our model can explain the emergence of diverse shapes of receptive fields of V1 simple cells: Gabor-like receptive fields and a large percentage of blob-like receptive fields. Second, phase-reversed cortico-thalamic feedback naturally emerges because of the structure of learned connections when natural images are used as input stimuli to train the model. Third, feedforward and feedback connections tend to be identical during learning. Fourth, the overall strength of inhibitory connections between LGN and V1 can significantly alter the connection structure and shape the receptive fields of V1 simple cells. Our model of implement efficient coding incorporates many biological facts such as Dale’s law, non-negative firing rates, local learning rule and the existence of cortico-thalamic feedback. The results suggest that efficient coding can be realised using simple neural circuits and explain important physiological properties of V1.

## P25 Building and simulating a biophysically detailed network model of the mouse primary visual cortex

### Yazan Billeh, Sergey Gratiy, Kael Dai, Ramakrishnan Iyer, Nathan Gouwens, Stefan Mihalas, Christof Koch, Anton Arkhipov

#### Allen Institute for Brain Science, Modelling, Analysis and Theory, Seattle, WA, United States

##### **Correspondence**: Yazan Billeh (yazanb@alleninstitute.org)

*BMC Neuroscience* 2018, **19(Suppl 2):**P25

Rapid advancement in neuroscientific tools has yielded an extraordinary amount of data regarding the structural and dynamical properties of cortical circuits. In parallel, there has been vast progress in parallel computing and software to allow for unprecedented simulation capabilities. Herein we describe our efforts in combing these two exciting advances to develop, in a data-driven manner, a model of the mouse primary visual cortex (area V1) comprising ~ 230,000 neurons from all cortical layers. For developing our cortical model, we used the Brain Modeling ToolKit (BMTK): a python API developed by the Allen Institute (github.com/AllenInstitute/bmtk). BMTK allowed us to construct our network and integrate seamlessly with NEURON [Hines and Carnevale 1997] to allow for parallel simulations. Approximately 51,000 cells are biophysically detailed, pooled from > 100 models of individual neurons from the Allen Cell Types database (celltypes.brain-map.org). The network receives spike-train inputs from filter models representing a variety of functional cell types from the Lateral Geniculate Nucleus (LGN) of the thalamus. The LGN filter models were based on spatiotemporal fits from experimental recordings in vivo [Iyer et. al, in preparation]. The projection architecture from the LGN to the visual cortex neurons was based on experimental literature [Lein & Scanziani 2018]. Purely feedforward simulations showed that the origin of direction selective responses observed in certain cortical cell-types in our model is dependent on the thalamocortical topology. Moreover, experimental measurements were used to fit the excitatory post-synaptic current magnitude that V1 neurons receive in response to grating stimuli.

After optimizing the LGN input to the column, the recurrent connectivity between cell-types and layers was introduced. The probability of connections, strength of connections (unitary PSP), functional connectivity rules, and synaptic placement between all cell-types was obtained via a thorough literature search, resulting in a knowledge graph that combines the connectivity information with the records of literature sources; assumptions were used where data was not available. As the next critical step, the synaptic weights were optimized to produce irregular network activity in response to visual stimulation. We will describe the construction and simulations of the V1 model and discuss how available or hypothesized information about properties of cell types, feedforward connectivity from LGN, and recurrent connectivity has resulted in certain functional properties—such as, for example, orientation and direction selectivity. We will also discuss the plans to utilize the developed model to unravel the role of certain cell-types and connections in generating patterns of neuronal activity and computations in the cortex. The model represents a milestone in the development of data-driven simulations of brain activity and computations in vivo based on extensive characterization of the brain structure in vitro and should provide a valuable resource for the computational neuroscience community, in conjunction with the standardized model construction and simulation interfaces of the Brain Modeling ToolKit.

**References**

1. Hines ML, Carnevale NT. The NEURON simulation environment. *Neural Comput*. 1997, 15, 9(6), 1179–1209.
2. Lien AD, Scanziani M. Cortical direction selectivity emerges at convergence of thalamic synapses. *Nature* 2018, 558, 80–86.

## P26 Nonlinear dynamics tools unfold brain activity in optogenetic experiments

### Jessica Helms^1^, Xandre Clementsmith^1^, Sorinel Oprisan^1^, Tams Tompa^2^, Antonieta Lavin^3^

#### ^1^College of Charleston, Department of Physics and Astronomy, Charleston, SC, United States; ^2^University of Miskolc, Miskolc, Hungary; ^3^Medical University of South Carolina, Charleston, SC, United States

##### **Correspondence**: Sorinel Oprisan (oprisans@cofc.edu)

*BMC Neuroscience* 2018, **19(Suppl 2):**P26

Electrophysiolgy and pharmacology tools have been widely used for exploring and controlling the activity of neural networks. While electrophysiology offers a good temporal resolution and pharmacology allows very narrow targeting of specific cells, they both have a poor spatial resolution. Recently, optogenetic pushed the limits of spatial resolution and accuracy to the level of single cell. Light-induced (optogenetic) control of neuronal activity utilize light-activated photosensitive proteins (microbial opsins), such as channelrhodopsins to switch on/off ionic channels. We carried out a series of optogenetic experiments on male PV-Cre mice infected with a viral vector hChR2(H134R) delivered to the mPFC. Channelrhodopsins hChR2 is adapted for mammalian expression with the H134R mutation that produces a larger and slower photocurrent than wild-type hChR2. In our experiments, the optical stimulation was delivered in vivo by a 473 nm laser and the local filed potential (LFP) was sampled at 10 kHz. We carried out two previous studies, i.e. the control and a cocaine study, whereas here we investigated the effect of D1 receptors antagonist SCH23390 and D2 antagonist sulpiride. Using the delay embedding method, we identified a low-dimensional attractor and unfold its phase space trajectory. The main reason we focus on these two dopamine antagonists is because we want to quantify their ability to bring the neural activity changed by cocaine back to its control (no cocaine) range. As in the previous studies, the mPFC response to a brief 10 ms light pulse was recorded for 2 s. During data post-processing, the first 0.5 s were discarded to remove the transient response of the neural network. We performed a nonlinear time series analysis of LFPs recorded from PV+ neurons in the mPFC using time reversal asymmetry and false nearest neighbor (FNN) statistics between the original signal and surrogate data to identify the nonlinearity in the data set. Delay-embedding method used one-dimensional data (time series) of the membrane potential to unfold the true high-dimensional phase space dynamics. As in the previous study, we used both (1) the autocorrelation and (2) the average mutual information for estimating the lag time. The embedding dimension was determined using the false nearest neighbor method.

## P27 On the subthreshold resonance properties of neurons

### Rodrigo F. O. Pena, Vinícius Cordeiro, Cesar C. Ceballos, Antônio C. Roque

#### University of São Paulo, Department of Physics, Ribeirão Preto, Brazil

##### **Correspondence**: Rodrigo F. O. Pena (rodrigo.pena@usp.br)

*BMC Neuroscience* 2018, **19(Suppl 2):**P27

The subthreshold resonance properties of neurons are usually measured by submitting a neuron to the so-called ZAP function and constructing the impedance amplitude profile as the ratio of Fourier transforms of output and input: Z(f) = FFTout/FFTin [1, 2]. The resonance frequency corresponds to a peak in Z(f). In general, for low amplitude (~ 10 pA) ZAP functions the voltage response oscillations are symmetric about a reference voltage line. However, there is evidence of asymmetric responses to ZAP functions, with non-coincident depolarizing and hyperpolarizing membrane resonance frequencies [3]. Here we study this effect for high amplitude ZAP functions (> 10 pA). We propose two different measures than the usual Z(f). We take the holding membrane potential (Vhold) as reference voltage line (voltages above/below it are positive/negative) and, for each frequency, measure the magnitudes of the maximum and minimum voltages normalized by the ZAP amplitude. These will be called Z + (f) and Z-(f).We studied Z + (f) and Z-(f) for a neuron model [4, 5] submitted to a ZAP function. For low ZAP amplitudes, Z + (f) and Z-(f) are identical but for high ZAP amplitudes, Z + (f) and Z-(f) have different resonance frequencies. We characterized the differences between magnitudes ΔZ = Z + (f +)−Z-(f-) and resonance frequencies Δf = f+− f–in the two-dimensional diagram spanned by Vhold and the time constant of the hyperpolarization-activated currentIh. There are regions in the diagram where the neuron can discriminate the frequency change of the input current based on its voltage response profile. This suggests that a neuron can be sensitive to changes in the frequency of its synaptic inputs, and this sensitivity depends on intrinsic parameters of its ionic currents. Our theoretical results reproduce a phenomenon which has been observed experimentally [3] suggesting that the quantities Z + (f) and Z-(f) as defined here can be useful in further studies of resonance phenomena in neurons.

**Acknowledgements**

This work was produced as part of the activities of FAPESP Research, Disseminations and Innovation Center for Neuromathematics Grant 2013/07699-0. VLC and RFOP are recipients of the respective FAPESP scholarships: 2017/05874-0 and 2013/25667-8. CCC is supported by a CAPES PhD scholarship. ACR is partially supported by the CNPq fellowship Grant 306251/2014-0. RFOP and ACR are also part of the IRTG 1740/TRP 2015/50122-0, funded by DFG/FAPESP.

**References**

1. Hutcheon B, Yarom Y. Resonance, oscillation and the intrinsic frequency preferences of neurons. *Trends Neurosci*. 2000, 5 216–222.
2. Rotstein HG, Farzan N. Frequency preference in two-dimensional neural models: a linear analysis of the interaction between resonant and amplifying currents. *J Comput Neurosci*. 2014, 37, 9–28.
3. Fischer L, Leibold C, Felmy F. Resonance properties in auditory brainstem neurons. *Front Cell Neurosci*. 2018, 12, 8.
4. Pena RFO, Ceballos CC, Lima V, Roque AC. Interplay of activation kinetics and the derivative conductance determines the resonance properties of neurons. *arXiv preprint* 2017, arXiv:1712.00306.
5. Pospischil M, Toledo-Rodriguez M, Monier C, et al. Minimal Hodgkin–Huxley type models for different classes of cortical and thalamic neurons. *Biol Cybern*. 2008, 99, 427–441.

## P28 Implementation of the Potjans-Diesmann cortical microcircuit model in NetPyNE/NEURON with rescaling option

### Cecilia Romaro^1^, Fernando Najman^2^, Salvador Dura-Bernal^3^, Antônio C. Roque^1^

#### ^1^University of São Paulo, Department of Physics, Ribeirão Preto, Brazil; ^2^University of São Paulo, Math and Statistics Department, São Paulo, Brazil; ^3^SUNY Downstate Medical Center, Department of Physiology and Pharmacology, Brooklyn, NY, United States

##### **Correspondence**: Cecilia Romaro (cecilia.romaro@usp.br)

*BMC Neuroscience* 2018, **19(Suppl 2):**P28

The Potjans-Diesmann (PD) model [1] reproduces the cortical network under a 1 mm^2^ surface area of early sensory cortex in 1x1 scale. The network consists of around 80,000 leaky integrate-and-fire (LIF) neurons divided in eight cell populations representing excitatory and inhibitory neurons in cortical layers 2/3, 4, 5 and 6. External input is provided by thalamic and cortico-cortical afferents. The model generates spontaneous activity with layer-specific average firing rates and synchrony and irregularity features similar to the ones observed experimentally, and allows a study of the propagation of thalamic inputs from layers 4 and 6 through all layers. The network, originally built in NEST [2], specifies fixed numbers of excitatory and inhibitory neurons per layer, the number of connections between these neuronal populations and the number of external inputs to each cell population. These numbers are based on experimental data. In this work, we converted the PD model with rescaling option from NEST to NetPyNE (www.netpyne.org) [3], a high-level interface to the NEURON simulator [4] that facilitates the development, parallel simulation and analysis of biological neuronal networks. The rescaling option for the PD model, not addressed in the original article, but included in the source code available at the Open Source Brain (OSB) platform [5], which generates layer-specific average firing rates within the margins of error determined in the original article. The rescaling implemented in the NetPyNE version depends on a single parameter in the interval [0, 1], which is used to resize the numbers of network neurons, connections and external inputs as well as the synaptic weights while keeping the matrix of connection probabilities and the proportions of cells per population fixed. The NetPyNE implementation, which employs parallel NEURON as its backend simulator, opens the possibility of constructing network models with the PD model connection topology but using compartmental conductance-based neuron models instead of LIF neurons. This allows a new array of possible studies, such as investigating the interaction between network topology and dendritic morphology or channel-specific parameters. Additionally, NetPyNE employs a high-level declarative format that clearly separates the model parameters from the underlying implementation, making the PD model easier to understand and manipulate. NetPyNE enables efficient parallel simulation of the model with a single function call and provides a wide array of built-in analysis functions to further explore the model.

**Acknowledgements**

This work was produced as part of the activities of FAPESP Research, Disseminations and Innovation Center for Neuromathematics (Grant 2013/07699-0, S. Paulo Research Foundation).

**References**

1. Potjans TC, Diesmann M. The cell-type specific cortical microcircuit: relating structure and activity in a full-scale spiking network model. *Cerebral Cortex* 2014, 24, 785–806.
2. Gewaltig MO, Diesmann M. NEST:NEural Simulation Tool. *Scholarpedia* 2007, 2, 1430.
3. Lytton WW, Seidenstein H A, Dura-Bernal S, et al. Simulation Neurotechnologies for Advancing Brain Research: Parallelizing Large Networks in NEURON. *Neural Computation* 2016, 28, 10, 2063–2090.
4. Carnevale NT, Hines ML. *The NEURON Book*. 2006, Cambridge, UK: Cambridge University Press.

## P29 Effects of spike frequency adaptation on dynamics of a multi-layered cortical network with heterogeneous neuron types

### Renan O. Shimoura, Nilton Liuji Kamiji, Rodrigo F. O. Pena, Vinícius Cordeiro, Antônio C. Roque

#### University of São Paulo, Department of Physics, Ribeirão Preto, Brazil

##### **Correspondence**: Renan O. Shimoura (renanshimoura@usp.br)

*BMC Neuroscience* 2018, **19(Suppl 2):**P29

The cerebral cortex displays a rich repertoire of internally-generated dynamic states. Different rhythmic activity can be generated by mechanisms at network level (e.g. recurrent excitation-inhibition loops) and at neuronal level (e.g. spike frequency adaptation, SFA). Several processes can influence SFA and one of them is related to application of acetylcholine, which decreases SFA of neocortical neurons [1]. Theoretical studies of cortical activity under SFA effects on population dynamics are based on artificial architectures built from random networks. In spite of the usefulness of these models, it is important to have computational models that try to accurately represent cortical architecture. Recently, Potjans and Diesmann (PD) [2] introduced a multi-layered network model of the cortical microcircuit based on experimental data of mammal neocortex. All neurons of the PD model are described by the same leaky integrate-and-fire (LIF) neuron model. Here we study how the dynamic properties of the model change when the excitatory and the inhibitory neurons are different and described by the adaptive exponential integrate-and-fire (AdEx) model. Neuronal parameters are tuned so that excitatory neurons are of the regular spiking (RS) type and inhibitory neurons are of the fast spiking (FS) type. SFA can be implemented in RS neurons by the change of a single parameter. We will call this the heterogeneous PD model (hPD). Initially, we characterized the spontaneous activity patterns generated in the hPD model by varying the excitation-inhibition balance and the firing rate of the Poissonian background input. Then, we repeated the characterization study for different SFA levels of RS neurons. In general, the hPD model with SFA displayed lower layer-specific average firing rates than the hPD model without SFA. The hPD model with SFA also had mean population spike frequencies closer to experimental data for the awake state. Additionally, we found regions in the parameter space displaying intermittent network oscillations. We observed the emergence of high frequency oscillations in the beta-gamma bands by decreasing SFA, in similar fashion to what has been observed when acetylcholine is released in the visual cortex [3]. In conclusion, the PD model with heterogeneous neuron types provides a good in silico framework to study complex network activity behavior and modulatory effects due to spike frequency adaptation.

**Acknowledgements**

This work is part of the activities of FAPESP Research, Innovation and Dissemination Center for Neuromathematics (Grant 2013/07699-0, S. Paulo Research Foundation).ROS, RFOP and VL are recipients of the respective FAPESP scholarships: 2017/07688-9, 2013/25667-8 and 2017/05874-0. NLK is supported by FAPESP Grant 2016/03855-5. ACR is partially supported by the CNPq fellowship Grant 306251/2014-0. RP and ACR are also part of the IRTG 1740/TRP 2015/50122-0, funded by DFG/FAPESP.

**References**

1. Tang A, Bartels AM, Sejnowski TJ. Effects of cholinergic modulation on responses of neocortical neurons to fluctuating input. *Cereb Cortex* 1997, 7, 502–509.
2. Potjans TC, Diesmann M. The cell-type specific cortical microcircuit: Relating structure and activity in a full-scale spiking network model. *Cereb Cortex* 2014, 24, 785–806.
3. Rodriguez R, Kallenbach U, Singer W, et al.Short- and long-term effects of cholinergic modulation on gamma oscillations and response synchronization in the visual cortex.*J Neurosci* 2004; 24, 10369–78.

## P30 Information processing from external inputs to the entorhinal cortex grid cells

### Anu Aggarwal

#### Central Michigan University, Engineering and Technology, Mt Pleasant, MI, United States

##### **Correspondence**: Anu Aggarwal (aaagganu@gmail.com)

*BMC Neuroscience* 2018, **19(Suppl 2):**P30

Hippocampal formation is a C-shaped structure present on the medial aspect of the cerebral hemispheres [1]. It is believed to be involved in spatial navigation and creation of new episodic memories [2], [3]. This is achieved by the unique firing patterns of the cells which comprise the HF viz., the place cells [4], head direction cells [5], [6] and the grid cells [7]. Several mathematical models have been proposed to explain these firing patterns. One of these [8], explains that the firing pattern of the medial entorhinal cortex grid cells is based on integration of animal motion in environment based on trigonometric principles and its projection on to the three rings of intermediate neurons which are aligned at 120 degrees to each other. One neuron on each of the three rings is connected to a single grid cell which fires when it receives coincident input from all the three neurons. This mathematical model explains well how the regular hexagonal firing pattern of the grid cells, as seen experimentally in animal studies might be obtained. However, this model does not explain as to how the trigonometric calculation of motion inputs will be achieved during animal motion. The present work proposes a model of how this trigonometric calculation might be occurring as the animal moves around freely in the environment. As the animal moves along a particular direction, the head direction cells provide information about the angle along which the animal is moving. Let us assume that this information is available for every degree along the azimuth. Along with this information, the neurons pre-processing (say pre-processing neuron or PN) information for the entorhinal grid cells will also receive proprioceptive information from the foot pads of the animal (assuming the animal is moving freely in the environment using its limbs). For motion along 0, 120 and 240 degree of the azimuth w.r.t. the orienting cues in the environment, the animal motion will be mapped on to the respective rings of intermediate neurons in clockwise (CW) direction. And for motion along 180, 300 and 60 degrees w.r.t. the orienting cues, the animal motion will be mapped on to the respective rings of intermediate neurons in counterclockwise (CCW) direction. For these 6 directions, thus, there is a separate PN which integrates motion and head direction information from the proprioceptive and head direction cell neurons respectively. Thus, each head direction cell neuron in head direction cell system projects to one PN while the proprioceptive inputs are provided equally to all the PN neurons. For the remaining head directions, the PN receive equal proprioceptive inputs but the head direction cell inputs are scaled according to the cosine of the head direction angle due to different strength of synapses which connect the head direction cells and the PN cell. Therefore, in all there are 360 PN cells each of which receives inputs from the corresponding head direction cell. All the 360 PN cells also receive equal proprioceptive inputs. After this calculation, the different PN cells send currents of different intensity to the rings of intermediate neurons. Connectivity between the PN cells and the rings of intermediate neurons is such that PN cells coding for 330–30 degrees will send information to the first ring for motion along the CW direction, those for 150–210 degrees to the same ring for CCW motion, those for 30–90 degrees to third ring for CCW motion and those from 210 to 270 degrees to third ring for CW motion, and those for 90–150 degrees to second ring for CW motion and those from 270 to 330 degrees to second ring for CCW motion. Thereafter, the rings process this information as per the computational model in [8] (Fig. 1).

**Fig. 1:**
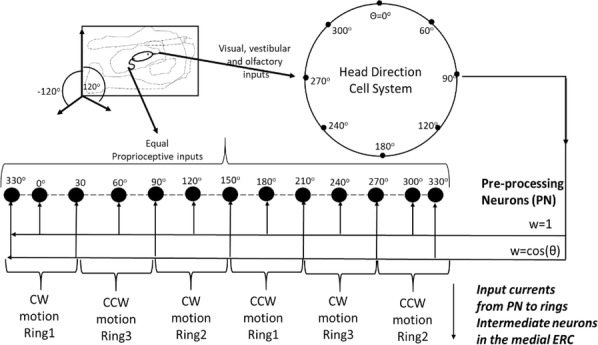
Schematic representation of the model connectivity and working

**References**

1. Purves D, Augustine GJ, Fitzpatrick D, et al. *White*, *Neurosci*ence., 5th ed., Sinauer Associates, MA, USA, 2012.
2. McNaughton BL, Battaglia FP, Jensen O, et al. Path integration and the neural basis of the ‘cognitive map’, *Nat*. *Rev*. *Neurosci*. 2006, 7, 663–678.
3. Hasselmo ME. *How We Remember*, MIT Press, Cambridge, MA, 2012.
4. O’Keefe J, Dostrovsky J. The hippocampus as a spatial map. Preliminary evidence from unit activity in the freely moving rat, *Brain Res*.1971, 34 (1), 171–175.
5. Taube JS, Muller RU, Ranck. Jr JB. Head direction cells recorded from the post-subiculum in freely moving rats. I. Description and quantitative analysis, *J*. *Neurosci*. 1990, 10, 420–435.
6. Taube JS, Muller RU, Ranck. Jr JB. Head direction cells recorded from the post-subiculum in freely moving rats. II. Effects of environmental manipulations, *J*. *Neurosci*. 1990, 10, 436–447.
7. Hafting T, Fyhn M, Molden S, et al. Microstructure of a spatial map in the entorhinal cortex, *Nature* 2005, 436, 801–806.
8. Mhatre H, Gorchetchnikov A, Grossberg S. Grid cell hexagonal patterns formed by fast self-organized learning within entorhinal cortex, *Hippocampus* 2012, 22, 320–334.

## P31 Understanding action potential evolution in axon due to focal geometric deformation using a hybrid 1D-3D model

### Yuan-Ting Wu, Ashfaq Adnan

#### University of Texas Arlington, Mechanical and Aerospace Engineering, Arlington, TX, United States

##### **Correspondence**: Yuan-Ting Wu (yuanting.wu@mavs.uta.edu)

*BMC Neuroscience* 2018, **19(Suppl 2):**P31

Localized deformation on axon is observed in many scenarios, such as traumatic brain injury, Alzheimer’s disease (AD), or multiple sclerosis (MS). Those observations open up the question—how much deformation can block or change the action potential transport? Specifically, any deviation of the near-cylindrical axonal cross section due to cell–cell contact can lead to changes in action potential. However, predicting the answer to such question is challenging. The major challenge here is the length and time scale. The characteristic length scale of a human axon spike (non-myelinated) is around 10 mm (spike to spike), but the focal geometry change can be 1—10 µm. To simulate the shape better with numerical method, the discretized size can go as small as 0.1 µm when needed. It requires roughly ~ 10,0003 number of data points for a 3-D model, or ~ 10,0002 points for a 2-D model. The other issue is the nature of the action potential. Since axon potential is a mutated wave function which is numerically unstable when solved using explicit method. For the size of 0.1 µm mesh size, a time step of 10–6 µs is needed for an explicit method, or ~ 1 µs time step for an implicit method (less overall computational resource). To solve the dilemma, we proposed a hybrid 1D-3D model for it. The model we proposed consists of two parts: 1. An one-dimensional cable theory model with Hodgkin–Huxley membrane capacitor simulating the cylindrical of before and after the deformation site. 2. A 2-D meshed finite element method (FEM) model for the deformed part and its neighbor. The 2-D model currently uses cylindrical coordinate, and it can be updated with a 3-D model in the future. The 2-D model uses the Laplace equation at the intracellular medium and the Hodgkin–Huxley capacitor at the membrane. Those two integrated models interact with each other at each time step to ensure that the simulated condition in the FEM part is representing its behavior in a long axon.

## P32 A quantitative model for estimating the scale of photochemically induced ischemic stroke

### Zhaojie Yao, Azadeh Yazdan-Shahmorad

#### University of Washington, Departments of Bioengineering & Electrical Engineering, Seattle, WA, United States

##### **Correspondence**: Zhaojie Yao (zjyao@uw.edu)

*BMC Neuroscience* 2018, **19(Suppl 2):**P32

Photothrombosis is an established technique for inducing ischemic stroke in animal models. It produces focal cerebral infarcts induced by the photodynamic effect of anionic xanthene dyes, among which Rose Bengal is most frequently used. The general procedure involves the administration of the dye in the blood stream, followed by photo radiation at the interested location. In the blood circulation system, the hydrophilic dyes bind to the vascular endothelium, platelets and other blood cells. Upon light exposure, the photoexcitation energy of the dye molecules is transferred to singlet oxygen, leading to oxidative stress. The subsequent cascade, including damage to vascular endothelium and platelet aggregation, eventually results in ischemia and neuronal death [1]. Here, we propose a computational approach to quantitatively predict the scale of the lesion in photothrombotic procedures which can offer crucial insight in the development and implementation of light-induced stroke models in animals. We modeled the relative light intensity distribution in the cortex resulted from the light-tissue interaction of a collimated beam. We based our model upon McLean’s [2] light beam spread function method, which is capable of resolving light distributions in highly forward scattering turbid media, such as the brain tissue. This method assumes that photons arrive to locations via multiple paths of various lengths, thus, resulting in time dispersion of light intensity. The impulse response, whose vertical section shown in Fig. 1A at different time points as the light propagates, was first calculated using the beam spread function. It was then temporally integrated to generate the scattering profile of a continuous pencil-beam response as shown in Fig. 1B. The pencil-beam response was subsequently convolved spatially in the transverse plain with the geometry of the beam (Fig. 1C) to acquire the intensity distribution of photon energy in tissue as illustrated in Fig. 1D. We simulated the penetration and scattering profile of 532 nm-wavelength light, which is one of the characteristic absorption wavelengths for Rose Bengal. Fig. 1E demonstrates the normalized intensity distribution of 532 nm light in the medium, and Fig. 1F is the contour of such distribution. We further illustrated that our model could estimate the spatial extent of the effective region under photothrombotic protocols (Fig. 1G). This model could also be applied in titrating the intensity of light beams used to generate infarcts of interested penetration depth (Fig. 1H). Photothrombotic models can create well-defined ischemic cerebral lesions. Our simulation quantitatively demonstrated how the scale of infarction depends on the intensity and the diameter of the beam. Our future effort would be oriented in two directions. One is to develop a multilayer model to account for the tissue-optics heterogeneity of cortex. The other direction will be to integrate our modelling approach with developing animal models of micro cortical strokes. We would calibrate and modify the models according to pathology examinations and incorporate additional stages to bridge potential mismatches between the simulation and the histology/pathology.

**Fig. 1:**
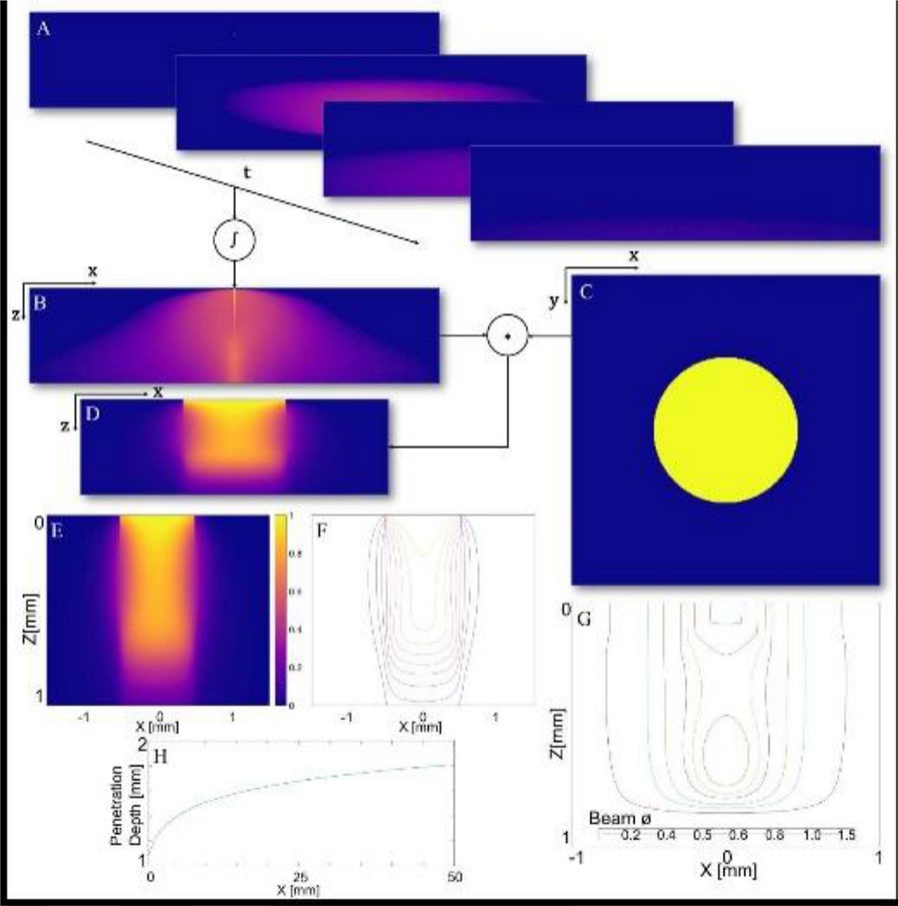
Figure A, B, C and D, Model Implementation. The impulse response, vertical section shown in A, at different time in light propagation was first calculated using the beam spread function. They were then temporally integrated to generate the scattering profile of a continuous pencil-beam response as shown in B. The pencil-beam response was subsequently convolved spatially with the geometry of the beam (C) to acquire the intensity distribution of photon energy in tissue as illustrated in D. Figure E and F, Light Intensity Spatial Distribution. E, spatial distribution of normalized intensity for a 0.5 mm-diameter beam of 532 nm light. Intensity is normalized to the unit volumetric photon power density at the tissue surface. F, normalized intensity contour for 532 nm light. Iso-intensity lines are at 0.1, 0.2, 0.3, 0.4, 0.5, 0.6, 0.7, 0.8 and 0.9. Figure G, Effective Region. Iso-intensity curves delineate normalized intensity at 1/e for 532 nm light beams of different sizes. Intensity is normalized to the unit volumetric photon power density at the tissue surface. The color in the legend indicates the diameter of the beam. Figure H, Relation between Penetration Depth and Surface Intensity. The penetration depth is defined along the central axis of the beam where the light intensity decays to 0.1 arbitrary unit

**References**

1. Uzdensky AB. Photothrombotic Stroke as a Model of Ischemic Stroke. *Translational stroke research 2017*, 1–15.
2. McLean JW, Freeman JD, Walker RE. Beam spread function with time dispersion. *Applied optics*, 1998. 37(21), 4701.

## P33 Visualization of pre-motor and parietal network activity patterns during free behavior in rats

### Medorian Gheorghiu^1^, Jonathan Withlock^2^, Raul Muresan^3^, Bartul Mimica^2^

#### ^1^Transylvanian Institute of Neuroscience, Cluj, Romania; ^2^Norwegian University of Science and Technology (NTNU), Kavli Institute for Systems Neuroscience, Trondheim, Norway; ^3^Romanian Institute of Science and Technology, Center for Cognitive and Neural Studies, Cluj-Napoca, Romania

##### **Correspondence**: Medorian Gheorghiu (medorian@gmail.com)

*BMC Neuroscience* 2018, **19(Suppl 2):**P33

The patterns of neuronal activation during planning and decision-making in freely behaving rats is little understood, partly because the cortical areas responsible for behavior integrate a multitude of sensory and motor information. Also, with freely behaving animals, the trials do not have precise length and structure, the number of events is dynamic and they depend on the decisions the animals make while exploring the environment. Here, we use a visualization technique based on color sequences [3] to investigate the expression of multi-neuron firing patterns across the posterior parietal cortex (PPC) and frontal motor cortex (AGm) that are specific to various behavioral states.

**Experimental design and behavioral paradigms:** We implemented an instructed task where the rat runs to a “Home” well with fixed location during a trial, then a free-choice exploratory task where the rat searches for a “Target” well located randomly across an arena with 36 wells. A custom made NeuroNexus micro-drive was implanted in the rat’s brain targeting PPC and AGm simultaneously (8 tetrodes in each area). Data was high-pass filtered using non-causal Gaussian 300 Hz and spikes were detected using an amplitude threshold set at four standard deviations. Spikes were sorted and the best responding cells (four from PPC and seven from AGm) were selected based on their responses during the experimental task.

**Visualization using 3D Kohonen maps:** Spike-trains were convoluted with an exponentially decaying kernel [3] yielding activity vectors by sampling the signals at a frequency of 1 kHz. Activity vectors were presented to a 3D Kohonen map for learning, where each node of the map has a dimensionality equal to the input space (the number of cells). After learning, Kohonen nodes represent cluster centers, i.e., stereotypical activity vectors. For visualization, the 3D Kohonen cube is mapped on an RGB cube, thus each node of the lattice will have a color corresponding to its position in 3D space. Activity vectors sampled from the data are compared one by one to each of the nodes so they are marked with the color of the best matching cluster found in the lattice. Therefore, each color presented in Fig 1A corresponds to a certain pattern of activity (Fig 1C). By plotting the patterns of activity along a trial, one can extract the sequence in which the cells fire over time (Fig 1B).

**Fig. 1:**
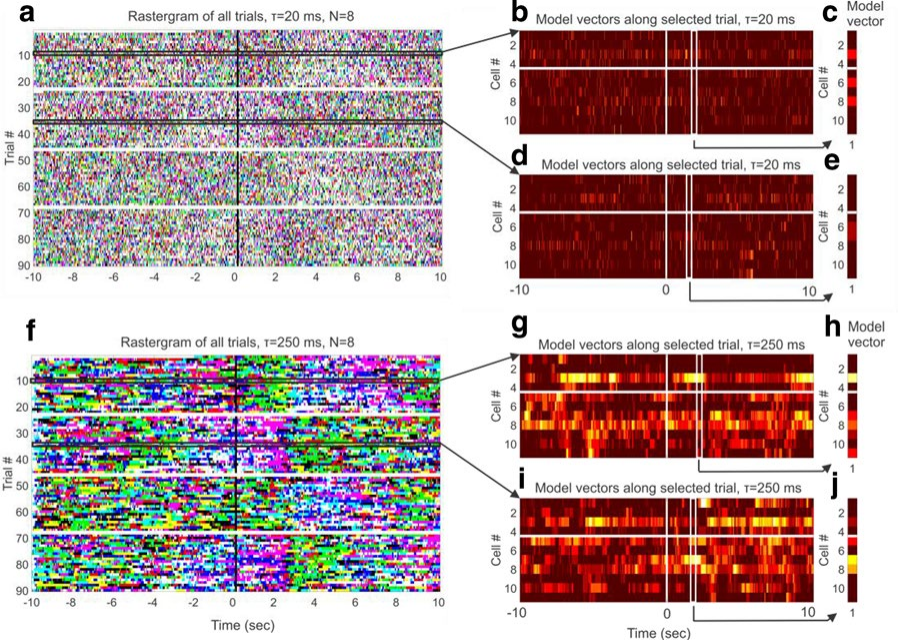
A: Activity patterns recorded from PPC and Agm of free behaving rats at multiple timescales. A and F: Rastergram of 11 cells (4 from PPC and 7 from Agm) are presented for 4 different experimental events, each event delimited by white horizontal lines. The events are (marked with a black line, from top to bottom): start “Home”, end “Home”, start “Target”, end “Target”. Any selected trials in A and F (black boxes) are composed of color coded patterns (model vectors) extracted from the 3D Kohonen map (B, D, G and I). The white boxes in B, D, G and I contain the firing patterns of the recorded neurons at a selected time t, patterns highlighted in C, E, H and J

**Conclusions:** Using the visualization technique, we can extract synchronous pattern of spikes (Fig 1A) or firing-rate patterns (Fig 1F). The synchronous patterns of spikes were not clearly detected for small τ (Fig 1A, τ = 20 ms). For large τ (Fig 1F, τ = 250 ms) a rate covariation was visible for approx. 3 s after the rats started to lick in the “Home” well, where a specific firing pattern was visible (Fig 1H). This was reflected in the color sequence as a greenish color. A different combination of cells’ firing was visible (Fig 1J) as a purple pattern immediately after the rat left the location of the “Home” well. Our results suggest that behavioral events are correlated to specific and coordinated firing patterns across PPC and AGm. These patterns evolve on relatively slow time scales (> 200 ms). A further investigation is required involving more cells to determine if joint-spikes events are present at small time scales.

**References**

1. Withlock J, Sutherland RL, Witter MP, et al. Navigating from hippocampus to parietal cortex. -*PNAS* 2008, 105(39), 14755–14762
2. Pesaran B, Nelson MJ, Andersen RA. Free choice activates a decision circuit between frontal and parietal cortex. *Nature* 2008, 453, 406–409
3. Ovidiu Jurjut, et al. *J Neurophysiol*. 2009

## P34 Mean field theory of large and sparse recurrent networks of spiking neurons including temporal correlations of spike-trains

### Sebastian Vellmer^1^, Benjamin Lindner^2^

#### ^1^Bernstein Center for Computational Neuroscience, Complex Systems and Neurophysics, Berlin, Germany; ^2^Humboldt University Berlin, Physics Department, Berlin, Germany

##### **Correspondence**: Sebastian Vellmer (sebastian.vellmer@bccn-berlin.de)

*BMC Neuroscience* 2018, **19(Suppl 2):**P34

To describe the dynamics of large and sparse recurrent networks of spiking neurons, Brunel (2000) applied a mean field approach. In this theory, neural input, as a large sum of independent spikes, is modeled as uncorrelated (white) Gaussian noise, exhibiting a flat power spectrum.This approximation corresponds to a nonlinear 1D Fokker–Planck equation (FPE).Its analytical solution, combined with the condition of self-consistence of neural input and output, allows one to predict the firing rates in the network and to distinguish its different firing regimes (e.g. synchronized and synchronous states). The condition of a white network noise, however, is only approximately fulfilled in the limit of very rare firing. In general, spike-trains (ST) from neurons exhibit temporal correlations with non-flat power spectra and the neural input in recurrent networks (network noise), being the sum of many such STs, maintains these temporal correlations (Lindner 2006). A more faithful theory of single spike trains in recurrent networks also has to take into account these temporal correlations, yielding a more comprehensive condition of self-consistence that has so far been exploited in numerical schemes to determine ST spectra (Lerchner et al. 2006, Dummer et al. (2014), Pena et al. 2018). Here we present a theoretical framework, that allows one to take into account self-consistence of ST power spectra.In this framework, the network noisecan be approximated by the projection of an N-dimensional Ornstein–Uhlenbeck process (OU), using a Markovian embedding. Spectra of OUs can be seen as a Pàde approximation, the order of which increases with the dimension. We can formulate a corresponding (N + 1)D FPE, that describes the time evolution of a neural ensemble driven by colored noise. From its solution, we obtain the ST power spectrum of the neuron. We achieve self-consistence, if the power spectrum of our stochastic process is proportional to the ST power spectrum. A finite dimensional OU cannot fit arbitrary spectra. However, it can fulfill the condition of self-consistence in several points. The number of achievable intersections of input and output is 2 N + 1. Increasing of intersections yields better approximations for the spectra of single neurons and network noise. In our theory, Brunel (2000) is a zero-order approximation, in which the spectra are only self-consistent for the frequency f → ∞, i.e. self-consistent with respect to the firing rate.In general, we lack an analytical solution for a multidimensional FPE and, hence, we need to apply numerical tools for its solution. Furthermore, higher dimensional FPEs are difficult to treat numerically. For a 1D OU, we provided a robust numerical procedure to obtain single points of the spectrum from the corresponding 2D FPE in the Fourier domain. We can achieve self-consistence for three arbitrary points of the spectrum. As in Brunel (2000), we use the firing rate, i.e. the spectrum at f → ∞. Additionally, we require self-consistence of input and output spectra at f → 0 (corresponding to the Fano factor) and at the firing rate f = r0. To find a solution, we determine the coefficients of our OU from the solution of the FPE iteratively, until self-consistence is achieved. Fig. 1 shows one example. For the chosen parameters, the resulting ST spectrum from the 2D FPE are close to that measures in a large sparse recurrent network.

**Fig. 1:**
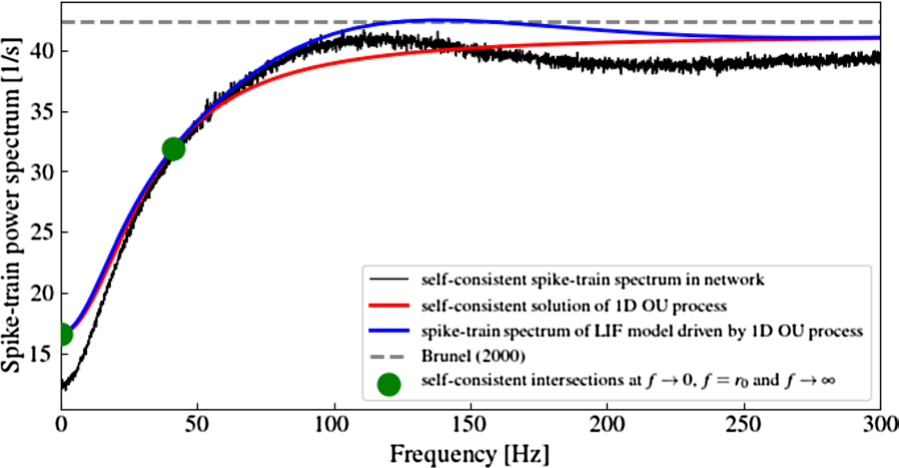
Self-consistent spike train power spectrum from a network of LIF neurons with high synaptic weights and dominating inhibition and the solution of our theory. We obtain a better approximation, where self-consistent is achieved. For a more adequate approximation, a higher dimensional OU process is required

## P35 Probabilistic analysis of high-dimensional stochastic firing rate models: Bridging neural network models and firing rate models

### Ehsan Mirzakhalili, Bogdan Epureanu

#### University of Michigan, Department of Mechanical Engineering, Ann Arbor, MI, United States

##### **Correspondence**: Ehsan Mirzakhalili (mirzakh@umich.edu)

*BMC Neuroscience* 2018, **19(Suppl 2):**P35

Advances in the characterization of neurons and the increase in computational capacity has enabled researchers to build larger and more detailed models of neural networks. While such models have proven to be helpful, the interpretation of results obtained from such models is not straightforward due the lack of necessary analytical and mathematical tools. Hence, a framework that enables rigorous analysis of detailed network models is invaluable. To establish our proposed framework, we start with a network that can resemble working memory. The duration of the recall and the average firing rate during the recall are used to quantify the characteristics of such network models. The mechanisms that can affect these metrics can be studied by varying different parameters of the model one by one. However, such analysis cumbersome in large detailed networks. Alternatively, rate models can be constructed that can faithfully represent key dynamics of detailed network models especially if noise is incorporated in such rate models. Rate models are attractive not only because they are computationally efficient, but because they can be analyzed based on a rich mathematical foundation of dynamical systems. Hence, the effect of parameters of the model on the working presence of working memory can be studied by examining of the bifurcation diagram of rate models that correspond to such network models. Such deterministic bifurcation analyses can only show the existence of multiple stable or unstable solutions for a firing rate model, which is not enough to describe dynamics of network models. However, adding noise to rate models enables establishing the connection between rate models and network models by allowing calculation of metrics such escape time and probability of finding the system in each point on the bifurcation diagram. Calculation of such metrics and the effects of noise on the bifurcation analysis of such dynamical systems and have not been investigated previously for analysis of dynamics of neural networks. In this research, we introduce a probabilistic framework that contains a stochastic bifurcation analysis of rate models in the presence of noise. We focus first on models that consist of an excitatory population and an inhibitory population. Stochastic differential equations are formulated for firing rate models by considering the states to be large, but the noise to be comparatively small. Hence, a linearization of the firing rate function with respect only to noise can be accurate. Next, the system of stochastic differential equations is converted to a Fokker–Planck equation. The stationary solution of the Fokker–Planck partial differential equation reveals the probability of finding the system at a certain firing rate. We solve the Fokker–Planck equation numerically to find such stationary solutions at various parameter values, hence building stochastic bifurcation diagrams. The results obtained from the stationary solutions of the Fokker–Planck equation shows how noise can change the probability of finding the system in each of the solutions in the bifurcation diagram. The results show that the same magnitude of noise can affect each stable solution differently. Therefore, evaluating the probability distribution of solutions to rate models can increase the capability of these models to analyze network population activity obtained from experiments or numerical models.

## P36 Input oscillations may stabilize working memory activity

### Nikita Novikov^1^, Boris Gutkin^2^

#### ^1^St.Petersburg School of Economics, Higher School of Economics, Moscow, Russian Federation; ^2^École Normale Supérieure, Paris, France

##### **Correspondence**: Nikita Novikov (nikknovikov@gmail.com)

*BMC Neuroscience* 2018, **19(Suppl 2):**P36

Working memory (WM) is the ability to temporarily maintain information about stimuli that are no longer present in the sensory systems. WM retention is associated with elevated firing rates in the neural populations that encode the memorized stimuli [1]. Classically, WM is modelled as a bistable system with a background low-activity state and the high-firing rate state that corresponds to the memory being retained [2]. Alongside with the firing ratess, oscillatory activity is also modulated during WM retention, notably one observes an increase of beta power in the stimulus-selective prefrontal populations [3]. One hypothesis is that the beta oscillations stabilize the persistent WM, thereby preserving a status quo [4]. However, the mechanisms for this network stabilization are not understood.

In this work, we propose a mechanism that allows to stabilize WM retention in the presence of distractors by a non-selective beta-oscillatory input. We consider two identical excitatory-inhibitory populations described by Wilson-Cowan like equations, the first one being selective to the stimulus (S), the second—to the distractor (D). The populations are bistable and coupled by mutual inhibition, so only one of them could be in the memory state at the same time. Both populations received beta-band oscillatory input. In our model, the memory state (as opposed to the background state) is associated with beta-band resonance. Consequently, the oscillatory input entrained a population only if it was in the memory state. Furthermore, oscillatory entrainment produced an increase of firing rate in the population, due to the non-linear properties of input–output relation of neurons.

We use our model to simulate a prototypical WM task (Fig. 1). Initially, both populations were in the background state. Then we delivered an excitatory pulse to the S-population (stimulus presentation), switching it to the memory state. Subsequently a similar “distractor” pulse was delivered to the D-population. We compared the responses of the model with and without the background oscillatory forcing. External beta-band input to both populations selectively excited the S-population, which lead to additional inhibition of the D-population (due to S- > D inhibitory connection). In such condition, presentation of the distractor was not enough to switch the D-population to the active state, so the state of the system (S is active, D is inactive) was preserved. Without oscillatory input, the D-population activated, returning the S-population to the background. Thus, we found that beta-band forcing prevented the distractor from disturbing the beta-resonant WM trace.

**Fig. 1:**
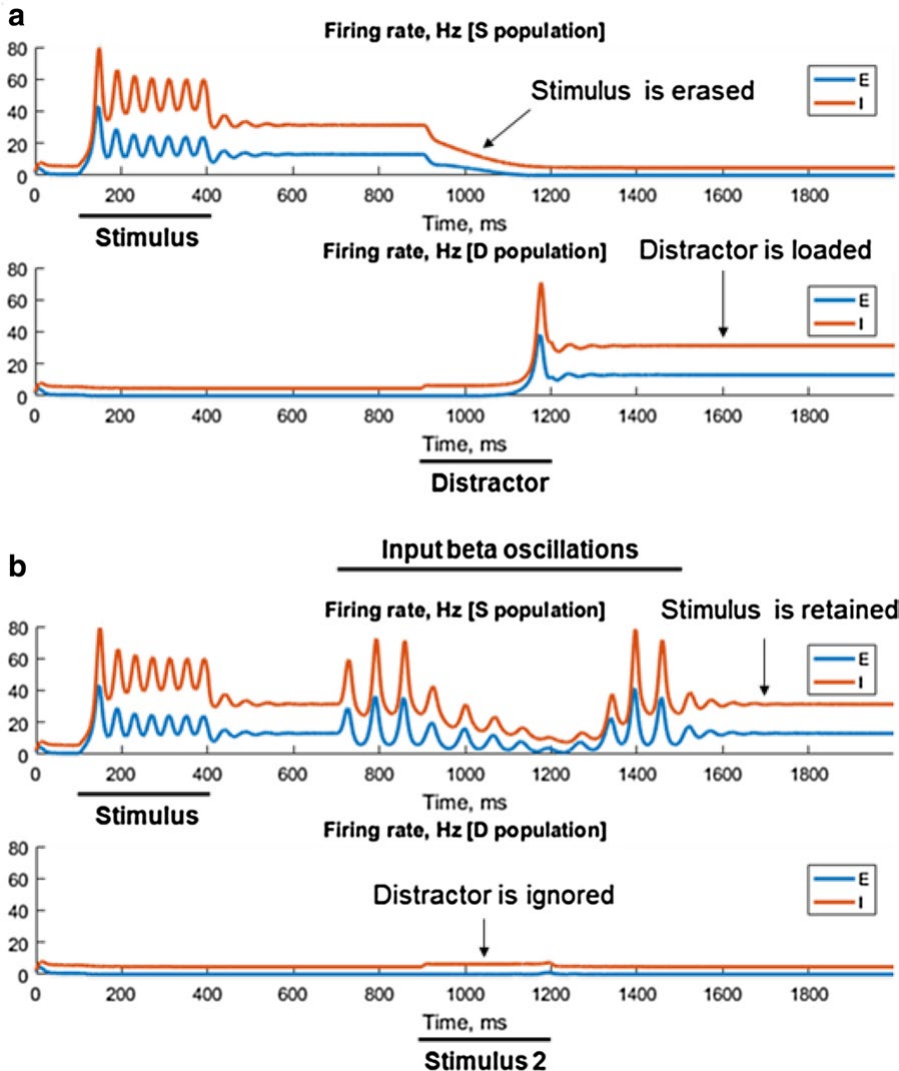
Results of the simulation. A. No input oscillations. B. Beta-band input oscillations. Blue: excitatory firing rates, red: inhibitory firing rates. Top panels: S-population, bottom panels: D-population

**Acknowledgement**

Supported by Russian Science Foundation grant (No: 17-11-01273).

**References**

1. Goldman-Rakic PS: Cellular basis of working memory. Neuron 1995, 14(3):477–485.
2. Amit DJ, Brunel N: Model of global spontaneous activity and local structured activity during delay periods in the cerebral cortex. Cereb Cortex 1997, 7(3):237–252.
3. Lundqvist, M, Rose, J, Herman, P, Brincat, SL, Buschman, TJ, Miller, EK: Gamma and Beta Bursts Underlie Working Memory. Neuron 2016, 90(1):152–164
4. Engel, AK, Fries, P: Beta-band oscillations—signalling the status quo? Curr Opin Neurobiol 2010, 20(2):156–165

## P37 Artificial evolution of networks of artificial adaptive exponential neurons for multiplicative operations

### Muhammad Khan, Borys Wrobel

#### Adam Mickiewicz University in Poznan, Evolving Systems Laboratory, Poznan, Poland

##### **Correspondence**: Borys Wrobel (wrobel@evosys.org)

*BMC Neuroscience* 2018, **19(Suppl 2):**P37

Although neuronal firing often represents the sum of inputs, many computations require multiplication. Multiplication by a constant corresponds to a change of slope (gain) of the input–output relationship of a neuron or network [1]. Such gain modulation have been observed in individual biological neurons in vivo [1, 2]. In this work, however, we evolve a network (rather than single neurons) of model neurons (adaptive exponential integrate and fire, [3]) to perform multiplicative operations. The networks were evolved using a model of evolution of complex networks [4] in which the structure of the networks is encoded in linear genomes in a way inspired by RNA world), and allows for addition or removal of connections in the network, changes of synaptic weights, and addition or removal of interneurons. The fitness function in the artificial evolution rewarded for the correct number of spikes in response to the input (with linear or non-linear input–output relationship, and maximum firing rate lower than 200 Hz; Fig. 1); this correct number of spikes varied depending on the level of modulation (a second input to the network), with the expected response that corresponded to a multiplicative operation. Both the input and modulation were encoded as a varying level of synaptic activation of interneurons, and the number of spikes on the output was measured over 240 ms. We have evolved small networks of spiking neurons with both linear and non-linear (exponential, sigmoid) input–output relationships (Fig. 1). Our preliminary results indicate that it is possible to evolve small networks for such a task, and suggest, perhaps unsurprisingly, that larger networks allow for a more precise computation of this type.

**Fig. 1:**
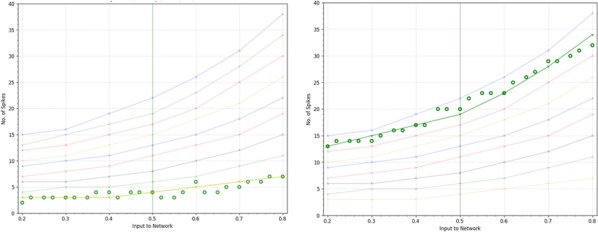
The input–output relationship of the network for two extreme levels of modulation the network was evolved for, corresponding to multiplying the response for low modulation (left panel) by about 5 for high modulation (right panel)

**Acknowledgements**

This work was supported by the Polish National Science Center (project EvoSN, UMO-2013/08/M/ST6/00922). MAK acknowledges the support of the PhD programme of the KNOW RNA Research Center in Poznan (No. 01/KNOW2/2014). We are grateful to Volker Steuber and Neil Davey for discussions.

**References**

1. Rothman JS, Cathala L, Stuber V, Silver RA. Synaptic depression enables neuronal gain control. *Nature* 2009, 457, 1015–1018
2. Chance FS, Abbott LF, Reyes AD. Gain Modulation from Background Synaptic Input. *Neuron* 2002, 35, 773–782.
3. Brette R, Gerstner W. Adaptive exponential integrate-and-fire model as an effective description of neuronal activity. *Journal of Neurophysiology* 2005, 94, 3637–3642.
4. Yaqoob M, Wróbel B. Very small spiking neural networks evolved to recognize a pattern in a continuous input stream. *in 2017 IEEE Symposium Series on Computational Intelligence*, 2017.

## P38 Artificial evolution of very small spiking neural network robust to noise and damage for recognizing temporal patterns

### Muhammad Yaqoob, Borys Wrobel

#### Adam Mickiewicz University in Poznan, Evolving Systems Laboratory, Poznan, Poland

##### **Correspondence**: Borys Wrobel (wrobel@evosys.org)

*BMC Neuroscience* 2018, **19(Suppl 2):**P38

Biological systems maintain their functionality in presence of noise and damage (for a more detailed introduction, see [1]). Such robustness can stem from redundancy of elements, but large artificial neural networks are not necessarily more robust than very small networks. In order to understand how robustness emerges in spiking neural networks performing simple computational tasks, we have evolved very small networks of adaptive exponential integrate and fire neurons [2]. The networks consisted of 3 inputs, and up to 4 adaptive exponential neurons: up to 3 interneurons, and 1 output, with inputs connecting to interneurons, interneurons to output, and recurrent connections allowed between interneurons. The task of the networks was to produce spike(s) on output when the inputs present signals to the interneurons in a certain order (Fig. 1). Each signal lasts for 6 ms and is followed by silence on all inputs for 16 ms. The artificial evolutionary process (in which the structure of the network is encoded in linear genomes in a way inspired by RNA world; see [1] for details) allows for addition or removal of connections in the network, changes of synaptic weights, and addition or removal of interneurons [3]. We have investigated the evolution of such networks in presence of additive Gaussian noise (SD 2 mV) on membrane potential, and were successful in obtaining networks who were almost perfect (responding with one spike to all target subsequences in the output, with hardly any spikes elsewhere). Our results show that evolved networks were very robust to perturbation of neuronal parameters. The best network from 50 independent runs was robust to changes of all neuronal parameters tested, such as effective resting potential (evolved for − 70 mV, range of robustness: [− 60, − 83] mV), reset potential after the spike (− 58; [− 45, − 63] mV), spike initiation threshold potential (− 50; [− 48, − 53] mV), membrane time constant (20; [9, 100] ms), slope factor (2; [1.0, 2.7] mV), membrane capacitance (0.2; [0.18, 0.23] nF), subthreshold adaptation conductance (2; [− 10, 17] nS), and spike-initiated adaptation (0; [0, 40] pA). Moreover, we have also observed that the evolved networks are robust to variation on the length of silences (evolved for 16 mS, robustness range [10, 50] ms) between signals and the length of signals (6; [5, 7] ms), indicating that such network can maintain their state.

**Fig. 1:**
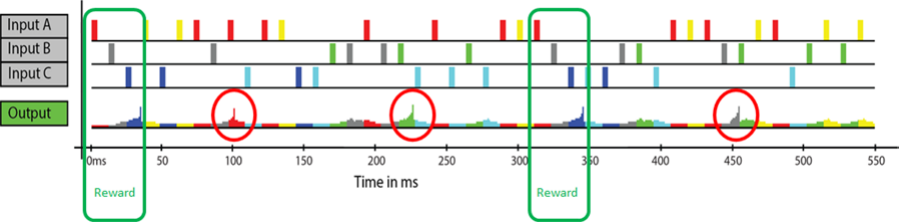
The input and response of a suboptimal network. The top three lines show the input signals, colour coded for the order in which they appear (to facilitate the analysis of the network activity). The bottom line shows the voltage of the output neuron (the activity of the interaneurons is not shown). During artificial evolution, the spikes after the input sequence “A B C” are rewarded (green rectangles) and any other spikes (red circles) are penalized (such spikes occur very rarely in the response of the best networks at the end of the successful evolutionary runs)

**Acknowledgements**

This work was supported by the Polish National Science Center (project EvoSN, UMO-2013/08/M/ST6/00922). MY acknowledges the support of the PhD programme of the KNOW RNA Research Center in Poznan (No. 01/KNOW2/2014). We are grateful to Volker Steuber and Neil Davey for discussions.

**References**

1. Wróbel B. Evolution of spiking neural networks robust to noise and damage for control of simple animats, in *Parallel Problem Solving from Nature*—PPSN XIV, 2016.
2. Brette R, Gerstner W. Adaptive exponential integrate-and-fire model as an effective description of neuronal activity, *Journal of Neurophysiology* 2005, 94, 3637–3642.
3. Yaqoob M, Wróbel B. Very small spiking neural networks evolved to recognize a pattern in a continuous input stream, *IEEE Symposium Series on Computational Intelligence*, 2017.

## P39 Population and single-neuron measures of multisensory integration

### Brian Fischer

#### Seattle University, Department of Mathematics, Seattle, WA, United States

##### **Correspondence**: Brian Fischer (fischer9@seattleu.edu)

*BMC Neuroscience* 2018, **19(Suppl 2):**P39

Perceptual detection and estimation may be improved in the multisensory condition relative to the unisensory condition by combining evidence across sensory modalities. The integrative properties of multisensory neurons are often evaluated by comparing the relative strength of responses to unimodal and multimodal stimuli using the multisensory enhancement index [1]. However, it remains unknown which features of multimodal neural responses lead to enhanced perceptual performance in multisensory conditions. Here we use a model for the neural implementation of Bayesian inference to reassess the commonly used multisensory enhancement index as a measure of multisensory integration. The non-uniform population code model describes how populations of neurons can perform Bayesian inference. It assumes that the population structure is matched to the statistics of the environment, where preferred stimuli are drawn from the prior distribution and the neural population response to a stimulus is proportional to the likelihood function. It has been shown that, in this framework, optimal cue combination is multiplicative at the population level [2]. Specifically, a center-of-mass decoding of the population will approximate a Bayesian estimate when the population response to multiple stimulus cues is proportional to the produce of the responses to the individual cues. We show here that the mechanism used to implement multiplicative selectivity determines whether multisensory enhancement is correlated with performance enhancement. In the non-uniform population code, optimal multisensory integration only depends on the pattern of activity over the population, not the strength of the responses. Therefore, if neurons implement perfect multiplication of their inputs, multisensory enhancement will be unrelated to performance. If neurons implement an approximation multiplication, then multisensory enhancement may be correlated with performance enhancement. Specifically, we examined the responses of model neurons that use a sigmoid input–output transformation to perform approximate multiplication [3]. In this network, both the accuracy of the multiplication and the enhancement index changed depending on where the input fell on the sigmoidal curve. Thus, multisensory enhancement was correlated with performance enhancement because it was correlated with the accuracy of the multiplication. Therefore, in this framework, multisensory enhancement may be correlated with, but is not causally related to performance enhancement. This work highlights the importance of using population measures to determine which features of multisensory neural responses lead to enhanced perceptual performance in multisensory conditions.

**References**

1. Stein BE, Stanford TR. Multisensory integration: current issues from the perspective of the single neuron. *Nat Rev Neurosci*. 2008;9: 255–266.
2. Fischer BJ, Peña JL. Optimal nonlinear cue integration for sound localization. *J Comput Neurosci*. 2017;42: 37–52.
3. Fischer BJ, Anderson CH, Peña JL. Multiplicative auditory spatial receptive fields created by a hierarchy of population codes. *PLoS One*. 2009;4: e8015.

## P40 Optimizing deep convolutional network architectures to match visual cortex

### Bryan Tripp

#### University of Waterloo, Systems Design Engineering, Waterloo, Canada

##### **Correspondence**: Bryan Tripp (bptripp@gmail.com)

*BMC Neuroscience* 2018, **19(Suppl 2):**P40

It is a significant challenge to develop physiologically grounded neural models that produce ethologically relevant behavior. Deep convolutional networks have potential in this direction, as they can perform fairly realistic visual processing. However, many aspects of their behavior, activity, and mechanisms are unrealistic. For example, although the architectures of well-known convolutional networks (e.g. ResNet) were inspired by primate visual cortex, they are dissimilar in their specific layers and connections, and in the statistics of these connections (e.g. distributions of sparseness and in-degree). If convolutional networks had physiologically realistic architectures, they could be compared more directly with the brain. In this direction, the current study optimizes convolutional network hyperparameters to produce networks that match various anatomical and physiological data. The networks have one-to-one homologies with primate visual areas. The main steps in this approach are as follows: (1) assemble data related to physiological network architecture (e.g. receptive field sizes in each area; fraction of inputs to each area that come from each source) from databases and literature; (2) find mathematical expressions for these network properties in terms of convolutional network hyperparameters; (3) define a cost function based on the difference between physiological parameters and corresponding network parameters; (4) find hyperparameters that minimize the cost. Care is needed in formulating the cost function, to ensure consistency between receptive field sizes and spatial resolution across converging paths. If the optimization step is successful, this procedure produces a convolutional network architecture that is driven by physiological data. A convolutional layer generally corresponds to a single layer of a single cortical area. Physiological parameters associated with a layer are the number of neurons (estimated from cell density, layer thickness, and cortical surface area), the number of extrinsic inputs per neuron (estimated from cell reconstructions), and receptive field sizes (estimated from electrophysiology studies). Parameters associated with inter-area connections include the fraction of neurons innervating each target that come from each source area, and the percentage of supragranular versus infragranular cells that contribute to these projections.

The optimization is a non-convex integer programming problem, a type of problem that can be difficult in general. However, good results have been consistently obtained so far by converting integer parameters to floating point numbers, optimizing with the Adam algorithm, and rounding the results. Work in progress includes accounting for varying degrees of certainty of different parameters, and testing networks derived with these methods on standard vision problems such as CIFAR-10. Open-source code is available from https://github.com/bptripp/calc. Future work will include application of this approach to a model of visually guided grasping. It is also hoped that the approach can be generalized to mouse and human networks.

This work is a step toward physiologically grounded neural models that produce ethologically relevant behavior. Multiple other steps are not addressed here, include developing realistic learning experiences. Ultimately, such models may lead to new insights into relationships between low-level mechanisms, representations, and behavior.

## P41 SIMNETS: a novel mathematical framework to detect functional neuronal sub-ensembles

### Jacqueline Hynes^1^, David Brandman^2^, John Donoghue^1^, Carlos Vargas-Irwin^1^

#### ^1^Brown University, Department of Neuroscience, Providence, RI, United States; ^2^Brown University, Department of Engineering, providence, RI, United States

##### **Correspondence**: Jacqueline Hynes (jacqueline_hynes@brown.edu)

*BMC Neuroscience* 2018, **19(Suppl 2):**P41

The biological computations underlying sensory perception, cognition, and goal-directed movements are thought to emerge from interactions across large groups of cortical neurons. While it is now possible to record ever larger neural populations, detecting functional groupings of neurons and characterizing their computational operations has proven notoriously difficult. The problem of finding functional groupings is at one level statistical in nature, i.e., showing that neurons belonging to one group of neurons are significantly more similar in function than other neurons. In cortical areas where the tuning properties of individual neurons are well established, the task of grouping neurons according to their functional similarities could seem like a trivial matter (e.g., shared orientation tuning). However, a growing number of studies have shown that these simple tuning models are often task or context-dependent and will break down under more complex or ethologically relevant conditions. Although measures of coordinated spike activity have been widely used to infer the functional relationships between neurons, statistical and theoretical issues have limited the success of this approach. Here, we introduce a new approach for identifying NETworks of functionally SIMilar neurons (SIMNETS). Our approach is based on the premise that we can characterize the computation being performed by a neuron by examining the intrinsic relationship between the outputs (spike trains) it emits across different sets of inputs. We can represent these relationships using a pairwise distance matrix, where each entry represents the similarity between two spike trains. We refer to this as a ‘trial similarity matrix’ (TSM). Comparing the TSMs of simultaneously recorded neurons allows us to quantify the relationship between their computational properties. The SIMNETS algorithm involves: (i) calculating the similarities between different spike-train time-series generated by a single neuron on a neuron-by-neuron basis, (ii) calculating the correlation between the resulting TSMs to produce and NxN Correlation matrix (NCM), and (iii) using dimensionality reduction tools combined with agglomerative clustering techniques to identify neurons with similar functional properties within the NCM. We have tested the SIMNETS algorithm using synthetic data with known ground truth. Results show that SIMENTS can identify groups of neurons with similar computational properties, even if they use different encoding schemes (based on firing rate or precise spike timing). We also demonstrate that clustering performance is severely impaired using standard approaches that directly compare spike-trains*between*different neurons. To show the generality of the method, we applied SIMNETS to two publicly available datasets, including 112 primate V1 neurons recorded during the presentation of drifting gratings and a dataset of 80 rat hippocampal neurons during a navigation task. Our results shown that our algorithm can detect groups of functionally related neurons within these diverse neuronal populations. The SIMNETS framework provides a principled way to describe the relationship between neurons and determine if functional categories are present, without having to impose specific encoding models*a priori*. This data driven approach will greatly facilitate the analysis of networks of neurons engaged during complex natural behaviors.

## P42 Finite size effect for spiking neural network with spatially dependent coupling

### Siwei Qiu, Carson Chow

#### National Institute of Health, NIDDK, Lab of Biological Modeling, Bethesda, MD, United States

##### **Correspondence**: Siwei Qiu (siwei.qiu@gmail.com)

*BMC Neuroscience* 2018, **19(Suppl 2):**P42

We study finite-size fluctuations in a deterministic coupled spiking neural network with nonuniform coupling. We generalize a previously developed theory of finite size effects for globally coupled neurons. In the uniform case, mean field theory is well defined by averaging over the network as the number of neurons in the network goes to infinity. However, for nonuniform coupling it is no longer possible to average over the entire network. We show that if the coupling function approaches a continuous function in the infinite system size limit then an average over a local neighborhood can be defined such that mean field theory is well defined. We then derive a perturbation expansion in the inverse system size around the mean field limit for the covariance of the synaptic drive. We also show that the fluctuations in the firing rate of a neuron cannot be computed perturbatively in a similar series.

## P43 A space–time continuum in the hippocampus?

### Tristan Aft^1^, Sorinel Oprisan^1^, Mona Buhusi^2^, Catalin Buhusi^2^

#### ^1^College of Charleston, Department of Physics and Astronomy, Charleston, SC, United States; ^2^Utah State University, Department of Psychology, Logan, UT, United States

##### **Correspondence**: Tristan Aft (afttj@g.cofc.edu)

*BMC Neuroscience* 2018, **19(Suppl 2):**P43

Spatial and temporal dimensions are fundamental for orientation, adaptation, and survival or organisms. Hippocampus has been identified as the main neuroanatomical structure involved both in space and time perception. It has been hypothesized that hippocampus may be in fact involved in conceptual understanding of many other dimensions. The spatial position of an animal can be reliably decoded from the neuronal activity of several cell populations in the hippocampus. In particular, place cells in the hippocampus fire at only a few locations in a spatial environment, and the position of the animal can be readily read out from single active neurons. It has been recently found that some neurons in the hippocampus, called “time cells”, fire as time cells coding the time interval during a behavioral task. In this study we investigated the interval timing, i.e. the ability of perception and use of durations in the supra-second range. One important characteristic of interval timing is the scale invariance, i.e., the time-estimation error seems to linearly increase with the estimated duration. Scale invariance is extremely stable over behavioral, lesion, pharmacological, and neurophysiological manipulations. Scale invariance has been observed also across species from invertebrates to fish, birds, and mammals, such as mice, rats, and humans. Although the neuroanatomy of interval timing is still under debate, hippocampal lesions have been shown to affect peak time in peak-interval procedures. For example, dorsal hippocampal (DH) lesions produced leftward shifts in peak times while ventral hippocampal (VH) lesions produced a temporary rightward shift of peak times. We mathematically modeled the hippocampus memory of time as a random variable with a wide range of values around the desired criterion time. The key assumption of our study is that the hippocampus creates a topological map of durations, similar to the spatial map created by place cells. As a result, we successfully modeled peak shift due to the extent and location of the lesions and were able to identify the effect of lesions on scale invariance of interval timing.

## P44 Bayesian filtering of uncertain sensory data in the brain: Hamilton’s principle approach

### Chang Sub Kim

#### Chonnam National University, Department of Physics, Gwangju, Republic of Korea

##### **Correspondence**: Chang Sub Kim (cskim@jnu.ac.kr)

*BMC Neuroscience* 2018, **19(Suppl 2):**P44

We cast the free energy principle (FEP) in the neurosciences following the principles of mechanics, which articulates that all living organisms are evolutionally self-organized to tend to minimize the sensory uncertainty about environmental encounters.The FEP is a recent endeavor trying to answer to `what is life?’[1], life is characterized by temporal regularity and self-adaptiveness, which may be encapsulated, in contemporary terms, into autopoiesis and enaction.The FEP suggests that the organisms implement minimization by calling forth the informational FE (IFE) in the brain and thatthe time-integral of the IFE gives an estimate of the upper bound of the sensory uncertainty [2].We propose that the minimization of the IFE must continually take place over a finite temporal horizon of an organism’s unfolding environmental event.Our scheme is a generalization of the conventional theory which approximates minimization of the IFE at each point in time when it performs the gradient descent computation [3]. We adopt the Laplace-encoded IFE as an informational Lagrangian in implementing the variational FEP in the framework of the principle of least action (Hamilton’s principle) [4].And, by subscribing to the standard Newtonian dynamics, we consider the IFE a function of position and velocity as the metaphors for the organism’s brain variable and their first-order time derivative, respectively.The brain variable maps onto the first-order sufficient statistics of the probability density launched in the organism’s brain to perform Bayesian filtering of noisy sensory data called recognition dynamics (RD). In the ensuing Hamiltonian formulation, the RD prescribes momentum, conjugate to a position, as a mechanical measure of prediction error weighted by mass, the precision. We apply our formalism to a biophysically grounded model for neuronal dynamics by suggesting that the large-scale architecture of the brain be an emergent coarse-grained description of the interacting many-body neurons. The resulting RD is deterministic and hierarchical, which notably incorporates dynamics of both predictions and prediction errors of the perceptual states [5]. Consequently, the detail of the neural circuitry from our formulation differs from those supported by the generalized filtering which generates only dynamics of predictions of the causal and hidden states, not their prediction errors [6]. However, the general structure of message passing, namely descending predictions and ascending prediction errors in the hierarchical network, shows the close similarity.

**References**

1. Schrödinger E: *What is Life? Mind and Matter*, Cambridge: Cambridge Univ. Press: 1967.
2. Friston K (2010). The free-energy principle: a unified brain theory?*Nature Review Neurosci*, 11: 127–138.
3. C L, Kim C S, McGregor S, and Seth A K (2017). The free energy principle for action and perception: A mathematical review, *Journal of Mathematical Psychology*. 81: 55–79. http://dx.doi.org/10.1016/j.jmp.2017.09.004.
4. LandauLP and Lifshitz E M:*Classical Mechanics*, 3rd Edition, Amsterdam: Elsevier Ltd.1976.
5. Kim C S (2018). Recognition dynamics in the brain under the free energy principle. *Neural Computation*, submitted.https://arxiv.org/abs/1710.09118.
6. Friston. K, Stephan K, Li B, and Daunizeau J (2010). Generalized filtering. *Mathematical problems in engineering*. 261670.

## P45 Simultaneous recording of micro-electrocorticography and local field potentials for decoding rat forelimb movement

### Jinyoung Oh^1^, Soshi Samejima^1^, Abed Khorasani^1^, Adrien Boissenin^1^, Sam Kassegne^2^, Chet Moritz^1^

#### ^1^University of Washington, Rehabilitation Medicine, Seattle, WA, United States; ^2^San Diego State University, Mechanical Engineering, San Diego, CA, United States

##### **Correspondence**: Jinyoung Oh (ohj7@cs.washington.edu)

*BMC Neuroscience* 2018, **19(Suppl 2):**P45

**Introduction:** In the development of a brain-computer interface, the type of signal recording method is crucial. To date, a closed-loop neural interface can improve hand and arm function for individuals by using intracortical recordings to control muscle stimulation. Here we explore whether using brain surface signals recorded via electrocorticography (ECoG) is sufficient for decoding forelimb movement and compare the results to simultaneous intercortical recordings of local field potentials (LFP) in rats.

**Methods:** Three Long-Evans rats received implantations of both intracortical microwires and micro electrocorticography (µECoG) electrodes targeting the left forelimb sensorimotor cortex. Before implantation, the animals were trained for a novel lever task allowing us to measure forelimb extension movement. While animals performed this task, we recorded brain activity from 16 intracortical electrodes and 12 µECoG electrodes (Fig. 1A). The movement-related information was identified in the high frequency band (> 150 Hz). To decode the animals’ movement, a Canonical Correlation Analysis Filter (CCA) algorithm was applied to the multi-channel envelopes of high-gamma signals for both methods. Finally, we compared the quality of the decodes based on each recording method.

**Fig. 1:**
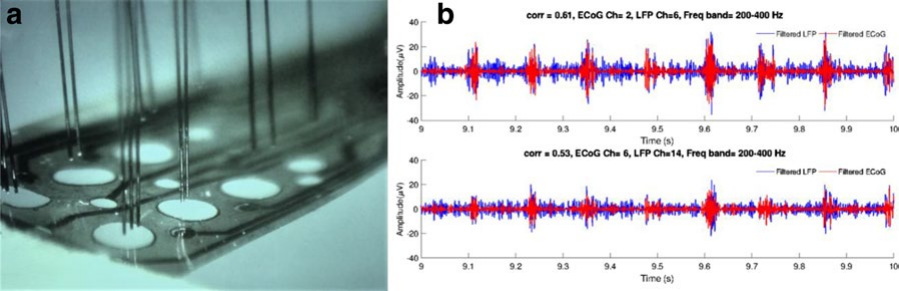
(a) Alignment of Intracortical microarrays and µECoG electrodes prior to implantation. (b) Filtered signals from of intracortical LFP and µECoG during the lever tasks

**Results:** Figure 1B illustrates the filtered signal 200–400 Hz of both the µECoG electrode array and the intracortical array and demonstrates a high correlation between two signals (correlation coefficient r > 0.5). The decoding performance of µECoG was similar to LFP for this lever task in all three animals (LFP: r = 0.48 +- 0.05, µECoG: 0.45 +- 0.06, p > 0.05).

**Discussion:** Our results suggest that µECoG may replace intracortical LFP functionally when developing a closed-loop brain-computer interface that decodes forelimb movement. Less invasive recordings with less required power due to smaller recording frequency bandwidth will likely speed the development of a clinically viable closed-loop brain-spinal interface. In addition, signal processing must be kept efficient in order to process all signals on the implanted device. Here we find that µECoG processed as described above allows the decoding of forelimb movement in similar accuracy compared to LFPs. Computational efficiency may be a substantial advantage when designing the clinical neural devices to treat brain and spinal cord injury.

## P46 Predicting the effects of deep brain stimulation using a coupled oscillator model

### Gihan Weerasinghe^1^, Benoit Duchet^1^, Rafal Bogacz^1^, Christian Bick^2^

#### ^1^University of Oxford, Nuffield Department of Clinical Neurosciences, Oxford, United Kingdom; ^2^University of Oxford, Mathematical Institute, Oxford, United Kingdom

##### **Correspondence**: Gihan Weerasinghe (gihan.weerasinghe@ndcn.ox.ac.uk)

*BMC Neuroscience* 2018, **19(Suppl 2):**P46

Deep brain stimulation (DBS), as it is currently available, involves administering a constant frequency pulse train via electrodes implanted into the brain and is known to be an effective treatment for a variety of neurological disorders, including Parkinson’s Disease and Essential Tremor (ET). There is significant evidence to suggest that the ‘closed loop’ approach of delivering stimulation according to the ongoing symptoms of the patient has the potential to improve both the effectiveness and efficiency of the treatment. The success of closed loop DBS depends on being able to devise a stimulation strategy according to the measurable and quantifiable symptoms of the patient. A useful stepping stone towards this is to construct a mathematical model which can describe the dynamics of the oscillations in addition to describing how such oscillations should change as a result of applying stimulation. Our work focuses on the use of the Kuramoto model to describe tremor oscillations found in patients with ET. We show how this model can capture the basic dynamics of tremor oscillations found in such patients and then, using a reduced form of the Kuramoto model, we derive expressions which describe how a patient should respond to stimulation at a given phase and amplitude. We predict that, provided certain conditions are satisfied, the best stimulation strategy should be phase specific but also that applying stimulation at lower amplitudes should have a greater effect. We support this surprising prediction with some preliminary results obtained from ET patients. In light of our predictions, we also propose a new hybrid strategy which effectively combines two of the strategies found in the literature, namely phasic and adaptive DBS.

## P47 Retinal motion-detection under noisy conditions

### Frances Chance, Christina Warrender

#### Sandia National Laboratories, Department of Neural and Data-Driven Computing, Albuquerque, NM, United States

##### **Correspondence**: Frances Chance (fschanc@sandia.gov)

*BMC Neuroscience* 2018, **19(Suppl 2):**P47

While retinal circuitry may appear simple compared to higher-level areas of the brain, the retina contains a surprising diversity of retinal ganglion types (detecting motion, color, etc.) that perform an equally wide range of computations to “preprocess” visual input before transmission through the optic nerve. It is often assumed that specific retinal ganglion cell types are selective for visual features that are particularly useful for encoding visual stimuli (e.g. center-surround cells) or particularly relevant for an animal’s perceptual world (e.g. sensitivity for looming stimuli), comprising a behaviorally-relevant information channel encoding specific information about the visual environment. This research focuses on motion-sensitive retinal ganglion cells. Specifically, we ask which types of motion-sensitive cells perform best under challenging conditions, for example when the moving target is dim relative to the background or under noisy conditions and are particularly interested in understanding which ganglion cell types are best suited for incorporation into a neuromorphic system for specific visual tasks. We construct a number models of retinal ganglion cell types implicated in motion-processing, including direction-selective models, such as the Hassenstein & Reichardt model [6] or the Barlow-Levick model [2], as well as motion-sensitive cell types, such as the OMS (object-motion sensitive) cell [1] and the W3 cell [3–5]. We then examine the performance of these models at detecting varying visual stimuli over a range of conditions, including noise and jitter, and discuss strategies by which outputs of different cell types can best be combined to track moving targets in a visual scene. We then compare the effectiveness of these strategies on “real-world” videos of visual scenes.

**References**

1. Baccus SA, Ölveczky BP, Manu M, Meister M. *J*. *Neuroscience* 2008 28: 6807–6817.
2. Barlow HB, Levick WR. J. Physiology 178: 477–504.
3. Kim T, Kerschensteiner D. *Cell Reports* 2017, 19: 1343–1350.
4. Kim T, Soto F, Kerschensteiner D. *eLife* 2015, 4: e08025.
5. Zhang Y, Kim I-J, Sanes JR, Meister M. *PNAS* 2012, 109: E2391-E2398
6. Hassenstein B, Reichardt WZ. *Naturforsch* 1965. 11b: 513–524.

## P48 Using information theory and a bayesian model to examine the factors that influence the decision to consume alcohol in a rodent model of alcoholism

### Nicholas Timme, David Linsenbardt, Christopher Lapish

#### Indiana University-Purdue University, Department of Psychology, Indianapolis, IN, United States

##### **Correspondence**: Nicholas Timme (nicholas.m.timme@gmail.com)

*BMC Neuroscience* 2018, **19(Suppl 2):**P48

About 16 million Americans have been diagnosed with an alcohol use disorder and alcohol use costs the United States approximately 250 billion dollars a year. Therefore, identifying the factors that lead to excessive drinking and understanding the neural mechanisms of how they do so is a vital goal in neuroscience. In this study, we used information theory and Bayesian modelling techniques to examine both neural and behavioral signals that predict alcohol consumption in rodents. We performed in vivo electrophysiological recordings in the dorsal medial prefrontal cortex (mPFC, a brain region heavily involved in decision-making) of a validated rodent model of excessive drinking (alcohol preferring (P) rat) and a control rat line (Wistar) during a simple cued alcohol drinking task. We used dynamic information theory (mutual information) to examine changes in encoding of future drinking (intent) at multiple time points throughout this task by individual neurons. We found that P rats showed decreased intent encoding compared to Wistars when consuming alcohol, but similar intent encoding when consuming water. These results indicate that encoding of alcohol drinking intent is diminished in the mPFC in animals with a genetic risk for excessive drinking (P rats). Next, we used behavioral data and Bayesian modelling techniques to construct a logistic regression model incorporating behavioral variables to predict when these rodents would drink. Model coefficients for number of previous drinking bouts and distance to sipper were significant for many recordings indicating predictive power in determining if the animal would drink on a given trial. The model was able to predict future drinking well. For instance, receiver operating characteristics area under the curve was above 0.8 for 22 of 26 individual animal recordings and for all animals combined. These results indicate that the logistic regression model developed herein is capable of predicting future drinking in this experimental paradigm. Overall, these results identify key behavioral variables that influence the decision to consume alcohol and provide evidence that the neural processes underlying this decision-making process are fundamentally altered in excessive drinking animals. In future studies, we will continue to combine these techniques to examine encoding of behavioral signals and latent variables relevant to the prediction of drinking in other brain regions to more fully understand the key changes in information processing underlying maladaptive decision-making in alcohol use disorder.

## P49 Stochastic facilitation of encoding process of a dynamical pattern in mouse retina

### Arthur Hung^1^, Chi Keung Chan^2^, Chuan-Chin Chiao^3^

#### ^1^National Tsing Hua University, Department of Physics, Hsinchu, Taiwan, Province of China; ^2^Academia Sinica, Department of Physics, Taipei, Taiwan, Province of China; ^3^National Tsing Hua University, Department of Life Sciences, Hsinchu, Taiwan, Province of China

##### **Correspondence**: Arthur Hung (arsene813@yahoo.com.tw)

*BMC Neuroscience* 2018, **19(Suppl 2):**P49

The task of the nervous system is to detect, compute, and make decisions in order to make responses that best suit the survival of the organism. Under evolutionary pressures, the organism evolves their nervous system to achieve high reliability and precision. Take the visual system for example: the in vitro experiment demonstrates that the retina can count single photons. However, the nervous system is also accompanied with various types of noises, such as synaptic conduction, thermal fluctuations of photosensitive molecules, as well as stochastic openings and closings of ion channels. Noise is usually regarded as a disturbance which lowers reliability and precision. However, there is a phenomenon in nonlinear physics known as “stochastic resonance (SR)” which states something entirely different: the presence of noise can enhance the detection of a weak sub-threshold signal. In this study, we studied the encoding process of a dynamic pattern under different contrast levels, and then examined if adding noise can enhance the information transfer when the contrast is low. We used in vitro electrophysiological recording and computer simulation to investigate how noise influences the encoding process of different light intensity patterns in the retina and tried to identify the relevant circuity components that would result in the enhancement. We generated our stimulus sequence by hidden markov model, and we used a gamma corrected LCD panel for light stimulation which focused on the photoreceptor layer of the retina by a microscope lens and calibrated by a separate digital microscope on the top with an amplified photodiode. The extracellular recording was conducted by using 64 channels (electrodes) MEA with the diameter of each electrode 10 mmor 30 mm, and 200mm apart ordered in a square fashion to measure the spiking pattern (action potentials) of the retinal ganglion cells. Time shifted mutual information analysis (Shannon information) was performed for different contrast conditions to quantify information transfer. We found that lower the contrast is, lower the peak height of time shifted mutual information would be, and this scaling was nonlinear. There were different patterns and shapes of this time shifted mutual information. Roughly, there were two kinds of the pattern: (1) single peak, and (2) double peak, and that lowering the contrast would not change the peak location in time and the shape of it, just the peak height. This result gives us insights about what the limitation of the encoding/detection process is. We are in the process of adding spatial uniform or non-uniform noise in the sub-threshold contrast level and test SR directly. As for the effect of noise, we performed a simulation using FitzHugh–Nagumo model and the input was a periodic sine wave, the results showed that adding noise can indeed enhance the phase lock ability of the cells.

## P50 Diverse dynamics in small recurrent networks: A case study of coupled recurrent and coupled inhibitory neurons

### Pei Hsien Liu^1^, Cheng-Te Wang^2^, Alexander White^3^, Tung-Chun Chang^4^, Chung-Chuan Lo^3^

#### ^1^National Tsing Hua University, Interdisciplinary Program of Engineering, Hsinchu City, Taiwan, Province of China; ^2^National Tsing Hua University, Institute of Bioinformatics and Structural Biology, Hsinchu, Taiwan, Province of China; ^3^National Tsing Hua University, Institute of Systems Neuroscience, Hsinchu, Taiwan, Province of China; ^4^Academia Sinica, Institute of Information Science, Taipei, Taiwan, Province of China

##### **Correspondence**: Pei Hsien Liu (belle.l24358@gmail.com)

*BMC Neuroscience* 2018, **19(Suppl 2):**P50

Amongst many studies of small circuits and brain network topologies, recurrently connected neurons are universally found in the brain of most species, including primates, rodents and insects. These recurrent circuits have been suggested to play multiple roles in brain functions. Indeed, from an evolutionary point of view, it is cost effective for the nervous systems to develop a “Swiss army knife” solution, in which a small set of neural circuit motifs are able to perform a variety of functions. However, the exact structure of these circuits as well as how they give rise to the diverse functions is still unclear. Some of the known functions include robustness, balancing of excitation and inhibition, decision making, oscillations and memory. In this project, we systematically studied the functions of a class of recurrently connected microcircuits, using a computational modeling approach. We first identified four-node motifs that are abundant in the current Drosophila connectome (around 22,835 neurons) in comparison to random networks. Two approaches are then employed to study the functionalities of the over-represented circuits: a dynamical and an information-theoretical one. For the dynamical approach, our analysis demonstrated that one of the most abundant motifs exhibits diverse functionalities, including working memory, decision making, flip-flop switching and oscillation. For the information-theoretic approach, we obtained a rudimentary set of metrics that partially reflects the system’s dynamics without information about the details of parameters, including the distribution of firing rate Fano factor and ISI Fano factor. This can serve as a reference for experimentalists who wish to understand emergent properties that arises from the interconnection of neurons but do not know the precise parameters or inputs. In summary, our research reveals the potential functions of a class of small recurrent circuits, and provide insights into the canonical architecture of the nervous systems.

## P51 Morpho-electric properties and computational simulation of human dentate gyrus granule cells from the epileptogenic hippocampus

### Anatoly Buchin^1^, Rebecca de Frates^1^, Peter Chong^1^, Rusty Mann^1^, Jim Berg^1^, Ueli Rutishauser^2^, Ryder Gwinn^3^, Staci Sorensen^1^, Jonathan Ting^1^, Costas A. Anastassiou^1^

#### ^1^Allen Institute for Brain Science, Modelling, Analysis and Theory, Seattle, WA, United States; ^2^Cedars-Sinai Medical Center, California Institute of Technology, Los Angeles, CA, United States; ^3^Swedish Medical Center, Seattle, WA, United States

##### **Correspondence**: Anatoly Buchin (anat.buchin@gmail.com)

*BMC Neuroscience* 2018, **19(Suppl 2):**P51

Epilepsy is the fourth most common neurological disease characterized by unpredictable seizures interrupting normal brain function. Despite considerable advances in the treatment and diagnosis of seizure disorders, about 40% of patients remain pharmacoresistant [1]. Seizures are often correlated with hippocampal sclerosis [2], which is classified by Watson Grade (WG) ranging from 0 to 5, from less to more severe case [3]. To elucidate mechanisms underlying epileptogenesis in human hippocampus we use an in vitro workflow to study the excitability of hippocampal neurons in tissue slices from specimens excised during brain surgery for the treatment of focal, pharmacoresistant epilepsy. We systematically analyzed the morphological and electrophysiological properties of human hippocampal dentate gyrus granule cells with different degree of hippocampal sclerosis (WG1 vs. WG4). We find that spiking properties such as f-I curve and spike-frequency adaptation are correlated with WG, while passive properties such as input resistance and the resting potential are not. The majority of morphological properties of single-neurons do not correlate with the degree of hippocampal sclerosis, further pointing to an excitability difference as the most prominent single-neuron biomarker. To test the implications of the observed differences under realistic scenarios we develop biophysically detailed computational models of granule cells with active dendrites that reproduce key electrophysiological features of human hippocampal granule cells as function of WG. Using these models we explored relevant scenarios associated with hippocampal sclerosis and the propensity towards seizure initiation.

**References**

1. Fisher R, Boas W, Blume W, et al. Epileptic Seizures and Epilepsy: Definitions Proposed by the International League Against Epilepsy (ILAE) and the International Bureau for Epilepsy (IBE).
2. Cendes F, Cook M, Watson C, et al. Frequency and characteristics of dual pathology in patients with lesional epilepsy.
3. Watson C, Nielsen SL, Cobb C, et al. Pathological grading system for hippocampal sclerosis: correlation with magnetic resonance imaging-based volume measurements of the hippocampus.

## P52 Development of realistic single-neuron models of mouse V1 capturing in vitro and in vivo properties

### Yina Wei, Anirban Nandi, Costas A. Anastassiou

#### Allen Institute for Brain Science, Modelling, Analysis and Theory, Seattle, WA, United States

##### **Correspondence**: Yina Wei (yinaw@alleninstitute.org)

*BMC Neuroscience* 2018, **19(Suppl 2):**P52

The Allen Institute for Brain Science has a mission to understand cortical computations related to the rodent visual pathway. Part of this approach is to develop accurate single-neuron models that capture basic observations across multiple spatiotemporal scales [1]. The Allen Cell Types Database reports an approach for generating single-neuron models from 3D morphologies and somatic electrophysiological recordings. While informative, these models are limited to observations at the soma whereas approx. 95% of neural surface area is along their dendrites. Improper characterization of these dendrites can lead to gross distortion of their synaptic integration capabilities given the main postsynaptic target is along dendritic cables (especially of excitatory synapses). To overcome these challenges, we develop a model generation workflow based on experimental data from two modalities:in vitro somatic intracellular recordings from slice experiments and in vivo extracellular recordings of single units from behaving rodent experiments. Especially regarding the latter data modality, a novel extracellular probe called Neuropixels offers the ability to measure extracellular action potential (EAP) signatures from multiple (up to 10) contacts in vivo [2]. We use these EAP signatures extending over hundreds of μm as a constraint for modeling various passive and active dendritic properties [3]. Specifically, we modify the fitness function of the genetic algorithm within our optimization framework to include extracellular features, such as the amplitude and the width of the backpropagating EAP, extracted from multi-channel recordings in freely moving animals alongside intracellular features at the soma. We evaluate the two models with regards to their goodness-of-fit against in vitro and in vivo data for excitatory and inhibitory cell classes and show how adding in vivo dendritic features to the optimization contributes to capturing key intracellular and extracellular observables. These results lay the groundwork towards a powerful modeling approach leveraging the rich data set at our disposal.

**References**

1. Hawrylycz M, Anastassiou C, Arkhipov A, Berg J, Buice M, Cain N, Gouwens NW, Gratiy S, Iyer R, Lee JH et al.: Inferring cortical function in the mouse visual system through large-scale systems neuroscience. *Proc Natl Acad Sci U S A* 2016, 113(27), 7337–7344.
2. Jun JJ, Steinmetz NA, Siegle JH, Denman DJ, Bauza M, Barbarits B, Lee AK, Anastassiou CA, Andrei A, Aydin C et al.: Fully integrated silicon probes for high-density recording of neural activity. *Nature* 2017, 551(7679), 232–236.
3. Gold C, Henze DA, Koch C: Using extracellular action potential recordings to constrain compartmental models. *J Comput Neurosci* 2007, 23(1), 39–58.

## P53 A multi-modal discovery platform toward studying mechanisms-of-action of electric brain stimulation

### Fahimeh Baftizadeh^1^, Soo Yeun Lee^1^, Sergey Gratiy^1^, Taylor Cunnington^2^, Shawn Olsen^1^, Costas A. Anastassiou^1^

#### ^1^Allen Institute for Brain Science, Modelling, Analysis and Theory, Seattle, WA, United States; ^2^University of Washington, Seattle, WA, United States

##### **Correspondence**: Fahimeh Baftizadeh (fahimehb@alleninstitute.org)

*BMC Neuroscience* 2018, **19(Suppl 2):**P53

There has been increased interest and progress in the field of neural prosthetics with many exciting efforts focusing on the development of brain-machine interfaces to drive sensory devices (e.g. retina, cochlea) or motor devices (e.g. prosthetic limbs). Yet, with a few notable exceptions, employment of neuro-prosthetics for monitoring and intervention in cognitive physiology and pathologies has remained limited. Concurrently, cognitive impairment has proven to be among the least tractable and most disabling in a wide variety of brain disorders including autism, epilepsy, depression and schizophrenia. Despite the intense interest and potential regarding the use of electrical stimulation in cognitive disorders, there is still today a debilitating lack of understanding about where, when and how to inject current into cortical circuits to modulate higher-level brain processing. At the same time, the Allen Institute for Brain Science has developed a large-scale approach for the robust and reproducible deconstruction of cortical circuitry toward understanding how the interplay of components gives rise to high-level processing [1]. We use a similar approach to address the challenges linked to understanding and predicting brain stimulation effects with the primary goal being to tackle the fundamental question of how to inject current to transform the specificity and capability of electrical stimulation devices either in open- or closed-loop mode to ameliorate cognitive disorders. Specifically, using a combination of biophysically detailed simulation workflow (allowing the exploration and permutation of key parameters in electrical stimulation entrainment) in parallel with novel, multi-patch brain slice experiments we seek to understand electric field effects at the single-neuron level and how different parameters such as the distance from the electrode or stimulation characteristics impact sub-threshold and spiking responses of single neurons. We use this platform to generate novel insights toward significantly refining brain stimulation techniques in therapeutic neuroscience research.

**Reference**

1. Koch C, Reid RC. Observation of the mind, *Nature* 2012, 7, 1–14.

## P54 Kernel current source density revisited

### Chaitanya Chintaluri^1^, Marta Kowalska^2^, Michał Czerwiński^2^, Władysław Średniawa^2^, Joanna Jędrzejewska-Szmek^2^, Daniel Wójcik^2^

#### ^1^University of Oxford, Centre for Neural Circuits and Behaviour, Oxford, United Kingdom; ^2^Nencki Institute of Experimental Biology of PAS, Laboratory of Neuroinformatics, Warsaw, Poland

##### **Correspondence**: Daniel Wójcik (d.wojcik@nencki.gov.pl)

*BMC Neuroscience* 2018, **19(Suppl 2):**P54

Extracellular potential in the brain reflects the activity of transmembrane currents of neural and glial cells. The long range of the electric field leads to significant correlations between recordings at distant sites, complicating the analysis. Reconstructing the Current Source Density (CSD) which is the local origin of the potential facilitates data interpretation. In 2012 we introduced Kernel Current Source Density method (KCSD), a model-based reconstruction method, which allows source estimation from arbitrary distribution of electrodes. The method is also guarded against over-fitting by constraining complexity of the inferred CSD model. Here we revisit the method on the occasion of a new open Python implementation which includes new functionality and several additional diagnostic tools as compared to the original. The goal of this presentation is to advertise the method, the new implementation, and the new diagnostics available. Specifically we (1) analyze spectral properties of the method; (2) introduce error maps to investigate accuracy of the reconstruction; (3) introduce L-curve for estimation of optimal reconstruction parameters. The new implementation allows to perform reconstruction for 1D, 2D, and 3D setups, assuming sources distributed in the whole tissue, inside a slice, or on single cells when the cell morphology is available and the potential comes from that cell. The toolbox accompanied by a tutorial Jupyter notebook is available at https://github.com/Neuroinflab/kCSD-python

## P55 Modelling the electrical impedance of neural tissue based on its cellular building blocks

### Anthony Burkitt^1^, David Grayden^1^, Hamish Meffin^2^, Omid Monfared^1^, Bahman Tahayori^1^, Dean Freestone^3^, Dragan Nesic^4^

#### ^1^University of Melbourne, Department of Biomedical Engineering, Parkville, Australia; ^2^National Vision Research Institute, Carlton, Australia; ^3^University of Melbourne, Department of Medicine, Parkville, Australia; ^4^University of Melbourne, Department of Electrical & Electronic Engineering, Parkville, Australia

##### **Correspondence**: Hamish Meffin (hmeffin@yahoo.com)

*BMC Neuroscience* 2018, **19(Suppl 2):**P55

Knowledge of electrical properties of neural tissue, such as conductivity, is important in various applications such as therapeutic electrical stimulation of the nervous system and electrical impedance tomography. It is also essential for the interpretation of intrinsic electrical signals in neuroscience such as single and multi-unit activity, the local field potential and electroencephalogram. It is usually assumed that neural tissue can be described by a locally homogeneous conductivity that captures the bulk properties of heterogeneous cellular microstructure. However, the cellular structure of tissue creates a complex partition of intra- and extra-cellular spaces that are separated by a high impedance membrane. These microstructural inhomogeneities lead to complicated current paths through the tissue, invalidating assumptions that allow a description based on a simple conductivity.

Here, we review our recent work that begins with the underlying heterogeneous microstructure of neural tissue and derives its bulk electrical properties in the form of the tissue admittivity, which generalized the usual conductivity [1–4]. A novel aspect of the admittivity is that it has both spatial and temporal spectral frequency dependence. New expressions are given for the admittivity of several tissue types including isotropic tissues with fibers oriented randomly in all (three-dimensional) directions and laminar tissues types with fibers oriented randomly within planes that are stacked upon each other. The spatio-temporal spectral frequency dependence of the tissue admittivity leads to non-trivial spatiotemporal electrical filtering properties of neural tissue, which we illustrate here. First, we show how a variation in a temporal parameter, namely applied pulse-width, can affect a spatial property like the profile of the extracellular potential. Second, we showed that, for tissue with a homogeneous structural anisotropy, variation in a spatial variable, namely distance from the electrode, can nonetheless affect the degree of electrical anisotropy.

**Acknowledgements**

OM acknowledge support from a NICTA/Data61 postgraduate research scholarship. HM acknowledge support from the Australian Research Council Centre of Excellence for Integrative Brain function.

**References**

1. Meffin H, Tahayori B, Grayden DB, Burkitt AN. Modeling extracellular electrical stimulation: I. Derivation and interpretation of neurite equations, *J*. *Neural*. *Eng*. 2012, 9(6), Art# 060505.
2. Tahayori B, Meffin H, Dokos D, et al. Modeling extracellular electrical stimulation: II. Computational validation and numerical results, *J*. *Neural*. *Eng*. 2012, 9(6), Art# 060506.
3. Meffin H, Tahayori B, Sergeev EN, et al. Modelling extracellular electrical stimulation III: Derivation and interpretation of neural tissue equations, *J*. *Neural Eng*, 2014, 11(6), Art# 065004.
4. Tahayori B, Meffin H, Sergeev EN, et al. Modelling extracellular electrical stimulation IV : Effect of the cellular composition of neural tissue on its spatio-temporal filtering properties, *J*. *Neural Eng*. 2014, 11(6), Art# 065005.

## P56 Local and global neuronal network structure influence synchronous events

### Brittany Baker, Duane Nykamp

#### Univeristy of Minnesota, School of Mathematics, Minneapolis, MN, United States

##### **Correspondence**: Brittany Baker (bake0573@umn.edu)

*BMC Neuroscience* 2018, **19(Suppl 2):**P56

We have developed a network model where one can independently modulate both local and global features of the network connectivity. Our application of local microstructures is based on the SONET model [1], where one can specify the frequencies of different two-edge motifs in the network. We have extended this approach to allow for the inclusion of global structure in the patterns of connections, such as connections based on an underlying geometry. Using this model, we investigated how the influence of microstructure (motifs) on the emergence of synchronous events is modulated by spatial features of the network.

**Reference**

1. Zhao L, Beverlin II B, Netoff T, Nykamp DQ. Synchronization from second order network connectivity statistics. *Frontiers in Computational Neuroscience*, 5(28), 2011. 10.3389/fncom.2011.00028.

## P57 Interplay of synaptic noise and chaos determines limits of cortical reliability

### Max Nolte, Michael Reimann, James King, Henry Markram, Eilif Muller

#### École Polytechnique Fédérale de Lausanne, Blue Brain Project, Lausanne, Switzerland

##### **Correspondence**: Max Nolte (max.nolte@epfl.ch)

*BMC Neuroscience* 2018, **19(Suppl 2):**P57

The combined impact of cellular noise sources and network dynamics on the intrinsic variability of cortical activity is not known. We quantified this variability by analyzing how somatic membrane potentials in simulations of neocortical microcircuitry with biological noise sources diverged from identical initial conditions. By selectively disabling noise sources, we found that any combination of noise or subthreshold perturbations causes chaotic divergence of membrane potentials with similarly high steady-state variability. However, the rate at which membrane potentials diverged depended on which noise sources were active, with synaptic noise dominating the rate. We found that, in spite of this high intrinsic variability, thalamocortical inputs can overcome chaotic network dynamics to produce reliable spike timing. However, synaptic noise causes a substantial residual spike-timing variability, and the rate by which this evoked activity diverges is similar to spontaneous activity. Thus, any mechanism of reliable cortical coding must be robust to the limits set by the interplay of synaptic noise and chaos.

## P58 Shedding light on the cellular origins of voltage-sensitive dye imaging: an in silico study

### Taylor Newton, Juan Hernando, Jafet Villafranca Díaz, Stefan Eilemann, Grigori Chevtchenko, Henry Markram, Eilif Muller

#### École Polytechnique Fédérale de Lausanne, Blue Brain Project, Lausanne, Switzerland

##### **Correspondence**: Taylor Newton (taylor.newton@epfl.ch)

*BMC Neuroscience* 2018, **19(Suppl 2):**P58

We report on an in silico implementation of voltage-sensitive dye imaging (VSDI) for validating and exploring mesoscopic neural activity in a biologically detailed digital reconstruction of rat somatosensory cortex. This model comprises a network of 31,346 morphologically detailed neurons arranged in a 462 × 400 × 2082 μm columnar microcircuit. We evaluated the behavior of our in silico VSDI model against several findings reported in literature, including the relative contributions of different cell morphologies and cortical layers to the VSD signal, the fraction of the VSD signal due to spiking versus subthreshold activity, and the phase velocity of a stimulus driven VSDI wavefront. Using simulated VSDI measurements, we confirm that our reconstructed microcircuit exhibits stimulus-evoked response dynamics that are qualitatively similar to experimental findings. Furthermore, we show that contrary to widely held assumptions, dendritic projections within L2/3 but emanating from L5 pyramidal cells contribute significantly to the VSD signal (Fig. 1). In addition, we observe a morphology-dependent low-pass filtering effect in L5 contributions to the VSD signal, due to the increased dendritic path length between L5 somas and their L2/3 apical projections. Last, we find that individual spikes do not make measurable contributions to the VSD signal except during periods of highly synchronous activity, during which back propagating action potentials, and not somatic spikes, dominate spike-related contribution.

**Fig. 1:**
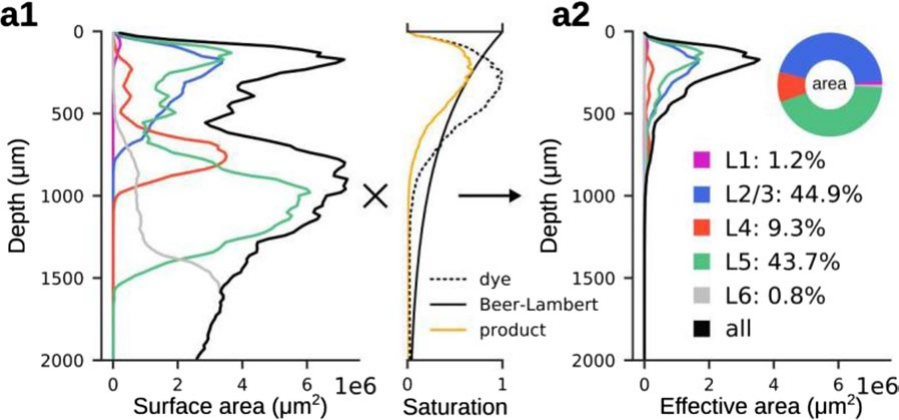
Neurites within layer 2/3, belonging to cells in layers 2/3 and 5, drive the VSD signal. (a) Surface area contributions for each layer by depth. a1 Raw (unscaled) surface areas by depth; a2 Effective (scaled) surface area by depth

## P59 Reconstruction and simulation of a full-scale model of rat hippocampus CA1

### Michele Migliore^1^, Lida Kanari^2^, James King^2^, Szabolcs Kali^3^, Henry Markram^2^, Armando Romani^2^, Nicolas Antille^2^, Luca Leonardo Bologna^5^, Julian Martin Leslie Budd^5^, Jean-Denis Courcol^2^, Adrien Devresse^2^, Andras Ecker^2^, Joanne Falck^6^, Cyrille PH Favreau^2^, Michael Gevaert^2^, Attila Gulyas^5^, Olivier Hagens^2^, Juan Hernando^2^, Silvia Jimenez^2^, Sigrun Lange^7^, Carmen Alina Lupascu^1^, Rosanna Migliore^1^, Maurizio Pezzoli^2^, Srikanth Ramaswamy^2^, Christian A Rössert^2^, Sara Sáray^5^, Ying Shi^2^, Werner Alfons Hilda Van Geit^2^, Liesbeth Vanherpe^2^, Tamas Freund^5^, Audrey Mercer^7^, Alex M Thomson^7^, Eilif Muller^2^

#### ^1^Institute of Biophysics, National Research Council, Palermo, Italy; ^2^École Polytechnique Fédérale de Lausanne, Blue Brain Project, Lausanne, Switzerland; ^3^Institute of Experimental Medicine, Hungarian Academy of Sciences, Budapest, Hungary; ^4^University College London & Deutsches Zentrum für Neurodegenerative Erkrankungen, Germany; ^5^University College London, United Kingdom

##### **Correspondence**: Michele Migliore (michele.migliore@cnr.it)

*BMC Neuroscience* 2018, **19(Suppl 2):**P59

We present a full-scale cellular level model of the CA1 area of the hippocampus of a rat. The model is built using a bottom-up data-driven workflow, along the same lines followed to implement a cortical column [1]. Starting from a set of reconstructed morphologies for primary morphologically defined cell types, associated electrophysiological traces, and data-driven channel kinetics, we implemented biophysically accurate neurons models consistent with statistics of features extracted from the experimental traces. A virtual volume [2] was populated according to densities and proportions determined in [3]. The neurons we connected according to the approach previous developed for the neocortex [4], and the resulting connectivity and synaptic properties were validated against a number of experimental findings. The current release is composed by 42 types of neuron (24 excitatory and 18 inhibitory) divided into 13 morphological types, 17 morpho-electrical types, 156 potential pathways, and 7 intrinsic synapse types. Simulations of the network show interesting emergent properties, such as theta oscillations in a LFP-like signal. The oscillations emerge from the intrinsic connectivity of the CA1 circuit driven by the spontaneous miniature events without any external input, as observed experimentally [5]. Furthermore, the network activity propagates along the septo-temporal axis, consistently with what has been observed experimentally [6]. Phenomena like oscillations and traveling waves in the theta rhythm range can play important roles in shaping the hippocampus function, but their mechanisms are not completely understood. The full-scale CA1 model represents an important tool to shed light on the cellular mechanisms behind such phenomena, elucidate the physiological conditions in which they can occur, and eventually reveal their role in the brain.

**References**

1. Markram H, et al., 2015, Cell 163:456–92.
2. Ropireddy D, et al., 2012, Neurosci. 205:91–111.
3. Bezaire MJ, et al., 2016, Elife Dec 23;5.
4. Reimann MW, et al., 2015, Front Comput Neurosci.9:120
5. Goutagny R, et al., 2009, Nat Neurosci. 12:1491–3.
6. Lubenov EV, Siapas AG. 2009, Nature 459:534–9.

## P60 The SONATA data format: A new file format for efficient description of large-scale neural network models

### Kael Dai^1^, Yazan Billeh^1^, Jean-Denis Courcol^2^, Sergey Gratiy^1^, Juan Hernando^2^, Adrien Devresse^2^, Michael Gevaert^2^, James King^2^, Werner Alfons Hilda Van Geit^2^, Daniel Nachbauer^2^, Arseny Povolotskiy^2^, Anton Arkhipov^1^, Eilif Muller^2^

#### ^1^Allen Institute for Brain Science, Modelling, Analysis and Theory, Seattle, WA, United States; ^2^École Polytechnique Fédérale de Lausanne, Blue Brain Project, Lausanne, Switzerland

##### **Correspondence**: Kael Dai (kaeld@alleninstitute.org)

*BMC Neuroscience* 2018, **19(Suppl 2):**P60

Increasing computing power and availability of high-performance computing (HPC) resources have made it easier for neuroscientists to simulate and visualize large-scale brain network models.

However, one bottleneck for scientists developing, researching and sharing large-scale networks is the lack of efficient data formats to describe such models. A widespread practice is to represent models with simulator specific code such as hoc, SLI or python. XML based formatshave been proposed as a solution.But the use of XML quickly becomes problematic when scaling up to large realistic networks. Thus, an open specification is needed that is compact, computationally fast, yet also easy to read and edit. To meet these demands, the Allen Institute (AI) and the Blue Brain Project (BBP) have jointly developed the SONATA (Scalable Open Network Architecture TemplAte) Data Format—an open-source framework for representing neuronal circuits. The framework utilizes both organizations’ expertise with large-scale HPC network simulation, visualization and analysis. It was designed for memory and computational efficiency, as well as to work across multiple platforms. Even though AI and BBP use different approaches to modeling and use different tools, the format allows networks built by one institute to be simulated by the other and vice versa. We provide the specification documentation, open-source reference APIs, and model and simulation output examples with the intention of catalyzing support and adoption of the format in the modeling community. The specification describes a format for representing nodes (cells) and edges (synapses/junctions) of a network. It uses table-based data structures, hdf5 and csv, to represent nodes, edges and their respective properties. Furthermore indexing procedures for fast and parallelizable lookup of individual nodes and edges. The use of hdf5 provides both efficiency in space and read-time. The format includes specifics properties and naming conventions, but also allows modelers to extend node and edge model properties as they desire, to ensure models can be used with a variety of simulation frameworks and use cases. Besides network representation, saving the output of large-scale network simulations presents formidable challenges. The output format must not only be standardized for reproducibility and analysis across teams, but also optimized for memory and read/write performance. The data format architecture we present here offers solutions to both problems. A systematic schema for describing simulation reports makes it easy for users to exchange their data, and moreover the underlying hdf5 based format permits efficient storage of variables like spike times, membrane potential, and Ca2+ concentration. Lastly, to bring together network models, simulation output, and various run-time conditions (duration, time step, temperature, etc.), the specification includes aJSON-based fileformat for configuring simulations, including specifying variables to record from, and stimuli to apply. This will help reduce the guesswork normally needed to reproduce and adjust other organization’s simulations. The rapid advancement in neuroscientific data generation, large-scale data-driven modeling, and simulation capabilities makes the development of standards for network simulations necessary. The SONATA Data Format and framework are open to the community to use and build upon with the goal of achieving such a standard data format.

## P61 Stability of synaptic weights in a biophysical model of plasticity in the neocortical microcircuit without explicit homeostatic mechanisms

### Michael Reimann, Giuseppe Chindemi, Henry Markram, Eilif Muller

#### École Polytechnique Fédérale de Lausanne, Blue Brain Project, Lausanne, Switzerland

##### **Correspondence**: Michael Reimann (michael.reimann@epfl.ch)

*BMC Neuroscience* 2018, **19(Suppl 2):**P61

Spike-timing dependent synaptic plasticity has been characterized on a pairwise level in vitro. However, many of the identified forms of plasticity are inherently unstable in recurrent networks. For example, for hebbian-style plasticity the strengthening of a connection increases the likelihood that it will be strengthened further, leading to runaway potentiation. Homeostatic mechanisms have been proposed to stabilize the system, but physiological evidence for them remains indirect and inconclusive. For a morphologically detailed model of a cortical microcircuit in conjunction with a biologically constrained, calcium-based model of plasticity we characterized the stability of plastic connectivity in a population of neurons in the absence of an explicitly homeostatic mechanism. We explored the evolution of the strengths of 24 million recurrent glutamatergic synapses and their stability under in vivo-like conditions with simulated external input. We found that while individual synapse weights evolved significantly, there was a remarkable degree of stability in terms of average synaptic strength both on the single cell and population level. We then further characterized how the observed shift of synaptic strength between individual synapses affected the response properties of neurons, such as their average firing rates or their selectivity for individual stimuli and observed an increase in both for neurons in cortical layer 5.

## P62 Biophysical modeling of synaptic plasticity in the somatosensory cortex

### Giuseppe Chindemi^1^, James King^1^, Srikanth Ramaswamy^1^, Michael Reimann^1^, Christian A Rössert^1^, Werner Alfons Hilda Van Geit^1^, Henry Markram^1^, Vincent Delattre^1^, Adrien Devresse^1^, Michael Doron^2^, Jeremy Fouriaux^1^, Michael Graupner^3,^ Pramod Kumbhar^1^, Max Nolte^1^, Rodrigo Perin^3^, Fabien Delalondre^1^, Idan Segev^2^, Eilif Muller^1^

#### ^1^École Polytechnique Fédérale de Lausanne, Blue Brain Project, Lausanne, Switzerland; ^2^Hebrew University of Jerusalem, Department of Neurobiology, Jerusalem, Israel; ^3^Université Paris Descartes, Laboratoire de Physiologie Cérébrale—UMR 8118, CNRS, Paris, France; ^4^École Polytechnique Fédérale de Lausanne, Laboratory of Neural Microcircuitry, Lausanne, Switzerland

##### **Correspondence**: Giuseppe Chindemi (giuseppe.chindemi@epfl.ch)

*BMC Neuroscience* 2018, **19(Suppl 2):**P62

Synaptic connections in the brain form a highly dynamical map, constantly adapting to external stimuli and internal dynamics: new connections can be formed, while existing ones can be modified or eliminated throughout the entire life of the organism. This adaptability of synaptic connections is referred to as “synaptic plasticity” and it is thought to be the foundation of learning and memory. Despite all the interest of the scientific community, experimental and theoretical work on synaptic plasticity is highly fragmented: only a few connection-types have been characterized experimentally, i.e. those between layer 5 thick-tufted pyramidal cells in the neocortex, and no model so far has been able to reconcile this sparse body of data. In this work, we integrated state of the art data and theories on synaptic plasticity to design a unifying model of a plastic glutamatergic synapse in the neocortex. In particular, we extended a previous calcium-based model of spike-timing dependent plasticity (STDP) [1] to account for more detailed synaptic dynamics: stochastic vesicle release, accurate NMDAR- and VDCC-mediated calcium currents, postsynaptic calcium accumulation and clearance, and timescales of plasticity expression. Parameters of the model were then constrained to reproduce in vitro STDP data from layer 5 thick-tufted pyramidal cells in the somatosensory cortex [2]. The optimized parameters were than applied to all other excitatory connections in the same brain area, with the sole exception of the potentiated over depressed synapse ratio, re-calculated for each connection-type to match the expected mean release probability [3]. We successfully validated our generalization approach against independent plasticity data on layer 2/3 to layer 5 pyramidal connections [4], layer 2/3 to layer 2/3 pyramidal connections and layer 4 to layer 4 spiny stellate connections [5]. Our results show that the biophysics of synaptic transmission and the spatial extent of neuronal morphologies play a crucial role for synaptic plasticity, due to their influence on the magnitude and time course of postsynaptic calcium transients. Furthermore, we demonstrated how a few data points are required to parametrize a large and heterogeneous set of connections, hinting that only a small set of targeted in vitro experiments could be necessary to completely characterize the features of synaptic plasticity in the brain.

**References**

1. Graupner M, Brunel N. Calcium-Based Plasticity Model Explains Sensitivity of Synaptic Changes to Spike Pattern, Rate, and Dendritic Location. *Proceedings of the National Academy of Sciences* 2012, 109 (10): 3991–96.
2. Markram H, Lübke J, Frotscher M, Sakmann B. Regulation of Synaptic Efficacy by Coincidence of Postsynaptic APs and EPSPs. *Science* 1997, 275 (5297): 213–15.
3. Markram H, Muller E, Ramaswamy S, et al. Reconstruction and Simulation of Neocortical Microcircuitry. *Cell* 2015, 163 (2): 456–92.
4. Per Jesper S, Häusser M. A Cooperative Switch Determines the Sign of Synaptic Plasticity in Distal Dendrites of Neocortical Pyramidal Neurons. *Neuron* 2006, 51 (2): 227–38.
5. Egger V, Feldmeyer D, Sakmann B. Coincidence Detection and Changes of Synaptic Efficacy in Spiny Stellate Neurons in Rat Barrel Cortex. *Nature Neuroscience* 1999, 2 (12): 1098.

## P63 Dynamic Worm: Moving model of Caenorhabditis elegans worm controlled by the nervous system

### Jimin Kim, Eli Shlizerman

#### University of Washington, Electrical Engineering & Applied Mathematics, Seattle, WA, United States

##### **Correspondence**: Jimin Kim (jk55@u.washington.edu)

*BMC Neuroscience* 2018, **19(Suppl 2):**P63

Neural circuits generate body movements using rhythmic signals, however, little is known about characteristics of these signals, and their transformation to behavior. Here, we implement the dynamic worm, an in silico model for the nematode Caenorhabditis elegans, to understand how the nematode’s nervous system transforms stimuli to behavior. The model emulates the response of the nervous system to stimuli, translates neural activity to muscle forces and muscle impulses to body movements, and implements feedback to account for environmental entrainment. We validate the dynamic worm in both signal propagation directions; back propagating external force applied to the body, and investigate touch response behaviors by injection of current into sensory and inter neurons. We are able to generate locomotion behaviors typical to the nematode from external force waves and identify a set of stimuli that generate coherent locomotion. In particular, we show that only precise combinations of both sensory and inter neurons generate coherent movements, such as crawling forward, backward and turns. The characteristics of the movements resemble experimentally identified locomotion patterns. We show that neural dynamics associated with distinct movements can be mapped and classified using low dimensional space, but even more importantly, we show that the transformations between the layers of the model are dynamic and require full simulation for each stimulus. By exploring the effect of the environment on locomotion, we find that specific environmental parameters facilitate typical locomotion behavior, and environmental sensory feedback can entrain, sustain and switch between movements. Taken together, our results show that the nervous system encompasses mechanisms of movement initiation, activated by constant stimulation, and mechanisms of movement sustainment through entrainment by the environment (Fig. 1).

**Fig. 1:**
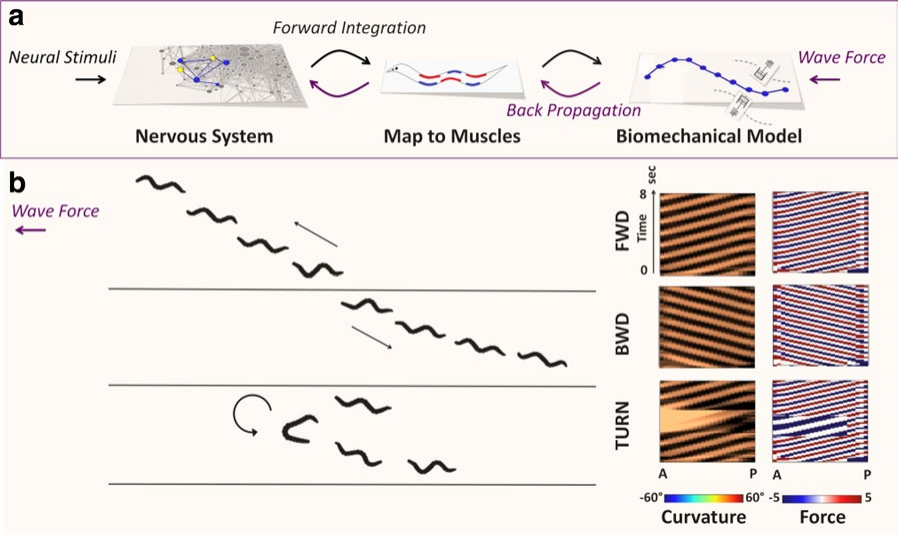
A: Layers in modeling C. elegans neuromuscular activity. Left to right: Layer 1: Modeling the nervous systems as a dynamical system encompassing the full somatic connectome including connectivity neural dynamics. Layer 2: Mapping neural dynamics to dynamic muscle impulses (forces). Layer 3: Muscle impulses are mapped to a biomechanical body model that incorporates body responses and interaction with the environment. Neural stimuli are integrated forward to resolve body movements. External forces are propagated backward to resolve corresponding neural dynamics. B: Typical locomotion patterns generated by three types of external wave forces, corresponding to forward (top), backward (middle), 180° turn (bottom) movements. The external force is propagated backward to resolve neural dynamics, which are integrated forward to produces movements. Locomotion patterns are characterized using body snapshots, sampled every 2 s, (left), curvature (middle) and muscle force (right)

## P64 Modeling network connectivity for dopamine-mediated olfactory learning in mosquitos

### Suh Woo Jung^1^, Jeffrey Riffell^2^, Eli Shlizerman^1^

#### ^1^University of Washington, Department of Electrical Engineering, Seattle, WA, United States; ^2^University of Washington, Department of Biology, Seattle, WA, United States

##### **Correspondence**: Suh Woo Jung (sjung94@uw.edu)

*BMC Neuroscience* 2018, **19(Suppl 2):**P64

Although it is widely known that dopamine (DA) neurons play critical roles in associative learning, the mapping of neurons and their effect on learning still remains unclear. In olfactory learning, it has been shown that superfusion of dopamine on mosquito brain strongly modulates activities of antennal lobe (AL) neurons. We therefore study neural population coding of mosquitoes AL projection neurons subject to dopamine modulation.

Projection neurons encode different stimuli via population fixed points in the response of Projection Neurons (PNs). Methods of dimension reduction, such as Optimal Exclusive Threshold Reduction (OETR), have been shown to be effective in inference of low dimensional odor space which spans fixed points associated with monomolecular stimuli. Furthermore, the odor space assists in recognition and classification of PN responses to mixtures of these stimuli. In our work, we apply the OETR method to multi-neuron recordings from mosquitoes PNs in three phases: before (S1), during (Dop), and after (S2) superfusion of dopamine over the brain of mosquitoes. We first test which stimuli produce separable fixed points, and once we found these, we construct a low dimensional odor space to map the fixed points and transients reaching them. We then examine the representation of the fixed points in different phases. The trajectories of the fixed points in three different phases (S1, Dop, and S2) reveal that (i) superfusion of dopamine has longer term effects on the AL encoding space, and that (ii) the degree of modulation differs from one odor to another. Particularly, neural responses to benzaldehyde show significant fixed point relocations in both Dop and S2 phases, while the fixed points associated with ammonia almost do not change their location in all three phases (see Fig. 1A). To investigate how dopamine modulation could rearrange neural connectivity in the AL, we employ a network model which infers the connectome of neurons in the AL from neural data and the odor space. In particular, the model is obtained through convex optimization and provides connectivity matrices of interneurons (LNs) and PNs which interact through lateral inhibition. We solve the convex optimization problem in three different phases of dopamine superfusion (S1, Dop, and S2) and find the connectomes associated with each phase. Connectome analysis indicates that the distribution of connectivity matrices is being modified to accommodate the observed relocation of fixed points in the odor space (see Fig. 1B). We therefore ask what are the changes the lateral inhibition from LN to PN is undergoing by fixing the connectivity within the LNs for all phases. We find that the distribution of lateral inhibition connections narrows in transition from S1 to Dop and narrows further in transition from Dop to S2. Taken together our results provide a first structural description of neural encoding modulation and possible anatomical changes associated with dopamine-mediated olfactory learning. These results can be used to explore the connectivity patterns of dopamine neurons within the AL and their functionality with respect to learning.

**Fig. 1:**
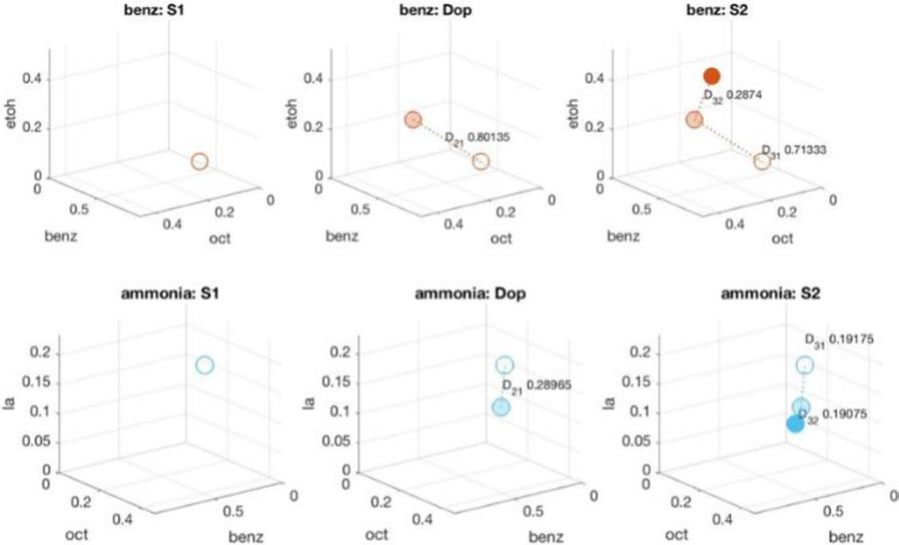
Fixed points of benzaldehyde and ammonia in three phases, S1, Dop, and S2. D_ij indicates the euclidean distance from phase j to i in the odor space. Figure B. Distribution of lateral inhibition matrix, B, from LN to PN in S1, Dop, and S2 phase respectively

## P65 Detection of spatio-temporal spike patterns in motor cortex during a reach-to-grasp task

### Pietro Quaglio^1^, Sonja Gruen^1^, Alper Yegenoglu^2^, Emiliano Torre^3^

#### ^1^Jülich Research Centre, Institute of Neuroscience and Medicine (INM-6), Jülich, Germany; ^2^Jülich Research Centre, Institute of Neuroscience and Medicine (INM-6) & Institute for Advanced Simulation (IAS-6), Jülich, Germany; ^3^ETH Zürich, Chair of Risk, Safety and Uncertainty Quantification, Zürich, Switzerland

##### **Correspondence**: Pietro Quaglio (p.quaglio@fz-juelich.de)

*BMC Neuroscience* 2018, **19(Suppl 2):**P65

In 1949 Hebb [1] proposed cell assemblies, i.e. groups of interacting neurons, as building blocks of information processing in the cortex. A signature of an active cell assembly in parallel spike recordings are synchronous or spatio-temporal spike patterns (STPs) [2, 3]. Modern electrophysiological techniques enable the simultaneous recording of hundred(s) of neurons and thereby increase the chances to observe active cell assemblies.

In two recent publications we developed a method, called SPADE, to detect statistically significant spike patterns in massively parallel spike data (MPST), where 100 or more parallel spike trains are available. The method was first limited to synchronous spikes [4], and then extended to spatio-temporal patterns [6]. The method reduces the computational costs for extraction of all possible repeated spike patterns by employing frequent itemset mining. To avoid a massive multiple testing problem it reduces the number of pattern candidates by pooling patterns with the same number of neurons and number of occurrences. SPADE then evaluates the statistical significance of the found patterns using a non-parametric Monte-Carlo sampling under the null hypothesis of independence. Finally, significant patterns are tested for conditional significance against each other. In [5] we applied SPADE to search for repeated synchronous patterns in MPST from electrophysiological data recorded from motor and premotor cortex of macaque monkeys. The monkeys performed a delayed reach-to-grasp task, where they had after a preparatory period to pull and hold an object using a side or precision grip and with high or low force. The recorded data were analyzed for the occurrence of significant synchrony in different behavioral epochs. We found a variety of significant synchronous patterns with high specificity to behavior. Here we present the challenges that such data pose when aiming to detect significant STPs and how this can be addressed by deploying SPADE. In particular we extend the statistical evaluation to test separately patterns of different temporal length, because otherwise the statistic presents a bias in favor of shorter patterns. By doing so, we now complement the previous results with the information provided by STPs. We analyze pattern compositions in terms of involved neurons and temporal arrangements in relation to behavior, confirming the expectation that extending the search to STPs increases the chance to detect patterns involving a larger number of neurons. In conclusion we show that the majority of the found spatio-temporal patterns is temporally locked to the movement onset and exhibit different neuronal composition for different grip modalities (precise grip or side grip).

**References**

1. Hebb (1949) *The organization of behavior*. Wiley & Sons
2. Singer W, Engel AK, Kreiter AK, et al. Neuronal assemblies: necessity, signature and detectability. *Trends in Cognitive Sciences* 1997, 1, 252–261
3. Harris KD. Neural signatures of cell assembly organization. *Nature Reviews Neuroscience* 2005, 5, 339–407
4. Torre E, Picado-Muino D, Denker M, et al. Statistical evaluation of synchronous spike patterns extracted by frequent item set mining. *Frontiers in Computational Neuroscience* 2013, 7:132
5. Torre E, Quaglio P, Denker M, et al. Synchronous Spike Patterns in Macaque Motor Cortex during an Instructed-Delay Reach-to-Grasp Task. *Journal of Neuroscience* 2016, 36(32), 8329–8340
6. Quaglio P, Yegenoglu A, Torre E, et al. Detection and Evaluation of Spatio-Temporal Spike Patterns in Massively Parallel Spike Train Data with SPADE. *Frontiers in Computational Neuroscience* 2017, 11

## P66 Random contrastive Hebbian learning as a biologically plausible learning scheme

### Georgios Detorakis^1^, Travis Bartley^2^, Emre Neftci^1^

#### ^1^University of California, Irvine, Department of Cognitive Sciences, Irvine, CA, United States; ^2^University of California, Irvine, Department of Electrical Engineering & Computer Science, Irvine, CA, United States

##### **Correspondence**: Georgios Detorakis (gdetor@protonmail.ch)

*BMC Neuroscience* 2018, **19(Suppl 2):**P66

Many computational models and widely used learning algorithms, such as back-propagation [1] (BP), require a form of bidirectional synaptic weights. Xie and Seung [2] have shown that BP, under some circumstances, can be equivalent to the Contrastive Hebbian Learning (CHL) algorithm. CHL has been proposed to explain biological phenomena such as hippocampal replay, where neural activity is transmitted back-and-forth between the hippocampus and the prefrontal cortex. CHL uses the transpose of the synaptic matrix to form a reverse connection between layers to account for weight changes in the forward connection. However, there is no evidence that cerebral areas talk to each other in a direct bidirectional way (weight transport problem). This means that using neural networks with symmetric synaptic weights is not biologically plausible. In this work, we propose an alternative mechanism that enables CHL without the use of symmetric weights for feedback transmission. The proposed mechanism is not solely based on synaptic plasticity but exploits the dynamics of neurons in combination with a Hebbian learning rule. We combine a recently proposed random back-propagation algorithm [3] with CHL. As with CHL, the neural network is trained in two phases, but in the reconstruction phase, feedback to previous layers is done using fixed random matrices. The proposed learning scheme uses continuous non-linear ordinary differential equations to describe the neural dynamics of the model. The layers of the feed-forward and the feedback subnetworks are treated as coupled neural systems, meaning that the information can be transmitted in a synchronous or asynchronous way without affecting the overall computation, as long as there is enough time for the individual dynamics to reach their corresponding equilibria. The current algorithm embeds dynamics from both the input and the output (target) signals to the neural dynamics through the feedback and the non-linear coupling. In addition, during the backward phase, a feedback corrects the error of the network based on the target signal. This error is propagated backward through constant random matrices. This draws some similarity to target propagation [4], where the gradient of the loss is computed with respect to the output and is propagated backwards to the previous layers of the network. We demonstrate that the proposed model is capable of performing on a variety of different tasks, such as digit (MNIST, 98% test accuracy) and letter classification (eMNIST, 85% test accuracy), logical operation (XOR problem, 100% accuracy), sequence prediction (successful prediction of a sinusoidal wave and Lorentz attractor), and an autoencoder encoding and decoding MNIST data set. The proposed learning scheme can be used in combination with other neural models so that more complex biological phenomena can be studied.

**References**

1. Hinton GE, McClelland JL. In *Neural information processing systems*, 358–366, 1988.
2. Xie X, Seung HS. *Neural Computation*, 15(2), 441–454, 2003.
3. Lillicrap TP, Cownden D, Tweed DB, Akerman CJ. *Nature Communications*, 7, 13276, 2016.
4. Lee DH, Zhang S, Fischer A, Bengio Y. *In ECML PKDD*, 498–515. Springer, 2015.

## P67 Activity of neural circuit in V1 during locomotion demystified

### Doris Voina^1^, Stefan Mihalas^2^, Stefano Recanatesi^3^, Eric Shea-Brown^1^

#### ^1^University of Washington, Department of Applied Mathematics, Seattle, WA, United States; ^2^Allen Institute for Brain Science, Modelling, Analysis and Theory, Seattle, WA, United States; ^3^University of Washington, Department of Physiology and Biophysics, Seattle, WA, United States

##### **Correspondence**: Doris Voina (dorisvoina@gmail.com)

*BMC Neuroscience* 2018, **19(Suppl 2):**P67

The processing of visual information depends strongly on the statistics of features in the visual field. The visual circuit that processes this information should be both robust to the precise statistics of the visual environment, and flexible to account for a broad variety of visual features. Perhaps the most compelling changes in visual statistics are due to movement, yet how the neural circuitry underlying visual processing accounts for these remains unknown. Allen Brain Observatory data provided by the Allen Institute for Brain Science, along with other studies, suggest that the brain’s response to sensory input is strikingly modulated by locomotion. Specifically, the VIP group of neurons becomes preferentially activated during locomotion and influences multiple synaptic pathways in V1. The goal of this study is to investigate synaptic weights and firing rates of populations of neurons in V1 thought to be responsible for the coding of Gabor-like features and explain how these change when the animal switches behavioral state (from static to running an vice versa). The activity of these neurons is determined not only by their receptive fields, but also by lateral connections which modulate activity due to the surround. VIP neurons have been shown to interact with this circuit in a switch-like fashion, but there is presently no computational model that accounts for the algorithmic consequences of these interactions. We use a Bayesian model previously developed for visual inference in both images and videos (thus emulating what animals would see while moving in the environment). In this model the connection between neurons primarily depends on the co-occurrence probability of features that the neuron responds preferentially to. The differences in the synaptic connectivity computed on images and videos capture the predicted influence of movement on the neural processing of visual information. The model further enables us to propose a role for VIP neurons at the circuit level, and to explain movement dependent changes in the signaling pathways. Finally, the obtained neuronal activity trends can be compared to activities of neurons in mice brains’ during a visual recognition task when the mice are running. As such our results may play a key role in interpreting the high variability seen in V1 activity.

## P68 Dimensionality of recurrent neural networks trained to classify spatially clustered inputs

### Matthew Farrell^1^, Stefano Recanatesi^2^, Eric Shea-Brown^1^

#### ^1^University of Washington, Department of Applied Mathematics, Seattle, WA, United States; ^2^University of Washington, Department of Physiology and Biophysics, Seattle, WA, United States

##### **Correspondence**: Matthew Farrell (msf9@uw.edu)

*BMC Neuroscience* 2018, **19(Suppl 2):**P68

The functional role of neural circuits in the brain can be modeled as a transformation of inputs into some desired outputs. In this work, a recurrent neural network is trained to classify spatially clustered inputs into classes. This task serves as a testbed to ask questions about the nature of neural representations as clusters are transformed into outputs defined by class assignment. We use a metric of dimensionality to quantify this transformation and see how it behaves as a function of the input and output dimension. We explore possible ways that this metric can be used to gain deeper insight into the workings of the network and used to influence the training process, resulting in new classes of solutions (Fig. 1).

**Fig. 1:**
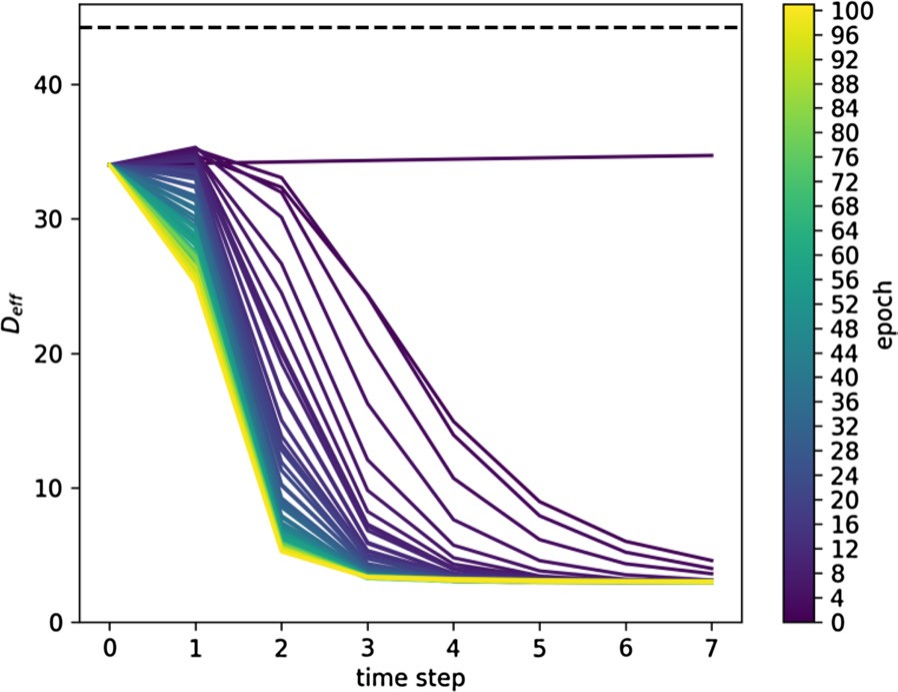
In the simple case of high dimensional input and low dimensional output (here there are sixty input clusters and four class labels), the dimensionality of the network representation smoothly transforms from high to low across the unrolling of the network dynamic (time step). More interesting phenomena happen when the task parameters are modified

## P69 How connectivity motifs shape the dimensionality of network response

### Stefano Recanatesi^1^, Gabriel Ocker^2^, Eric Shea-Brown^3^

#### ^1^University of Washington, Center for Computational Neuroscience, Seattle, WA, United States; ^2^Allen Institute for Brain Science, Modelling, Analysis and Theory, Seattle, WA, United States; ^3^University of Washington, Department of Applied Mathematics, Seattle, WA, United States

##### **Correspondence**: Stefano Recanatesi (stefanor@uw.edu)

*BMC Neuroscience* 2018, **19(Suppl 2):**P69

How does the connectivity of a network system combine with the input that the network receives so to shape the response to the input itself? We approach this question by relating the internal network response to the statistical prevalence of connectivity motifs, a set of surprisingly simple and local statistics on the network topology. The resulting motif description provides a reduced order model of the network dynamics. Through this framework we compute the dimensionality of the response. The dimensionality that we study is tightly link to the number of PCA components that are needed to describe the state of the network. We study this measure at the vary of the connectivity (statistics of motifs) and of the input structure. We find that different network topologies are able to expand or compress the dimensionality and this can be accomplished locally at the single neuron level by increasing or decreasing specific network motifs (e.g. divergent connections). Furthermore, we link these properties to how the network responds to inputs. The total dimensionality of the network response does depend on the input properties and in particular by the strength of the input drive and its dimensionality. The network can then operate in different regimes by compressing the input dimensionality or by matching it being more or less sensitive to the input drive. At last we connect these properties to whether the network is fully excitatory or balanced (considering a balanced network of excitatory and inhibitory neurons). Balanced networks show a variety of behaviors that go beyond the capabilities of fully excitatory systems. We characterize how the dimensionality of such systems varies with connectivity motifs and to input properties. Overall the framework we develop provides powerful theoretical tools to understand the functionality of neural network systems in terms of high level descriptors such as the dimensionality that are linked to neural correlations and neural representation properties.

## P70 Action potential propagation in axons: Effect on sodium conductance of collateral and sub-branch distance from soma

### Ngwe Sin Phyo, Erin Munro Krull

#### Beloit College, Department of Mathematics and Computer Science, Beloit, WI, United States

##### **Correspondence**: Erin Munro Krull (ecmun2002@yahoo.com)

*BMC Neuroscience* 2018, **19(Suppl 2):**P70

Realistic axons are complex. This makes it difficult to predict the behavior during action potential (AP) propagation. It is often assumed that APs successfully propagate down the axon. When previous literature investigated APs in axons, it predicted AP propagation in electronically symmetric axons. Yet, we cannot predict propagation in electronically asymmetric axons. In this study, we looked at the sodium conductance (gNa) of the axon, which determines the axon’s excitability. We initiated APs in a collateral branch and tested if it successfully propagates to the end of the axon. The simplest model we used was a neuron with maximum three collateral branches. We simulated our neurons, studying the threshold gNa required for APs to propagate while varying the distance of the collateral branches along the axon and sub-branches within collateral branches. From this research, we would like to develop a theory to predict AP propagation. That, in turn, we hope will tell us more about how neurons compute. Since the neurons are basic blocks of our nervous system, we also hope this will help future studies to improve treatment for neurological disorders.

## P71 Action potential propagation in axons: how sodium conductance can estimate propagation as collateral and sub-branch length vary

### Yizhe Tang, Erin Munro Krull

#### Beloit College, Department of Mathematics and Computer Science, Beloit, WI, United States

##### **Correspondence**: Erin Munro Krull (ecmun2002@yahoo.com)

*BMC Neuroscience* 2018, **19(Suppl 2):**P71

Predicting when an action potential can propagate in neuronal axons is a long-outstanding problem in both mathematics and neuroscience. Previous related research showed when an axon is electrotonically symmetric, the action potential propagation can be predicted. However, most axons are not electrotonically symmetric. This research uses simulation to provide evidence by looking at a key parameter: the sodium conductance of the axon. We hope to generate a fundamental theory to predict the action potential propagation linearly that can be used for different axon geometries. Predicting action potential propagation may help us better understand neuron computation as well as how disorders may affect computation. For instance, axonal sprouting as seen in epilepsy may hinder propagation. My colleagues and I each looked at different configurations of axons. I tested the case where the length of a sub-branch on a collateral branch varies. I looked at four parameters: the electrotonic length of the sub-branch, the electrotonic length of collateral branch, the distance of the sub-branch from the main axon, and the distance of the parent branch from the soma. We may approximate how the sub-branch affects propagation by looking at different combinations of these four parameters.

## P73 The ratio of specialist and generalist neurons in the feature extraction phase determines the odor processing capabilities of the locust olfactory system

### Aaron Montero, Jessica Lopez-Hazas, Francisco B Rodriguez

#### Universidad Autónoma Madrid, Ingeniería Informática, Madrid, Spain

##### **Correspondence**: Aaron Montero (aaron.montero.m@gmail.com)

*BMC Neuroscience* 2018, **19(Suppl 2):**P73

Measuring the processing capabilities of different nervous systems has always been an interesting point for neuroscience. We observed that some capabilities can be measured by the ratio of specialist and generalist neurons that belong to the Kenyon cells (KCs) of the locust olfactory system [1]. These types of neurons are part of the neural diversity of the biological nervous systems, specifically, they represent the heterogeneity in neural response to stimuli. While specialists react to a few stimuli, generalists respond to a wide range of them [2]. Hence, it is suggested that specialist neurons are essential for stimuli discrimination and generalist ones extract common and generic properties from them [3]. The requirement of specialists for pattern recognition was proven by us [4], but we also observed that sometimes generalists were needed for this task. Thus, there is a certain ratio of these two types of neurons depending on the stimulus complexity [1]. When the input complexity was low, the minimum classification error was achieved with almost any ratio of specialists/generalists (S/G). When this complexity was intermediate, both were required to minimize the classification error, usually in a similar proportion. Finally, when the complexity was high, only specialists were needed for the error minimization. As we linked the complexity level to a S/G ratio and to a classification success [1], we can invert this relationship to estimate the stimuli complexity and olfactory system accuracy by analyzing the S/G number from neural recordings. Therefore, we used recordings from KCs of the locust for calculating this ratio, based on the neural responses of 43 neurons for 17 different stimuli [5]. We estimate that the percentage of generalists in the KCs of locust is 23.26% [6]. This ratio involves an intermediate complexity of 51.34% according to our calculations, which also provides information about the number of differentiable odors by the locust since complexity and capacity seem to be related [7]. To contrast these results, we measured the complexity of patterns in the projection neurons (PNs) of the antennal lobe, using the recordings of 14 PNs for 3 different odorants. The complexity degree observed for this reduced number of neurons and odors was 63.38% that is not too far from the 51.34% calculated from KCs. This complexity implies that all PNs are generalists, which coincides with the recordings data [5]. Finally, from the two complexity values shown, we can estimate that the reliability of odor discrimination process in the locust could be comprised between 74.87% and 92.04%.

**Acknowledgements**

We thank Ramon Huerta for his helpful discussions and Javier Perez-Orive and Gilles Laurent for providing the neural recordings of locust. This research was supported by the Spanish Government projects TIN2014-54580-R and TIN2017-84452-R.

**References**

1. Montero A, Huerta R and Rodriguez FB. Stimulus space complexity determines the ratio of specialist and generalist neurons during pattern recognition. *Journal of the Franklin Institute* 2018, 355(5), 2951–2977.
2. Christensen TA. Making scents out of spatial and temporal codes in specialist and generalist olfactory networks. *Chem*. *Senses* 2005, 30283–284.
3. Wilson RI, Turner GC, Laurent G. Transformation of olfactory representations in the Drosophila antennal lobe. *Science* 2004, 303(5656) 366–370.
4. Montero A, Huerta R, and Rodriguez FB. Specialist Neurons in Feature Extraction Are Responsible for Pattern Recognition Process in Insect Olfaction. In *International Work*-*Conference on the Interplay Between Natural and Artificial Computation*, Springer, Cham, 2015. part I p. 58–67.
5. Perez-Orive J, Mazor O, Turner GC, Cassenaer S, Wilson RI and Laurent G. Oscillations and sparsening of odor representations in the mushroom body. *Science* 2002, 297(5580), 359–365.
6. Rodriguez FB and Huerta R. Techniques for temporal detection of neural sensitivity to external stimulation. *Biological cybernetics* 2009, 100(4), 289–297.
7. Rodriguez FB and Huerta R. Analysis of perfect mappings of the stimuli through neural temporal sequences. *Neural networks* 2004, 17(7), 963–973.

## P74 Regulation of neural threshold in Kenyon cells through their sparse condition improves pattern recognition performance

### Jessica Lopez-Hazas, Aaron Montero, Francisco B Rodriguez

#### Universidad Autónoma Madrid, Ingeniería Informática, Madrid, Spain

##### **Correspondence**: Aaron Montero (aaron.montero.m@gmail.com)

*BMC Neuroscience* 2018, **19(Suppl 2):**P74

Insect olfactory system is capable classifying an almost infinite quantity of odorants at different concentrations. This task is carried out in three processing stages, starting at the insect antenna, passing through antennal lobe (AL) and finally the odorants are classified at the mushroom body (MB). The strategies that the system applies in each of these layers to discriminate stimuli have been extensively studied. Regarding the AL and MB, three mechanisms have been proposed as of great importance to assure and improve the success of odorant classification. These strategies are an heterogeneous threshold distribution in the Kenyon Cells (KCs) at the MB [1, 2], a mechanism for gain control at the AL layer [3] and sparse coding in the KCs [5] layer to improve pattern differentiation while providing energetic efficiency. In this work, we use a model of the insect olfactory system that takes into account the biological facts about the network architecture and also includes the three strategies explained above. The model is based on neural networks and supervised learning [7, 2] and our goal is to study how information processing takes place in the biological system by testing the relevance of these mechanisms in the energetic cost and the performance of the network on a pattern classification task, paying more attention to threshold distribution and sparse coding, The heterogeneous thresholds are introduced in the model through a learning algorithm that allows the network to find an optimum threshold distribution for KCs for a certain classification problem. Gain control is achieved through the renormalization of patterns in the input layer so that the activation of the neurons is uniform for all patterns. Finally, an activity regulation term is introduced in the supervised algorithm learning rule with the aim of controlling the level of activity in the KCs. The activity regulation term (ART) is defined as*1/N*KC [SUMKC(yi-s)]2, where the parameter *N*KC is the number of KCs, *s ∈ [0*,*1]* allows to control the level of activity in the KCs layer from no activity with *s *=* 0* to maximum activity with *s *=* 1*, and *y*i is the activation of i-th KC in the network. The results show that a model including the activity regulation term outperforms one that lacks it for the classification problem presented (a simplified version of MNIST dataset [8]). Also, the model obtains better results when the connection probability between AL and MB neurons is low, in the interval [0.1–0.3], and the sparsity level in KC layer is high, which is consistent with what is observed in the real biological system [5, 6] and assures energetic efficiency.

**Acknowledgements**

We thank Ramón Huerta for his useful discussions on this work. This research was supported by the Spanish Government projects TIN2014-54580-R and TIN2017-84452-R.

**References**

1. Montero A, Huerta R, Rodriguez FB. *Neurocomputing*, 2015, 151, 69–77.
2. Montero A, Huerta R, Rodríguez FB. Springer, Berlin, Heidelberg 2013, 16–25.
3. Montero A, Mosqueiro T, Huerta R, Rodriguez FB. Springer, Cham 2017, 317–26.
4. Olsen SR, Wilson RI. *Nature*. 2008, 452:956.
5. Perez-Orive J, Mazor O, Turner GC, Cassenaer S, Wilson RI, Laurent G. *Science*. 2002, 297, 359–65.
6. Sanda P, Kee T, Gupta N, Stopfer M, Bazhenov M. 2016, 115, 2303–16.
7. Huerta R, Nowotny T, García-Sanchez M, Abarbanel HDI, Rabinovich MI. *Neural Comput*. 2004, 16, 1601–40.
8. MNIST handwritten digit database [http://yann.lecun.com/exdb/mnist/]

## P76 Local excitatory/inhibitory imbalances shape global patterns of activity: A model for desynchronized activity under anesthesia in Alzheimer’s disease

### Merav Stern^1^, Gabriel Ocker^2^

#### ^1^University of Washington, Department of Applied Mathematics, Seattle, WA, United States; ^2^Allen Institute for Brain Science, Modelling, Analysis and Theory, Seattle, WA, United States

##### **Correspondence**: Merav Stern (merav.stern@mail.huji.ac.il)

*BMC Neuroscience* 2018, **19(Suppl 2):**P76

It has been shown that the highly correlated neural activity known to appear under anesthesia is severely reduced in a mouse model of Alzheimer’s disease (AD) [1]. It has also been shown that AD mice develop a sub-group of silent excitatory neurons alongside with highly hyperactive excitatory neurons [2] and that the correlated neuronal activity under anesthesia can be restored by enhancing the inhibitory synaptic inputs into the hyperactive excitatory neurons [1]. Taken together, these studies suggest that in AD mice changes in the balance of inhibitory connections to subgroups of excitatory cells shift network-wide activity. We propose a neural network model that explains these phenomenological changes in the overall network behavior. We characterize how a separation between excitatory and inhibitory functional connectivity gives rise to correlated population activity. Our analysis explains why these correlations are disrupted by changes in circuit connectivity.

Our model includes rate-based neuron units that are explicitly separated into excitatory and inhibitory types. Hence, our model connectivity matrix is constrained to have columns with positive entries and columns with negative entries, representing input from excitatory and inhibitory populations accordingly. The eigenvalue spectra of such random matrices have been shown to have outliers [3] which we further constrain by requiring a tight balance—a sum of excitatory and inhibitory connections into each unit that are matched exactly. This constraint has been shown to remove the outlier eigenvalues [4] and give rise to highly correlated activity across the network with slow-wave-like dynamic of the mean activity [5] that resembles activity in wild-type mice.

In addition to this population-wide component of activity, our tightly balanced network model exhibits chaotic fluctuations of single-unit activity around the population mean. We show that the residual activity resembles fully random neural network models but with a time-varying magnitude that depends on the mean activity. The strength of the residual chaotic activity in the tightly balanced network is determined by the variance of the synaptic strengths, while the magnitude of the correlated activity component is determined by the mean strengths of the excitation and inhibition. We model the pathology observed in AD mice by breaking the tight balance between excitation and inhibition within subgroups of excitatory neurons, while maintaining the overall excitation/inhibition balance in the network. This pathology shifts the network into an uncorrelated, chaotic state that resembles the recordings from AD mice.

**References**

1. Busche MA, Kekus M, Adelsberger H et al. Rescue of long-range circuit dysfunction in Alzheimer’s disease models. *Nature Neuroscience* 2015, 18, 1623–30
2. Busche MA, Eichoff G, Adelsberger H et al. Clusters of hyperactive neurons near amyloid plaques in a mouse model of Alzheimer’s disease. *Science* 2008, 321, 1689–9
3. Tao T, Hu V, Krishnapur M. Random matrices: Universality of ESDs and the circular law. *arXive* 2010, 38(5), 2023–65
4. Rajan K, Abbott LF. Eigenvalue spectra of random matrices for neural networks. *Phys*. *Rev*. *Lett*. 2006, 97(18)
5. Stern MS, Abbott LF. CNS meeting 2016; Takashi H, Fukai T. *arXiv* 2017; Landau I, Sompolinsky H. COSYNE meeting 2018

## P77 Neural automata

### Martin Schumann^1^, Gabriele Scheler^2^

#### ^1^Technical University of Munchen, Computer Science, Munich, Germany; ^2^Carl Correns Foundation for Mathematical Biology, Mountain View, United States

##### **Correspondence**: Martin Schumann (j.martin.schumann@gmail.com)

*BMC Neuroscience* 2018, **19(Suppl 2):**P77

We present a first view of a new type of neuron model currently under development which is significantly different from membrane or action potential based models. The neuron is conceptualized as a unit with an internal processing system G (including the proteome and the nuclear transcription system), an axonal system A, conceptualized as a Boolean vector plus parameters h attached to each vector unit (presynapse), and a dendritic system D (postsynapse) similar to system A but with an evaluation function F. GLIF type models can be recovered by equating G with a sigmoidal activation function, F with a majority rule, and parameters h with synaptic weights. We concentrate on the interaction of G and h under random conditions. A system of neurons of 100–1000 neurons is set up with systemically differing conditions, according to the biological observations on lognormal properties [1]. We load it with patterns which adjust h parameters by Hebbian learning and where the h parameters inform the internal network G to adjust protein expression levels. The internal network G will reach a state where the internal values are read out to adjust h parameters, in this way altering the processing properties of the neurons, both dendritic (postsynaptic) and axonal (presynaptic). So we have an internal storage of previous information that can adjust h parameters at a later time. A neuron may be ready for read-out after a succession of storage events (avalanche model), but different rules also may be used. The system is able to replicate detailed data on neural plasticity (e.g. [2]). It creates levels of memory for pattern storage and retrieval. The h parameters are able to record an active pattern and to construct a frequentist representation by pre- and postsynaptic connections. The internal G system stores selected features from the h system and rewrites them back to the system. In this way the learned patterns of synaptic connectivity can be adjusted and locally overwritten by the internal storage systems G. Synaptic connectivity overall is adjusted based on those local overwrites through continuing network activity. The G-based overwrite may happen continuously or according to an avalanche model, i.e. rare updates followed by a concerted rewrite of all instances of h values that have differing G system values. We evaluate the system at first by a randomized overwrite for h in order to study the evolution of the system between elimination of overwritten weights and escalation/dominance of these weights. This is the basis for meaningful editing, which allows to process information. The results of the random test runs are used to evaluate storage and processing properties of the combined G/h system. The G read-out extends to the Boolean evaluation functions f. At present, these operate according to majority rules, but they can be edited to include localized cluster supralinear summation or inhibitory veto. The edit of the Boolean evaluation function will be studied separately, also in a randomized fashion. The goal of the system is to perform meaningful pattern memory and abstraction tasks.

**References**

1. Scheler G. Logarithmic distributions prove that intrinsic learning is Hebbian. *F1000Research* 2017, 6, 1222
2. Dehorter N, Ciceri G, Bartolini G, et al. Tuning of fast-spiking interneuron properties by an activity-dependent transcriptional switch. *Science* 2015, 349(6253) 1216–1220

## P78 Predictable variability in sensory-evoked responses in the awake brain: optimal readouts and implications for behavior

### Audrey Sederberg^1^, Aurélie Pala^1^, He Zheng^1^, Biyu He^2^, Garrett Stanley^1^

#### ^1^Georgia Institute of Technology, Coulter Dept. of Biomedical Engineering, Atlanta, GA, United States; ^2^Langone Medical Center, New York University, Departments of Neurology, Neuroscience and Physiology, and Radiology, New York, NY, United States

##### **Correspondence**: Audrey Sederberg (audrey.sederberg@gatech.edu)

*BMC Neuroscience* 2018, **19(Suppl 2):**P78

In a near-threshold sensory detection task, an animal sometimes detects and sometimes misses the same physical stimulus. A simple hypothesis is that perceptual variability is linked to variability in sensory-evoked responses in the brain as early as primary cortex. Response variability arises in part from the interaction of sensory inputs with ongoing activity and is partially predictable based on the pre-stimulus cortical state. If variability in evoked responses is linked to perception, and if that variability is predictable, we would expect that it would be possible to predict based on ongoing activity whether sensory cortex is primed to detect a sensory input. Here, we determine the pre-stimulus features that are predictive of variability in the evoked response in the awake animal. We then ask what implications these observations have for the detectability of a stimulus. Using data obtained from multi-electrode recordings across the cortical depth in S1 of awake mice, we systematically quantify how much variability in the sensory-evoked LFP response is predictable from ongoing LFP activity (Fig. 1AB). This interaction has been studied extensively in the anesthetized animal [e.g., 1, 2], where the major predictors of response variability are the degree of cortical synchronization, quantified by the amount of low-frequency power, and the phase of low-frequency oscillations at which sensory input occurred. Similarly, we found that the degree of synchronization was predictive, but instead of oscillation phase, the instantaneous level of activation of the LFP in layer 4 was a useful predictor. Specifically, positive excursions in the LFP and more low-frequency (1–5 Hz) power in the LFP in the pre-stimulus period predicted larger sensory-evoked responses (“high-response state”). Using a regularized estimator of current-source density (CSD)[3] on single trials, we localized the most predictive ongoing signal to a current source location near layer 4. Finally, we found that no significant predictive power was gained by increasing the complexity of the decoder or by utilizing the full array of channels. Thus, the most predictive signatures of ongoing activity are remarkably simple and could be accessible to downstream areas. Next, we examined the impact of predictable variability on an ideal observer analysis of the detectability of sensory events (Fig. 1C). We built a detection model, in which the detection threshold is either fixed, or adaptive and based on the pre-stimulus features that are predictive of evoked variability. We quantified the accuracy of the model in terms of the simulated hit rate and the false alarm rate. Detection was more accurate in the adaptive threshold model. In the fixed-threshold model, pre-stimulus features predicted hit and miss trials. This relationship was weaker in the adaptive-threshold model, where hits as well as false alarms were nearly equally as likely to occur in low- or high-response state. In summary, if sensory perception is built on the cortical response and variability in this response is completely unpredictable, then perceptual variability would to some extent be determined by cortical variability. However, if cortical variability is predictable and downstream circuits in the brain make this prediction, then the perceptual variability could be decoupled from cortical variability.

**Fig. 1:**
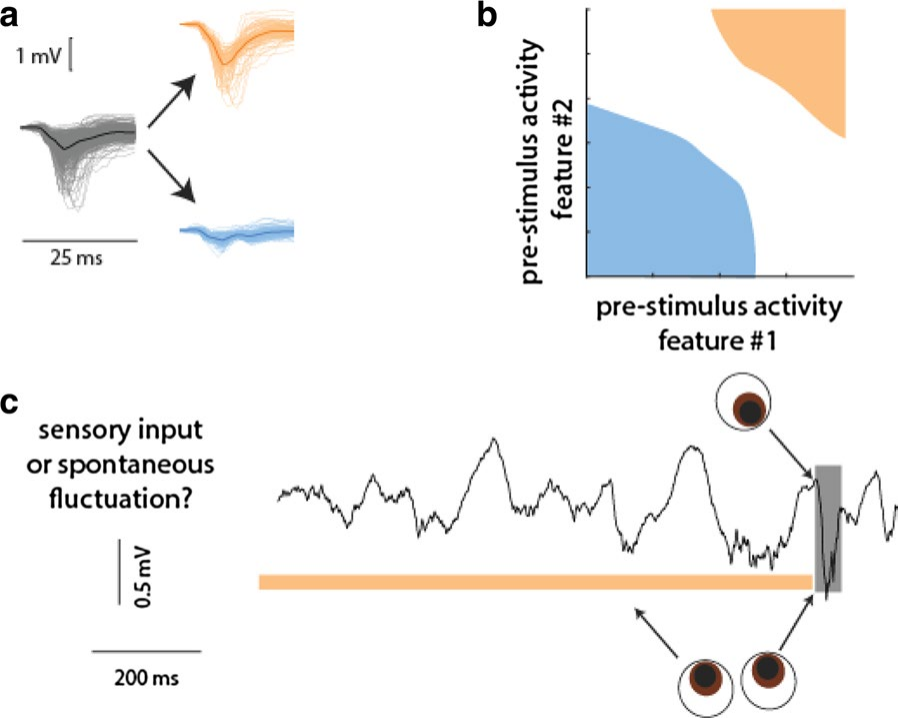
A: Sensory-evoked responses are variable. Here we predict whether responses are large (orange) or small (blue), using (B) features of the pre-stimulus activity. C: Ideal observer analysis of spontaneous and sensory-evoked activity, based on matched filter detection alone (single eye) or pre-stimulus state-dependent matched-filter detection (pair of eyes). LFP trace shown is a period of spontaneous (no sensory inputs) activity in which accounting for pre-stimulus state (during orange bar) would prevent a false alarm (gray box)

**References**

1. Curto C, Sakata S, Marguet S, et al. A Simple Model of Cortical Dynamics Explains Variability and State Dependence of Sensory Responses in Urethane-Anesthetized Auditory Cortex. *J Neurosci* 2009, 29(34), 10600–10612.
2. Kelly C, Uddin LQ, Shehzad Z, et al. Broca’s region: linking human brain functional connectivity data and non‐human primate tracing anatomy studies. *J Comput Neuro* 2010
3. Potworowski J, Jakuczun W, Leski S, et al. Kernel current source density method. *NECO* 2012, 24(2), 541–575.

## P79 Selectivity and sensitivity of cortical neurons to electric stimulation using ECoG electrode arrays

### Pierre Berthet^1^, Espen Hagen^1^, Torbjørn V Ness^2^, Gaute Einevoll^2^

#### ^1^University of Oslo, Department of Physics, Oslo, Norway; ^2^Norwegian University of Life Sciences, Faculty of Science and Technology, Ås, Norway

##### **Correspondence**: Pierre Berthet (berthetp@gmail.com)

*BMC Neuroscience* 2018, **19(Suppl 2):**P79

High-resolution, non-penetrating devices for direct electric stimulation of sensory cortex have the potential to become neuroprosthetic devices that can compensate for deficits in sight or hearing. The effect of extracellular electric stimulation with such devices, however, has so far not been investigated thoroughly, in particular in context of the variety of stimulation patterns possible with high-density electrode arrays and neuron types. In the context of visual neuroprosthetic devices, the ability to selectively stimulate different groups of neurons to potentially create many different phosphenes (visual impressions), is important.

Here, we combine neuronal modeling and electrostatic volume-conductor theory to investigate the effect of electrical stimulation on the generation of neuronal action potentials. In general a successful stimulation will depend on properties of the neuron like the position, morphology, and membrane properties, as well as the electrical stimulation pattern, i.e., the geometrical arrangement of the stimulating contacts, electric pulse amplitudes and temporal forms, etc. To quantify the stimulus excitability of the neurons we first consider the sensitivity, that is, the minimum stimulation current amplitude (threshold current) needed to generate an action potentials for a particular neuron and stimulation pattern. We also investigate the selectivity, that is, the dependence of the threshold current on the position of the neuron. Biophysically detailed multicompartment models of cortical neurons using the NEURON simulation environment [1] and LFPy [2] are used in the simulation. The neurons are assumed to be embedded in an infinite homogeneous, isotropic and ohmic medium. We compute electric potentials as generated by intracranial electroencephalography (ECoG) electrode arrays, and impose these as boundary conditions for the electric potential immediately outside each neuronal compartment. These imposed potentials in turn affect the neuronal dynamics, and the generation of action potentials.We first study stylized morphologies and demonstrate a critical role of their orientation and position relative to the applied electric field, and also of the polarity of the stimulation current [3, 4]⁠. We further investigate the sensitivity and selectivity of morphologically detailed biophysical models, including models from the Allen Brain Institute and the Blue Brain Project, to various configurations of the electrode arrays.

**Acknowledgement**

Neural Engineering System Design (NESD) program from the Defense Advanced Research Projects Agency (DARPA).

**References**

1. Carnevale NT, Hines ML. *The NEURON Book*. *Cambridge*: Cambridge University Press, 2006.
2. Lindén H, Hagen E, Łęski S, et al. LFPy: a tool for biophysical simulation of extracellular potentials generated by detailed model neurons. *Front*. *Neuroinform*. 2014, 7(1), 1–15.
3. Rattay F, Modeling the excitation of fibers under surface electrodes. *IEEE Trans*. *Biomed*. *Eng*. 1988, 35(3), 199–202.
4. Rattay F. The basic mechanism for the electrical stimulation of the nervous system. *Neuroscience* 1999, 89(2), 335–346.

## P80 Patterns of gastrointestinal motility and the effects of temperature and menthol: A modelling approach

### Parker Ellingson^1^, Taylor Kahl^1^, Sarah Johnson^1^, Natalia Maksymchuk^1^, Sergiy Korogod^2^, Chun Jiang^3^, Gennady Cymbalyuk^1^

#### ^1^Georgia State University, Neuroscience Institute, Atlanta, GA, United States; ^2^Bogomoletz Institute of Physiology, National Academy of Sciences of Ukraine, Kiev, Ukraine; ^3^Georgia State University, Department of Biology, Atlanta, GA, United States

##### **Correspondence**: Parker Ellingson (pellingson3@gmail.com)

*BMC Neuroscience* 2018, **19(Suppl 2):**P80

Proper digestive functioning requires a variety of coordinated activities in the gastrointestinal tract. During the starved state, peristaltic waves are the dominant pattern of motility in the small intestine. After a meal, the intestine switches to a mixing pattern reminiscent of the beating pattern produced by interacting oscillators with different frequencies. This helps to break up food particles and increase nutrient absorption. These patterns are also modulated by temperature and a variety of pharmacological agents. Rhythm-generating cells electrically connected to the smooth muscle known as interstitial Cells of Cajal (ICC) drive the patterns of motility. The mixing pattern is particularly dynamically interesting and is generally explained as an interaction of two classes of ICC, oscillating at different frequencies (1). We suggest that intrinsic dynamics of a single ICC can explain the mixing pattern as well. We developed models of ICC contains intracellular calcium dynamics, and Hodgkin-Huxley representations of key ionic currents. Both the endoplasmic reticulum and mitochondria are intracellular calcium stores that could produce calcium oscillations with different periods in the cytosol. We used a mathematical model of subcellular dynamics (4) to observe interactions between calcium oscillations from the ER and mitochondria. Variation in the concentration of one organelle can control the period of oscillation through the other organelle. A combination of two of the subcellular models, assuming weak diffusive coupling, produced a beating pattern. We compared the results of this model to our experimental recordings of muscle contractions from murine small intestines. Our model suggests a mechanism for this mixing pattern: interactions between two oscillatory calcium subsystems in a single ICC. We also investigated the effects of temperature on motility patterns by adjusting Q10 values and incorporating the dynamics of TRPA1 channels into our model. These results explain how temperature can affect the frequency of oscillations, which is consistent with experimental data (3). As the TRPA1 channel is also sensitive to menthol, we show that our model reproduces experimental data on menthol treatment of ICC (2). This model shows that factors affecting the internal calcium dynamics impact the period of oscillations, while factors which affect membrane based currents primarily affect amplitude In conclusion, we demonstrate that ICC are capable of producing a variety of basic regimes of activity corresponding to key motility patterns.

**Acknowledgement**

This project was supported by the GSU Brains and Behavior program

**References**

1. Huizinga JD et. al. The Origin of Segmentation Motor Activity in the Intestine. *Nature Comm* 2014. 5, 3326.
2. Kim HJ, Wie J, So I, Jung MH, Ha KT, Kim BJ. Menthol Modulates Pacemaker Potentials through TRPA1 Channels in Cultured Interstitial Cells of Cajal from Murine Small Intestine. *Cell Physiol Biochem*. 2016, 38(5), 1869–82
3. Kito Y, Suzuki H. Properties of pacemaker potentials recorded from myenteric interstitial cells of Cajal distributed in the mouse small intestine. *J Physiol*. 2003, 552, 803–818.
4. Marhl, M, Haberichter, T, Brumen, M, Heinrich, R. Complex calcium oscillations and the role of mitochondria and cytosolic proteins. *Biosystems* 2003. 57, 75–86.

## P81 Mechanisms underlying locomotion and paw-shaking rhythms in cat multifunctional central pattern generator

### Jessica Green^1^, Boris Prilutsky^2^, Gennady Cymbalyuk^1^

#### ^1^Georgia State University, Neuroscience Institute, Douglasville, GA, United States; ^2^Georgia Institute of Technology, Department of Biology, Atlanta, GA, United States

##### **Correspondence**: Jessica Green (jgreen59@student.gsu.edu)

*BMC Neuroscience* 2018, **19(Suppl 2):**P81

Could two drastically different rhythms such as in cat locomotion and paw-shaking be controlled by the same network of neurons? To answer this question, we built a model of a multifunctional central pattern generator (CPG). Our model, constructed as a half-center oscillator (HCO), is able to produce multistability of a locomotion-like rhythm and a paw-shake-like rhythm. It uses a novel mechanism involving two slow currents, i.e. slowly inactivating calcium current and slowly inactivating sodium current [1]. Transient paw-shake-like activity can be elicited in our model, and this transient activity exhibits asymmetric trends throughout consecutive bursts in accordance with experimental data. Here, our model has only the locomotion-like rhythm present, and generates only transient paw-shake-like activity. We investigated the model’s responses to various types of afferent stimulation during locomotion-like activity and transient paw-shake-like activity. We predict that applying a 1-second pulse of current to groups Ia and II afferents from cat hip flexors and extensors during locomotion, which have access to the flexor- and extensor half-centers of CPG rhythm generator [2], will evoke a paw-shake response in that hindlimb. According to our model, the duration of this transience depends on the phase of stimulation in the locomotion rhythm. Also, the duration of transience increases with the duration of the pulse. The duration of transient paw-shake-like activity could be extended when a short pulse of current is applied during transient paw-shake-like bursting. We predict that applying a short 20-millisecond pulse of excitatory current to groups Ia and II afferents from either hip flexors or extensors during a paw-shake response will extend the duration of the paw-shake response. Furthermore, the duration of the paw-shake response would increase as the duration of this stimulus increases until some threshold duration is reached at which the duration of the paw-shake response will remain roughly constant as the stimulus duration increases. In addition, the extension of the response would depend on the phase of pulse application in the paw-shaking cycle. The extended paw-shake response could last longer if the pulse is applied during the extensor phase as opposed to the flexor phase if the pulse is applied near the beginning of the paw-shake response. The asymmetry weakens if the pulse onset is delayed during the paw-shake response. These predictions are robust and can be tested experimentally to investigate whether the obtained responses during locomotion and paw-shaking are consistent with the idea that the two rhythmic behaviors are generated by the same multifunctional CPG. Confirming these predictions experimentally would provide strong evidence for the hypothesis that the paw-shake response in cats is generated as a transient response of the locomotion CPG.

**Acknowledgements**

We acknowledge support by the Brains and Behavior Fellowship for Jessica Parker at Georgia State University and by NIH P.01 HD32571, R01 EB012855, and R01 NS100928 to Boris I. Prilutsky.

**References**

1. Bondy B, Klishko AN, Edwards DH, Prilutsky BI, Cymbalyuk G: *Control of cat walking and paw*-*shake by a multifunctional central pattern generator*. *In: Neuromechanical Modeling of Posture and Locomotion*. edn. New York: Springer; 2016, 333–359.
2. McCrea DA, Rybak IA. Organization of mammalian locomotor rhythm and pattern generation. *Brain Res Rev* 2008, 57, 134–146.

## P82 The role of Na+/K+ pump in intrinsic intermittent bursting dynamics in model neuron of the Pre-Bötzinger Complex

### Alex Vargas, Gennady Cymbalyuk

#### Georgia State University, Neuroscience Institute, Atlanta, GA, United States

##### **Correspondence**: Gennady Cymbalyuk (gcymbalyuk@gmail.com)

*BMC Neuroscience* 2018, **19(Suppl 2):**P82

Central Pattern Generators (CPGs) are oscillatory neuronal circuits controlling rhythmic movements [1]. Movements like breathing have to be continually regulated for an animal to meet environmental demands [2–4]. The Pre-Bötzinger Complex (PBC), located in the medulla of the brainstem, produces patterns controlling inspiratory phase of breathing. Prolonged hypoxia leads to dysrhythmia in this CPG, causing apnea or cessation of breathing. We focus our study on potential role of Na+/K+ pump in intermittent intrinsic patterns which we discovered in a model of a Pre-Bötzinger Complex neuron. Our hypothesis is that these patterns are similar to intermittent patterns of tadpole swimming [5]. The major function of the Na+/K+ pump is to maintain the ion gradients of Na+ and K+ in a 3:2 exchange ratio, consuming one ATP molecule per cycle. The pump is electrogenic and activity dependent, it directly contributes to neuronal dynamics across entire voltage range of operation. By serving the function of maintaining the ionic gradients and contributing to neuronal dynamics, the pump presents advantages and pathological risks. We developed a model of a PBC neuron which is intrinsically bursting based on a persistent sodium current dynamics. The model describes dynamic intracellular Na+ concentration which determines the reversal potential for all sodium currents. Fast sodium, persistent sodium, delayed-rectifier potassium, slow calcium, leak, h-current and the pump contribute to membrane potential. The pump current is controlled by a parameter for maximal pump strength which reflects ATP levels within the cell. The higher value corresponds to more ATP available for the pump to consume. We demonstrate that our model produces functional bursting under normoxia and that the decrease of the pump strength corresponding to hypoxia generates intermittent bursting. Shifting K+ reversal potential (EK) drastically affected the interbout interval of the intermittent activity, the more hyperpolarized, the longer the interbout interval. The more depolarized EK is, the less of an increase in the maximal pump strength is necessary to restore functional activity. We investigated dynamical mechanisms underlying the role of the Na+/K+ pump. We find that this carries significance towards further understanding pathological vulnerabilites in the respiratory centers of the brain.

**References**

1. Marder, E. and R.L. Calabrese, Principles of rhythmic motor pattern generation. *Physiological reviews*, 1996. 76(3): p. 687–717.
2. Tryba, A.K., et al., Differential modulation of neural network and pacemaker activity underlying eupnea and sigh-breathing activities. *Journal of Neurophysiology*, 2008. 99(5): p. 2114–25.
3. Koizumi, H. and J.C. Smith, Persistent Na+ and K+ -dominated leak currents contribute to respiratory rhythm generation in the pre-Botzinger complex in vitro. *The Journal of Neuroscience* : the official journal of the Society for Neuroscience, 2008. 28(7): p. 1773–85.
4. Bell, H.J. and N.I. Syed, Hypoxia-induced modulation of the respiratory CPG. *Frontiers in Bioscience*, 2009. 14: p. 3825–3835.
5. Zhang, H.Y. and K.T. Sillar, Short-term memory of motor network performance via activity-dependent potentiation of Na+/K+ pump function. *Current Biology*: CB, 2012. 22(6): p. 526–31.

## P83 Changes in relaxation time predict stimulus-induced reduction of variability at the single-cell level

### Luca Mazzucato^1^, Ahmad Jezzini^2^, Alfredo Fontanini^3^, Giancarlo La Camera^3^, Gianluigi Mongillo^4^

#### ^1^Columbia University, Center for Theoretical Neuroscience, New York, NY, United States; ^2^Washington University, Department of Neuroscience, St. Louis, WA, United States; ^3^Stony Brook University, Department of Neurobiology and Behavior, Stony Brook, NY, United States; ^4^Université Paris Descartes, Centre de Neurophysique, Physiologie et Pathologie, paris, France

##### **Correspondence**: Luca Mazzucato (lmazzuca@uoregon.edu)

*BMC Neuroscience* 2018, **19(Suppl 2):**P83

It has been reported that stimulus presentation reduces the level of neuronal variability. The mechanism underlying this phenomenon, however, is yet to be elucidated. Here, we present evidence suggesting that changes in trial-to-trial variability are determined by changes in single-neuron relaxation times. We estimated non-parametrically the single-cell autocorrelation (AC) times during spontaneous and stimulus-evoked activity in the cortex (gustatory and pre-frontal) and the medio-dorsal thalamus of alert rats. We found broad distributions of AC times in all areas, ranging from less than 20 ms to more than 4 s (our largest observation window); their distributions were right-skewed and long-tailed. We found that single-cell AC times changed between the two conditions: neurons with spontaneous slow AC times became fast after stimulus presentation, and vice versa. We uncovered a relationship between changes in AC times (between spontaneous and evoked conditions) and stimulus-induced changes in trial-to-trial variability, at the single-neuron level. While the overall Fano factor dropped during evoked periods compared to spontaneous periods in all areas, consistent with previous reports, we found that such reduction was entirely driven by the subpopulation of neurons whose AC times was also reduced by the stimulus. Changes in AC time between spontaneous and evoked condition thus predict the observed changes in trial-to-trial variability at the single-cell level. These results suggest that local circuit dynamics in both cortex and thalamus evolves through sequences of metastable states, where state durations are modulated by stimulus presentation.

## P84 Intracellular fluxes contributing to [Ca2+]i responses in rat magnocellular neurons

### Martin Zapotocky^1^, Stepan Kortus^1^, Govindan Dayanithi^2^

#### ^1^Czech Academy of Sciences, Institute of Physiology, Prague, Czechia; ^2^Czech Academy of Sciences, Institute of Experimental Medicine, Prague, Czechia

##### **Correspondence**: Martin Zapotocky (zapotocky@biomed.cas.cz)

*BMC Neuroscience* 2018, **19(Suppl 2):**P84

Calcium signaling in neurons is typically initiated by Ca2+ influx through voltage-gated channels in the plasma membrane. In many neuronal types, however, it has been shown that the resulting increase of cytosolic [Ca2+] can be significantly modulated by a release/uptake of Ca2+ by intracellular stores. A protocol commonly used toanalyzesuch modulation consists of depolarizing the membrane by exposure to a high-K+ pulse and recording the resulting transient [Ca2+] response, either in control conditions or in the presence of drugs that activate/inhibit Ca2+ fluxes arising from specific intracellular stores. The detailed time course of these fluxes, however, is rarely analyzed. We have developed a combined experimental and computational method that permits to separate the principal contributing fluxes and to extract their time courses. We applied this method to freshly isolated magnocellular neurons from the rat supraoptic nucleus, with [Ca2+] kinetics recorded using Fura-2 based ratiometric imaging. Wemodeledthe [Ca2+] kinetics as resulting from depolarization-induced Ca2+ entry, Ca2+ clearance by pumps and exchangers at the plasma membrane, Ca2+ release from the endoplasmic reticulum (ER), and Ca2+ uptake by the ER. The clearance rate function was identified from experiments in which the ER fluxes were blocked. We show that in response to a series of depolarization steps, the [Ca2+] elevation can be either potentiated or attenuated, depending on the filling state of the ER. We identify the time course of the calcium-induced-calcium-release flux mediating the potentiation and of the ER re-uptake flux mediating the response attenuation. The principal functional role of the magnocellular neurons consists in the release of hormones arginine-vasopressin or oxytocin, in response to physiological stimuli. Weanalyzethe role that the usage-dependent potentiation/attenuation of the [Ca2+] response may play in the patterning of action potential bursts, which in turn control the release of vasopressin from the nerve terminals into the bloodstream.

## P85 Spatiochromatic integration by double opponent neurons in macaque V1

### Abhishek De, Gregory D. Horwitz

#### University of Washington, Department of Physiology and Biophysics, Seattle, WA, United States

##### **Correspondence**: Abhishek De (abhisd@uw.edu)

*BMC Neuroscience* 2018, **19(Suppl 2):**P85

The color of a light depends on surrounding lights. This effect is likely mediated, at least in part, by double opponent (DO) neurons in area V1. DO neurons have two characteristic properties: they are cone opponent and they have opposite color preferences in different parts of their spatial receptive field (RF). As a result, DO neurons respond maximally to color boundaries and weakly to full-field color stimuli. How these neurons integrate color signals across their RFs, however, is not well understood. For this reason, physiological and psychophysical spatial color processing are difficult to relate quantitatively. We identified V1 DO neurons in awake behaving monkeys using spike triggered averaging. We presented stimuli that activated non-overlapping regions of the RF individually or simultaneously. Using an adaptive closed-loop stimulus generator, we identified stimuli that drove the same neuronal response but differed in how strongly they activated two regions of the RF. We encountered two classes of DO neurons that were selective for either blue-yellow or red-green edges. Almost all blue-yellow and some red-green DO neurons responded to a weighted sum of color signals from the two non-overlapping regions of their RFs. Consequently, these neurons responded to chromatic contrast between the two regions of their RFs irrespective of the absolute chromaticities that defined the edge. For example, a blue-yellow DO neuron responded identically to a blue-yellow edge and to an edge between a saturated and an unsaturated blue (or yellow). A subset of red-green DO neurons combined color signals across their RFs nonlinearly. This nonlinearity may be due to complex interactions between cone opponent and cone non-opponent signals across space that have previously been identified with spike triggered covariance analysis.

## P86 A dynamic causal modeling of voltage sensitive dye imaging (VSDI-DCM) in the rodent hippocampus

### Jiyoung Kang, Kyesam Jung, Hae-Jeong Park

#### Yonsei University, College of Medicine, Seoul, Korea, Republic of

##### **Correspondence**: Hae-Jeong Park (parkhj@yonsei.ac.kr)

*BMC Neuroscience* 2018, **19(Suppl 2):**P86

Voltage-sensitive dye imaging (VSDI), which is an important neurophysiological technique to investigate the dynamics of the brain, can extract changes in the membrane potentials of large neural populations with high spatial (20 ~ 50 m) and temporal resolution (1 ~ 2 ms). The VSDI signal reflect changes in the neural population activities. However, the VSDI signal itself does not reflect connectivity across neural populations. Thus, a computational analysis is essential to estimate effective connectivity among neural populations reflected in the VSDI data. In the present study, adopting the dynamic causal modeling (DCM) [1], we developed a novel framework for effective connectivity analysis of VSDI data; VSDI-DCM. The VSDI-DCM consists of two parts; hidden neural state and VSDI observation models, which describe dynamics of the activity of neural population and transformation from hidden neural states to the VSDI signals, respectively. All model parameters in both of neural and observation models are simultaneously estimated to minimize prediction errors with observed VSDI data by Bayesian inferences. We analyzed VSDI data of the hippocampal slices of mice, downloaded from [2]. In this experiment, the temporoammonic pathway was stimulated four times with 100 ms intervals. For the for first stimulus, hyperpolarization after the stimulation was observed in CA1 region, but this inhibition was reduced for the latter stimuli. We extracted VSDI signals at the Hilus, CA1, and CA3 regions of the hippocampus. For the neural state model among these three regions, we employed a Jansen and Rit model [3], with three sub-populations (two excitatory and one inhibitory neural populations) for each region and three types of directional interactions between pairs of regions. We further added memory term to describe adaptive properties of the neural spikes. We used linear combinations of three sub-populations for observation model of VSDI signals. As a result, VSDI-DCM successfully fits VSDI signals in both wild type mouse and the epileptic Arx conditional knock-out mutant mouse. In the mutant mouse, hyperpolarization did not decrease for the consecutive stimuli. We found that adaptive parameters of the VSDI-DCM play an essential role for differentiate responses in the mutant from those of the wild type. We believe that VSDI-DCM could be used for the investigation of the mesoscale brain dynamics.

**Acknowledgement**

This research was supported by Brain Research Program through the National Research Foundation of Korea (NRF) funded by the Ministry of Science and ICT (NRF-2017M3C7A1049051).

**References**

1. Friston KJ, et al. Dynamic causal modelling. *Neuroimage* 2003, 19(4): 1273–1302.
2. Bourgeois EB, et al. A toolbox for spatiotemporal analysis of voltage-sensitive dye imaging data in brain slices. *Plos One* 2014, 9(9): e108686.
3. Jansen BH Rit VG. Electroencephalogram and visual evoked potential generation in a mathematical model of coupled cortical columns. *Biol Cybern* 1995, 73(4): 357–366.

## P87 Estimation of effective connectivity in the microcircuits of the mouse barrel cortex using dynamic causal modeling of calcium imaging

### Kyesam Jung, Jiyoung Kang, Hae-Jeong Park

#### Yonsei University, College of Medicine, Seoul, Korea, Republic of

##### **Correspondence**: Hae-Jeong Park (parkhj@yonsei.ac.kr)

*BMC Neuroscience* 2018, **19(Suppl 2):**P87

The computational modeling of the cerebral cortex may be useful to unravel the encoding mechanism for the given stimuli in the form of effective connectivity. The purpose of this study is to estimate effective connectivity among neuronal populations of the mouse barrel cortex using calcium imaging functional data.

We analyzed functional calcium imaging data of neuronal responses evoked by whisker stimulation in the mouse barrel cortex, which was downloaded from a public database (https://crcns.org/data-sets/ssc/ssc-1) [1]. Calcium image data were measured while a mouse was performing a pole localization task. To estimate the effective connectivity among five clusters of neurons, we used Dynamic causal modeling (DCM) [2] with Jansen and Rit model [3] and a calcium image time series evoked by touching whiskers. We performed waveform sorting of valid neuronal responses in the barrel cortex using principal component analysis for time series of behaviorally successful trials. We visually identified five representative calcium signal modes evoked by whisker stimulation from 6176 neurons. Using time series of these modes, we estimated effective connectivity among these modes evoked by whisker stimulation using DCM, a Bayesian method for inferring a causal architecture in the dynamic system (Fig. 1). For the DCM, we used a convolution-based model for neural state transition model, which was initially proposed by Jansen and Rit. We also made an observation model for the calcium imaging, which maps neuronal state to calcium imaging data. The calcium imaging data with a given external input were used to fit both neural state transition model and calcium imaging observation model. Among several possible models with different inputs, we chose an optimal model after Bayesian model comparison. As a result, three modes showed strongly excitatory while two modes inhibit other modes. The degree of inhibition was relatively weak compared to excitatory connectivity. The current method provides a method to explore the relationship between amplification and suppression of the canonical microcircuit in the barrel cortex using DCM for calcium imaging data set. In this framework, we are further working on establishing more precise models for calcium imaging data and validation test using simulation studies.

**Fig. 1:**
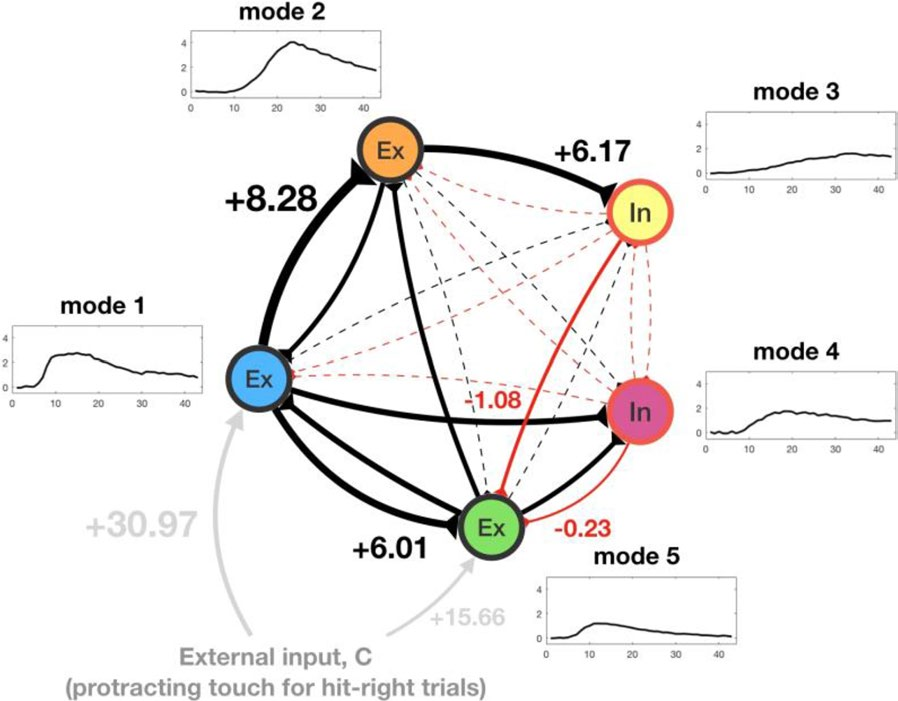
Effective connectivity among 5 modes (waveforms). Black solid and dashed lines mean excitatory effects and red solid and dashed lines mean inhibitory effects

**Acknowledgement**

This research was supported by Brain Research Program through the National Research Foundation of Korea (NRF) funded by the Ministry of Science and ICT (NRF-2017M3C7A1049051).

**References**

1. Peron, S. et al. Calcium imaging data from vibrissal S1 neurons in adult mice performing a pole localization task. CRCNS.org 2014.
2. Friston KJ, et al. Dynamic causal modelling. *Neuroimage* 2003 19(4): 1273–1302.
3. Jansen BH, Rit VG. Electroencephalogram and visual evoked potential generation in a mathematical model of coupled cortical columns. *Biol Cybern* 1995, 73(4): 357–366.

## P88 Predictions of neuronal connectivity from axonal and dendritic arbors

### Alexander Bird^1^, Lisa Deters^1^, Hermann Cuntz^2^

#### ^1^Frankfurt Institute for Advanced Studies (FIAS), Computational Neuroanatomy, Frankfurt am Main, Germany; ^2^Frankfurt Institute for Advanced Studies (FIAS) & Ernst Strüngmann Institute (ESI), Computational Neuroanatomy, Frankfurt/Main, Germany

##### **Correspondence**: Alexander Bird (alex.neurosci@gmail.com)

*BMC Neuroscience* 2018, **19(Suppl 2):**P88

The functionality of the brain depends fundamentally on the connectivity of its neurons for everything from the propagation of afferent signals to computation and memory retention. Connectivity arises from the apposition of complex branched axonal and dendritic arbors which each display a diverse array of forms, both within and between neuronal classes. Despite this complexity, neurons of different classes have been observed to form synapses in highly specific ways [1], leading to potentially highly structured connectivity motifs within neuronal networks. Whilst the large-scale EM studies necessary to definitively constrain synaptic connectivity remain prohibitively slow [2]and viral synaptic tracing is limited to small neuron numbers [3], putative synaptic locations from the close juxtaposition of dendrite and axon are more readily measured [4] and provide the potential set of all possible synaptic contacts; the backbone upon which neuronal activity can fine tune connectivity. It has been shown that much of the specificity in putative connectivity can be explained by a detailed analysis of the statistical overlap of different axonal and dendritic arbors [5] in a manner analogous to Peters’ rule where synapses are assumed to form uniformly where possible [6]. However such analyses rely on full neuronal reconstructions with large numbers of parameters and are difficult to apply intuitively to microcircuits. We have investigated the number of putative synapses that form between artificial arbors generated using a generalised minimum-spanning tree algorithm that mirrors the structure of real neurons [7]. We have found that the number of putative synaptic contacts depends linearly on just four properties of the arbors: the volume of the region where dendrite and axon overlap, the length of the axonal and dendritic arbors within this region, and the maximum dendritic spine length at which synaptic contacts can form. The relationship between these four parameters and the estimated synapse number can be expressed as a single equation (adapted from results in [8] and [9]) and accurately models the number of putative synapses between reconstructed cortical neurons [10]. We have additionally shown that this relationship is specific to typical dendritic and axonal structures as morphologies that resemble knock-out mutants with pathologically clustered dendrites do not fit our predictions. Other deviations from the predictions of our study could provide insights into the degree of targeting in neurite growth processes in different brain regions as more detailed connectome data becomes available. Overall our work provides an intuitive way to estimate the putative synaptic connectivity of microcircuits, greatly simplifying the parameters necessary for analytical and numerical studies of biophysically detailed neuronal networks.

**References**

1. Xiang X, Shen S, Cadwell CR, et al. *Principles of connectivity among morphologically defined cell types in adult neocortex*. Science (New York, NY). 2015, 350(6264):aac9462. 10.1126/science.aac9462.
2. Helmstaedter, M. Cellular-resolution connectomics: challenges of dense neural circuit reconstruction. *Nature methods* 2013, 10(6), 501.
3. Wall NR, De La Parra M, Callaway EM, Kreitzer AC. Differential innervation of direct- and indirect-pathway striatal projection neurons. *Neuron* 2013, 79(2):347–360. 10.1016/j.neuron.2013.05.014.
4. Markram H, Lübke J, Frotscher M, Roth A, Sakmann B. Physiology and anatomy of synaptic connections between thick tufted pyramidal neurones in the developing rat neocortex. *The Journal of Physiology* 1997, 500(Pt 2):409–440.
5. Hill SL, Wang Y, Riachi I, Schürmann F, Markram H. Statistical connectivity provides a sufficient foundation for specific functional connectivity in neocortical neural microcircuits. *Proceedings of the National Academy of Sciences of the United States of America* 2012, 109(42):E2885-E2894. 10.1073/pnas.1202128109.
6. Braitenberg, V., & Schüz, A. (2013). *Cortex: statistics and geometry of neuronal connectivity*. Springer Science & Business Media.
7. Cuntz H, Forstner F, Borst A, Häusser M. One Rule to Grow Them All: A General Theory of Neuronal Branching and Its Practical Application. Morrison A, ed. *PLoS Computational Biology* 2010, 6(8):e1000877. 10.1371/journal.pcbi.1000877.
8. Liley, D. T., & Wright, J. J. Intracortical connectivity of pyramidal and stellate cells: estimates of synaptic densities and coupling symmetry. *Network: Computation in Neural Systems* 1994, 5(2), 175–189.
9. Chklovskii, D. B. Synaptic connectivity and neuronal morphology: two sides of the same coin. *Neuron* 2004, 43(5), 609–617.
10. Ascoli, G. A., Donohue, D. E., & Halavi, M. NeuroMorpho. Org: a central resource for neuronal morphologies. *Journal of Neuroscience* 2007, 27(35), 9247–9251.

## P89 Optimal wiring imposes fixed cortical hypercolumn sizes

### Marvin Weigand^1^, Hermann Cuntz^2^

#### ^1^Frankfurt Institute for Advanced Studies (FIAS), Frankfurt, Germany; ^2^Frankfurt Institute for Advanced Studies (FIAS) & Ernst Strüngmann Institute (ESI), Computational Neuroanatomy, Frankfurt/Main, Germany

##### **Correspondence**: Marvin Weigand (mweigand@fias.uni-frankfurt.de)

*BMC Neuroscience* 2018, **19(Suppl 2):**P89

The concept of a hypercolumn is used to subdivide discriminable patterns of continuously shifting feature preferences into discrete topographical units [1–3]. In the visual cortex such hypercolumns consist for example of repeating pinwheel patterns [4–6] and seem to follow a universal design principle across mammalian species [7] because the number of pinwheels per hypercolumn area is constant near pi [8]. We find using curated biological data that this constant relationship is a general consequence of a fixed number of neurons per hypercolumn and that differences in absolute pinwheel densities are a mere consequence of differences in the neuronal density. Low neuronal densities would therefore result in large hypercolumns and vice versa. In agreement with previous results [9], our analysis of the characteristic orientation preference hypercolumns in the primary visual cortex yields a constant number of ~ 30,000 neurons per pinwheel and defines a minimum of ~ 300 pinwheels below which organisms lack hypercolumns altogether. Using a computational model based on optimal wiring principles we confirm our empirical results by showing that similarly structured hypercolumns appear with fixed cell numbers independently of the overall network size. Furthermore we show that a fixed hypercolumn size is compatible with the absence of hypercolumns in rodent species. Overall, our results provide further evidence for a universal design principle in the visual cortex across mammalian species.

**References**

1. Mountcastle, V. B. The columnar organization of the neocortex. *Brain a J*. *Neurol*.1997, 120, 701–722.
2. Kaas, J. H. Evolution of columns, modules, and domains in the neocortex of primates.*Proc*. *Natl*. *Acad*. *Sci*.2012 109, 10655–10660.
3. Horton JC, Adams DL. The cortical column: a structure without a function.*Philos*. *Trans*. *R*. *Soc*. *B Biol*. *Sci*. 2005, 360, 837–862.
4. Hubel DH, Wiesel TN. Sequence regularity and geometry of orientation columns in the monkey striate cortex. *J*. *Comp*. *Neurol*.1974, 158, 267–293.
5. Blasdel GG, Salama G. Voltage-sensitive dyes reveal a modular organization in monkey striate cortex. *Nature* 1986, 321, 579–585.
6. Ohki K,.et al. Highly ordered arrangement of single neurons in orientation pinwheels. *Nature* 2006, 442, 925–928.
7. Weigand M, Sartori F, Cuntz H. Universal transition from unstructured to structured neural maps.*Proc*. *Natl*. *Acad*. *Sci*. 2017, 114, E4057–E4064.
8. Kaschube M, et al. Universality in the evolution of orientation columns in the visual cortex. *Science* 2010, 330, 1113–1116.
9. Srinivasan S, Carlo CN, Stevens CF. Predicting visual acuity from the structure of visual cortex. *Proc*. *Natl*. *Acad*. *Sci*. 2015, 112, 7815–7820.

## P90 Dendritic branching statistics explained from minimal wiring constraints

### Felix Effenberger, Hermann Cuntz

#### Frankfurt Institute for Advanced Studies (FIAS) & Ernst Strüngmann Institute (ESI), Computational Neuroanatomy, Frankfurt/Main, Germany

##### **Correspondence**: Hermann Cuntz (hermann.neuro@gmail.com)

*BMC Neuroscience* 2018, **19(Suppl 2):**P90

In neural circuits, neurons send out tree-shaped dendritic structures to collect inputs from their presynaptic partners. Different cell types are visually identifiable by the characteristic shapes of their dendrites [4, 5] and these also critically affect their respective computations. A large number of branching statistics have been proposed as objective criteria to capture differences between cell types and to distinguish disease or mutation phenotypes [1]. Yet, as we show here, most of those widely used statistics show trivial correlations that are essentially entirely explained by optimal wiring considerations [6], consistent with their poor power for sorting dendritic tree shapes into their respective cell types [2]. Using a simple maximum entropy model based on minimum spanning trees, we were able to reproduce almost all relationships between the commonly used branching statistics. To verify our model we studied a large set of real dendritic trees, covering a multitude of different cell types, species, developmental stages and brain regions [3]. Our study not only gives a comprehensive overview of all commonly used statistics and emphasizes the need for more powerful branching statistics, but more generally indicates a potential randomness of dendritic arborizations in the brain constrained only by optimal wiring considerations and the space they innervate. The model we propose can furthermore serve as a basis to test the power of yet to be invented branching statistics and is also likely useful to study other branching structures found in nature, such as river networks, botanical trees, and blood vessel structures.

**References**

1. Scorcioni R, Polavaram S, Ascoli GA. L-Measure: a web-accessible tool for the analysis, comparison and search of digital reconstructions of neuronal morphologies. *Nature Protocols* 2008, 3(5), 866.
2. Polavaram S, Gillette TA, Parekh R, Ascoli GA. Statistical analysis and data mining of digital reconstructions of dendritic morphologies. *Frontiers in Neuroanatomy* 2014, 8, 138.
3. Ascoli GA, Donohue DE, Halavi M. NeuroMorpho. Org: a central resource for neuronal morphologies. *Journal of Neuroscience* 2007, 27(35), 9247.
4. Ascoli GA, Alonso-Nanclares L, Anderson SA, et al. Petilla terminology: nomenclature of features of GABAergic interneurons of the cerebral cortex. *Nature Reviews Neuroscience* 2008, 9(7), 557.
5. Cuntz H, Forstner F, Haag J, Borst A. The morphological identity of insect dendrites. *PLoS Computational Biology* 2008, 4(12), e1000251.
6. Cuntz H, Forstner F, Borst A, Häusser M. One rule to grow them all: a general theory of neuronal branching and its practical application. *PLoS Computational Biology* 2010, 6(8), e1000877.

## P91 Dissecting the structure and function relationship in Drosophila dendrite development with the help of computational modelling

### André Castro^1,2^, Lothar Baltruschat^3^, Tomke Stuerner^3^, Gaia Tavosanis^3^, Hermann Cuntz^4^

#### ^1^Ernst Strüngmann Institute (ESI) for Neuroscience in Cooperation with Max Planck Society, Frankfurt am Main, Portugal; ^2^Frankfurt Institute for Advanced Studies (FIAS); ^3^German Center for Neurodegenerative Diseases (DZNE), Dendrite Differentiation Unit, Bonn, Germany; ^4^Frankfurt Institute for Advanced Studies (FIAS) & Ernst Strüngmann Institute (ESI), Computational Neuroanatomy, Frankfurt/Main, Germany

##### **Correspondence**: André Castro (castro.neuro@protonmail.com)

*BMC Neuroscience* 2018, **19(Suppl 2):**P91

Dendritic growth is the process that ultimately leads to cell type specific neuronal morphologies and contributes to building mature neural circuits, shaping their computational properties. Dendritic tree morphology is strongly constrained by optimal wiring considerationsand by functional properties relevant to behaviour. However, the rules controlling the fine regulation of branch outgrowth, pruning and stabilisation that leads to the mature arbour elaboration remain largely unknown. In this work we study the growth phases of ventral Class I dendritic arborisation (da) neurons of the *Drosophila melanogaster* larva peripheral nervous system at a high temporal resolution that allows resolving the fine elements that compose the growth process. The Class I da neurons, which are proprioceptive and respond to contractions in the larva body during crawling, do not obviously gain from satisfying optimal wiring constraints. Therefore, we use this system to study how their specific functional requirements may be combined with optimal wiring constraints during the developmental growth process that leads to the dendritic morphologies of these cells. Genetic manipulation of the sensory neuron’s shape interferes with their sensory function and disrupts crawling behaviour, suggesting that the feedback of information about body movement depends on precise dendritic morphology. Hence, we probed the contribution of the class I ventral cell’s dendrite characteristic comb-like geometry in sampling the mechanosensory inputs arising from the contraction of body wall during crawling behaviour, by recording high resolution calcium imaging in freely crawling larvae. Using these recordings, we show strong correlations between calcium signal change in the deformed comb-like dendritic branches caused by the contraction of the body wall in a series of periodic strides during forward and reverse crawling. We then utilized genetically encoded green fluorescent protein markers for ventral Class I da cells, and recorded high temporal resolution non-invasive, in vivo time-lapse microscopy images of dendrite arbour morphogenesis in the embryo and its maturation in the larva. The time-lapse data enabled us to constrain computational growth models that clearly defined the different development stages of dendritic pattern formation. Furthermore, they revealed how this individual type of neurons controls branching to achieve its mature shape while respecting minimal wiring constraints. Our findings unveil how single neurons can develop specialised dendrite patterns that support a well-defined function while minimizing wiring costs associated with their dendritic trees, shedding light on general principles of structure–function emergence in single neurons.

## P92 Dimensionality reduction of brain signals of rats by Spectral Principal Component Analysis (SPCA)

### Altyn Zhelambayeva^1^, Hernando Ombao^2^

#### ^1^Nazarbayev University, Department of Computer Science & Biological Sciences, Astana, Kazakhstan; ^2^King Abdullah University of Science and Technology, Statistics Program, Thuwal, Saudi Arabia

##### **Correspondence**: Altyn Zhelambayeva (altyn.zhelambayeva@nu.edu.kz)

*BMC Neuroscience* 2018, **19(Suppl 2):**P92

A stroke occurs when the blood supply to the brain is either halted or significantly reduced. The study of the effectiveness of brain stimulation techniques is of growing interest in modern stroke research. The major challenges of the exploratory analysis of brain signals are high dimensionality and massive size of the data. Dimensionality reduction is necessary to facilitate computation and interpretation of the results. One of the techniques for dimensionality reduction introduced in [1] and [2] is Spectral Principal Component Analysis (SPCA), which accounts the oscillatory patterns in these signals. In contrast to the conventional Principal Component Analysis (PCA) which is the instantaneous linear mixture of the signal, SPCA is defined as a linear convolution of the signal at all lags. In this project, we study LFP signals recorded from 32 channels located on rats’ brain cortices for 1 h before and 5 h after a stroke. A stroke was artificially induced by occluding the medial cerebral artery (MCA). We extract signal summaries from these high-dimensional data by SPCA to study the difference in the brain network among rats that received early stimulation post-stroke and those that did not receive stimulation. Also, the LFP signal at each epoch was regressed against the first two spectral principal components to examine the temporal correlation of the coefficient that corresponds to the loading of signal summary 1. We studied the spatial correlation of the regions of electrodes placement by combining channels into 4 clusters using hierarchical clustering algorithm. The number of clusters was determined by the inspection of dendograms across all epochs. We extracted signal summaries of each cluster for every epoch and computed their cross-correlation. The results suggest that post-stroke LFP signals of the non-stimulated rat are highly synchronized as opposed to the rat whose whisker was stimulated (Fig. 1). These results are consistent with the brain signals of sham (non-occlusion) rats.

**Fig. 1:**
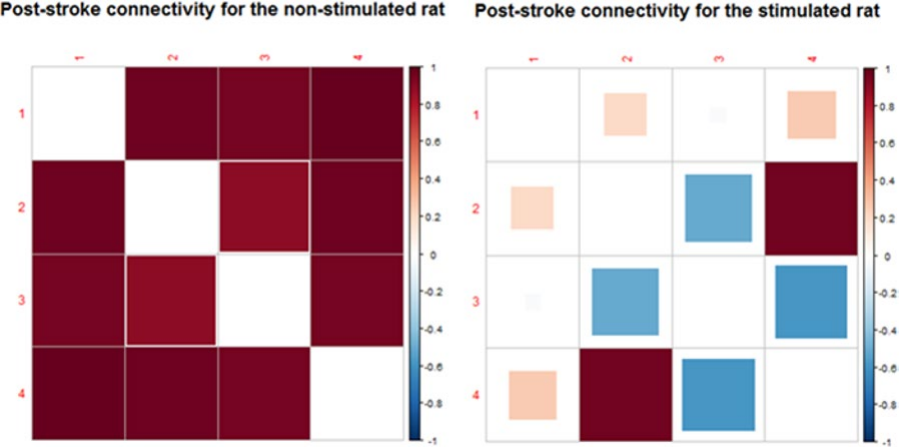
Connectivity between different clusters post-stroke. Left is the non-stimulated rat. Right is the stimulated rat

## P94 Advancing computational studies of the nervous system: Publishing models not paper descriptions of models

### James Bower^1^, David Beeman^2^, Hugo Cornelis^3^

#### ^1^Southern Oregon University, Department of Biology, Ashland, OR, United States; ^2^University of Colorado, Department of Electrical, Computer and Energy Engineering, boulder, CO, United States; ^3^Neurospaces Development GCV, Martelarenlaan, Belgium

##### **Correspondence**: James Bower (bowerj@sou.edu)

*BMC Neuroscience* 2018, **19(Suppl 2):**P94

1440 is the date generally accepted for the invention of movable type printing (i.e. the printing press) by Johannes Gutenberg. Don Quixote, generally regarded as the first novel with modern structure was published by Cervantes in 1605, 165 years later. It was another 60 years (1665) before the Secretary of the Royal Society of London organized the first periodical focused exclusively on Science. The full adoption and best use of a new technology clearly takes time. Originally titled Philosophical Transactions: Giving some Account of the present Undertakings, Studies, and Labours of the Ingenious in many considerable parts of the World” the journals founder, Henry Oldenburg, defined the purpose of the new journal at the outset to support “registration” (date stamping and provenance), “certification” (peer review), “archiving” and of particular importance “dissemination”. Referring to the importance of dissemination in particular, Oldenburg wrote to Robert Boyle in 1664, that through the journal: “…all ingenious men will thereby be incouraged to impact their knowledge and discoverys”. The question that we address in this presentation is whether and how the digitalization of scientific (and human) communication could and should re-implement these original objectives for scientific publishing. Specifically, we will argue that the more than 350 year old form of communication currently represented by off-line and now on-line scientific journals is particularly inappropriate and limiting for computational neuroscience. Having worked on this problem through the GENESIS simulation platform for 35 years (Fig. 1), we will present a specific new form of publication workflow and associated tools for the review, archiving and dissemination of computational models through the process of publishing the models themselves, not paper versions of the models. Perhaps most importantly, this new workflow intrinsically considers and supports collaboration in a way that the printing press never could and current journals can not. Our presentation will attempt to make clear that such a change is not simply a digital nicety, but instead will likely be crucial for advancing our understanding of nervous systems by accelerating the convergence of concepts and theories in computational neuroscience. As one more historical note, while Philosophical Transactions was established in 1665, it wasn’t accepted as the official journal of the Royal Society until 1752 when the name was changed to the “Transactions of the Royal Society”. The question this presentation will pose to consider is whether it will take less than 225 years from the establishment of the internet to launch a new form of scientific journal and whether it will take less than 87 years for the scientific community to accept that form?

**Fig. 1:**
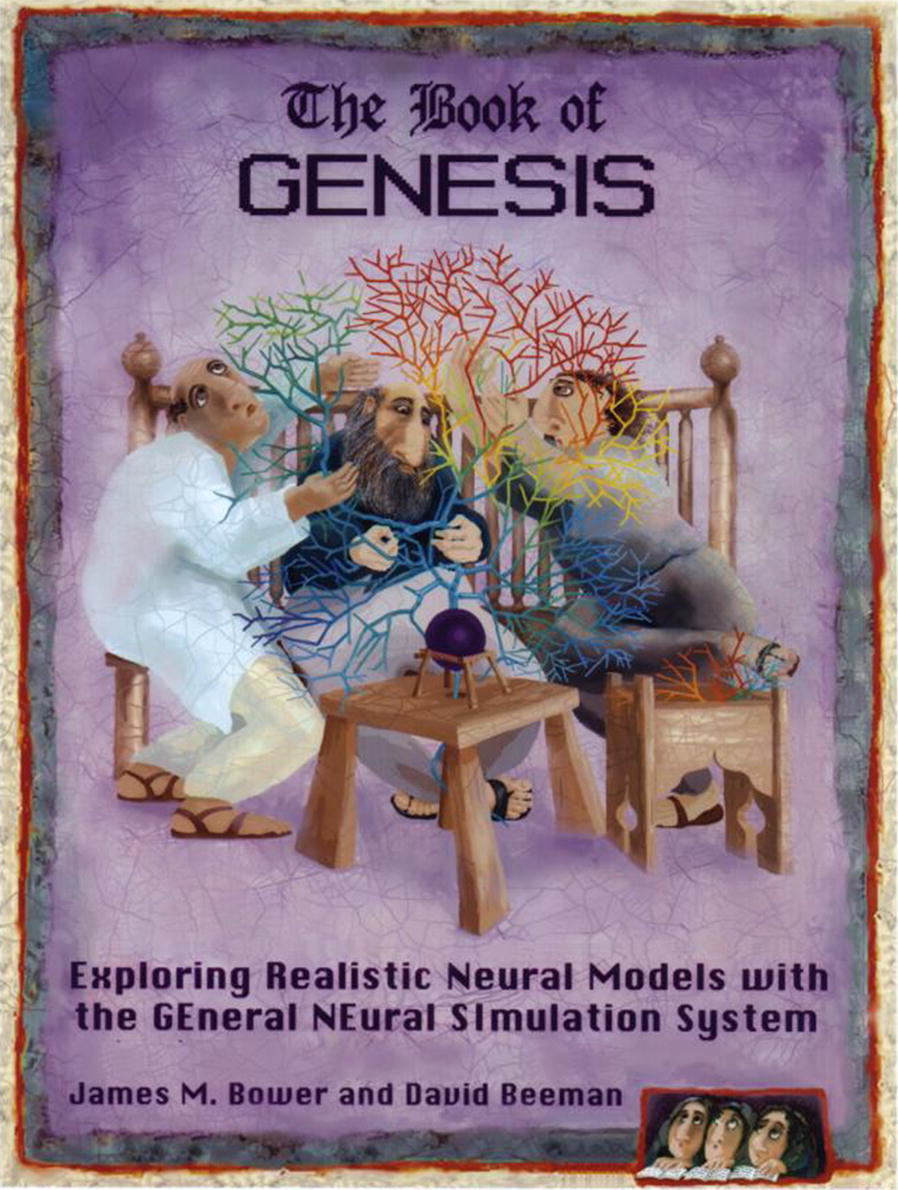
Cover for the first Book of GENESIS—representing the importance and hope for collaborative science

## P95 Disentangling diverse patterns of synaptic efficacy in vivo and their causes

### Abed Ghanbari^1^, Naixin Ren^2^, Christian Keine^3^, Carl Stoelzel^2^, Bernhard Englitz^4^, Harvey Swadlow^2^, Ian H. Stevenson^2^

#### ^1^University of Connecticut, Department of Biomedical Engineering, Storrs, CT, United States; ^2^University of Connecticut, Department of Psychological Sciences, Storrs, CT, United States; ^3^Carver College of Medicine & University of Iowa, Department of Anatomy and Cell Biology, IA, United States; ^4^Radboud University & Donders Institute for Brain, Cognition and Behaviour & Department of Neurophysiology, Netherlands

##### **Correspondence**: Abed Ghanbari (abed.ghanbari@uconn.edu)

*BMC Neuroscience* 2018, **19(Suppl 2):**P95

Short-term synaptic plasticity (STP) causes the effect of presynaptic spikes on a postsynaptic neuron to vary on timescales ranging from a few milliseconds to a few seconds. STP has been extensively studied in vitro by stimulating a presynaptic input with pulses of different frequencies and observing depression or facilitation in the postsynaptic potentials or currents. These studies have shown that the type and timescale of STP varies by cell type and brain region. However, since recording postsynaptic potentials (PSP) or currents (PSC) in vivo is challenging, STP has not been fully characterized in awake, behaving animals. Here rather than observing PSP/PSCs directly, we model how presynaptic spikes alter postsynaptic spiking and infer STP parameters from spike observations alone. In particular, we model the short-term changes in the probability of a postsynaptic spike following a presynaptic spike—the synaptic efficacy. Previous work has argued that, in depressing synapses, this probability or efficacy is larger when presynaptic spikes are preceded by long interspike intervals (ISIs), and in facilitating synapses efficacy is larger for short intervals. However, in practice, the observed correlation between pre- and postsynaptic spiking is a mixture of multiple underlying phenomena. Here we develop a model-based approach for decomposing these short-term changes into four components: (1) short-term synaptic plasticity, (2) integration of PSPs, (3) history effects, and (4) slow common inputs. The observed spike probability depends on each of these factors as well as the synaptic strength itself and the distribution of presynaptic spike times. We developed an extension of a typical generalized linear model (GLM) to use only pre- and postsynaptic spike observations. This method allows us to characterize short-term synaptic dynamics of a wide range of synaptic behaviors in vivo. The estimated synaptic parameters as well as plasticity parameters could be compared with in vitro measurements. To validate our model, we examined its performance for four putative synapses using only pre- and postsynaptic spike observations. We find that lateral-geniculate nucleus-to-visual cortex (LGN-V1) data is consistent with short-term synaptic depression where postsynaptic spike probability increases at long presynaptic ISIs (which allow for recovery from the depression). Data from the auditory nerve-to-spherical bushy cells (ANF-SBC) synapses, on the other hand, is consistent with short-term synaptic facilitation, and spike history causes decreased postsynaptic spiking at short presynaptic ISIs. There is a wide range of efficacy patterns in the multi-electrode hippocampus (HC) data, but, in many cases, common input from theta oscillations has an impact on the observed efficacy. Lastly, a pair within thalamus shows depressing pattern similar to LGN-V1 connection with stronger integration. These results demonstrate how short-term synaptic efficacy reflects a combination of many factors, and interactions between these factors give rise to a wide diversity of effects of presynaptic spikes on postsynaptic spiking. As the number of simultaneously recorded neurons increases, this approach is likely to be useful for characterizing STP in multi-electrode array recordings as well as studying how differences in STP affect postsynaptic spiking.

## P96 Optimizing stimulation protocols for prosthetic vision based on retinal anatomy

### Michael Beyeler^1^, Ariel Rokem^1^, Devyani Nanduri^2^, James D. Weiland^3^, Geoffrey M. Boynton^4^, Ione Fine^4^

#### ^1^University of Washington, eScience Institute, Seattle, WA, United States; ^2^University of Southern California, Biomedical Engineering, Los Angeles, CA, CA, United States; ^3^University of Michigan, Biomedical Engineering, Ann Arbor, MI, MI, United States; ^4^University of Washington, Psychology, Seattle, WA, WA, United States

##### **Correspondence**: Michael Beyeler (mbeyeler@uw.edu)

*BMC Neuroscience* 2018, **19(Suppl 2):**P96

By 2020 roughly 200 million people worldwide will suffer from degenerative retinal diseases. While a variety of sight restoration technologies are being developed, retinal neuroprostheses (‘bionic eyes’) are the only devices with FDA approval. These devices aim to restore functional vision by electrically stimulating remaining cells in the retina, analogous to cochlear implants. However, these devices stimulate retinal axon fibers as well as cell bodies: this leads to elongated and poorly localized percepts that severely limit the quality of the generated visual experience1. We previously developed a computational model that describes these distortions and accurately predicts a patient’s perceptual experience for any pattern of electrical stimulation 3–5. However, improving the design of neuroprosthetic devices will require a solution of the inverse problem: What is the optimal stimulation protocol that elicits a desired visual percept? To answer this, we used our model to generate synthetic data that predicted elicited percepts in an Argus II epiretinal prosthesis patient. These synthetic percepts were used as features in a regularized regression optimized to find the stimulation protocols that would minimize perceptual distortions of Snellen letters. Compared to conventional protocols currently used in patients, in which each electrode is stimulated with an amplitude that is linearly related to the luminance of the corresponding location in the visual field, the percepts produced with the optimized stimulation protocols confer a potential substantial advantage, both in terms of expected visual acuity and overall delivered charge: Stimulation protocols proposed by the algorithm only sparsely activated the electrode array and compensated for the perceptual distortions thought to be caused by axonal stimulation. Future work will include more sophisticated machine learning methods that can compensate for spatiotemporal distortions across a wider range of implants.

## P97 Effect of use dependent plasticity on information transfer at hippocampal synapses

### Emily Stone^1^, Elham Bayat-Mokhtari^1^, J. Josh Lawrence^2^

#### ^1^University of Montana, Department of Mathematical Sciences, Missoula, MT, United States; ^2^Texas Tech University Health Sciences Center, Department of Pharmacology and Neuroscience, Lubbock, TX, United States

##### **Correspondence**: Emily Stone (stone@mso.umt.edu)

*BMC Neuroscience* 2018, **19(Suppl 2):**P97

Simple models of short term synaptic plasticity that incorporate facilitation and/or depression have been created in abundance for different synapse types and circumstances. The analysis of these models has included computing mutual information between a stochastic input spike train to the presynaptic synapse, and some sort of representation of the postsynaptic response. While this approach has proven useful in many contexts, for the purpose of determining the type of process underlying a stochastic output train, it ignores the ordering of the responses, leaving an important characterizing feature on the table. In this work we use a broader class of information measures on output only, and specifically construct hidden Markov models (known as epsilon machines or causal state models) to differentiate between synapse type, and classify the complexity of the process. We find that the machines allow us to differentiate between processes that otherwise have similar output distributions. We are also able to understand these differences in terms of the dynamics of the model used to create the output response, bringing the analysis full circle. Hence this technique provides a complimentary description of the synaptic filtering process, and potentially expands the interpretation of future experimental results.

## P98 A neural network model of complementary learning systems

### Mika Jain, Jack Lindsey

#### Stanford University, Departments of Physics, Computer Sciences & Biology, NYC, NY, United States

##### **Correspondence**: Mika Jain (mikasarkinjain@gmail.com)

*BMC Neuroscience* 2018, **19(Suppl 2):**P98

We introduce a computational model capturing the high-level features of the complementary learning systems (CLS) framework. In particular, we model the integration of episodic memory with statistical learning in an end-to-end trainable neural network architecture. We model episodic memory with a nonparametric module which can retrieve past observations in response to a given observation, and statistical learning with a parametric module which performs inference on the given observation. We demonstrate on vision and control tasks that our model is able to leverage the respective advantages of nonparametric and parametric learning strategies, and that its behavior aligns with a variety of behavioral and neural data. In particular, our model performs consistently with results indicating that episodic memory systems in the hippocampus aid early learning and transfer generalization. We also find qualitative results consistent with findings that neural traces of memories of similar events converge over time. Furthermore, without explicit instruction or incentive, the behavior of our model naturally aligns with results suggesting that the usage of episodic systems wanes over the course of learning. These results suggest that key features of the CLS framework emerge in a task-optimized model containing statistical and episodic learning components, supporting several hypotheses of the framework.

## P99 Separation of hemodynamic signals from GCaMP fluorescence measured with widefield imaging

### Matt Valley^1^, Michael Moore^2^, Jun Zhuang^1^, Natalia Mesa^1^, Mark Reimers^2^, Jack Waters^1^

#### ^1^Allen Institute for Brain Science, Modelling, Analysis and Theory, Seattle, WA, United States; ^2^Michigan State University, Department of Neuroscience, East Lansing, MI, United States

##### **Correspondence**: Matt Valley (mattv@alleninstitute.org)

*BMC Neuroscience* 2018, **19(Suppl 2):**P99

Over the last few years there has been a dramatic increase in the number of mouse lines with GCaMP expression throughout much of neocortex offering the opportunity to image cortical activity using calcium indicators with high signal-to-noise and genetically-targeted expression. Widefield calcium imaging has emerged as a valuable tool to measure meso-scale brain calcium dynamics using these new indicators. Its strengths, a large field-of-view with a high sampling rate, permit experiments that simultaneously image activity from the entirety of dorsal cortex, revealing a dynamic systems-level representation of cortical interactions during mouse behavior. However, the interpretation of these widefield data is complicated by the substantial mixing of calcium signals with hemodynamics. Here, we present a new approach to demixing hemodynamic signals from calcium activity using the strategy that signals observed in GFP mice should be removed from GCaMP mice. We motivate this approach by showing that a linearization of the Beer-Lambert equation—where in our system four pathlength parameters would typically be estimated using simulations—can be exactly captured by a linear model with two variables. Using this insight, we gather data from awake mice expressing GFP or GCaMP using a multi-spectral widefield macroscope that alternates two backscatter measurements (575 nm and 630 nm) simultaneously with a fluorescence measurement. With two backscatter measurements, we train spatially-detailed regression models to find what amount of fluorescent variance in GFP mice can be explained by the backscatter data at each pixel. We generalize these primary models across mice using a meta-model trained on shared features of the data to estimate spatial detail unique to each animal. We quantify the success of this approach demixing hemodynamic variance in several commonly used Cre driver lines with different cortical laminar expression patterns and show in what conditions this approach offers advantages over non-spatially-detailed demixing. We also demix GCaMP data and show that we can remove stereotyped hemodynamic responses to visually-evoked activity (Fig. 1). This method offers a means to quantify and demix hemodynamic contamination at every pixel in a widefield movie, and is an essential step towards converting fluorescence measurements to calcium activity.

**Fig. 1:**
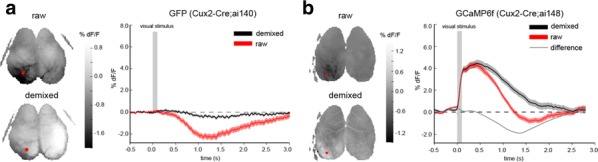
Demixing hemodynamics in GFP and GCaMP mice. A) Left, map of average response (averaged 1.5–2.0 s post stimulus) to a 100 ms flashed visual stimuli given to the right eye of a GFP mouse (Cux2-IRES2-CreERT2;Ai140, left). Right, time-course of raw trace (red) and a trace demixed using the meta model (black) extracted from a pixel near V1 cortex (red dot, left). B) Map of response (averaged 1.5–2.0 s post stimulus) of a visual response in a GCaMP6f mouse (Cux2-IRES2-CreERT2;Ai148). Time course of responses plotted as in A, with their difference (raw-demixed) in gray

## P100 Brains on board: Neuromorphic control of flying robots

### Thomas Nowotny^1^, Eleni Vasilaki^2^, Andrew O. Philippides^1^, Paul R. Graham^3^, Lars Chittka^4^, Mikko Juusola^5^, James A. R. Marshall^2^

#### ^1^University of Sussex, School of Engineering and Informatics, Brighton, United Kingdom; ^2^University of Sheffield, Department of Computer Sciences, Sheffield, United Kingdom; ^3^University of Sussex, School of Life Sciences, Brighton, United Kingdom; ^4^Queen Mary, University of London, School of Biological & Chemical Sciences, London, United Kingdom; ^5^University of Sheffield, Department of Biomedical Science, Sheffield, United Kingdom

##### **Correspondence**: Thomas Nowotny (t.nowotny@sussex.ac.uk)

*BMC Neuroscience* 2018, **19(Suppl 2):**P100

What if we could design an autonomous flying robot with the navigational and learning abilities of a honeybee?

In the ‘Brains on Board’ project we have brought together experts in computational neuroscience, bio-inspired robotics, animal behaviour and neurophysiology from three UK universities to realize this vision. Autonomous control of mobile robots requires robustness to environmental and sensory uncertainty, and the flexibility to deal with novel environments and scenarios. Animals solve these problems by having flexible brains capable of unsupervised pattern detection and learning. Even ‘small’-brained animals like bees exhibit sophisticated learning and navigation abilities using very efficient brains of only up to 1 million neurons, 100,000 times fewer than in a human brain. Crucially, these mini-brains nevertheless support high levels of multitasking and they are adaptable, within the lifetime of an individual, to completely novel scenarios; this is in marked contrast to typical control engineering solutions. In the Brains on Board project we fuse computational and experimental neuroscience to develop a ground-breaking new class of highly efficient robot controllers, able to exhibit adaptive behaviour while running on powerful yet lightweight accelerated embedded systems hardware such as NVIDIA’s Jetson TX2 and Movidius’ Myriad II systems. On this poster we present an overview of the Brains on Board project and discuss preliminary results:

1. We have developed the SpineCreator-SpineML-GeNN toolchain to make best use of embedded GPU accelerators for autonomous robots and obtain sufficient compute power to run bee brain simulations in real time on a flying robot.

2. We have completed a first proof of concept system, based on a Parrot bebop II with NVIDIA Jetson TX1 “backpack” (see Fig. 1), that has flown autonomously and avoided walls using a simulation of the bee’s visual system.

**Fig. 1:**
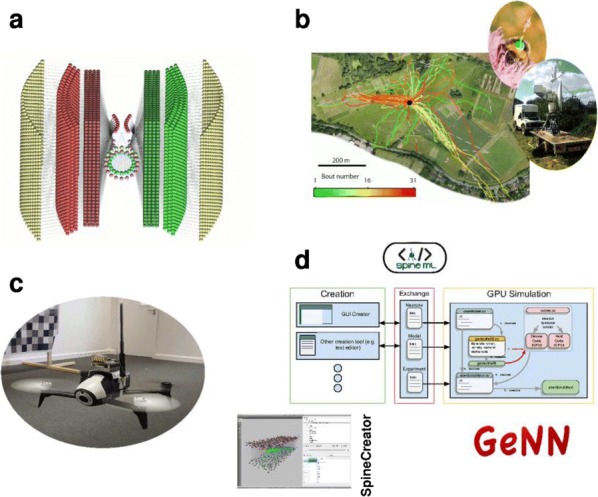
Aspects of the Brains on Board project: a) Computational model of the central complex of bees, b) radar tracked flight trajectories of bees, inset: bumble bee with harmonic radar tracking antenna, harmonic radar instrument, c) Parrot bebop quadcopter with Jetson TX1 “backpack”, c) Illustration of the SpineCreator-SpineML-GeNN toolchain

3. We have created a bee virtual reality system for closed-loop behavioural experiments with walking bees.

4. We have obtained large quantities of 2D bee flightpath data through radar tracking and a 3D harmonic radar tracking system is close to completion.

5. We have developed novel computational neuroscience models for reward estimation in bees and fruit flies, models of the visual system and oculo-motor reflex, and a model of the central complex related to navigation.

**Acknowledgements**

The Brains on Board project is financed by the Engineering and Physical Sciences Research council (EPSRC), grant EP/P.006094/1.

## P101 Gamma genesis and phase-amplitude coupling in a model of striatal fast-spiking interneurons

### Sebastien Naze, James Humble, James Kozloski

#### IBM TJ Watson Research Center, Multiscale Brain Modeling and Neural Tissue Simulation, Yorktown Heights, NY, United States

##### **Correspondence**: Sebastien Naze (sebastien.naze@gmail.com)

*BMC Neuroscience* 2018, **19(Suppl 2):**P101

Abnormal gamma band power across cortex and striatum is observed in Huntington’s disease (HD) in both patients and animal models. The origin of this phenomenon is not well understood, nor is its functional relevance to disease pathology. To address the former, we developed three hypotheses and a computational model for fast-spiking interneurons (FSIs) that was based on observations from mice striatal anatomy and physiology. First, we considered if abnormal cortical activity alone can account for an increased gamma power recorded in the striatum, with the common assumption that FSIs are responsible for such high frequency oscillations. Second, we asked if a reorganization of corticostriatal projections in terms of driving strength can account for increased gamma in the striatum. Third, we considered if changes within the striatal micro-circuit can explain the increase in gamma power therein. Changes of peak gamma frequency and power ratio were readily reproduced by our computational model, accounting for several experimental findings reported in the literature. Our results also suggest that cortical changes alone are unlikely to account for the full range of phenomena observed in striatum, and that instead both a reorganization of corticostriatal drive and specific population changes to intra-striatal synaptic coupling are present in HD.

## P102 Brain activity in a spherical geometry via neural field theory

### Kamrun Mukta, Xiao Gao, Peter Robinson, James MacLaurin

#### The University of Sydney, School of Physics, NSW, Sydney, Australia

##### **Correspondence**: Kamrun Mukta (kamrun.mukta@sydney.edu.au)

*BMC Neuroscience* 2018, **19(Suppl 2):**P102

Corticothalamic neural field theory (NFT) has successfully explained a wide variety of phenomena, ranging from EEG spectra and evoked potentials to nonlinear phenomena such as seizures and Parkinsonian oscillations. Measures such as spectra, correlation and coherence functions are widely used to probe cognitive events and information processing experimentally. Most recently, prior work showed that the eigenmodes of a single brain hemisphere are close analogs of spherical harmonics. They are also the building blocks for bihemispheric modes, whose structure and symmetry properties explain many features of resting state and task-related activity. This eigenmode expansion is of use because it helps us understand the dynamics of the brain’s activity in terms of its natural modes. Here, corticothalamic NFT is analyzed on a sphere and used to derive the transfer function, the power spectrum, the correlation function, and the cross spectrum in terms of spherical harmonics. The results are analyzed and compared with planar NFT in both finite and infinite geometries. The results of spherical and finite-planar geometries converge to the infinite-planar geometry in the limit of large brain size. The main effects of the spherical modal structure are explored, particularly to understand the number of modes that contribute significantly to these observable quantities and the effects of the finite spatial extent of the cortex.

The main results are that when we truncate the modal series it is found that, for physiology plausible parameters, only the lowest few spatial eigenmodes are needed for an accurate representation of macroscopic brain activity. Cortical modal effects can lead to a double alpha peak structure in the power spectrum, although the main determinant of the alpha peak is corticothalamic feedback. At the large brain size limit, spherical and finite-planar geometries converge to the infinite geometries. In the spherical geometry, the coherence function between points decays monotonically as their separation increases at a fixed frequency, but persists further at resonant frequencies. The correlation between two points is found to be positive, regardless of the time lag and spatial separation, but decays monotonically as the separation increases at fixed time lag. This analysis of physiologically-based corticothalamic NFT in a spherical geometry will enable more realistic modeling and analysis of experimental brain signals in future.

**Acknowledgements**

This work was supported by a University of Sydney International Scholarship (USydIS), by the Australian Research Council Center of Excellence for Integrative Brain Function (ARC Center of Excellence Grant CE140100007), and by the Australian Research Council Laureate Fellowship Grant FL140100025.

## P103 Model of plasticity in re-learning auditory and visual localization cues

### Petr Marsalek^1^, Jan Vokral^2^

#### ^1^Charles University of Prague, Institute of Pathological Physiology, Praha, Czechia; ^2^Charles University of Prague, Department of Phoniatrics, Praha, Czechia

##### **Correspondence**: Petr Marsalek (petr.marsalek@lf1.cuni.cz)

*BMC Neuroscience* 2018, **19(Suppl 2):**P103

We study how the two sound localization cues, interaural time difference (ITD) and interaural level difference (ILD) can be re-weighted in order to re-learn new peripheral condition in spatial hearing. In human the ITD is used for low frequency sound localization, the ILD is used for high frequency localization, and between 1000 Hz and 1500 Hz there is a transition zone, where both mechanisms play a role. The ITD and ILD are computed in the early (peripheral) binaural auditory pathway and then the information is transduced into the late (central) processing involving mainly cerebral cortex. Hearing impairment of certain type on one ear leads to re-calibration of the localization mechanisms. The ITD can deliver its time difference (phase difference) as long as the attenuation of the affected ear does not exceed the phase difference discrimination capabilities. The ILD re-calibration can be quickly re-learned to set a new level balance between the two ears to signal the sound direction in the intersection of the horizontal and the middle plane. Experiments show that such re-learning is accomplished in one to two days. These experiments of a partner group in our joint experimental and theoretical project aim at describing the situation in hearing impaired listeners and after introducing binaural hearing aids or binaural cochlear implants. We study the dynamic of the re-learning, re-learning spurious location with the enforced visual cue and spurious or distorted ITD and ILD cues. We will present preliminary results of phenomenological modeling the late (central) processing of the localization cues with the implications for further experimenting and the use of binaural hearing prosthetics for sound and speech localization.

## P104 Optimal readout of neural activity near criticality

### Matias Calderini, Eric Kuebler, Philippe Lambert, Jean-Philippe Thivierge

#### University of Ottawa, Department of Psychology, Ottawa, Canada

##### **Correspondence**: Matias Calderini (mcald052@uottawa.ca)

*BMC Neuroscience* 2018, **19(Suppl 2):**P104

Advances in recording of ongoing activity from large populations of neurons have increasingly shown that information processing arises from the collective behaviour of whole neural circuits. Both in vitro and in vivo recordings suggest that these circuits operate near a critical state poised between fully random and structured activity. Investigations on the role of neural criticality have focused on processing advantages in neural encoding, including transmission, storage and computational power [1]. However, little attention has been paid to the role of neural criticality on accurate downstream decoding of information. The aim of this study is to understand the impact of neural criticality on linear readout of in vitro multi-electrode activity.

We recorded spontaneous population activity from cultured cortical networks under control and pharmacological conditions including a GABA_A receptor antagonist (PTX) and an NMDA/AMPA receptor antagonist (APV-DNQX). As previously reported, the distribution of active electrodes during burst activity followed a power law and the slope of the control condition in log–log scale was close to the expected mean field exponent in the critical state (α = 1.52) (Fig. 1 a. left) [2]. We then trained a linear readout to determine the network of origin of sampled bursts from different networks. Classification error was nearly 0% until approximately 90% of units were discarded. By comparison, deviations from the critical state through pharmacological alteration markedly disrupted performance (panel a. right). We then used a phenomenological branching model where each spike causes a given number of spikes in downstream units [1] as determined by a branching ratio (σ) defined as the average number of descendant to ancestor units across consecutive time-points. Criticality within the branching paradigm arises at a value of σ = 1 at which activity is propagated without amplification (> 1) or extinction (< 1). We simulated a population of N = 100 units and as with in vitro networks, we trained a linear readout to classify network bursts. Again, classification accuracy of the readout was highest near the critical state (σ = 1). These results were robust across different probabilities of spontaneous activity (Fig. 1 b) and connection probabilities between pairs of units. We generalized these findings through a probabilistic analysis of bursting behaviour within the branching paradigm. We found that as the branching ratio decreased from the critical value (σ < 1) and when it increased (σ > 1), the probability of spiking within a burst approached 0 and 1 respectively (Fig. 1 c). Hence, it is only in the critical state that the unique dynamics of the network can emerge (not fully silent nor saturated) and accurate readout is possible. Taken together, our results suggest a novel role of neural criticality in formatting network activity to promote its accurate readout by downstream structures. Furthermore, our findings provide a platform for characterizing neural codes that are optimally advantageous for sensory and motor decoding (Fig. 1).

**Fig. 1:**
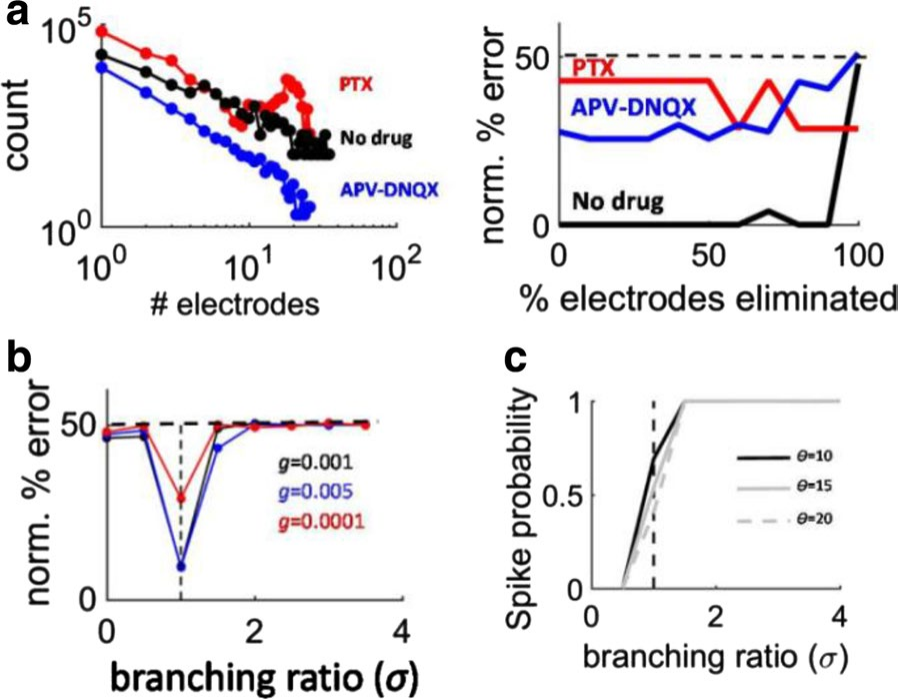
Left: Number of electrodes active per avalanche for control, APV-DNQX and PTX conditions. Right: Readout accuracy of population activity. Classification performance is normalised by number of networks classified. b. Linear discrimination performance of network bursts from networks with various probabilities of spontaneous activity g. Vertical dashed line, σ = 1. Horizontal dashed line, chance-level performance. c. Average spike probability of individual units within a burst for different burst threshold levels (θ). Numerical values are averaged over units and time-steps

**References**

1. Beggs JM. The criticality hypothesis: how local cortical networks might optimize information processing. *Philos Trans A Math Phys Eng Sci* 2008, 366(1864), 329–343.
2. LeBlanc M, Angheluta L, Dahmen K, Goldenfeld N. Universal fluctuations and extreme statistics of avalanches near the depinning transition. *Phys*. *Rev*. *E* 2013, 87, 22126.

## P105 Robust dendritic computations with sparse distributed representations

### Subutai Ahmad^1^, Max Schwarzer^2^, Jeff Hawkins^1^

#### ^1^Numenta, Redwood City, CA, United States; ^2^Pomona College, Department of Computer Science, Claremont, CA, United States

##### **Correspondence**: Subutai Ahmad (sahmad@numenta.com)

*BMC Neuroscience* 2018, **19(Suppl 2):**P105

Empirical evidence suggests that the neocortex represents information using sparse distributed patterns of activity. There exist a variety of sparse coding algorithms demonstrating how to compute sparse representations, and a number of mathematical results on the capacity of sparse representations. Here we focus on dendritic computations and analyze properties of sparse representations from a pattern recognition viewpoint. Are sparse representations useful for neuronal pattern recognition, and under what conditions? The literature on active dendrites and NMDA spikes suggest that a large portion of the dendrites on pyramidal neurons recognize patterns with a small number of synapses. As few as 8–10 active synapses out of 20–30 can initiate dendritic spikes. Given the presence of noisy and unreliable neural inputs, can such a small number of synapses reliably detect patterns? We propose a formal mathematical model for recognition accuracy of binary sparse representations using active dendrites. We derive scaling laws that characterize the chance of false positives and false negatives when detecting patterns under adverse conditions. We describe three primary results. First, we show that using high dimensional sparse representations, a network of neurons can reliably classify a massive number of patterns under extremely noisy conditions. The results hold even when synapses subsample a tiny subset of the target patterns or when individual neurons themselves are unreliable. Second, the equations predict dendritic NMDA spiking thresholds that closely match experimental findings. Finally, we consider two existing computational models of active dendrites: the Poirazi/Mel neuron and the HTM neuron. Through simulations we show that the scaling behavior of these two models closely matches the theory. We show dramatically improved recognition accuracy over published results when “good parameters” (as predicted by the theory) for sparsity and dimensionality are applied, even when the total number of synapses are held constant. The equations assume uncorrelated inputs. Using simulations we also show that the overall trends hold with correlated inputs, although the absolute errors are higher. In summary, the theory presented here complements existing work and represents a practical mathematical framework for understanding the accuracy and robustness of sparse representations in cortical networks (Fig. 1).

**Fig. 1:**
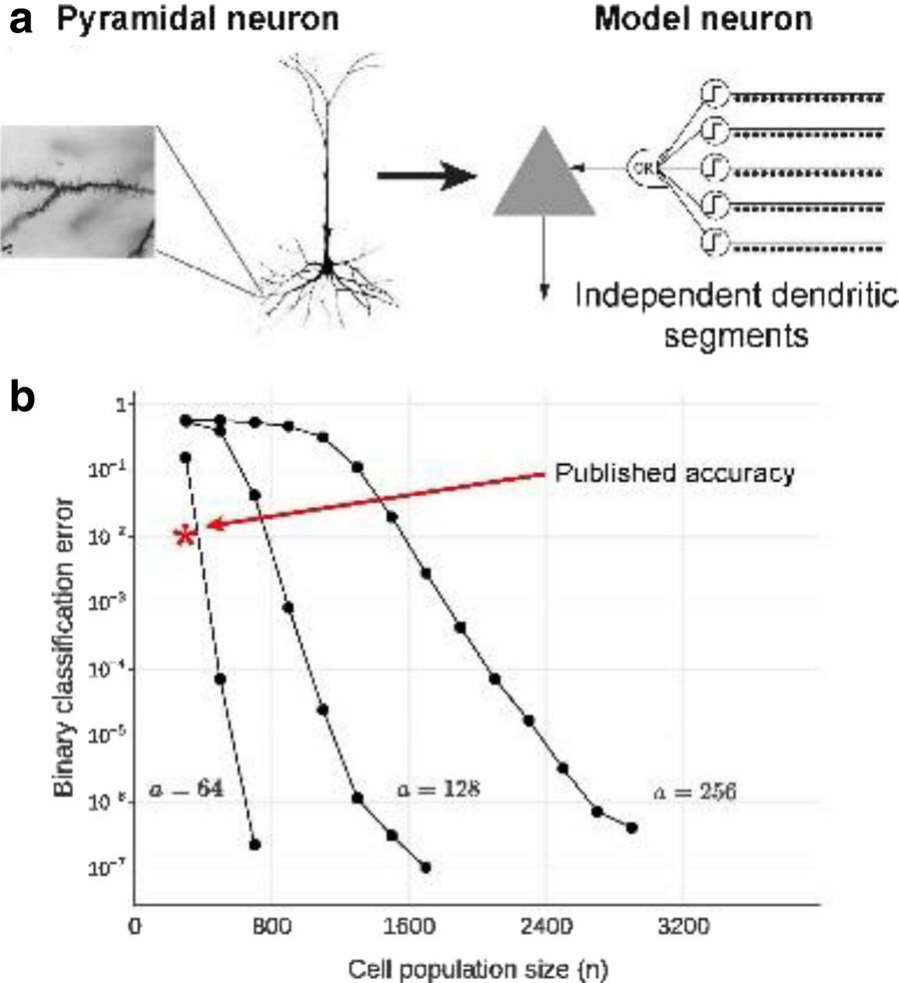
(A) Pyramidal neurons (left) have thousands of excitatory synapses, most of which are located on active distal dendritic segments. Neuron model containing an array of independent active segments (right). (B) Error on the binary classification task described in (Poirazi and Mel 2001). Error rates are dramatically improved by increasing the underlying dimensionality and sparsity of the input representation even when the number of synapses are held constant. The behavior is an almost exact match to error probabilities as predicted by the theory

## P106 Sparse coding and dimensionality reduction in the brain

### Michael Beyeler^1^, Emily L. Rounds^2^, Kristofor D. Carlson^2^, Nikil Dutt^2^, Jeffrey L. Krichmar^2^

#### ^1^University of Washington, eScience Institute, Seattle, WA, United States; ^2^University of California, Irvine, Cognitive Sciences, Irvine, CA, CA, United States

##### **Correspondence**: Michael Beyeler (mbeyeler@uw.edu)

*BMC Neuroscience* 2018, **19(Suppl 2):**P106

Supported by recent computational studies, nonnegative sparse coding (NSC) is emerging as a ubiquitous coding strategy across brain regions and modalities. A combination of nonnegative matrix factorization (NMF) and sparse coding, NSC allows populations of neurons to collectively encode high-dimensional stimuli spaces using a compressed, sparse, and parts-based neuronal code. Specifically, we argue that neuronal circuits can (1) achieve sparse codes through competition, and (2) implement NMF by utilizing spike-timing dependent plasticity with homeostasis (STDPH). We applied NMF to two different datasets: (1) receptive fields in the dorsal subregion of the medial superior temporal area (MSTd), and (2) neurophysiological and behavioral recordings from rat retrosplenial cortex (RSC). In both cases, we were able to show that applying NMF to major inputs into these brain regions can result in a sparse representation that captures important aspects of neuronal response properties of these brain regions. Furthermore, we found similar results applying STDPH to the RSC dataset. These findings support a growing body of evidence that suggests biological neurons use plasticity, such as STDPH, to produce sparse, compact stimulus representations that vastly reduce the dimensionality of their inputs..

## P107 Network interactions can mask intrinsic dynamics in rhythmic circuits

### Jonathan Rubin^1^, Jessica Ausborn^2^, Abigail Snyder^3^, Ilya Rybak^2^, Jeffrey Smith^4^

#### ^1^University of Pittsburgh, Department of Mathemathics, Pittsburgh, PA, United States; ^2^Drexel University, Neurobiology & Anatomy, PA, United States; ^3^Pacific Northwest National Laboratory, WA, United States; ^4^National Institute of Health, MD, United States

##### **Correspondence**: Jonathan Rubin (jonrubin@pitt.edu)

*BMC Neuroscience* 2018, **19(Suppl 2):**P107

Various neuronal circuits, including a range of central patterns generators (CPGs) in the brainstem and spinal cord of many species, exhibit rhythmic activity patterns. In many CPGs, these patterns consist of sequential activations of different neuronal populations that interact through synaptic connections. Significant effort has gone into exploring, using experimental and theoretical methods, the extent to which the intrinsic bursting or pacemaking capabilities of neurons within these populations are responsible for the existence of the network rhythms in which they participate. For example, experimental studies have established the existence of intrinsically bursting neurons in the pre-Botzinger complex (preBotC) of the mammalian respiratory brainstem, and certain experimental manipulations of burst-supporting conductances in these neurons have eliminated respiratory rhythms. Moreover, recent optogenetic studies in the rodent spinal cord have shown that neurons active in extensor or flexor phases of locomotor rhythms can autonomously generate rhythmic activity. These studies, however, leave open an important question: What happens to this intrinsic bursting when the burst-capable neurons are embedded within the full network with which they interact? In many cases, it remains unknown whether the intrinsic bursting capabilities of subsets of neurons affect the emergent dynamics once these neurons are embedded within a synaptically interconnected circuit and how this bursting capability contributes to the properties of these circuits’ rhythmic outputs. In this study, we use highly reduced neuronal models of CPGs composed of small numbers of neuronal populations to highlight some key principles relating to these issues. In particular, we show that neurons’ intrinsic dynamic properties naturally become masked by the network interactions that support multi-phase rhythmic outputs. We establish these results using two models: a half-center locomotor network in which extensor and flexor units are coupled with reciprocal synaptic inhibition and a respiratory network comprising several neuronal populations, including respiratory neurons in the preBotC. In the locomotor case, we demonstrate that changes in drives that switch units’ intrinsic dynamics from oscillatory or bursting to tonic spiking have no impact on the existence or frequency of network rhythms. Effects of drives on rhythm frequency are shown to derive instead from the transition mechanisms, such as escape or release, underlying phase switching within the rhythms, with particular transition mechanisms persisting across parameter changes that alter intrinsic dynamics. Subtly, however, intrinsic dynamics can affect which transition mechanisms can arise within a given parameter regime. In the respiratory case, we similarly illustrate a lack of impact of preBotC intrinsic dynamics on a variety of properties of network rhythms including frequency and amplitude responses to changes in drive; consequences of modulation of inhibition; and even effects of blockade of the persistent sodium current that may underlie the intrinsic rhythmicity within the preBotC. We also show that inclusion of a second excitatory component in the network, the recently identified post-inhibitory complex (PiCo), has little effect on network rhythms, despite the intrinsic oscillation capability of the PiCo.

## P108 Modeling predicts altered ion channel mechanisms and firing properties in striatal neurons of the Q175 mouse model of Huntington’s disease

### Hanbing Song^1^, Christina Weaver^1^, Joseph Goodliffe^2^, Jennifer Luebke^2^

#### ^1^Franklin and Marshall College, Department of Mathematics and Computer Science, Lancaster, PA, United States; ^2^Boston University School of Medicine, Department of Anatomy and Neurobiology, Boston, MA, United States

##### **Correspondence**: Hanbing Song (hsong1@fandm.edu)

*BMC Neuroscience* 2018, **19(Suppl 2):**P108

Huntington’s disease (HD) is a neurodegenerative disorder with severe movement and cognitive dysfunction. Structural and functional neuropathology in HD occurs in the striatum, mainly targeting medium spiny neurons (MSNs), which are regulated largely by striatal fast spiking interneurons (FSIs). MSNs are categorized by the expression of dopamine receptors (D1 or D2) and their contribution to the direct (D1) and indirect (D2) pathways of the basal ganglia. Q175, a transgenic mouse model of HD, exhibits molecular phenotype changes, neuronal dysfunction, and involuntary limb movement. Our recent in vitro work showed increased input resistance in both D1 and D2 MSNs of 12-month old Q175 mice compared to wildtype (WT), but reduced rheobase and action potential amplitudes only in D1 MSNs of Q175 versus WT [1]. This modeling study aims to identify mechanisms that might account for this differential vulnerability, allowing us to gain further insight into striatal dysfunction mechanism in the context of HD. We constructed a 122-compartment conductance-based MSN model in NEURON, based on two published models [2, 3]. We used our recent optimization method [4] to fit parameters controlling the conductance and kinetics of several ion channels of the model to empirical data from several D1 and D2 neurons in WT and Q175 mice. Error functions comprised multiple features of voltage traces from several current clamp steps. Applying machine learning techniques that rank parameters’ importance to firing properties reduced the number of optimized parameters from 17 to 8. This technique was also used to fit parameters of an FSI model to data from WT and HD model mice. Compared to WT MSN models, the Q175 MSN models had lower conductances of fast and persistent sodium (Na+), slow A-type potassium (K+), and T-type calcium channels. These findings were consistent with published RNA sequencing analysis in the striatum of Q175 mice [5, 6]. Rheobase, differentially reduced in D1 but not D2 neurons of Q175 mice, is a strong correlate of neuronal suprathreshold excitability. Analyses showed that the conductance of the persistent Na+ , fast and slow A-type K+, and delayed rectifying K+ channels were the most important determinants of rheobase in our models. The mean conductance of persistent Na+ and slow A-type K+ channels were decreased in both Q175 D1 and D2 MSN models; delayed rectifier K+ channel conductance was reduced only in Q175 D1 MSN models. Adjusting conductance parameters of the fitted WT MSNs based on known up/downregulation of certain genes in Q175 mice was sufficient to account for the rheobase differences between WT and Q175 for D1 but not D2 model MSNs. This computational study of cellular modeling study complements our recent findings of increased dendritic branching complexity and lower EPSC frequency in D1 but not D2 MSNs of Q175 mice [1]. Together this work lays the foundation for constructing a model of the pathological effects of HD on the striatal network.

**References**

1. Goodliffe J et al. Differential changes to D1 and D2 Medium Spiny Neurons in the 12-month-old Q175 ± mouse model of Huntington’s Disease. Submitted, (2018).
2. Wolf JA, Moyer JT, Lazarewicz MT, et al. NMDA/AMPA Ratio Impacts State Transitions and Entrainment to Oscillations in a Computational Model of the Nucleus Accumbens Medium Spiny Projection Neuron. *J Neurosci* 2005, 25:9080–9095.
3. Evans RC, Morera-Herreras T, Cui Y, et al. The Effects of NMDA Subunit Composition on Calcium Influx and Spike Timing-Dependent Plasticity in Striatal Medium Spiny Neurons. *PLoS Comput Biol* 2012, 8:e1002493.
4. Rumbell TH, Dragulic D, Yadav A, et al. Automated evolutionary optimization of ion channel conductances and kinetics in models of young and aged rhesus monkey pyramidal neurons. *J Comput Neurosci* 2016, 41:65–90.
5. Beaumont V, Zhong S, Lin H, et al. Phosphodiesterase 10A Inhibition Improves Cortico-Basal Ganglia Function in Huntington’s Disease Models. *Neuron* 2016, 92:1220–1237.
6. Langfelder P, Cantle JP, Chatzopolou D, et al. Integrated genomics and proteomics define huntingtin CAG length-dependent networks in mice. *Nat Neurosci* 2016, 19:623–633.

## P109 Influence of cortical network topology and delay structure on EEG rhythms in a whole-brain connectome-based thalamocortical neural mass model

### John Griffiths^1^, Jeremie Lefebvre^2^

#### ^1^Rotman Research Institute, Baycrest Health Sciences, Toronto, Canada; ^2^Krembil Research Institute, University Health Network, Toronto, Canada

##### **Correspondence**: John Griffiths (j.davidgriffiths@gmail.com)

*BMC Neuroscience* 2018, **19(Suppl 2):**P109

Large-scale oscillatory activity such as that observed in human M/EEG is believed to arise from a combination of cortical (e.g. intracolumnar excitatory-inhibitory coupling) and thalamocortical rhythmogenic mechanisms. Whilst considerable progress has been made to characterize these mechanisms separately, relatively little work has been done that attempts to unify intracortical and thalamocortical rhythmogenesis within a single theoretical framework. Building on previous work [1–8], here we present and examine a whole-brain connectome-based neural mass model that combines detailed long-range cortico-cortical connectivity based on primate and human tract tracing data with strong, recurrent thalamocortical circuitry. In the model each network node represents an individual cortico-thalamo-cortical motif with four components: a classic Wilson-Cowan1ensemble of excitatory and inhibitory cortical neuronal populations, coupled to a pair of excitatory specific relay and inhibitory reticular thalamic nucleus populations. This system is able to reproduce a variety of known features of human M/EEG recordings, including a 1/f spectral profile; spectral peaks in the alpha, theta, beta, and gamma ranges; and distance-dependent covariance (functional connectivity) structure that is shaped by the underlying anatomical connectivity. Consistent with previous theoretical and experimental observations [2, 3], we also find that increasing sensory drive to thalamic regions triggers a suppression of dominant low frequency rhythms in favour of higher-frequency activity, and also results in an increased susceptibility to entrainment of the entire system by exogeneous stimulation. We find that increasing cortico-cortical connectivity does not disrupt but in fact stabilizes the thalamocortical alpha rhythm, and that varying cortico-cortical conduction delays within physiologically plausible limits modifies, but does not fundamentally alter, the power spectrum and overall dynamics. Finally, we investigate the role of convergence and divergence of corticothalamic and thalamocortical projections, respectively, in determining oscillatory and resonance behaviour in the model, and their implications for the role of the thalamus in promoting and coordinating cortico-cortical synchronization. Taken together, our results clarify the role of cortical network topology and conduction delay structure in shaping both thalamocortical and corticortical rhythmic activity and large-scale brain communication.

**References**

1. Lefebvre J, Hutt A, Frohlich F. Stochastic resonance mediates the state-dependent effect of periodic stimulation on cortical alpha oscillations. *eLife* 2017, e32054 (2017).
2. Mierau A, Klimesch W, Lefebvre J. State-dependent alpha peak frequency shifts: Experimental evidence, potential mechanisms and functional implications. *Neuroscience* 2017, 360, 146–154.
3. Alagapan S, Schmidt SL, Lefebvre J, et al. Modulation of Cortical Oscillations by Low-Frequency Direct Cortical Stimulation Is State-Dependent. *PLOS Biology* 2016, 14, e1002424.
4. van Albada SJ, Robinson PA. Relationships between electroencephalographic spectral peaks across frequency bands. *Frontiers in Human Neuroscience* 2013, 7, 56.
5. Robinson R, O’Connor R, Gordon. *Philosophical Transactions of the Royal Society B: Biological Science* 2005, 360, 1043–1050.
6. Robinson, Whitehouse, Rennie.*Physical review*. *E*, *Statistical*, *nonlinear*, *and soft matter physics* 2003, 68.
7. Cona F, Lacanna M, Ursino M. A thalamo-cortical neural mass model for the simulation of brain rhythms during sleep. *Journal of Computational Neuroscience* 2014, 37, 125–148.
8. Kunze T, Hunold A, Haueisen J, et al. Transcranial direct current stimulation changes resting state functional connectivity: A large-scale brain network modeling study. *NeuroImage* 2016, 140, 174–187.
9. Wilson HR, Cowan JD. Excitatory and inhibitory interactions in localized populations of model neurons. *Biophysical Journal* 1972, 1–24.

## P110 Characterizing neural selectivity in multidimensional sensory feature space

### Chang-Eop Kim, Jihong Oh

#### Gachon Uniersity, Department of Physiology, Seoul, Korea, Republic of

##### **Correspondence**: Chang-Eop Kim (eopchang@gachon.ac.kr)

*BMC Neuroscience* 2018, **19(Suppl 2):**P110

Neurons are conventionally said to be “specific” or “selective” to a specific feature of stimulus if it responds differentially to the feature characterizing the given stimulus. For instance, neurons in the primary somatosensory cortex (S1) have been classified as “noxious-specific” when they respond to pinching by forceps (noxious stimulus), but not to bush stroke (innocuous stimulus) in many studies. Despite the widespread adoption of this simple approach, however, it should be recognized that the given stimulus could have another feature that can be encoded by the neurons, such as texture or dynamics. If we consider these additional features as candidates for the selectiveness of the neurons, the differential responsiveness of the neurons to pinching or brush stroke cannot be interpreted as “noxious-specific” or not. In this case, additional stimulus that has distinct feature characteristics with pinching and brush could help characterizing the neural selectivity. Indeed, we found many S1 neurons of mice showing differential responsiveness to pinching by forceps are not “noxious-specific”, but selective to the features of texture or dynamics by applying 3 types of stimuli with distinct feature characteristics (pinching by forceps, brush stroke, and touching by forceps) using in vivo two-photon Ca2+ imaging. Moreover, we introduce a theoretical framework to characterize the neural selectivity in multidimensional sensory feature space, which are based on the stimulus-feature design matrix and the acquired experimental results. 1. If all feature vectors of the stimulus-feature matrix are unique and the number of unique feature vector (d) equals to 2 s, (s is the number of stimuli with unique feature characteristics), unique selectivity of neurons can be specified, regardless of the experimental results. 2. If there is a unique orthogonal (hyper) plane that can be implemented to classify the experimental results, unique selectivity of neurons can be specified. 3. If there are orthogonal (hyper) plane implementable, but they are not unique, the selectivity cannot be specified and more stimuli are necessary for characterizing the neural selectivity. 4. If there is no orthogonal (hyper) plane implementable to the experimental results, there are two scenarios. First, it can be possible to add reasonable unique feature vector and try to implement orthogonal plane again. Second, interpret the results as “mixed selectivity” of the neurons. We systematically analyzed previous studies characterizing selectivity of sensory neurons using brush and forceps based on our framework and it turned out that many of the previous results that characterized neural selectivity cannot be justified in multidimensional sensory feature space.

## P111 Twin fingerprinting: Optimal mapping of heritable traits in the human connectome

### Uttara TIpnis^1^, Enrico Amico^1^, Linhui Xie^2^, Jingwen Yan^3^, Michael Wang^1^, Mario Dzemidzic^4^, David Kareken^4^, Li Shen^5^, Joaquin Goni^1^

#### ^1^Indiana University-Purdue University, School of Industrial Engineering, West Lafayette, IN, United States; ^2^Indiana University-Purdue University, Electrical and Computer Engineering, Indianapolis, IN, United States; ^3^Indiana University-Purdue University, School of Informatics and Computing, Indianapolis, IN, United States; ^4^Indiana University School of Medicine, Department of Neurology, Indianapolis, IN, United States; ^5^University of Pennsylvania, Perelman School of Medicine, Philadelphia, PA, United States

##### **Correspondence**: Uttara TIpnis (utipnis@purdue.edu)

*BMC Neuroscience* 2018, **19(Suppl 2):**P111

Physical connections between different human gray matter regions occur through long-range white-matter fiber-bundles. These fiber-bundles can be traced through diffusion weighted imaging and processed to estimate a whole-brain structural connectivity (SC) matrix (the human connectome). Using this network approach, anatomic connections between any two brain regions connected by white matter fibers (or streamlines) constitute an*edge*. This complex topological organization is based partly on genetics and environment, with a high common architecture across individuals. However, a more unique individual *fingerprintt* relies on deviations from this common architecture due to genetics and environment. Here we expand a recently proposed framework obtaining optimal identifiability in brain connectomics, to identify the extent to which genetically identical mono-zygotic (MZ) twins share SC, and to isolate the sub-circuits that display high MZ twin shared (or genetic) fingerprinting. To assess the results, we used the same approach for test–retest of di-zygotic (DZ) twins, and a null model based on randomly shuffling the SC profiles of the MZ group. Test–retest of the same subjects is an upper-boundary for the expected MZ identifiability, whereas DZ is a lower boundary and shuffled MZ is a null model for identifiability. The data sample included 148 pairs of twins from the Human Connectome Project (HCP), 74 MZ pairs and 74 DZ pairs. Weighted SC matrices included, for every edge, the average fractional anisotropy (FA) of the streamlines connecting each pair of brain regions within a multimodal 374 region parcellation. To avoid solutions with negative values, we used the non-negative matrix factorization (NNMF) heuristic algorithm to decompose and subsequently reconstruct SC matrices for a different number of components (ranging from 2 to 74). For each decomposition, we calculated the explained variance of each component and components were added in an explained variance descending fashion while evaluating the differential identifiability. Optimal reconstruction was obtained by choosing the reconstruction that corresponds to the maximum differential identifiability (*I*diff). As recently proposed, *I*diffis measured as the correlation gain in same-subject test–retest with respect to between-subject gain. Note that we here expand this concept to MZ- and to DZ-twins, hence allowing for genetic heritability as well as environmental fingerprint evaluation. At optimal*I*diff, individual SC were reconstructed for MZ and DZ subjects, and pairwise intra-class correlations (ICC) for every edge were obtained. Finally, we obtained a differential ICC matrix (i.e., *ICC*MZ–*ICC*DZ). Large positive ICC values indicate edges of high heritability, accounting for environment. The regions with most highly heritable connections include: parietal superior (L), precuneus (L), cingulum medial (L), cingulum anterior (R), temporal inferior (L), and fusiform (L). In summary, through a novel data-driven framework which expands on a recent approach for optimal identifiability on test–retest data, we can detect the most important structural connections and subsequent gray-matter regions that are associated with heritability.

## P112 Spike-timing-dependent plasticity effect on the patterns of neural synchrony

### Leonid Rubchinsky^1^, Joel Zirkle^2^

#### ^1^Indiana University Purdue University Indianapolis & Indiana University School of Medicine, Department of Mathematical Sciences & Stark Neurosciences Research Institute, Indianapolis, IN, United States; ^2^Indiana University Purdue University Indianapolis, Department of Mathematical Sciences, Indianapolis, IN, United States

##### **Correspondence**: Leonid Rubchinsky (lrubchin@iupui.edu)

*BMC Neuroscience* 2018, **19(Suppl 2):**P112

Synchronization of neural activity has been associated with several neural functions. Abnormalities of neural synchrony may underlie different neurological and neuropsychiatric diseases. Neural synchrony in the brain at rest is usually very variable and intermittent. Experimental studies of neural synchrony in different neural systems report a feature which appears to be universal: the intervals of desynchronized activity are predominantly very short (although they may be more or less numerous, which affects average synchrony). This kind of short desynchronization dynamics was conjectured to potentially facilitate efficient creation and break-up of functional synchronized neural assemblies. Cellular, synaptic, and network mechanisms of the short desynchronizations dynamics are not fully understood. In this study we use computational neuroscience methods to investigate the effects of spike-timing-dependent plasticity (STDP) on the temporal patterns of synchronization. We employed a minimal network of two simple conductance-based model neurons mutually connected via excitatory STDP synapses. The dynamics of this model network was subjected to the time-series analysis methods used in prior experimental studies. We found that STDP may alter synchronized dynamics in the network in several ways depending on the time-scale of action of plasticity. However, in general, the action of STDP tends to promote dynamics with short desynchronizations similar (i.e. dynamics similar to those observed in prior experiments). Complex interplay of the cellular and synaptic dynamics may lead to the activity-dependent adjustment of synaptic strength in such a way as to facilitate short desynchronizations in the activity of weakly coupled intermittently synchronized neurons.

## P113 Modeling the variability of spontaneous astrocyte calcium activity and responses to repeated stimuli

### Marsa Taheri^1^, John A. White^2^

#### ^1^University of Utah, Department of Bioengineering, Salt Lake City, UT, United States; ^2^Boston University, Biomedical Engineering, Boston, MA, United States

##### **Correspondence**: Marsa Taheri (marsa.taheri@utah.edu)

*BMC Neuroscience* 2018, **19(Suppl 2):**P113

Accumulating evidence suggests that astrocytes, a major glial cell type, communicate bidirectionally with neurons and play many important roles in the mammalian brain, such as modulating synaptic transmission. Many of these functions are regulated by or linked to astrocyte intracellular Ca2+ signaling. We showed in our recent experimental and computational work [1, 2] that astrocyte Ca2+ transients evoked by a single, focal application of ATP (activating astrocyte G-protein coupled receptors) are temporally heterogeneous due to specific variability in the biological mechanisms underlying the Ca2+ transients. In our current work, we examine astrocyte Ca2+ activity in response to multiple deliveries of ATP stimuli, to assess how astrocytes may respond to neuronal activity and what their Ca2+ dynamics under different experimental conditions reveal about the inputs they are receiving. We use two-photon microscopy to measure Ca2+ activity in mouse cortical astrocytes expressing the genetically-encoded Ca2+ indicator GCaMP5G. We evoke Ca2+ activity through brief (60 ms), focal applications of ATP with varying application time intervals (from 15 s to 4 min). We find that these evoked Ca2+ transients are much more variable than responses to single stimuli. This added variability arises mainly from interactions related to the timing of repeated stimuli, temporally heterogeneous Ca2+ responses to each stimulus (including variability in response latency), and spontaneous/intrinsic astrocyte Ca2+ activity (which is also noisy and unpredictable). Given this high variability, we are interested to see whether we can observe any patterns in the evoked Ca2+ responses and to better understand the variability underlying these responses. We use a phenomenological, statistical modeling approach (rather than a biophysically detailed, mechanistic one) to examine our data, due to the complexity of the data and the fact that many details of the biological mechanisms underlying spontaneous and evoked astrocyte Ca2+ activity remain unknown. First, we ignore the variability in the shape of Ca2+ responses and, instead, make the Ca2+ recordings binary, consisting of only two observable states: On or Off. We then examine the On and Off dwell times for both spontaneous and evoked astrocyte Ca2+ activity, and develop Hidden Markov Models based on our results and knowledge of the biology underlying the Ca2+ activity. By comparing the results generated from these models (e.g. dwell times, the probability of a cellular region being On at any given time during the recording, etc.), we find that the simplest model that reproduces our results consists of 3 hidden states (an Off/Closed state and two On/Open states). Furthermore, we determine which transition rates, at the minimum, must change and by how much in order to switch the Ca2+ activity from spontaneous to evoked. Lastly, we simulate Ca2+ responses to multiple stimuli (by incorporating time-variable transition rates) with different time intervals of application and compare the variability in the resulting Ca2+ activity with our experimental data.

**References**

1. Taheri M, Handy G, Borisyuk A, White JA. Diversity of Evoked Astrocyte Ca2+ Dynamics Quantified through Experimental Measurements and Mathematical Modeling. *Front Syst Neurosci*. 2017, 11, 79.
2. Handy G, Taheri M, White JA, Borisyuk A. Mathematical investigation of IP3-dependent calcium dynamics in astrocytes. *J Comput Neurosci*. 2017, 42(3), 257–273.

## P114 From connectivity to activity: Community detection reveals multiple simultaneous dynamical regimes within networks

### Zoë Tosi^1^, John Beggs^2^

#### ^1^Indiana University Bloomington, Cognitive Science Department, BLOOMINGTON, IN, United States; ^2^Indiana University Bloomington, Department of Physics, Bloomington, IN, United States

##### **Correspondence**: Zoë Tosi (ztosi@iu.edu)

*BMC Neuroscience* 2018, **19(Suppl 2):**P114

A key question in systems neuroscience is the nature of the relationship between synaptic connectivity and neural activity. The latter is thought to underpin cognition, perception, memory, etc., and the degree to which activity shapes topology and topology constrains or supports activity is of great interest to neuroscience and those developing biologically-inspired computers.We now have access to datasets containing recordings from large populations of neurons as well as techniques for assessing the the flow of information between them [1]. Also, highly sophisticated algorithms for the detection of communities on directed, weighted, graphs have been developed [2] as have sophisticated self-organizing microcircuit models capable of broadly replicating key features of synaptic topology [3]. Theoretical studies have predicted that even a small degree of clustering can drastically influence network activity [4]. Combined this data and these analytical tools allow us to begin to probe the nature of the activity/connectivity relationship. We use 512-MEA recordings on organotypic cultures whose information flow has been determined using transfer entropy (TE) [1]. We also used a plastic, self-organizing model MANA (Metaplastic Artificial Neural Architecture) which grows all synaptic connectivity in response to external drive while also organizing its own firing rates to a lognormal distribution. MANA has been shown to be able to realistically, broadly, replicate known features of synaptic topology [3]. In order to detect community structure we used OSLOM (Order Statistics Local Optimization Method) [2], which assesses putative communities according to their probability of occurring in a random network. Statistically significant modules were detected in both functional connectivity networks derived from cultures and synaptic connectivity networks derived from MANA. When rasters were segregated by community membership heterogenous inter- and homogenous intra-community activity was readily apparent (Fig. 1). A technique for comparing the inter-spike interval distributions (ISID) of different neurons was developed specifically to assess this. Clustering algorithms performed on the metric space of these ISID distances assigned neurons in the same community to largely distinct and contiguous regions of the space indicating that communities were indeed comprised of neurons with similar firing dynamics. These results held for both the organotypic cultures and MANA networks despite the differing nature of (functional vs. structural) of their topology. Interestingly, this technique can be used independently to cluster spike trains in arbitrary rasters according to activity type (e.g. regular spiking, fast spiking, intrinsically bursting, etc.). Lastly OSLOM communities in MANA were found to reliably correspond with their constituent neurons’ relationship to the network’s external drive, suggesting that OSLOM could be used to find functionally distinct assemblies in living networks.

**Fig. 1:**
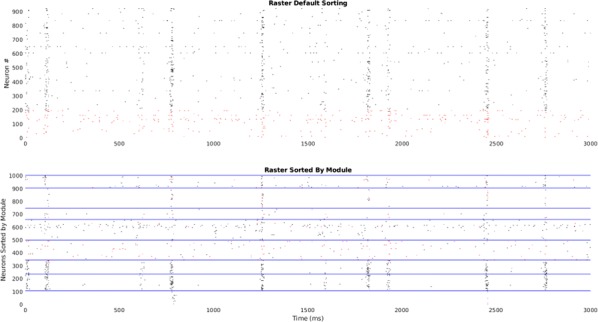
A raster with 1 ms bins over 3 s depicting activity in a MANA network when neurons are ordered arbitrarily and segregated by excitatory (black) and inhibitory (red) neurons. Below: The same raster when neurons are sorted by their community membership as identified by OSLOM. The differing number of rows arises since neurons can reside in more than one community

**References**

1. Nigam, et al. Rich-club organization in effective connectivity. *J*. *of Neuroscience* 2016, 36(3), 670–684.
2. Lancichinetti, et al. Finding statistically significant communities in networks. *PLOS One* 2011, 6(4), e18961
3. Tosi, B. Cortical Circuits from Scratch: A Metaplastic Architecture.… *arXiv* 2017, 1706, 00133
4. Litwin-Kumar, Doiron B. Slow dynamics and high variability in balanced cortical network. *Nat*. *neuro*. 2013, 15(11), 1498

## P115 A generalized platform for modeling electric field effects on neuronal dynamics

### Aaron Regan Shifman, John Lewis

#### University of Ottawa, Department of Biology, Ottawa, Canada

##### **Correspondence**: Aaron Regan Shifman (ashif060@uottawa.ca)

*BMC Neuroscience* 2018, **19(Suppl 2):**P115

The transmembrane ionic currents which underlie action potentials give rise to electric fields in the extracellular space. The high frequency component of these electric fields, due to spiking neurons, is referred to as multi-unit activity (MUA), whereas the lower frequencies, primarily due to synaptic activity, are referred to as local field potentials (LFPs). Interpretation of these signals and source-localization is often challenging, so accurate modeling approaches are critical. Typically, these fields are modeled in a post hoc form, i.e. a traditional neuronal model simulation is run, and then the electric fields are calculated from that simulation. Because the conductivity of the extracellular space is relatively high, the electric fields are generally assumed to be too weak to feedback and influence their own generation. However, in brain regions of lower conductivity, extracellular potentials may play a functional role by influencing membrane potentials, and therefore dynamics of nearby neurons—this is known as ephaptic coupling. The closed-loop nature of ephaptic coupling cannot be modeled using post hoc approaches. We are optimizing more appropriate methods to investigate how different conditions influence the magnitude of ephaptic effects. We have previously shown that extracellular field potentials in simplified networks of model cortical neurons can impede synchronization. In order to study these effects in greater detail, we have developed a generalized framework for modeling ephaptic coupling in morphologically more-realistic neurons. We compare the coupling properties of neurons with “stellate-like” and “pyramidal-like” morphologies to further understand the role that neural geometry plays in ephaptic coupling. Being able to efficiently explore ephaptic coupling from a computational perspective will allow us to better understand the conditions in which electric fields may influence neuronal dynamics in general.

## P116 Synchronization by uncorrelated noise: interacting rhythms in interconnected neuronal networks

### Hermann Riecke, John Meng

#### Northwestern University, Engineering Sciences and Applied Mathematics, Evanston, IL, United States

##### **Correspondence**: Hermann Riecke (h-riecke@northwestern.edu)

*BMC Neuroscience* 2018, **19(Suppl 2):**P116

Rhythmic activities reflecting the near-synchronous activity of large ensembles of neurons are commonly observed in many brain areas. We are here motivated by the observation of multiple, simultaneous gamma-rhythms in the olfactory bulb [4, 2] and by the modular structure that has been predicted to arise from the extensive structural plasticity of the olfactory bulb [5, 1]. It suggests that different bulbar subnetworks that result from the exposure to different odors can support their own different gamma-rhythms. More generally, gamma-rhythms and their coherence across different neuronal networks and brain areas have been proposed to be relevant for the communication between these networks. This raises the question how such rhythms interact with each other. Are the synchronization properties of these collective oscillations similar to those of individual oscillators? This is not the case. We show that for strong, inhibitory coupling different ING-rhythms can become synchronized by noise. Importantly, in contrast to the case of stochastic synchronization, noise synchronizes the rhythms even if the noisy inputs to different neurons are completely uncorrelated (Fig. 1). Key for the synchrony *across* networks is the reduced synchrony *within* the networks: it substantially increases the frequency range across which the networks can be entrained by other networks or by periodic pacemaker-like inputs. More specifically, the noise-enhanced synchronizability of these rhythms arises from a network mechanism: it requires a minimal network size and emerges from the variability in the number of oscillators that participate in the collective oscillation and the resulting variability of the oscillation frequency. We condense this new synchronization mechanism into a simple iterated map, which captures the reverse period-doubling bifurcation that leads to the synchronization. The synchronization mechanism is robust. We demonstrate it for networks comprised of different classes of neuron models (integrate–fire, Morris-Lecar of type 1 and of type 2) with different synaptic couplings and for different network connectivities. Functionally, we find that through this synchronization noise can enhance learning by spike-timing dependent plasticity. Some of these results have been presented in [3].

**Fig. 1:**
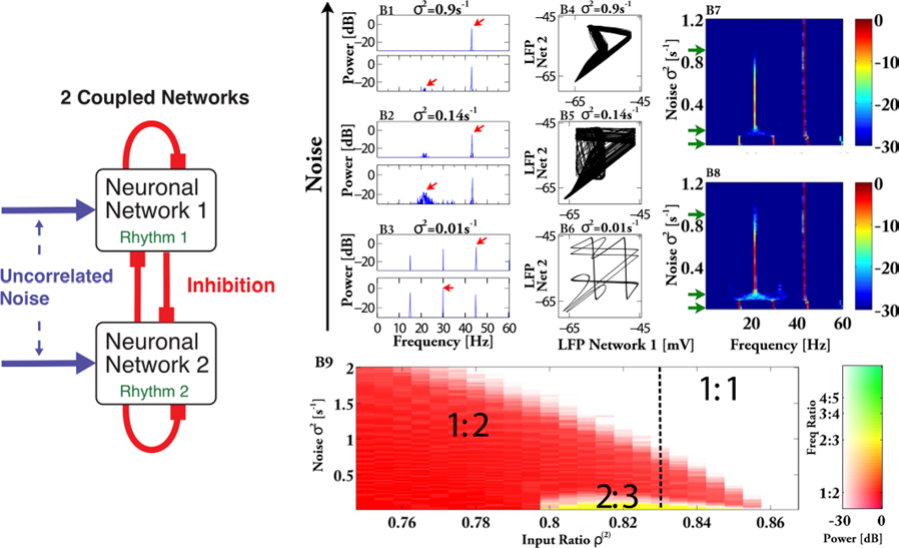
Interaction of two networks of IF-neurons exhibiting ING-rhythms. The inputs to the neurons consist of uncorrelated Poisson spike trains. Increasing the spike-rate variability in the inputs leads to the synchronization of the two rhythms (1:1) via other phase-locked states (2:3, 1:2). B1-B3: Spectra of the two networks. B4-B6: Two-dimensional projection of the attractor. B7-B8: Spectra as a function of noise. B9: Phase diagram as a function of the ratio of the mean inputs to the two networks and the noise strength. Color indicates the frequency ratio of the rhythms

**References**

1. W. Adams, J.N. Graham, X. Han, H. Riecke, “Top-down inputs drive neuronal network rewiring and context-enhanced sensory processing in olfaction”, BIORXIV/2018/271197 (2018).
2. L.M. Kay and P. Lazzara, “How global are olfactory bulb oscillations?”, Journal of Neurophysiology 104, 3 (2010), pp. 1768–1773.
3. [3]J.H. Meng and Hermann Riecke, “Independent Noise Synchronizing Networks of Oscillator Networks”, arxiv1612.06881 [nlin.AO]2016.
4. K. R. Neville and L. B. Haberly, “Beta and Gamma Oscillations in the Olfactory System of the Urethane-Anesthetized Rat”, J. Neurophysiol. 90, 6 (2003), pp. 3921–3930.
5. K.A. Sailor, M.T. Valley, M.T. Wiechert, H. Riecke, G.J. Sun, W. Adams, J.C. Dennis, s. Shirin, G.-L. Ming, H. Song, P.-M. Lledo. “Persistent Structural Plasticity Optimizes Sensory Information Processing in the Olfactory Bulb.”, Neuron 91, 2 (2016), pp. 384–396.

## P117 Classification of morphological and electrophysiological types in mouse visual cortex

### Nathan Gouwens, Staci Sorensen, Jim Berg, Changkyu Lee, Tim Jarsky, Jonathan Ting, Michael Hawrylycz, Anton Arkhipov, Hongkui Zeng, Christof Koch, Susan Sunkin, David Feng, Colin Farrell, Hanchuan Peng, Ed Lein, Lydia Ng, Amy Bernard, John Phillips

#### Allen Institute for Brain Science, Modelling, Analysis and Theory, Seattle, WA, United States

##### **Correspondence**: Nathan Gouwens (nathang@alleninstitute.org)

*BMC Neuroscience* 2018, **19(Suppl 2):**P117

Understanding the diversity of cell types in the brain has been an enduring challenge and requires detailed characterization of individual neurons in multiple dimensions. To profile morphological and electrophysk properties of mammalian neurons systematically, we established a single cell characterization pipeline using standardized patch clamp recordings in brain slices and biocytin-based neuronal reconstructions. We built a publicly-accessible online database, the Allen Cell Types Database, to display these data sets. Intrinsic physiological and morphological properties were measured from over 1,800 neurons from the adult laboratory mouse visual cortex. Quantitative features were used to classify neurons into distinct types using unsupervised methods. We establish a taxonomy of morphologically- and electrophysiologically-defined cell types for this region of cortex with 35 m-types, 17 e-types, and 112 me-types, as well as an initial correspondence with previously-defined transcriptomic cell types using the same transgenic mouse lines (Fig. 1).

**Fig. 1:**
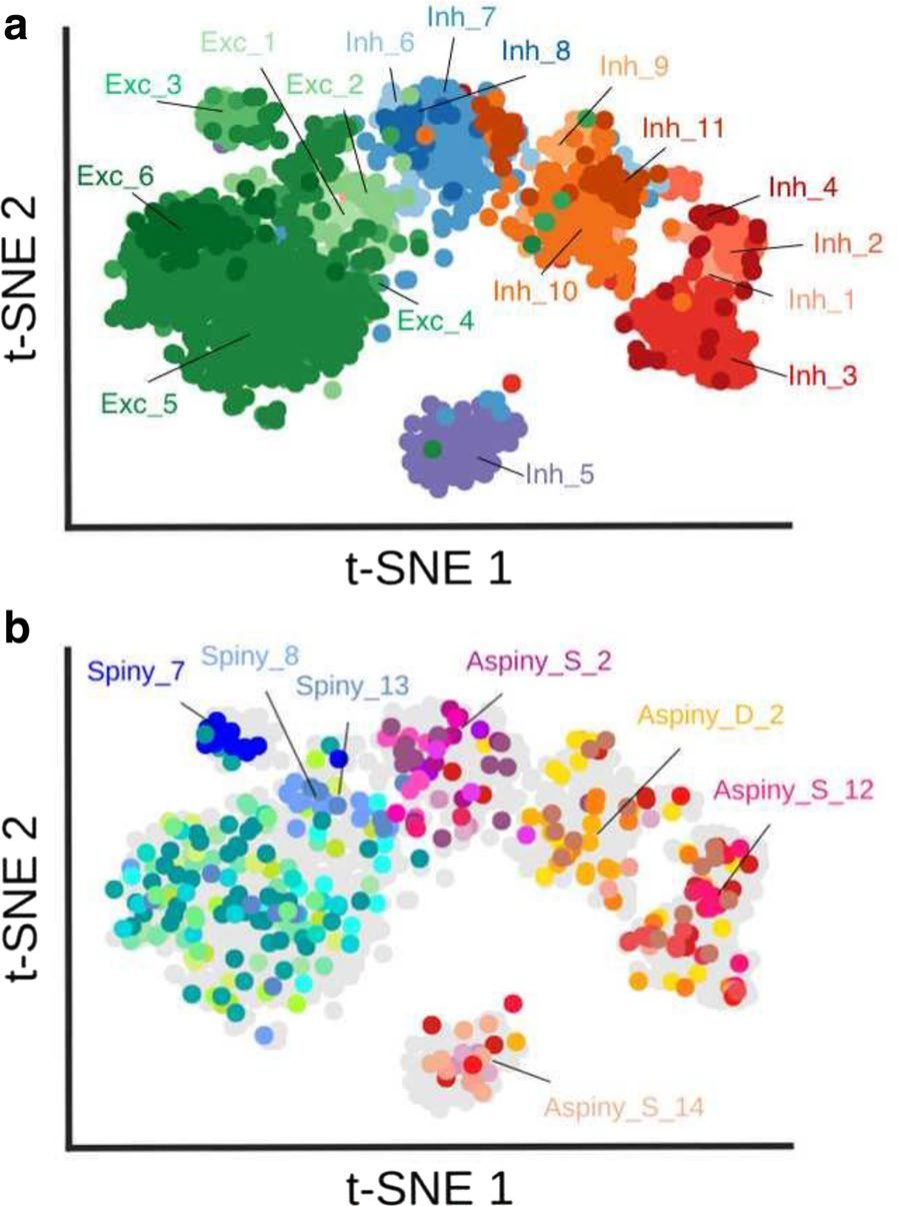
Correspondence between electrophysiology, morphology, and transgenic labels. (a-f) t-SNE projection in electrophysiology space showing e-types (a-b), m-types (c-d), and transgenic lines (e-f). (g) Correspondence between e- and m-types

## P118 Soma-axon coupling configurations that enhance neuronal coincidence detection

### Joshua Goldwyn^1^, Michiel Remme^2^, John Rinzel^3^

#### ^1^Swarthmore College, Swarthmore, PA, United States; ^2^Humboldt University in Berlin, Institute for Theoretical Biology, Berlin, Germany; ^3^New York University, Center for Neural Science & Courant Institute of Mathematical Sciences, New York, NY, United States

##### **Correspondence**: Joshua Goldwyn (jhgoldwyn@gmail.com)

*BMC Neuroscience* 2018, **19(Suppl 2):**P118

*Coincidence detector neurons* are cells that generate spikes preferentially to synaptic inputs that arrive (nearly) simultaneously. Coincidence detection is a fundamental computation by which neurons extract timing information from their inputs. Examples of superb coincidence detectors are principal cells of the medial superior olive (MSO) in the mammalian auditory brainstem. MSO neurons encode sound source location with high temporal precision by distinguishing submillisecond timing differences among inputs. Distinctive biophysical properties contribute to the remarkable temporal precision of MSO neurons. For instance, inactivation of sodium current (INa) and activation of low-threshold potassium current (IKLT) provide dynamic, voltage-gated, negative feedback in subthreshold voltage ranges that can deny adequate summation and spike generation unless the inputs occur with near simultaneity [1, 3]. We investigate additional structural and dynamical specializations in coincidence detector neurons. Using mathematical analysis and simulations of a two-compartment neuron model, we show that the electrical coupling between soma and axon, as well as the distribution of INa and IKLTin soma and axon regions of a model MSO neuron, can be configured to enhance coincidence detection sensitivity. Specifically, we find that a two-compartment model with a “feedforward” configuration—one in which the input regions of a cell (soma and dendrites) strongly drive activity in the spike-generating output region (axon), but backpropagation from the axon into the soma is weak—is significantly advantageous for coincidence detection. In the feedforward configuration, spikes are generated with greater efficiency (fewer INa channels) than a one-compartment model. In addition, INa inactivates more than in models with weak feed forward coupling. The feedforward configuration can, therefore, more effectively enable INainactivation to prevent spike-generation in response to non-coincident inputs. A dynamic IKLT current further enhances coincidence detection sensitivity in these models. Our findings confirm and elucidate physiological studies of MSO neurons, such as the observation that the site of spike-generation is electrically isolated from the soma with weak backpropagation of action potentials [2]. An innovation in our method is to formulate a family of two-compartment neuron models, parameterized by the strength of coupling between input regions (soma + dendrite) and output regions (axon) of a cell. We create a parameter space of coupling configurations, and systematically investigate this family of models to study the relationships between structure, dynamics, and computation in coincidence detection neurons. While our work focuses on the remarkable MSO neurons, our framework can be used more generally to explore effects of soma-axon coupling on dynamics and computation in neurons well-described by a two-compartment framework.

**References**

1. Huguet G, Meng X, Rinzel, J. Phasic Firing and Coincidence Detection by Subthreshold Negative Feedback: Divisive or Subtractive or, Better, Both. *Frontiers in Computational Neuroscience* 2017, 11, http://doi.org/10.3389/fncom.2017.00003
2. Scott LL, Hage TA, Golding NL. Weak action potential backpropagation is associated with high‐frequency axonal firing capability in principal neurons of the gerbil medial superior olive. *The Journal of Physiology* 2017, 583(2), 647–661. http://doi.org/10.1113/jphysiol.2007.136366
3. Svirskis G, Kotak VC, Sanes DH, Rinzel J. Sodium Along With Low-Threshold Potassium Currents Enhance Coincidence Detection of Subthreshold Noisy Signals in MSO Neurons. *Journal of Neurophysiology* 2004, 91(6), 2465. http://doi.org/10.1152/jn.00717.2003

## P119 Short-term plasticity of GABAergic synapses in the Substantia Nigra pars reticulata

### Ryan Phillips, Jonathan Rubin

#### University of Pittsburgh, Department of Mathemathics, Pittsburgh, PA, United States

##### **Correspondence**: Ryan Phillips (ryan.sean.phillips@gmail.com)

*BMC Neuroscience* 2018, **19(Suppl 2):**P119

The substantia nigra pars reticulata (SNr) is one of the primary output nuclei of the basal ganglia and receives converging GABAA receptor mediated synaptic inputs from the direct and indirect pathways. Due to this convergence, the SNr is thought to be important in behaviors associated with these two pathways such as decision making and motor control. Consistent with this idea, abnormal activity within the SNr is associated with parkinsonian symptoms, seizures and impaired decision making. Therefore, understanding how the SNr integrates inputs from these two pathways may be critical for understanding basal ganglia function.

The projections from indirect and direct pathways form synapses at distinct locations on SNr neurons and are known to undergo short-term plasticity. Striatal neurons of the direct pathway preferentially form synapses on the distal dendrites of the SNr neurons and undergo synaptic facilitation [1, 2]. In contrast, neurons from the external segment of the globus pallidus of the indirect pathway form basket-like synapses around the somas of SNr neurons and undergo synaptic depression [1, 3]. The functional significance of the location of these synapses is unclear; however, these spatial characteristics may influence their short-term plasticity properties. GABAA synapses are prone to breakdown of the reversal potential (EGABA) mediated by increases in the intracellular Cl-concentration [Cl-]i [4]. Due to the differences in size and in the distribution of the Cl-extruder KCC2, we hypothesize that dendritic and somatic compartments may have different susceptibilities to breakdown of EGABA, which may contribute to differences in the properties of direct and indirect pathway synapses on SNr neurons. To test this hypothesis, we constructed a novel conductance-based model of an SNr neuron with dendritic and somatic compartments. After establishing that the model’s dynamics matches a range of experimental observations on SNr firing patterns, we used the model to investigate the effects of [Cl-] dynamics on EGABA and short-term synaptic plasticity. We show that GABAA- and KCC2-mediated fluctuations in [Cl-]ican explain many aspects of the short-term plasticity seen with GABAergic inputs from the direct and indirect pathways in the SNr. Integration of GABAA receptor-mediated synaptic inputs to somatic and dendritic compartment is not unique to SNr neurons and therefore these results may have implications for other brain regions.

**References**

1. Connelly WM, et al. Differential short-term plasticity at convergent inhibitory synapses to the substantia nigra pars reticulata. *Journal of Neuroscience* 2010, 30, 44, 14854–14861.
2. Von Krosigk M, et al. Synaptic organization of GABAergic inputs from the striatum and the globus pallidus onto neurons in the substantia nigra and retrorubral field which project to the medullary reticular formation. *Neuroscience* 1992, 50, 3, 531–549.
3. Smith Y, Bolam JP. Convergence of synaptic inputs from the striatum and the globus pallidus onto identified nigrocollicular cells in the rat: a double anterograde labelling study. *Neuroscience* 1991, 44, 1, 45–73.
4. Raimondo JV, Markram H, Akerman CJ. Short-term ionic plasticity at GABAergic synapses. *Frontiers in Synaptic Neuroscience* 2012, 4, 5.

## P120 Simulating pharmacological blockade of persistent sodium currents in respiratory circuits

### Ryan Phillips, Jonathan Rubin

#### University of Pittsburgh, Department of Mathemathics, Pittsburgh, PA, United States

##### **Correspondence**: Ryan Phillips (ryan.sean.phillips@gmail.com)

*BMC Neuroscience* 2018, **19(Suppl 2):**P120

Pharmacological compounds that selectively block voltage-gated ion channels are a fundamental tool in neuroscience. Much of our current theoretical understanding is based on the interpretation of data from experiments dependent on pharmacological manipulations. The mechanism(s) of action for the most commonly used pharmaceutical blockers of voltage-gated ion channels are well understood and fall into one of three mechanistic categories: (1) pore obstruction, (2) shift in activation/inactivation curves, or, less commonly, (3) changes in ion selectivity. Despite this knowledge, the mechanism of blockade is rarely considered when interpreting or simulating experimental data. Generally it is assumed that selective blockade of an ion channel is functionally equivalent regardless of the mechanism. In this theoretical study we show that this assumption may not always be true. To illustrate this idea we simulated blockade of a persistent sodium current (INaP) in the respiratory pre-Bötzinger complex (pre-BötC) via two commonly used sodium channel blockers with distinct mechanisms of action: tetrodotoxin (TTX) and riluzole (RZ). TTX directly obstructs the Na+ pore [1], whereas RZ shifts inactivation in the hyperpolarizing direction [2]. In vitro blocking studies have shown that INaP is critical for rhythmogenic oscillations in the isolated pre-BötC, which has lead to the hypothesis that INaP may be a necessary component of rhythmogenesis in respiratory circuits. This hypothesis however, has fallen out of favor due to the observation that INaP block by RZ fails to stop respiratory rhythms in intact preparations. This conclusion is dependent on the assumption that RZ effectively blocks INaP in in vivo preparations.

Our study shows that, due to RZ’s mechanism of action, INaP can easily be reactivated after blockade by transient hyperpolarizing perturbations. This is not the case with simulated TTX blockade of INaP. In intact preparations, the pre-BötC receives strong inhibition during the interburst interval, which may allow INaP to recover from inactivation after RZ blockade. Consistent with this idea, our simulations of the RZ application in the intact respiratory network predict that RZ but not TTX will fail to effectively block INaP. Additionally, depending on the excitability of the pre-BötC, effective blockade of INaP by TTX may stop respiratory rhythms. Therefore, the failure of RZ to stop respiratory rhythms in experiments is not sufficient to rule out the role of INaP in respiratory rhythm generation. These simulations illustrate the importance of considering the mechanism of action when interpreting and simulating experimental data from pharmacological blocking studies.

**References**

1. Stevens M, Peigneur S, Tytgat J. Neurotoxins and their binding areas on voltage-gated sodium channels. *Front*. *Pharmacol* 2011, 9(2), 2–71
2. Song JH, Huang CS, Nagata K, et al. Differential action of riluzole on tetrodotoxin-sensitive and tetrodotoxin-resistant sodium channels.*J*. *Pharmacol*. *Exp*. *Ther*. 1997, 282(2), 707

## P121 Weak-noise-induced transitions with inhibition and modulation of neural oscillations

### Marius Yamakou, Juergen Jost

#### Max Planck Institute for Mathematics in Sciences, Leipzig, Germany

##### **Correspondence**: Marius Yamakou (yamakou@mis.mpg.de)

*BMC Neuroscience* 2018, **19(Suppl 2):**P121

We analyze the effect of weak-noise-induced transitions on the dynamics of the FitzHugh-Nagumo neuron model in a bistable state consisting of a stable fixed point and a stable unforced limit cycle. Bifurcation and slow-fast analysis give conditions on the parameter space for the establishment of this bi-stability.

In the parametric zone of bi-stability, weak-noise amplitudes may strongly inhibit the neuron’s spiking activity. Surprisingly, increasing the noise strength leads to a minimum (and even silencing of the spiking activity) in the spiking activity, after which the activity starts to increase monotonically with increase in noise strength. We investigate this inhibition and modulation of neural oscillations by weak-noise amplitudes by looking at the variation of the mean number of spikes per unit time with the noise intensity. We show that this phenomenon always occurs when the initial conditions lie in the basin of attraction of the stable limit cycle. For initial conditions in the basin of attraction of the stable fixed point, the phenomenon however disappears, unless the time-scale separation parameter of the model is bounded within some interval. We provide a theoretical explanation of this phenomenon in terms of the stochastic sensitivity functions of the attractors and their minimum Mahalanobis distances from the separatrix isolating the basins of attraction.

## P122 Randomness and structure in artificially generated neuronal networks

### Lida Kanari, Henry Markram, Julian Shillcock

#### École Polytechnique Fédérale de Lausanne, Blue Brain Project, Lausanne, Switzerland

##### **Correspondence**: Lida Kanari (lida.kanari@epfl.ch)

*BMC Neuroscience* 2018, **19(Suppl 2):**P122

The rodent cerebral cortex consists of a few millions neurons that are highly connected by a complex network of synapses. Many factors contribute to generating functional connections among neurons, although the precise mechanisms of this process are not well understood. The shape and relative positions of neurons in space are among the principal geometrical factors that govern the formation of physical connections between neurons and enable functional synapses to form [1–2]. How do the detailed anatomical properties of neuronal morphologies influence the connectivity of the network they generate? To what extent is the structure of the neuronal network encoded in the genetic information of an organism and to what extent do the connectivity patterns emerge from a combination of stochastic events and interactions between growing structures? These questions are important for the digital reconstruction of morphologically detailed neuronal networks that accurately reproduce the statistical properties of the equivalent biological networks. To study the effect of different morphological properties on the resulting network, we have designed a simple generative model based on the theory of random walks. We study the statistical properties of the connectivity of a sequence of networks each of which is generated by morphologies of increasing complexity, e.g., simple random walks, non-intersecting, targeting random walks. A large number of structural and statistical properties of the connectivity of biological networks can be reproduced by artificial networks of random morphologies. In addition, interactions between the growing morphologies are able to reproduce local structural properties that are present in the biological networks. This indicates that stochastic interactions play a significant role in the generation of complex connectivity patterns, and should not be ignored. Since the generative model proposed here represents a major simplification of the highly elegant process of neuronal development, we cannot conclude that the same rules govern the actual growth of neurons into connected networks. We can however, propose that basic principles, derived from fundamental mathematical and physical properties of interacting morphologies, are crucial in the formation of these networks.

**References**

1. Peters A. Thalamic input to the cerebral cortex. *Trends in Neuroscience* 1979, 2, 183–185.
2. Kalisman N, Silberberg G, Markram H. Deriving physical connectivity from neuronal morphology. *Biol*. *Cybern*. 2003, 88(3), 210–218.

## P123 Moving towards the Single Cell Projectome: A multi-modal approach to assessing single-cell morphology and connectivity for classification of layer 2/3 neurons in mouse V1

### Katie Link, Karla Hirokawa, Nile Graddis, Jennifer Whitesell, Bryan MacLennan, Changkyu Lee, Soumya Chatterjee, Staci Sorensen, Julie Harris

#### Allen Institute for Brain Science, Modelling, Analysis and Theory, Seattle, WA, United States

##### **Correspondence**: Katie Link (katiel@alleninstitute.org)

*BMC Neuroscience* 2018, **19(Suppl 2):**P123

Population studies of neurons in the mouse primary visual cortex (VISp) have elucidated numerous axonal projections to cortical and subcortical regions across the brain [1, 2]; however, the specific, long-range projections of individual neurons remain poorly understood. While previous studies have evaluated local connectivity in relationship to morphology [3, 4], the full morphology of individual neurons, including their dendrites and local and long-range projections, have rarely been described [5]. The combination of these important neuronal properties may significantly enrich our understanding of excitatory cell types and their circuitry in the cortex. [6]. We used a novel approach, relying on multiple imaging modalities, to efficiently extract quantitative features from the local morphology and long-range projections of single neurons labeled in vivo. Beginning with in vivo 2-photon microscopy, we were able to reconstruct the dendritic structure of neurons in the intact brain. We observed known and novel features of layer 2/3 neurons, including distinct apical dendrite polarity as well as rare basal dendrite extensions towards the pia. Subsequent serial, 2-photon tomography, followed by automatic image segmentation, directly provided the complete projection target map of single VISp neurons by brain region and layer with respect to the Allen Mouse Common Coordinate Framework (CCF). These images were also used for efficient reconstruction of the full morphology of individual neurons, although with coarser z-sampling than the current standard. Finally, reclaimed sections from serial tomography underwent confocal imaging, which confirmed these characteristic dendritic and axonal features, and allowed us to describe dendritic spine density. Statistical learning methods applied to single-cell, connectivity and morphology data were then used to describe multiple classes of layer 2/3 neurons in mouse visual cortex. These novel cell descriptions will inform circuit and connectivity models of the visual cortex, as well as reveal the significance of incorporating projection target information in future cell type classification studies.

**References**

1. Wang Q, Sporns O, Burkhalter A. Network analysis of corticocortical connections reveals ventral and dorsal processing streams in mouse visual cortex. *Journal of Neuroscience* 2012, 32(13), 4386–4399.
2. Oh SW, Harris JA, Ng L, et al. A mesoscale connectome of the mouse brain. *Nature* 2014, 508(7495), 207.
3. Jiang X, Shen S, Cadwell CR, et al. Principles of connectivity among morphologically defined cell types in adult neocortex. *Science* 2015, 350(6264), aac9462.
4. Oberlaender M, de Kock CP, Bruno RM, et al. Cell type–specific three-dimensional structure of thalamocortical circuits in a column of rat vibrissal cortex. *Cerebral cortex* 2011, 22(10), 2375–2391.
5. Nhan HL, Callaway EM. Morphology of superior colliculus‐and middle temporal area‐projecting neurons in primate primary visual cortex. *Journal of Comparative Neurology* 2012, 520(1), 52–80.
6. Zeng H, Sanes JR. Neuronal cell-type classification: challenges, opportunities and the path forward. *Nature Reviews Neuroscience* 2017, 18(9), 530.

## P124 Oscillatory and broadband contributions to directed functional connectivity in the human cortex

### Julio Chapeton, Sara Inati, Kareem Zaghloul

#### National Institutes of Health, NINDS, Bethesda, MD, United States

##### **Correspondence**: Julio Chapeton (julio.chapeton@nih.gov)

*BMC Neuroscience* 2018, **19(Suppl 2):**P124

In a previous study, we used a measure of directed connectivity, *W*, based on increases in mutual information at consistent time delays in order to define directed functional connections between intracranial EEG electrode sites in human participants. For each participant, we constructed functional networks for 20 data blocks (30 s each) using this measure and showed that the connectivity and topology of these networks, as well as the latencies of individual connections, are conserved over multiple time scales. Interestingly, for some electrode pairs there was clear periodicity in their cross-coupling functions, and the power spectra of the signals from these pairs displayed coincident peaks around theta/alpha frequencies, suggesting that the increases in directed coupling for these pairs may be related to rhythmic synchronous activity at the two electrode sites. Periodicities in the cross-correlation between two signals, or phase locked activity at the same frequency, can be well captured by the coherence function, therefore, to better understand the mechanisms behind the directed coupling we observe, we used the same recordings to compute the magnitude squared coherence function between different brain regions. We find that the coherence functions for pairs with a large value of *W* generally display a dominant peak in the 6–12 Hz range. Although the peak frequency varies across subjects, within a subject, the frequency of maximum coherence is consistent across different electrode pairs. This relationship between directed functional coupling and theta/alpha coherence is further evidenced by a strong correlation between the value of the maximum coherence, *C*max, and*W*across all participants and sites. Along with the dominant peak, there was an overall positive shift in coherence at all low frequencies as compared to higher frequencies (> 32 Hz), with*W*also being strongly correlated with the value of this mean low frequency coherence. Given the definition of coherence as a frequency domain representation of the cross-correlation function (normalized by power), these observations suggest that the rhythmicity of the cross-coupling functions arises from rhythmic synchronous activity in the theta/alpha band, whereas the peak in the cross-coupling function, which defines W, is due to a broadband correlated component in the 2–32 Hz range. Next, we estimated the latency in milliseconds between sites using the cross-spectrum phase at the most coherent frequency, and find that it is in good agreement with the preferred time delay estimated from the mutual information analysis. Such phase and timing relationships are critical if these oscillations are indeed responsible for modulating large-scale neuronal communication. Lastly, because we used relatively long blocks (30 s), it is possible that rhythmic and broadband coupling are not simultaneous, but rather occur independently at different times during the block. In order to assess the time course of these oscillatory and broadband components, we computed time resolved versions of *C* max and *W*, and find that these time series track each other quite reliably, indicating that the correlated broadband signals are concurrent with the synchronous oscillatory activity. Together, our findings support the idea that coherent low frequency rhythms may serve as a mechanism to reliably transmit neural representations encoded in correlated broadband activity over large distances.

## P125 Facilitatory mechanisms during the encoding of frequency-modulated sweeps in the auditory pathway

### Alejandro Tabas^1^, Katharina von Kriegstein^2^

#### ^1^Max Planck Institute for Human Cognitive and Brain Sciences, Leipzig, Germany; ^2^Technische Universität Dresden, Faculty of Psychology, Dresden, Germany

##### **Correspondence**: Alejandro Tabas (alextabas@gmail.com)

*BMC Neuroscience* 2018, **19(Suppl 2):**P125

Short frequency-modulated (FM) sweeps are an integral component of animal communication. In human speech, FM sweeps are the main constituents of formant transitions characterising speech sounds. In this study, we used a combined theoretical and experimental approach to investigate the neural representation and encoding mechanisms of FM-sweeps in the subcortical human auditory system. At the lower levels of the auditory pathway, FM-sweeps are represented as trajectories along the array of frequency-selective neurons forming the tonotopic axis. However, in the cerebral cortex and, to a smaller extend, in the sensory thalamus and the inferior colliculus, a more abstract representation occurs where neurons activate selectively to sweep direction.

Intracranial recordings in mammals identified delayed excitation as the principal neural mechanism inducing sweep direction selectivity in subcortical auditory regions. However, the specific circuitry underlying direction selectivity, and potential effects of top-down modulation are unknown. Here, we introduce? a novel biophysical model of sweep direction selectivity that addresses the specific circuitry and potential top-down modulation effects. The model consists of three arrays of neural ensembles: a frequency-selective array representing neurons along the tonotopic axis, and two direction-selective (up/down) arrays. Each ensemble in the up- (down-) specific layer receives direct input from a single ensemble in the frequency-selective layer, and delayed inputs from adjacent, lower (higher) frequency-selective neurons. Ensembles were modelled using a mean field approximation; forward connections were modelled using realistic synapses. In parallel, we ran an experiment where human participants were asked to adjust a probe pure tone to match the pitch elicited by a short FM sweep. The FM sweeps had two different directions, three different average frequencies, and five different absolute gaps between the starting and ending frequencies. FM-sweeps with frequency gaps up to 400 Hz were perceived as eliciting a pitch percept that was robustly replicated across subjects. Intriguingly, we found a linear relationship between the elicited pitch and the frequency gap. The shift between the average frequency of the sweep and the perceived pitch was biased towards the frequencies present during the ending sections of the sweep, so that up-sweeps were consistently perceived as having the higher pitch in contrast to down-sweeps of the same average frequency. We reasoned that this perceptual effect could be the consequence of a predictive mechanism facilitating the activation of forthcoming frequency-selective neurons in the tonotopic axis. To test this hypothesis, we incorporated an excitatory facilitatory predictive mechanism in our model. This mechanism shifted the activation weight of the populations away from the first milliseconds of the sweeps, when the sweep direction has not been resolved yet, resulting in a bias towards the frequencies activated at the ending of the sweep. The resulting bias was linearly related to the size of the frequency gaps, mirroring the observed human perceptual results. Together, our results indicate the presence of facilitatory predictive mechanisms in the sweep direction encoding system of the human auditory pathway.

## P126 A rigorous statistical test supports a new model of homeostatic plasticity

### Amanda Hanes, Andrew Koesters, Kathrin Engisch

#### Wright State University, Neuroscience, Cell Biology, & Physiology Department, Dayton, OH, United States

##### **Correspondence**: Amanda Hanes (mandy.hanes@gmail.com)

*BMC Neuroscience* 2018, **19(Suppl 2):**P126

Neurons require the ability to adapt to constantly-changing stimuli in order to maintain a functional nervous system. This process, synaptic plasticity, occurs in two known forms which have opposing effects on synaptic physiology. Hebbian plasticity induces rapid, persistent changes at individual synapses in a positive feedback manner. Homeostatic synaptic plasticity (HSP) is a negative feedback effect that occurs in response to chronic alterations in network activity, and prevents the network instability that would result if synaptic strengthening or silencing due to Hebbian plasticity were allowed to go unchecked. Together, these two forms of plasticity underpin nervous system functions such as movement, learning and memory, and perception, while preventing pathological states such as epilepsy. The current hypothesis for HSP states that synapses are modified globally in a uniform, multiplicative manner. Such a mechanism preserves the relative synaptic strengths encoded during Hebbian plasticity. Termed “synaptic scaling,” this hypothesis implies that HSP and Hebbian plasticity are independent of one another and thus operate via separate mechanisms. Here, we use a large HSP data set to demonstrate that multiplicative scaling does not fully account for the mathematical differences between a distribution of mEPSC amplitudes from chronically silenced cultured cortical neurons and a distribution of amplitudes from untreated control neurons. Using the concept of data standardization, we develop a novel and rigorous method for calculating the mathematical transformation accompanying HSP. Our method, comparative standardization, contains both a multiplicative and an additive factor but does not constrain the range of values these factors can take, allowing for an additive factor of zero in a purely multiplicative transformation. We show that homeostatic plasticity is best modeled with a non-zero additive factor. Because the value of the additive factor is negative, this results in the weakest synapses showing little amplification while stronger synapses show robust amplification. Based on these results, we propose a new model in which HSP, in addition to its main function of preserving network stability, heightens the differential synaptic strengths encoded during Hebbian learning.

## P127 Novel approaches to optimize biophysically detailed computational models of single neurons

### Roy Ben-Shalom^1^, Kyung Geun Kim^2^, Kevin Bender^1^

#### ^1^University of California, San-Francisco, Neurology, Oakland, CA, United States; ^2^University of California, Berkeley, EE/CS, Berkeley, CA, United States

##### **Correspondence**: Roy Ben-Shalom (bens.roy@gmail.com)

*BMC Neuroscience* 2018, **19(Suppl 2):**P127

Developing detailed neuronal models that account for the distribution of ion channels that governs neuronal function allows us to simulate brain activity at a high spatiotemporal resolution, and to simulate conditions that are not feasible within standard experimental methods. Naturally, the simulations produced from such models are determined entirely by how biologically accurate the model is. As such, model validation, including its refinement based on fitting relative to empirical observations, is a critical step for proper model construction. This process of fitting a model to neuronal recordings is both computationally intensive and difficult to constrain. Here, our goal is to understand the hurdles in optimization and develop new strategies to improve efficiency; that is, to optimize the optimization. The process of constructing biophysically realistic neuronal models starts with target data: empirical recordings from neurons that contain a range of neuronal responses to user-defined stimuli. The parameters in our neuronal models represent the biophysical properties of the neuron in question such as ion channels distribution and membrane electric properties. Parameters are allowed to vary according to the certainty with which they have been experimentally constrained, from determined values to large ranges of uncertainty. Constraining the unknown, free parameters of the model to fit the recorded data forms an inverse problem where one cannot constrain all the values of the biophysical properties of the neuron, forcing one to guess appropriate parameters based on observations from the neurons response to the stimuli. We compare the neuron’s response to the model response using a score function which rank how similar they are. Score functions compares different aspects of the neuronal response, such as AP height or width, or the root mean square difference between the two responses. To fit the model response to the neuronal response, we use an optimization algorithm to minimize the score function. This process often generates multiple models, each with different underlying parameter sets that nevertheless all match the experimental data to similar degrees. Identifying which of these models best reflects the actual neuronal physiology remains a challenge.

Here, we demonstrate a new technique that identifies the optimal combination of score functions, which in turn sheds light on how such scores can be improved with revised experimental stimuli. Using well-established models that contain 12 free parameters, we extensively sampled the parameter space at different distances from the original parameter set. By applying an array of score functions and evaluating their performance, we could identify the most effective score function for parameter estimation. We then performed a parameter sensitivity analysis for each of the parameters to estimate which score function would be most effective to constrain them. Using the above method, we were able to find the true value of 6/12 parameters of the model reliably.

These analyses instruct which stimulations and score functions should be used when fitting a model to a specific neuron and points which parameters can be constrained in the model. Our novel approach to analyze neuronal models’ parameter space will help neuroscientist quickly identify the biophysical properties required within models that best explain experimental observations.

## P128 Construction of a biochemically detailed single-compartment model for post-synaptic long-term potentiation: application to cortical plasticity

### Tuomo Mäki-Marttunen^1^, Andrew G. Edwards^1^, Kim T. Blackwell^2^

#### ^1^Simula Research Laboratory, Oslo, Norway; ^2^George Mason University, Krasnow Institute for Advanced Study, Fairfax, VA, United States

##### **Correspondence**: Tuomo Mäki-Marttunen (tuomo@simula.no)

*BMC Neuroscience* 2018, **19(Suppl 2):**P128

Ca2+ signaling underlies many processes central to the basic functions of neurons, such as synaptic plasticity. Altered functions of the proteins involved in Ca2+ signaling, alongside with those of ion channels and receptors, have been proposed as a mechanism for schizophrenia [1]. While modeling based on Hodgkin-Huxley-type of descriptions of the ion-channel dynamics and cable theory is a foundation of our understanding of the rapid neuronal biophysics and network interactions, the biochemistry of the intracellular signaling that governs slower processes in neurons remains poorly understood. New models of biochemical networks offer valuable tools for shedding light on these phenomena. However, the large temporal (milliseconds to hours) and spatial scales (nanometers to micrometers) underlying these processes, and the fundamentally stochastic nature imposed by the small numbers of molecules within each signaling domain (e.g., a dendritic spine), causes true multiscale simulation of these processes to be computationally costly, and often prohibitive. This is particularly evident in parameter optimization tasks where the model has to be simulated thousands or millions of times with different parameter values. In this work, we relax the requirement of an extensive spatial scale and build a biochemically detailed single-compartment model capable of predicting induction of long-term potentiation (LTP) in glutamatergic synapses. Our model is based on a previous stochastic multi-compartmental model of dendritic plasticity in a CA1 pyramidal cell [2]. Our model describes the dynamics of the signaling networks mediating beta-adrenergic receptor-dependent potentiation, and includes descriptions for the dynamics of the key signaling molecules PKA, cAMP, CaM, and CaMKII. Furthermore, we analyze how well the obtained single-compartment model can be tuned to reproduce the induction of plasticity as observed in experimental studies of cortical pyramidal cells. In particular, we aim at reproducing both tetanus-induced LTP (as in [3]) and spike-timing dependent LTP (as in [4]). For the latter, we employ multi-compartmental models of cortical neurons from the database of [5] to predict the interaction between the time of a spike in the post-synaptic neuron and the magnitude of the Ca2+ inputs at the synaptic site. Our model offers a unique platform for studying contributions of genes associated with mental disorders to the induction of synaptic plasticity. It also allows modeling the effects of candidate pharmacological treatments that could be used to maintain a baseline level of LTP induction under altered disease conditions.

**Acknowledgement**

Funding: Research Council of Norway, Grant 248828.

**References**

1. Devor A, Andreassen OA, Wang Y, Mki-Marttunen T, Smeland OB, Fan C–C, Schork AJ, et al.: Genetic evidence for role of integration of fast and slow neurotransmission in schizophrenia. *Mol Psychiatry* 2017, 22(6):792–801.
2. Jedrzejewska-Szmek J, Luczak V, Abel T, Blackwell KT: Beta-adrenergic signaling broadly contributes to LTP induction. *PLoS Comput Biol* 2017, 13(7):e1005657
3. Artola A, Singer W: Long-term potentiation and NMDA receptors in rat visual cortex. *Nature* 1987, 330(6149):649–652
4. Seol GH, Ziburkus J, Huang S, Song L, Kim IT, Takamiya K, Huganir RL, Lee H–K, Kirkwood A: Neuromodulators Control the Polarity of Spike-Timing-Dependent Synaptic Plasticity. *Neuron* 2007, 55(6):919–929

## P129 What is the resistivity of the human brain? Insights from direct electrical stimulation, electrocorticographic recordings of the human cortex, and analytic models

### David J. Caldwell^1^, Jeneva A. Cronin^1^, Rajesh P.N. Rao^2^, Andrew L. Ko^3^, Jeffrey G. Ojemann^3^, Larry B. Sorensen^4^

#### ^1^University of Washington, Department of Bioengineering, Seattle, WA, United States; ^2^University of Washington, Computer Science and Engineering, Seattle, WA, United States; ^3^University of Washington, Neurological Surgery, Seattle, WA, United States; ^4^University of Washington, Department of Physics, Seattle, WA, United States

##### **Correspondence**: David J. Caldwell (djcald@uw.edu)

*BMC Neuroscience* 2018, **19(Suppl 2):**P129

Since Penfield, direct electrical stimulation (DES) of the cortical surface has been used extensively to understand the function of different brain regions. Future applications will involve neuroprostheses to improve quality of life for people suffering from neurological damage. To effectively engineer DES for these applications, we need to understand how the electric current flows through the brain. Current flow is controlled both by the anatomy and by the associated electrical resistivity ⍴ of three components: (1) the cerebrospinal fluid (CSF), (2) gray matter, and (3) white matter. Knowing the effective resistivity of the different cortical layers, and how the currents spread is important for targeting electrical stimulation to different brain regions and for calculating the neural response to DES. It is also required for accurate EEG and MEG modeling and for understanding LFP propagation. Given its widespread importance, we were surprised to find the huge reported spread in the literature for previously measured resistivity values for CSF, gray matter, and white matter (Fig. 1).

**Fig. 1:**
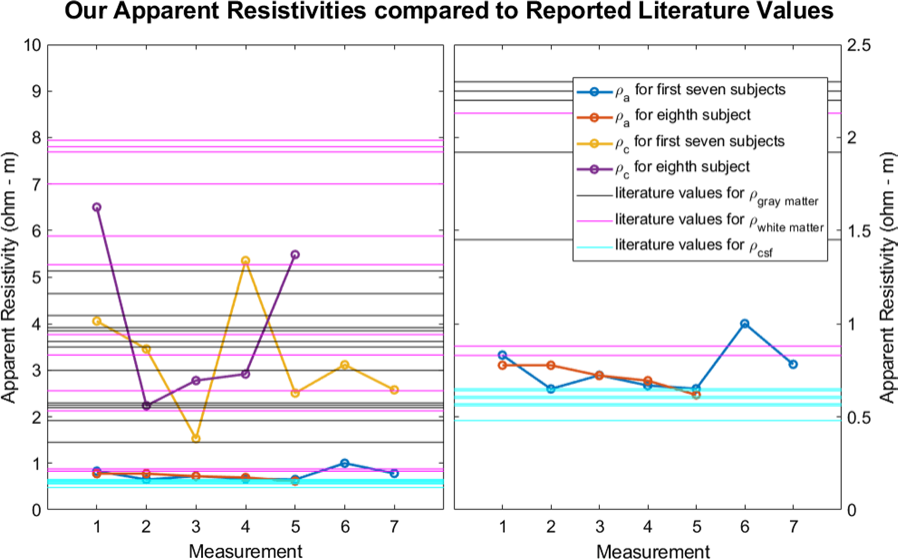
Comparison of our apparent and contact resistivities with values reported in the literature. ⍴a indicates the apparent resistivity, while ⍴c indicates the contact resistivity

Why is the variability so large? We have identified two reasons. First, most previous measurements employed a 2-point method, which actually measured the contact resistance ⍴c which is a property of the contacts in series with the brain, instead of the sheet resistance which is a property of the layered structure of the brain. We show our contact resistance measurements in Fig. 1. Note that our values are consistent with the large reported values for gray and white matter in the literature. Second, for layered systems, all of the layers must be included. This was neglected previously. The standard method is to use 4-point measurements [1]. We made multipoint measurements using clinical electrocorticography (ECoG) grids in 8 humans by stimulating between a pair of electrodes using biphasic, bipolar, constant-current pulses, while recording the voltages across all the non-stimulating electrodes. We fit our data using a simple apparent resistivity ⍴a model. For our first seven subjects, we stimulated electrodes 1 cm apart and measured the voltages at 62 locations. We found the mean apparent resistivity to be 0.76 with a standard deviation of 0.13 O-m. For our eighth subject, we stimulated electrodes 1, 3, and 5 cm apart and measured the voltages at 30 locations. We found the mean apparent resistivity to be 0.72 with a standard deviation of 0.07 O-m. Our results demonstrate the importance of correctly modeling all three components together in order to determine the true resistivity of each one. They also show the utility of multipoint measurements.

**Acknowledgements**

We would like to most importantly thank all our subjects who participated in these studies.

This work was supported by NSF grants EEC-1028725, IIS-1514790, and DGE-1256082, NIH Grant 1T32CA206089-01A1, and a WRF UWIN fellowship.

**Reference**

1. Miccoli I, Edler F, Pfnür H, Tegenkamp C. The 100th anniversary of the four-point probe technique: The role of probe geometries in isotropic and anisotropic systems. *J Phys Condens Matter*. 2015;27(22). 10.1088/0953-8984/27/22/223201.

## P130 Improvement of computational efficiency of a biochemical plasticity model

### Mikko Lehtimäki^1^, Marja-Leena Linne^1^, Lassi Paunonen^2^

#### ^1^Tampere University of Technology, Faculty of Biomedical Sciences and Engineering, Tampere, Finland; ^2^Tampere University of Technology, Mathematics Laboratory, Tampere, Finland

##### **Correspondence**: Mikko Lehtimäki (mikko.lehtimaki@tut.fi)

*BMC Neuroscience* 2018, **19(Suppl 2):**P130

Multi-scale models in neuroscience typically integrate detailed biophysical neurobiological phenomena from molecular level up to network and system levels. Such models are very challenging to simulate despite the availability of massively parallel computing systems. Model Order Reduction (MOR) is an established method in engineering sciences, such as control theory. MOR is used in improving computational efficiency of simulations of large-scale and complex nonlinear mathematical models. In this study the dimension of a nonlinear mathematical model of plasticity in the brain is reduced using mathematical MOR methods.

Traditionally, models are simplified by eliminating variables, such as molecular entities and ionic currents, from the system. Additionally, assumptions of the system behavior can be made, for example regarding the steady state of the chemical reactions. However, the current trend in neuroscience is incorporating multiple physical scales of the brain in simulations. Comprehensive models with full system dynamics are needed in order to increase understanding of different mechanisms in one brain area. Thus the elimination approach is not suitable for the consequent analysis of neural phenomena. The loss of information typically induced by eliminating variables of the system can be avoided by mathematical MOR methods that strive to approximate the entire system with a smaller number of dimensions compared to the original system. Here, the effectiveness of MOR in approximating the behavior of all the variables in the original system by simulating a model with a radically reduced dimension, is demonstrated. In the present work, mathematical MOR is applied in the context of an experimentally verified signaling pathway model of plasticity [1]. This nonlinear chemical equation based model describes the biochemical calcium signaling steps required for plasticity and learning in the subcortical area of the brain. In addition to nonlinear characteristics, the model includes time-dependent terms which pose an additional challenge both computational efficiency and reduction wise. The MOR method employed in this study is Proper Orthogonal Decomposition with Discrete Empirical Interpolation Method (POD + DEIM), a subspace projection method for reducing the dimensionality of nonlinear systems [2]. By applying these methods, the simulation time of the model is radically shortened. However, our preliminary studies show approximation error if the model is simulated for a very long time. The tolerated amount of approximation error depends on the final application of the model. Based on these promising results, POD + DEIM is recommended for dimensionality reduction in computational neuroscience. In summary, the reduced order model consumes a considerably smaller amount of computational resources than the original model, while maintaining a low root mean square error between the variables in the original and reduced models. This was achieved by simulating the system dynamics in a lower dimensional subspace without losing any of the variables from the model. The results presented here are novel as mathematical MOR has not been studied in neuroscience without linearisation of the mathematical model and never in the context of the model presented here (Fig. 1).

**Fig. 1:**
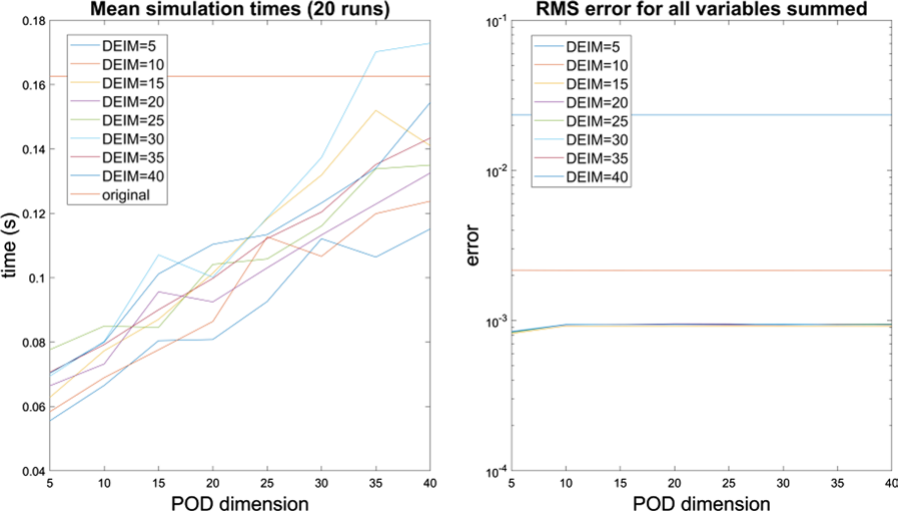
Simulation time (left) and Root Mean Square error (right) of the reduced model compared to the full dimensional model, for DEIM dimensions as function of POD dimension

**References**

1. Kim B, Hawes SL, Gillani F, et al. Signaling pathways involved in striatal synaptic plasticity are sensitive to temporal pattern and exhibit spatial specificity. *PLoS Computational Biology* 2013, 9(3), p.e1002953.
2. Chaturantabut S, Sorensen DC. Nonlinear model reduction via discrete empirical interpolation. *SIAM Journal on Scientific Computing* 2010, 32(5), 2737–2764.

## P131 Modeling traveling wave dynamics in the visual cortex

### Lawrence Oprea

#### McGill University, Physiology, Montreal, Canada

##### **Correspondence**: Lawrence Oprea (laurentiu.oprea@mail.mcgill.ca)

*BMC Neuroscience* 2018, **19(Suppl 2):**P131

In normal vision, our eyes flicker (saccade) between behaviourally pertinent objects in the environment. During ocular transit we become effectively blind as processing of motion-blurred images is suppressed in all parts of the visual system. Recent primate work has shown that traveling waves of local field potential propagate through visual area V4 after a saccade [1]. These waves help to increase visual sensitivity, and therefore may alleviate suppression. My research goal is to create a neuronal network model to classify and analyze the rapid dynamics of wave initiation and interactions with the substrate. Previous work has shown very fast switching between wave and non-wave states through the modulation of Gaussian coupling kernels, that mirror the center-surround topologies in the visual cortex [2]. I have investigated the wave dynamics produced by different kernel parameters through an optical flow methodology, that allows models of any dimensionality to be analyzed as 2D vector fields. Networks are composed of simple spiking (Izhikevich) neurons which allow large lattices with high computational efficiency [3]. Machine learning techniques can then be applied to automatically classify extracted features from very large numbers of simulations, to produce a databank of dynamic patterns for comparison with experimental data.

**References**

1. Zanos TP, Mineault PJ, Nasiotis KT, et al. A sensorimotor role for traveling waves in primate visual cortex. *Neuron* 2015, 85(3), 615–627.
2. Heitmann S, Gong P, Breakspear M. A computational role for bistability and traveling waves in motor cortex. *Frontiers in Computational Neuroscience* 2012, 6, 67.
3. Izhikevich E. Simple model of spiking neurons. *IEEE Transactions on Neural Networks* 2003, 14(6), 1569–1572. 10.1109/tnn.2003.820440

## P132 Cusps enable line attractors and graded information channels in neural computation

### Zhuocheng Xiao^1^, Jiwei Zhang^2^, Andrew Sornborger^3^, Louis Tao^4^

#### ^1^University of Arizona, Department of Mathematics, Tucson, AZ, United States; ^2^Beijing Computational Science Research Center, Applied and Computational Mathematics, Beijing, China; ^3^Los Alamos National Laboratory, Computer, Computational, and Statistical Sciences (CCS-3), Los Alamos, NM, United States; ^4^Peking University, Center for Bioinformatics, National Laboratory of Protein Engineering and Plant Genetic Engineering, Beijing, China

##### **Correspondence**: Zhuocheng Xiao (xiaoz@email.arizona.edu)

*BMC Neuroscience* 2018, **19(Suppl 2):**P132

Line attractors have been suggested as a dynamical mechanism for encoding graded information (i.e., value of a continuous variable), such as working memory, oculomotor control, locomotion, and sensory processing. As a natural extension of point attractors, the attractive states in a line attractor consist of an infinite set of fixed point, all of which lie on a line (or more generally, a curve) in the state space. Therefore, different initial conditions would potentially converge to different fixed points on the line, and graded information is preserved in this process. The stability along the line attractors could naturally provide stable propagation along a feed-forward network (FFN) that enables graded-information propagation with pulse gating, which is an information-processing structure that widely exists in brain. In this research, we use a Fokker–Planck method to describe the system to understand the effects of pulse gating in information transfer in a high dimensional, non-linear, feed-forward integrate-and-fire network. We use synaptic current amplitudes and moments of membrane potential’s probability distribution to describe the iterative population dynamics and identify an approximate line attractor (therefore, a graded information propagation domain) in the state space consisting of amplitude and the first 2 moments. An analysis of the line attractor shows that it is an attracting one-dimensional principal manifold consisting of a central saddle surrounded on either side by stable fixed points. Along the linear manifold defined by the fixed points, the dynamics are slow because of ghost effect from a fold bifurcation in the system. We show that a cusp catastrophe gives rise to the line attractor, whose robustness underlies the ghost dynamics near the fold of the cusp. For the robustness of the line attractor, a further investigation into the slowness measure on the linear manifold exhibits a broad, roughly linear range in the parameter space where the trajectories move slowly, indicating a large region over which graded information could be faithfully propagated. The graded propagation, or the line attractor, is robust, since the region has a non-zero area in the parameter space. The cusp catastrophe helps us show how line attractors work in biologically realistic neuronal networks. Moreover, since the fold of this cusp separates a graded propagation region in the parameter space, from a region of bi-stability, we demonstrate that pulse gating current, synaptic coupling strength, and variance of the system can be used to enable or disable attracting one-dimensional manifolds and thus, form a switch between graded/binary information-transferring channel in feed-forwards networks.

## P133 Population vector decoding for optical imaging with fNIRS (functional near-infrared spectroscopy)

### Nicoladie Tam^1^, George Zouridakis^2^, Luca Pollonini^2^

#### ^1^University of North Texas, Department of Biological Sciences, Denton, TX, United States; ^2^University of Houston, Department of Engineering Technology, Houston, TX, United States

##### **Correspondence**: Nicoladie Tam (nicoladie.tam@unt.edu)

*BMC Neuroscience* 2018, **19(Suppl 2):**P133

Population vector has been well established computationally to decode the intended movement direction recorded from motor cortical neuronal firings [1]. Movement direction can be computed frpm, the vectorial sum of the preferred directions of each participating motor neuron. However, decoding of movement direction using the non-invasive fNIRS (functional near-infrared spectroscopy) optical imaging has yet to be established. Towards this goal, the paper focuses on deriving the computation of the neural activation of a group of neurons imaged by each optrode to produce the computed population vector. In order to determine the groups of motor cortical neurons that are participating in generating the population vector, we apply the assumption that task-related neurons co-vary with the movement direction. Since the population of motor cortical neurons is recorded simultaneously by multiple optrodes, the difference between the hemodynamic responses of all other recorded channels with respect to a reference channel can be used a measure to determine whether the other channels co-vary with the reference channel. This co-varying relationship can be determined by the difference of the hemodynamic signals between any other channel and the reference channel. We will use a matrix format to show the co-varying relationship of the difference-signal between all other channels with respect to the reference channel to visualize which groups of neurons are participating in the movement. Once the task-related groups of neurons are identified, then the vectorial sum of these groups of neurons can be used to compute the population vector as described by Georgopoulos [1]. Using our proposed computational method to compute the vectorial sum of hemodynamic signals, we validated the population vector of the intended movement direction experimentally in human subjects which had been shown earlier that movement can be decoded from the hemodynamic signals from the optrodes [2, 3]. To validate the proposed computational method, human subjects were asked to move in two orthogonal directions while the hemodynamic signals were recorded. The results showed that the population vector could be estimated from the hemodynamic signals of the simultaneously-recorded optrodes, demonstrating the parallel-processing of neural firings by groups of neurons in the motor cortex.

**References**

1. Georgopoulos AP, Schwartz AB, Kettner RE: Neuronal population coding of movement direction. Science (New York, NY) 1986, 233(4771):1416–1419.
2. Tam ND, Zouridakis G: Decoding movement direction from motor cortex recordings using near-infrared spectroscopy. In: Infrared Spectroscopy: Theory, Developments and Applications. edn. Hauppauge, NY: Nova Science Publishers, Inc.; 2014.
3. Tam ND, Pollonini L, Zouridakis G: Decoding movement direction using phase-space analysis of hemodynamic responses to arm movements based on functional near-infrared spectroscopy. In: Proceedings of IEEE Engineering in Medicine & Biology Society: 2016; 2016: 1580–1583.

## P134 Firing-rate based network modeling of the dLGN circuit: Effects of cortical feedback on spatiotemporal response properties of relay cells

### Gaute Einevoll^1^, Milad Hobbi Mobarhan^2^, Geir Halnes^1^, Pablo Martinez-Canada^3^, Torkel Hafting^4^, Marianne Fyhn^2^

#### ^1^Norwegian University of Life Sciences, Faculty of Science and Technology, Aas, Norway; ^2^University of Oslo, Department of Biosciences, Oslo, Norway; ^3^University of Granada, Granada, Spain; ^4^University of Oslo, Institute of Basic Medical Sciences, Oslo, Norway

##### **Correspondence**: Gaute Einevoll (gaute.einevoll@nmbu.no)

*BMC Neuroscience* 2018, **19(Suppl 2):**P134

Visually evoked signals in the retina pass through dorsal lateral geniculate nucleus (dLGN) on the way to the primary visual cortex (V1). This is however not a simple one-way flow of information, as there is a significant feedback from V1 back to neurons in dLGN. Despite numerous experimental and modeling studies, the functional role of this feedback is still debated. In the present study we use a firing-rate model, the extended difference-of-gaussians (eDOG) model, to study key features of visually evoked cortical feedback effects on dLGN relay cells. Our analysis indicates that a special mix of excitatory and inhibitory cortical feedback accounts best for available experimental data. In this configuration ON-center relay cells receive feedback from ON-center cortical cells, consisting of a fast and spatially narrow excitatory component and an (indirect) inhibitory component which is slow and spatially widespread. Here, the excitatory and inhibitory ON–ON feedback connections are accompanied by inhibitory and excitatory OFF–ON connections, respectively, following a phase-reversed (push–pull) arrangement. To facilitate further applications of the eDOG model, we have developed the Python tool (pyLGN), which allows for easy adaptation of the model to new situations. The advantage of this tool lies in its computational and conceptual ease, allowing for fast and comprehensive exploration of various scenarios for the organization of the cortical feedback.

## P135 Computational modeling of neuron-astrocyte interactions: Evolution, reproducibility, comparability and future development of models

### Tiina Manninen^1^, Ausra Saudargiene^2^, Riikka Havela^3^, Marja-Leena Linne^3^

#### ^1^Tampere University of Technology & Stanford University, Faculty of Biomedical Sciences and Engineering & Department of Neurobiology, Tampere, Finland; ^2^Lithuanian University of Health Sciences & Vytautas Magnus University, Neuroscience Institute & Department of Informatics, Kaunas, Lithuania; ^3^Tampere University of Technology, Faculty of Biomedical Sciences and Engineering, Tampere, Finland

##### **Correspondence**: Marja-Leena Linne (marja-leena.linne@tut.fi)

*BMC Neuroscience* 2018, **19(Suppl 2):**P135

Astrocyte research has turned out to be a fascinating and popular research field with two groups of researchers having opposite opinions about the importance of astrocytes in brain information processing and plasticity [1–3]. We believe that computational modeling of the biophysics of neuron-astrocyte interactions can greatly help address the dilemma.We have therefore, as the first ones, characterized, categorized, and evaluated in detail more than a hundred published computational models of single astrocytes, astrocyte networks, neuron-astrocyte synapses, and neuron-astrocyte networks [4] as well as studied the reproducibility and comparability of some of the models [5]. Based on this knowledge and additional experimental findings, we have constructed and implemented new neuron-astrocyte synapse models [6]. In this study, we propose to gather the state-of-the-art experimental and computational knowledge to help guide the future astrocyte research. Two of the most important challenges in experimental work on astrocytes are the lack of selective pharmacological tools and the partially contradictory results obtained in in vivo and in vitro studies [1–3]. In computational studies on astrocyte, the most important challenges are the creationof new models without clear explanation how they differ from the previously published models and what new predictions the models make [4]. Furthermore, combining unclearly given model details in the publications with nonexistent online model implementations make the reproducibility and comparability studies as well as the development of previously published models impossible, or at least difficult [4, 5]. We want to emphasize the importance of using common description formats for defining the models in the publications and description languages for exchanging the models through online repositories. Our overall goal is to develop both detailed and reduced models of neuron-astrocyte interactions for different brain areas, allowing additional testing and clarification of the controversies observed in experimental wet-lab studies [1–3]. Only through systematic integration of in vivo, in vitro, and in silicodata, using reproducible science approach, are we be able to understand how astrocytes may contribute to brain information processing and plasticity.

**References**

1. Bazargani N, Attwell D. Astrocyte calcium signaling: the third wave. *Nat Neurosci* 2016, 19(2), 182–189.
2. Fiacco TA, McCarthy KD. Multiple lines of evidence indicate that gliotransmission does not occur under physiological conditions. *J Neurosci* 2018, 38(1), 3–13.
3. Savtchouk I, Volterra A. Gliotransmission: beyond black-and-white. *J Neurosci* 2018, 38(1), 14–25.
4. Manninen T, Havela R, Linne ML. Computational models for calcium-mediated astrocyte functions. *Front Comput Neurosci* 2018, 12, 14.
5. Manninen T, Havela R, Linne ML. Reproducibility and comparability of computational models for astrocyte calcium excitability.*Front Neuroinform* 2017, 11, 11.
6. Havela R, Manninen T, Saudargiene A, Linne ML. Modeling neuron-astrocyte interactions: towards understanding synaptic plasticity and learning in the brain.*13th International Conference on Intelligent Computing* (*ICIC 2017*) published in Intelligent Computing Theories and Application, Part II, Lecture Notes in Computer Science 10362, eds. D.-S. Huang et al., 157–168, Liverpool, UK, 07.-10.08.2017.

## P136 Data-driven study of synchronous population activity in generic spiking neuronal networks: How much do we capture using the minimal model for the considered phenomena?

### Jugoslava Acimovic^1^, Heidi Teppola^1^, Tuomo Mäki-Marttunen^2^, Marja-Leena Linne^1^

#### ^1^Tampere University of Technology, Faculty of Biomedical Sciences and Engineering, Tampere, Finland; ^2^Simula Research Laboratory, Oslo, Norway

##### **Correspondence**: Jugoslava Acimovic (jugoslava.acimovic@gmail.com)

*BMC Neuroscience* 2018, **19(Suppl 2):**P136

**Introduction and goals:** Analysis of Spiking Neuronal Network (SNN) dynamics has been one of the central topics within the computational neuroscience community, including theoretical studies on characterizing dynamical regimes in SNN models, incorporating various phenomenologically described biophysical mechanisms, and interpreting them through comparison of in vitro/in vivo against in silico data. Particularly, in vitro recordings from dissociated cultures (e.g. Marom and Shahaf 2002; Waagenar et al. 2006; Suresh et al. 2016) have been often combined with SNN models. A number of contributions examined SNN or mean-field models aiming to explain what initiates, maintains and modulates the experimentally observed properties of population activity (Latham et al. 2000; Giuliagno et al. 2004; Wallach et al. 2008; Baltz et al. 2011; Mesquelier and Deco 2013; Gigante et al., 2015; Lonardoni et al. 2017). The in vitro literature seems to converge towards the following: dissociated neurons self-organize into random networks (possibly with distinct hubs) and develop spontaneous population bursts, the brief periods of intensive spiking activity spreading across the culture. In neocortical cultures, it has been hypothesized that this activity emerges from random fluctuations of uncertain origin and is maintained by complex interaction of somatic and synaptic currents. Three major types of synaptic receptors, the excitatory glutamatergic (AMPA-R and NMDA-R) and the inhibitory GABAergic, have impact on the spread, duration, and frequency of bursts; AMPA-R is often associated with fast initiation, NMDA-R with maintenance, and GABAA-R with dampening of population activity (Teppola et al. 2011). We here examine how much of the experimentally observed complexity and variability in spiking patterns can be captured by the conventional SNNs. We particularly focus on contribution of the three indicated receptor types to the overall system dynamics.

**Methodology:** We constructed a model composed of integrate-and-fire neurons with appropriate somatic currents. The synaptic model includes short-term plasticity on the presynaptic side and the three considered receptor types on the postsynaptic side. In order to properly challenge our model, we used a rich set of data systematically collected while pharmacologically manipulating specific synaptic receptors (see Teppola et. al. 2011). We carried out model fitting using a powerful but computationally demanding genetic algorithm. The model was simulated for many parameter sets, and in silico data was collected and analyzed to extract the same measures used to quantitatively describe the experimental data. The analysis of in silico data had to be reliable and fully automatic, which is challenging for the existing algorithms (Cotterill et al. 2016). Distributions of the obtained measures for in silico and in vitro data were compared to compute the goodness of fit and identify optimal parameters. We tested various combinations of optimized parameters and data measures.

**Challenges and results:** Preliminary tests give relatively good fit of the measures reflecting the spontaneous population burst structure and less success in fitting the burst frequency (see Fig. 1). Comparable results are obtained across a relatively large parameter space suggesting the need to better constrain the model fitting procedure.

**Fig. 1:**
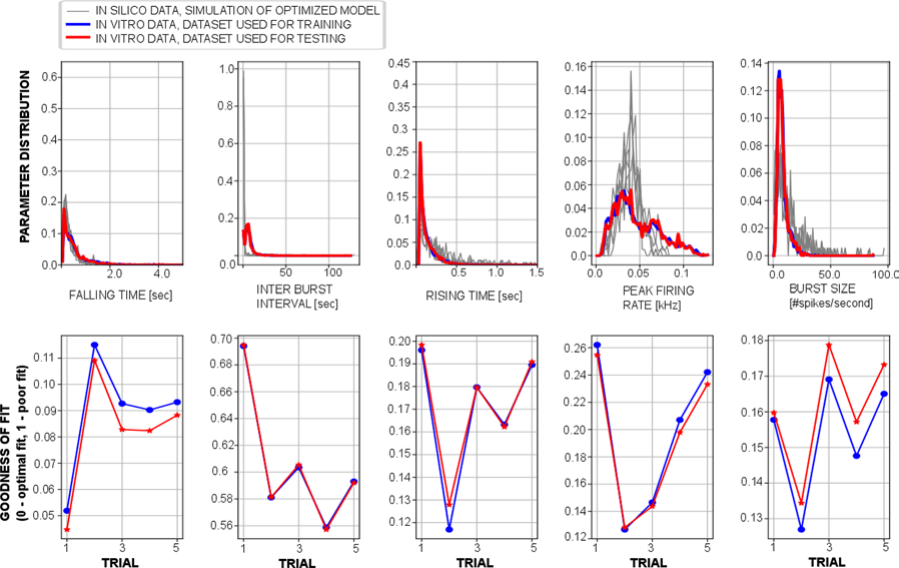
Preliminary results obtained from model fitting to the control data set (no pharmacological manipulations). After segmenting recordings into population bursts, the following measures are extracted: falling and rising times of a burst, interval between successive bursts, size of a burst (number of spikes divided with the bursting time), and population firing rate at the burst peak. Row one: Distribution of each extracted burst measure (blue and red—experimentally obtained data divided into training (blue) and test set (red); grey—in silico data obtained by simulating the model optimized using the training set). Row two: Goodness of fit on the scale of 0 (optimal fit) to 1 (fitting failed), five fitting trials shown (x axis)

## P137 Fast gabaergic neurotransmission inhibits diversely AMPA and NMDA receptor mediated network dynamics in cortical cultures: A model-driven experimental study

### Heidi Teppola, Jugoslava Acimovic, Marja-Leena Linne

#### Tampere University of Technology, Faculty of Biomedical Sciences and Engineering, Tampere, Finland

##### **Correspondence**: Heidi Teppola (heidi.teppola@tut.fi)

*BMC Neuroscience* 2018, **19(Suppl 2):**P137

Network burst activity promoted by neuronal populations plays a fundamental role in the formation of a functional network during early development of the neocortex [1]. The synchronous periodic patterns of activity are observed in cerebral cortex in vivo, in cortical slice preparations in vitro, and in dissociated in vitro cortical cell cultures [1, 2]. In dissociated cell cultures, the network burst (NB) activity is driven by excitatory neurotransmission, which is primarily mediated by the action of glutamate on two types of glutamatergic ionotropic receptors AMPA and NMDA. Inhibitory neurotransmission is mediated by the action of GABA on GABAergic receptors. GABAergic neurotransmission is thought to control the dynamic pattern formation in neuronal networks by organizing spatially and temporally the network activity rather than only reducing firing probability. The complex interplay and contribution of the excitatory and inhibitory receptors emerging on the level of network dynamics is, however, not well understood [3]. The aim of this study is to examine the diverse role of fast GABA_A_ receptors on shaping the fast AMPA receptor and the slow NMDA receptor mediated recurrent excitatory neurotransmission in initiating, maintaining and terminating the network wide bursts dynamics in three weeks’ old dissociated postnatal rat cortical cultures. In order to study the role of GABA_A_ receptors on AMPA and NMDA receptor driven network burst (NB) structures, the extracellular activity was systematically recorded with microelectrode array technique under several combinations of receptor antagonists such as I. mature control cultures without pharmacology, II. with partial AMPA receptor suppression (NBQX), III. with partial NMDA receptor suppression (D-AP5), IV. with GABA_A_ receptor suppression (PTX), V. disinhibited cultures from II with PTX, and VI. disinhibited cultures from III with PTX. The NB structures are analyzed as burst measures from the detected NBs such as burst length, rising and falling phase, maximum firing rate and burst-size as well as the electrode recruitment at time. We show the diverse actions of GABA_A_ receptors on shaping the NB structure and overall network dynamics. The action of GABA is shown to dampen the termination of the slowly recruited NBs in NMDA mediated cultures and to dampen the initiation of faster recruited NBs in AMPA mediated NBs in cultures at the end of the third week in vitro. The here presented results can be used to fine-tune data-driven computational network level models of in vitro cell cultures. Well-validated network models can help address the altered involvement of excitatory and inhibitory receptors in cognitive disorders such as schizophrenia and Alzheimer’s disease.

**Acknowledgement**

Central fund of Finnish Cultural Foundation and Finnish Brain Foundation sr.

**References**

1. Luhmann HJ, Sinning A, Yang JW, Reyes-Puerta V, et al.Spontaneous Neuronal Activity in Developing Neocortical Networks: From Single Cells to Large-Scale Interactions. *Front Neural Circuits* 2016 10, 40
2. Teppola H, Okujeni S, Linne M-, Egert U.AMPA, NMDA AND GABAAreceptor mediated network burst dynamics in cortical cultures in vitro. In:*Eighth International Workshop on Computational Systems Biology*, *WCSB 2011*, *June 6*–*8*, *Zurich*, *Switzerland*. *TICSP series* 2011, 181–184
3. Fong MF, Newman JP, Potter SM, Wenner P.Upward synaptic scaling is dependent on neurotransmission rather than spiking. *Nat Commun*. 2015, 6, 6339

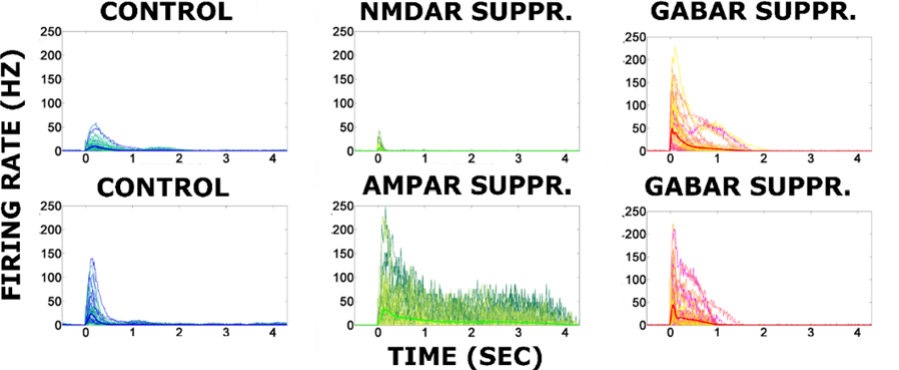
**Examples of network-wide firing rate burst profiles [Hz]. Row one:** The data of control recording (blue) in comparison to the data of NMDA-R (green) and NMDA-R with GABA_A_-R (red) suppression. **Row two:** The data of the control recording (blue) in comparison to the data of the recording condition when AMPA-R (green), and AMPA-R with GABA_A_-R (red) suppression. Thin lines represent individual network-wide burst profiles, whereas the thick line represents the average over all. The results show that the maximum firing rate decreases and the late state of the burst profile disappears after NMDA-R suppression. Furthermore, results demonstrate that the maximum firing rate increases and network-wide bursts lengthens after disinhbition. Firing rate maintains at a higher level for extended time period after AMPA-R suppression. In addition, the duration of burst profile shortens after disinhibition.

## P138 A neural mass model to predict electrical stimulation evoked responses in human brain

### Ishita Basu^1^, Britni Crocker^1^, Kara Farnes^1^, Madeline Robertson^1^, Angelique Paulk^1^, Darin Dougherty^1^, Sydney Cash^1^, Emad Eskandar^1^, Alik Widge^1^, Mark Kramer^2^

#### ^1^Massachusetts General Hospital, MA, United States; ^2^Boston University, Mathematical Neuroscience, Boston, MA, United States

##### **Correspondence**: Ishita Basu (ibjuslc@gmail.com)

*BMC Neuroscience* 2018, **19(Suppl 2):**P138

Deep brain stimulation (DBS) is a valuable tool for ameliorating drug resistant pathologies such as movement disorders and epilepsy. DBS is also being considered for complex neuro-psychiatric disorders, which are characterized by high variability in symptoms and slow responses that hinder DBS setting optimization. Experimental opportunities with human subjects are too limited to fully explore the stimulation space and design new stimulation approaches. In this work we developed an in-silico environment to examine the effects of electrical stimulation in regions neighboring a stimulated brain region. We used the Jansen-Rit neural mass model of single and coupled brain regions to simulate the response to a train of electrical current pulses at different frequencies (10–160 Hz) on the local field potential recorded in the amygdala and cortical structures in human subjects. We found that using a single region model, the evoked responses could be accurately modeled following a narrow range of stimulation frequencies. Including a second coupled region increased the range of stimulation frequencies whose evoked responses could be efficiently modeled. This modeling framework provides an environment to explore, safely and rapidly, a wide range of stimulation settings not possible in human brain stimulation studies. The model can be trained on a small dataset of stimulation responses to develop an optimal stimulation strategy for an individual patient (Fig. 1).

**Fig. 1:**
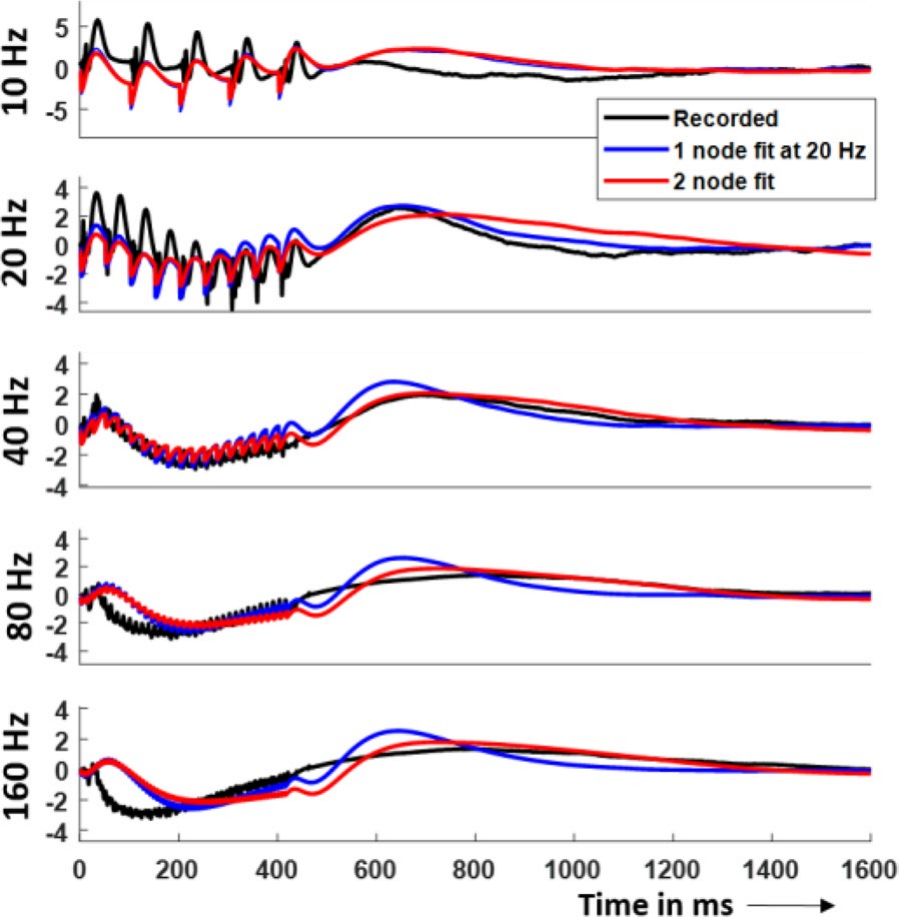
Recorded and simulated response to electrical stimulation in the amygdala. The stimulation was at 10–160 Hz, 6 mA. The simulation was done using a 1-region and 2-region Jansen-Rit neural mass model

## P139 Missing data for an electrodiagnostic nerve test

### James Bell^1^, Kelvin Jones^2^, Martha White^3^

#### ^1^University of Alberta, Departments of Neuroscience and Computing Science, Edmonton, Canada; ^2^University of Alberta, Faculty of Kinesiology, Sport, and Recreation, Edmonton, Canada; ^3^University of Alberta, Department of Computing Science, Edmonton, Canada

##### **Correspondence**: James Bell (jbell1@ualberta.ca)

*BMC Neuroscience* 2018, **19(Suppl 2):**P139

Neuroscientists make use of a wide variety of techniques for dealing with the problem of missing data. Some techniques are simple and in common use, such as adaptive*n*, listwise deletion (i.e. complete case analysis), mean substitution, or last observation carried forward (LOCF). Others come from the fields of statistics or machine learning, such as multiple imputation or alternating least squares (ALS). Yet others are ad hoc, designed by experts based on their knowledge of their specific data. In many cases, the choice of a missing data technique appears to be based on following past practices. While continuity with past research is important, significant improvements in results may be observed when empirically or theoretically proven techniques are used.

In this study, we compare five missing data techniques using an electrodiagnostic nerve test dataset. This electrodiagnostic nerve test, used clinically and for research, consists of five waveforms which are often summarized in about thirty key measurements. Since the test has not yet been used with matrix-based machine learning algorithms, past studies have most often used adaptive*n*, assuming missing values are missing completely at random. However, as international users of this test begin to combine datasets and collaborate with the aim to create a large international database of normative results, there is an increasing need for a systematic approach to missing data, which this study aims to meet. The five missing data techniques were chosen because they are in common use in neuroscience and clinical studies (mean substitution and complete case analysis) or because they are well established in statistics and machine learning (multiple imputation, matrix completion with ALS, and a linear regression predictor). The goal was to determine which techniques provided the best fit for both missing values and the overall variances and covariances. We found that matrix completion’s performance included much larger variance than the other methods, though it was not significantly different. Listwise deletion is unable to fill missing values and its performance in filling the covariance matrix was significantly worse than linear regression and multiple imputation. Linear regression performed better than mean substitution (*p*val = 0.014, *p*cov < 0.001) but not as well as multiple imputation (*p*val < 0.001, *p*cov < 0.001). The results are shown in Fig. 1. Multiple imputation is an effective method of filling missing data in this electrodiagnostic nerve test dataset. Linear regression also performs adequately, but the other methods should be avoided. We believe that these results, which are consistent with other literature, will generalize to other matrix datasets in neuroscience.

**Fig. 1:**
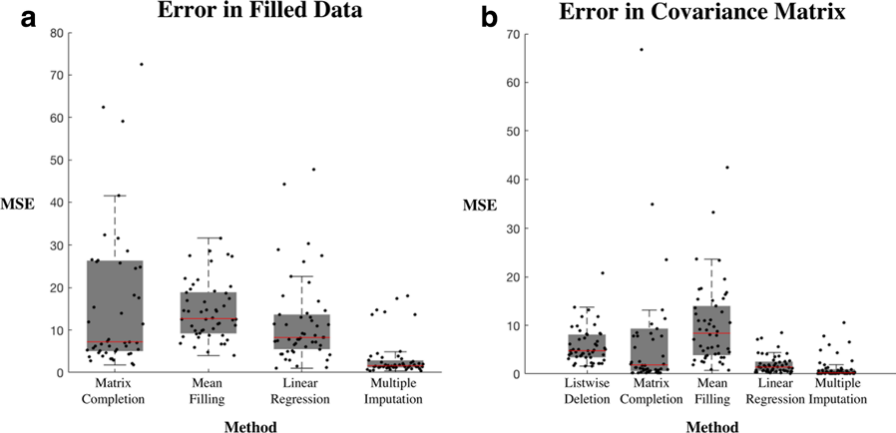
Error rates for each method when filling (A) the value matrix and (B) the covariance matrix. Outliers for matrix completion exist well beyond the bounds of the figures

## P140 Neural model of the multi-stable dynamics of the perception of body motion

### Leonid Fedorov^1^, Tjeerd Dijkstra^2^, Louisa Sting^3^, Howard Hock^4^, Martin Giese^5^

#### ^1^International Max Planck Research School for Cognitive and Systems Neuroscience, Tuebingen, Russian Federation; ^2^University of Tübingen, Dept of Cognitive Neurology, Tübingen, Germany; ^3^University of Tübingen, GTC & International Max Planck Research School, Tübingen, Germany; ^4^Florida Atlantic University, Center for Complex Systems and the Brain Sciences, Boca Raton, FL, United States; ^5^Center for Integrative Neuroscience & University Clinic Tübingen, Dept of Cognitive Neurology, Tuebingen, Germany

##### **Correspondence**: Leonid Fedorov (leonidf87@gmail.com)

*BMC Neuroscience* 2018, **19(Suppl 2):**P140

Multi-stable perception refers to the association of the same visual stimulus with multiple alternative percepts. So far multi-stability has been studied primarily in the context of low-level vision and shape recognition. Multi-stability has also been observed during the perception of body motion, especially if the associated depth information is ambiguous [1]. In this case the same action stimulus is associated, for example, with multiple alternative walking directions. In psychophysical experiments it has been demonstrated that body motion perception can show spontaneous perceptual switching between different interpretations and hysteresis, if a stimulus parameter is gradually varied that introduces a bias for one of the two perceptual interpretations. We present a physiologically-inspired neural model that provides a unifying account for this perceptual multi-stability and multiple psychophysical experiments that characterize the underlying perceptual dynamics. Our model includes the following parts: (1) a deep neural hierarchy that recognizes body shapes from silhouette features and shading gradients of the moving figure; (2) a fast dynamic neural layer that can be interpreted as 2D neural field whose dimensions encode the stimulus view and the temporal order of the body shapes within action sequences; (3) a slower bistable read-out network that pools neural responses over the body shapes belonging to the same action and view over time-points. Our model provides a unifying account for a number psychophysical results from the literature, and from our own experiments: (a) dependence of percept probabilities on shading cues, (b) illusory misperception of walking direction for body stimuli that are illuminated from below [2]; (c) perceptual hysteresis for the gradual variation of disambiguating shading cues of body motion stimuli [3]. Our results show that the multi-stability of body motion perception can be accounted for in a simple way by the interaction between deep example-based neural networks for the recognition of body shapes and an elementary physiologically plausible cortical activation dynamics (Fig. 1).

**Fig. 1:**
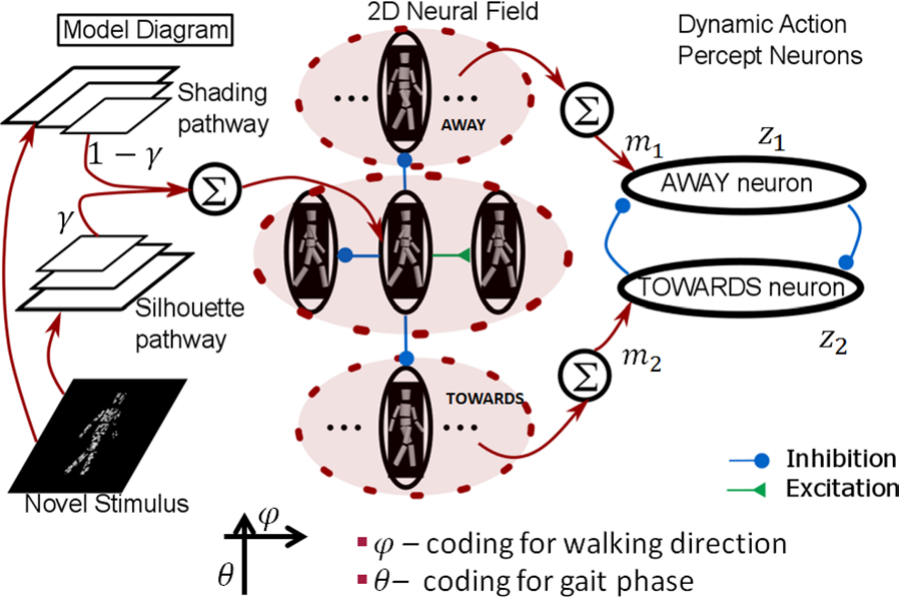
Schematic illustration of model architecture. Two feedforward pathways code for body snapshots at the ~ 70–150 ms timescale. Their outputs are combined to drive the non-autonomous dynamic neural field that codes for stimulus view (with local excitation, mid-range inhibition) and gait phase (forward excitation, backward inhibition) at full gait cycle time scale (~ 1.2–1.5 s). A final bistable read-out network represents perceptual dynamics at the ~ 2–10 s timescale and exhibits perceptual hysteresis

**References**

1. Vanrie J, Verfaillie K. Perceiving depth in point‐light actions” *Perception and Psychophysics* 2006, 68: 601‐612.
2. Leonid A, Fedorov T, Dijkstra MH, Giese MA. Lighting-from-above prior in biological motion perception *Scientific Reports* 2018, 8, 1507
3. Sting L, Fedorov LA, Dijkstra TMH, Hock H, Giese MA. Dynamics of multi stable biological motion perception. *VSS Conference 2017*, 19–24 May, St.Petersburg, Florida .

## P141 Detecting and classifying neocortical population codes via deep artificial neural networks

### Christopher Endemann, Matthew Banks

#### University of Wisconsin, Department of Anesthesiology, Madison, WI, United States

##### **Correspondence**: Christopher Endemann (endemann@wisc.edu)

*BMC Neuroscience* 2018, **19(Suppl 2):**P141

In order to build more accurate models of the neural circuitry and algorithms underlying learning, memory, and perception, it is imperative to properly characterize and identify the response properties and firing patterns of the neocortex across a multitude of experimental manipulations–allowing for a subsequent identification of the roles that different neocortical information traces play in orchestrating and facilitating our conscious experiences. Related to this mission, it is often useful to record neocortical firing patterns via laminar probe electrodes–which span the ubiquitously layered structure of the neocortex, and allow for an in vivo investigation of the response characteristics of individual feature-sensitive cortical columns–the computational units which make up the cortical hierarchy. One general pattern of spiking activity that is readily observed from such recordings involves elevated firing rates of cells spanning multiple layers of the cortical column. Such population-level firing events are often referred to as network burst events, or “UP” states, and are observed to occur in response to a given cortical column’s preferred stimulus feature as well as spontaneously. While past attempts have aimed to characterize the structure and function of these network burst events, such analyses have been limited, in part, by an overly generalized definition of network burst events; whereby such events are defined simply as periods of elevated firing rates spanning multiple neocortical layers. While useful in some contexts, this definition ignores the possibility that multiple activation profiles (and functionalities) may underlie different network burst events, and also makes it difficult to precisely define boundaries of burst events occurring among other independent spiking events or among trains of successive burst events. Acknowledging these limitations in studying network burst events, our goal was to engineer an analysis pipeline capable of detecting and discriminating between specific patterns of population spiking activity. This approach was chosen to allow researchers to define network burst events in a manner that implicitly includes spatiotemporal structure as a defining characteristic–while also providing a new methodological approach for defining and detecting other forms of population codes that occur throughout the brain. To meet our goal, we treated burst detection as an image-recognition problem, and trained a deep convolutional neural network to detect a specific activation pattern of spikes occurring across the cortical laminae during burst-like periods of activity. As a golden standard for learning this structure, isolated burst events emerging during quiescent periods were extracted from laminar probe data recorded from rat auditory cortex, and subsequently used to train a deep neural network to discriminate between the structure of such network burst events and other forms of firing activity (e.g. burst-like activity with shuffled spikes to remove spatiotemporal structure). This trained network was then applied to over a dozen laminar probe datasets recorded from different rats and different regions of auditory cortex in order to characterize the true, stereotyped structure of network burst events, as well as to characterize the effect of anesthesia on such events.

## P142 Blind recovery of transient responses with higher-order spectra

### Christopher Kovach^1^, Hiroto Kawasaki^2^, Matthew Howard^2^

#### ^1^University of Iowa, Caltech, Iowa City, IA, United States; ^2^University of Iowa Hospitals and Clinics, Neurosurgery, Iowa City, IA, United States

##### **Correspondence**: Christopher Kovach (christopher-kovach@uiowa.edu)

*BMC Neuroscience* 2018, **19(Suppl 2):**P142

The common approach to studying brain responses at macro- and mesoscopic scales relates some physiological measure, such as EEG, LFP or BOLD response, to the timing of external driving events. In this mode of inference (call it mode S), the stimulus is abstracted into a discrete set of event times associated with a typically limited number of alternative conditions, which, in effect, encode a low-dimensional parameter space within the stimulus. With microscale single- and multi-unit data, by contrast, it is common to elicit receptive fields over high-dimensional stimulus spaces, which might, for example, encode detailed spatial or spectro-temporal properties of the stimulus. In this mode of inference (call it mode R), the response, rather than the stimulus, is abstracted to a low-dimensional form (“spikes’’), facilitating the recovery of detailed receptive fields properties. Macro- and mesoscopic recordings are less amenable to mode R inference because few similarly unambiguous response elements are at hand. We describe here a novel strategy for overcoming this barrier which takes advantage of properties of higher order spectra. This approach recovers transient signal features that best approximate the bispectrum. The signal may then be characterized according to the occurrence times of the transients. Although demonstrated in its application the bispectrum, the extension to other higher order spectra is straightforward. The approach is validated through the blind identification of auditory evoked responses in human intracranial recordings from both controlled stimuli (click trains) and uncontrolled ecologically meaningful stimuli (a video soundtrack) with no prior information about the stimulus. In human intracranial data recorded from 17 epilepsy patients, the distribution of transient responses modulated by click-train stimuli was compared to that of traditionally computed evoked responses. For all patients with recordings in the vicinity of primary auditory cortex on Heschl’s gyrus, both the blindly recovered transient responses and normally computed evoked responses showed significant modulation by the stimulus, defined as one or more time points at which the response reached FDR corrected q < 0.01 significance. In most cases, a close correspondence was observed between the form of the transient and the evoked response, demonstrating the blind recovery of the evoked response. At locations on lateral temporal cortex, significant stimulus-driven modulation was observed less frequently than evoked responses, and the forms of recovered transients tended to differ from the evoked waveforms, implying that low-level acoustic stimulation was not the primary driver of responses in these areas.

Mode R inference with spectro-temporal properties of ecologically meaningful auditory speech stimuli revealed a gradation of the temporal response window along the medial-to-lateral axis of Heschl’s gyrus: transients recorded from lateral cortex responded to complex acoustic stimulation with a more prolonged window of integration. While medial responses were associated with low-level transient acoustic properties of the stimulus, lateral responses tended to accompany the onsets and transitions of phrases within speech stimuli. Responses in neither area showed clear evidence of spectral selectivity. These results validate the technique and demonstrate its ability to reveal new aspects of cortical auditory processing.

## P143 Early spontaneous activity predicts structural changes in layout of orientation domains during early development

### Bettina Hein^1^, Sigrid Trägenap^1^, David Whitney^2^, Gordon Smith^3^, David Fitzpatrick^2^, Matthias Kaschube^1^

#### ^1^FIAS, Department of Neuroscience, Frankfurt, Germany; ^2^Max Planck Florida Institute, Department of Neuroscience, Jupiter, FL, United States; ^3^University of Minnesota, Department of Neuroscience, Minneapolis, MN, United States

##### **Correspondence**: Bettina Hein (hein@fias.uni-frankfurt.de)

*BMC Neuroscience* 2018, **19(Suppl 2):**P143

Orientation preference is a prominent feature of the mammalian visual cortex, but the mechanisms underlying its development remain elusive. Spontaneous cortical activity is thought to play an important role in the maturation of orientation selectivity [1], yet it is still unclear how spontaneous activity shapes this process.

Here we address this question by combining chronic imaging experiments and computational analyses. We used GCaMP6 s to image visually evoked responses with moving grating stimuli and spontaneous activity in ferret primary visual cortex, from four days prior to the natural time of eye-opening (~ P30 in ferrets) until about a week after eye-opening. Prior to P30, the eye lids were transiently opened when probing the cortex with grating stimuli. This setup allows us to assess the emergence and refinement of orientation selectivity and its relationship to spontaneous activity during early development. We found that, already at the earliest time point measured (P26), visual stimuli robustly evoke responses that are modular (i.e., patchy), extend over the whole field of view, and are highly variable across trials. Typically, weak orientation tuning is evident at this stage, but its layout only coarsely resembles the mature organisation. Indeed we observe considerable reorganisation until a near mature layout is reached a few days after eye-opening. Intriguingly, we find signatures of the mature layout of orientation domains that are not yet present in its early layout, but in the spatial structure of early spontaneous activity. This is revealed by calculating the partial correlation coefficients between the future orientation preference map and the early spontaneous activity patterns while controlling for the correlation with the early orientation preference map. Thus, early spontaneous activity appears to contain information about the mature layout of orientation preference, suggesting a role in refining its organisation. To determine if early spontaneous activity could indeed drive the refinement of orientation preference, we built a correlation-based model of early visual cortex, assuming that locations with positively correlated spontaneous activity become more similar in their tuning at later stages in development, whereas negatively correlated locations become more dissimilar. Using the measured spontaneous correlation structure and layout of orientation preference in the early cortex, our model predicts aspects of the subsequent reorganization of orientation preference towards its mature layout. The model predicts that spontaneous activity and the orientation preference map become increasingly similar in their layout during development and we confirm this prediction with our experimental data. Thus, this correlation-based approach captures the refinement of orientation preference as well as of its relationship to spontaneous activity. We conclude that early cortical spontaneous activity plays a significant role in driving the refinement of the circuits underlying orientation preference in visual cortex.

**Reference**

1. B Chapman, MP Stryker. Development of orientation selectivity in ferret visual cortex and effects of deprivation. *J Neurosci*. 1993, 13 (12).

## P144 Multispike Tempotron performance under different task-related neural spiking statistics

### Hannes Rapp^1^, Martin Paul Nawrot^2^, Merav Stern^3^

#### ^1^University of Cologne, Computational Systems Neuroscience/Animal Physiology, Cologne, Germany; ^2^University of Cologne, Zoological Institute, Germany; ^3^University of Washington, Applied Mathematics, Seattle, WA, United States

##### **Correspondence**: Hannes Rapp (hannes.rapp@smail.uni-koeln.de)

*BMC Neuroscience* 2018, **19(Suppl 2):**P144

The Multispike Tempotron [4] is a synaptic-like model learning rule for spiking neurons. It is trained to elicit a precise number of spikes in response to a sequence of temporally precise presynaptic spike patterns that is embedded in background spiking activity. Each individual pattern elicits a specific number of spikes. As teaching signal, only the total number of output spikes for the complete input sequence is used. Thus, for successful learning, the neuron is required to arrange the total number of output spikes in time such that the occurrence times of the patterns are matched. The Multispike Tempotron model in [4] used the homogeneous Poisson process, the commonly used stochastic model for single neuron spiking statistics in the neocortex, to simulate both, the embedded patterns and the background noise. However, the Poisson process is a mathematically convenient but deficient model for the spiking statistics of cortical neurons, which is likely more regular than Poisson [1]. Moreover, large and task-related trial-by-trial variability [2–3] implies changing network conditions. In this work we examine how different spike train statistics of presynaptic input that deviate from Poisson, impact the learning and generalization capabilities of the Multispike Tempotron.

**References**

1. Nawrot MP, Boucsein C, Rodriguez MV, et al. Measurement of variability dynamics in cortical spike trains. *Journal of Neuroscience* 2008, 169(2), 374–390.
2. Rickert J, Riehle A, Aertsen A, et al. Dynamic encoding of movement direction in motor cortical neurons. *Journal of Neuroscience* 2009, 29(44), 13870–82
3. Riehle A, Brochier T, Nawrot MP, Gruen S. Behavioral context determines network state and variability dynamics in monkey motor cortex. *BioRxiv* 2017
4. Gutig R, Sompolinsky H. The tempotron: A neuron that learns spike-based decisions. *Nat*. *Neuro*. 2006, 9(3), 420–428.

## P145 Modeling mouse visual cortex

### Michael Oliver, Gabriel Ocker, Peter Ledochowitsch, Nicholas Cain, Saskia E.J. de Vries, Michael A. Buice

#### Allen Institute for Brain Science, Modelling, Analysis and Theory, Seattle, WA, United States

##### **Correspondence**: Michael Oliver (michael.d.oliver@gmail.com)

*BMC Neuroscience* 2018, **19(Suppl 2):**P145

Building predictive models of neural activity is a central aim of computational neuroscience. To better understand the various representations in mouse visual cortex, we aim to build stimulus–response models for neurons in cortical areas VISp, VISl, VISal, VISrl, VISpm, and VISam. Data from these brain areas was collected as part of the Allen Brain Observatory at the Allen Institute for Brain Science. The Allen Brain Observatory dataset includes data from hundreds of animals exposed to a standardized set of visual stimuli while calcium fluorescence was recorded from a cortical visual area. However, despite this large dataset, learning the stimulus–response mapping function for individual neurons is a challenging problem. The chief difficulty is that stimulus–response mapping functions are inherently high-dimensional and nonlinear while response data for a single neuron is limited by experimental demands and intrinsically noisy. One of the most successful approaches for tackling this problem is to linearize the stimulus–response mapping by preprocessing the stimulus with a carefully chosen nonlinear transform. For our model, we constructed a nonlinear transform using a pyramid of spatio-temporal Gabor filters (along with appropriate nonlinearities) to create a set of model “simple” and “complex” cells that densely tile the stimulus [1]. We then use regularized regression to find a linear mapping between the transformed stimuli and the estimated responses of each recorded neuron. Neural responses were estimated from the differential fluorescence traces using an L0 regularized deconvolution algorithm [2]. Before fitting the models, we separated the stimuli used in the Allen Brain Observatory into two classes, ‘natural’ and ‘artificial’. ‘Natural’ stimuli included natural movies and images. ‘Artificial’ stimuli included static gratings, drifting gratings and locally sparse noise. We fit models in these two stimulus regimes separately in order to determine which type of stimulus best allows for the construction of predictive stimulus–response models. We find that models fit using natural stimuli both generalize better and are more interpretable. We also find that across all cortical visual areas the fit models tended to utilize far more “complex cell” regressors than “simple cell” regressors. This suggests that neural representations in mouse cortex, even in the first cortical visual area VISp, contain few if any true “simple cells” and may exhibit higher-order tuning earlier in their visual hierarchy than other species, such as cats and primates.

**References**

1. Nishimoto S, Vu AT, Naselaris T, et al. Reconstructing visual experiences from brain activity evoked by natural movies. *Curr Biol*. 2011, 21(19), 1641–1646.
2. Jewell S, Witten D. Exact spike train inference via l Optimization. *arXiv* 2017,

## P146 On the correspondence between receptive fields derived from spikes versus calcium

### Peter Ledochowitsch^1^, Nicholas Cain^1^, Joshua Siegle^1^, Xiaoxuan Jia^2^, Michael Oliver^1^, Ulf Knoblich^3^, Lawrence Huang^3^, Brian Hu^1^, Gabriel Ocker^1^, Daniel Millman^1^, Séverine Durand^1^, Ramakrishnan Iyer^1^, Lu Li^3^, Shawn Olsen^1^, R Clay Reid^1^, Hongkui Zeng^1^, Stefan Mihalas^1^, Saskia E.J. de Vries^1^, Michael A. Buice^1^

#### ^1^Allen Institute for Brain Science, Modelling, Analysis and Theory, Seattle, WA, United States; ^2^Allen Institute for Brain Science, Neural Coding, Seattle, WA, United States; ^3^Allen Institute for Brain Science, Celltypes, Seattle, WA, United States

##### **Correspondence**: Peter Ledochowitsch (peterl@alleninstitute.org)

*BMC Neuroscience* 2018, **19(Suppl 2):**P146

Historically, most studies of receptive fields were undertaken using electrophysiology, with a recent shift towards calcium imaging as a method of choice. Electrophysiology and Ca2+ imaging both constitute noisy observations of the underlying neural activity. However, the statistical properties of the raw signals, as well as the sources and effects of the introduced noise, are quite distinct. To interpret receptive fields derived from Ca2+ imaging in the context of existing literature, it is crucial to understand how these differences are reflected in the detectability and properties of receptive fields derived from different recording modalities, for it is impractical to perform every experiment with each modality. To address this issue, we first computed receptive fields from extracellular spikes recorded using Neuropixels [1] silicon probes in three visual regions, directly, using the spike-triggered average (STA). Then, we calibrated a biophysically inspired model that relates spiking activity to observed fluorescence (MLSpike, [2]) on ‘ground truth’ data, in vivo Ca2+ recordings paired with juxtacellular electrophysiology, where the Ca2+ -dependent fluorescence was consistent with the Allen Brain Observatory (http://observatory.brain-map.org/visualcoding/), a public resource providing standardized in vivo characterization of single neuron activity in mouse visual cortex based on Ca2+ -imaging. Following calibration, we computed model calcium activity from above spike trains, and analyzed the synthesized Ca2+ data using techniques developed for mapping of classical receptive fields based on responses to locally sparse noise in the Allen Brain Observatory data processing pipeline. We found that such analysis readily yielded receptive fields, which largely agreed with those identified directly from the electrophysiological recordings via STA, and investigated the sensitivity of the obtained receptive field structure to the parameterization of the Ca2+ forward model. We observed that statistical detectability and the quality of the correspondence is dependent on several factors. (1) Quality of the synthetic calcium data: low signal-to-noise ration results in worse correspondence and detectability. (2) Event detection strategy: the advantage of sophisticated event detection strategies (e.g. L0 event detection [3]) is most pronounced with the calcium signal to noise ratio is low. (3) Region-specific properties of the spiking response (e.g. potentially degree of burstiness, sparseness). Generally, correspondence and detectability were better in primary visual cortex than in higher visual areas.

In the future, this data-driven modeling approach may provide a Rosetta Stone for receptive field comparison across recording modalities, as well as inspire improvements to algorithmic receptive field fitting procedures.

**References**

1. Jun JJ, Steinmetz NA, Siegle JH, et al. Fully integrated silicon probes for high-density recording of neural activity. *Nature* 2017, 551, 232. Retrieved from http://dx.doi.org/10.1038/nature24636
2. Deneux T, Kaszas A, Szalay G, et al. Accurate spike estimation from noisy calcium signals for ultrafast three-dimensional imaging of large neuronal populations in vivo. *Nature Communications* 2016, 7, 12190 EP -.http://doi.org/10.1038/ncomms12190
3. Jewell S, Hocking TD, Fearnhead P, Witten D. Fast Nonconvex Deconvolution of Calcium Imaging Data. *arXiv* 2018. https://arxiv.org/abs/1802.07380

## P147 The structure of population activity and coding in mouse visual cortex

### Gabriel Ocker, Peter Ledochowitsch, Daniel Millman, Michael Oliver, Nicholas Cain, Saskia E.J. de Vries, Michael A. Buice

#### Allen Institute for Brain Science, Modelling, Analysis and Theory, Seattle, WA, United States

##### **Correspondence**: Gabriel Ocker (gabeo@alleninstitute.org)

*BMC Neuroscience* 2018, **19(Suppl 2):**P147

How different neuronal populations represent and transmit information about the external world is a central question of systems neuroscience. The Allen Brain Observatory contains the results of two-photon calcium imaging from populations of hundreds of neurons in awake mice passively viewing visual stimuli. Its recordings span layers two through six in areas V1 and the secondary areas LM, AL, PM, AM and RL and are in response to both artificial and naturalistic stimuli. This dataset offers a powerful new tool for examining population coding in the mouse visual system. To assess information processing across visual areas and layers, we examined how well different visual stimuli can be decoded from the population’s trial-by-trial responses, comparing different visual areas and layers. We found that visual stimuli can overall be best decoded from layers 2/3 and 4 in areas V1 and LM, with different decoding performance in genetic lines (Cre lines) labelling different populations of cells. We then asked whether the structure of trial-by-trial correlations between neurons (“noise correlations”) was optimal for decoding. We found stimulus-specific and Cre line-specific effects of noise correlations on decoding. Noise correlations almost always improved the decodability of natural scenes, except in layer five-specific lines in area LM. In contrast, noise correlations decreased the decodability of drifting grating direction in most areas and Cre lines, but increased the decodability of drifting gratings in certain excitatory populations. These demonstrate differential modes of population encoding for different stimulus classes and by different neural populations. Thus motivated, we further examine the structure of noise correlations across neurons in these different layers and visual areas. We show universal patterns in how noise correlations depend on stimulus tuning and distance between neurons, and in the dimensionality of population activity.

## P148 Online biologically plausible decoding of clusters in retinal population activity

### Adrianna Loback, Michael Berry

#### Princeton University, Department of Neuroscience, Princeton, NJ, United States

##### **Correspondence**: Adrianna Loback (arl64@cam.ac.uk)

*BMC Neuroscience* 2018, **19(Suppl 2):**P148

An appealing recent principle for neural population codes is that correlations among neurons organize neural activity patterns into a discrete set of clusters, which can each be viewed as a noise-robust population codeword. Previous work supports that this clustering principle—which can in theory offer a neural code numerous advantages—applies to the retinal ganglion cell population (RGC) code. However, the previous approaches used to identify these latent clusters from data involved methods that are not biologically plausible. For example, these methods are restricted to an offline setting. Here, we combine experimental and theoretical methods to address the question of how downstream processing areas, such as primary visual cortex, could extract the previously identified noise-robust clusters. Based on recent results on the structure of clusters of the retinal ganglion cell population code, we investigated the use of a spiking neural network model with feedforward, activity-dependent plasticity that represents an implicit generative model whose latent states correspond to distinct combinations of neuronal activities. Importantly, learning and decoding of clusters with this type of network model can be implemented online. We found that the readout neurons of this neural network model developed, after learning on real RGC population response data, strong tuning for the latent clusters identified by our previous, non-biologically plausible machine learning method for the same data. This learned specificity for the previously-identified RGC population clusters was statistically significant when compared to control networks. To our knowledge, these results are the first to demonstrate the existence of a biologically plausible neural network model (in particular, unsupervised learning and decoding can be done in real time) that is capable of decoding clusters from real RGC population activity data that were previously identified using non-biologically plausible, sophisticated machine learning methods.

## P149 Towards a computational account of theta band (4–8 Hz) power modulation in the subthalamic nucleus during response conflict condition

### Prannath Moolchand^1^, Stephanie Jones^1^, Michael Frank^2^

#### ^1^Brown University, Department of Neuroscience, Providence, RI, United States; ^2^Brown University, Department of Cognitive, Linguistic & Psychological Sciences, Providence, RI, United States

##### **Correspondence**: Prannath Moolchand (prannath_moolchand@brown.edu)

*BMC Neuroscience* 2018, **19(Suppl 2):**P149

The Subthalamic Nucleus (STN) plays a fundamental role in arresting automated responses during response conflict. It prevents premature activation of the output of the Basal-Ganglia (BG) to buy the Executive-Control (EC) system in prefrontal cortex (PFC) time to resolve the conflict and elicit more appropriate behaviors. Experiments in primates have shown that theta band (4–8 Hz) power in the Local Field Potential (LFP) and spike rates in the STN increase commensurate with the level of conflict. Moreover, recent lines of evidence suggest that the STN can act as a conflict detector by integrating competing motor signals to prevent impulsive responses. Adapting prior cellular models of STN and Globus Pallidus externus (GPe), we have built a novel large-scale biophysically constrained and reciprocally coupled subthalamopallidal (STN- GPe) network. We perturb the network with simulated cortical signals representing competing motor actions to understand the electrophysiological basis of the STN signal modulations and how cortico-STN topography impact these computations. Our results show a balance between intrinsic behaviors of the STN-GPe network and specific patterns of cortical drive is necessary for theta band expression in the network. We conjecture that theta-dependent increased spiking in the STN network is the key component for “braking” unwanted impulsive responses.

## P150 Mediodorsal thalamus permits cognitive flexibility by suppressing conflicting prefrontal representations

### Rajeev Rikhye, Ralf Wimmer, Michael Halassa

#### Massachusetts Institute of Technology, Brain and Cognitive Sciences, Cambridge, MA, United States

##### **Correspondence**: Rajeev Rikhye (rvrikhye@mit.edu)

*BMC Neuroscience* 2018, **19(Suppl 2):**P150

Cognitive flexibility is a critical executive function, defined by the ability to adapt goal-directed behaviors to changes in the environment. One of its central components is the ability to prioritize relevant stimuli while ignoring conflicting representations. Despite their importance, the computational principles underlying cognitive flexibility are poorly understood. To examine these neural mechanisms, we developed a novel rule-guided, two-alternative forced choice sensory attention task in which mice flexibly used two different cue sets (an auditory or a visual cue set) to infer a rule (either attend to an auditory or a visual target). Mice performed the task in blocks where they had to switch from using one cue set to another without external cueing. On average, mice performed equally well in either block, regardless of the presentation order. The abrupt transition between the blocks led to an increase in error rate, indicating that mice readjusted to using different sensory cues over a span of approximately 10 trials. We recorded PFC ensemble activity during the task using multi-electrode arrays. A substantial fraction of PFC neurons responded selectively to one of the four cue types through a brief increase in trial-to-trial spiking reliability. In addition, a subset of neurons were selective for rule meaning and were invariant to the cue identity. This finding suggests that the PFC contains a cue set-specific pool of neurons that encode individual cues, and a cue set-invariant pool of neurons that encode rule meaning. How do these cue set invariant responses arise in the PFC? To address this question, we implemented a multi-neuron generalized linear model (GLM) to help infer connectivity profiles within the PFC. Neurons preferring the same cue-set are strongly positively coupled with each other. In contrast, inputs from neurons preferring the other cue set was weak and inhibitory. This suggests that the PFC is functionally organized into two ensembles, each preferring a different cue set. Additionally, cue set-invariant neurons sampled selectively from co-tuned neurons in both cue sets with coupling strengths that varied with trial number relative the onset of the cue set switch. In particular, inputs from co-tuned neurons in the current context are strengthened while those from the opposite context are weakened. We next asked how the mediodorsal thalamus (MD), which is recurrently connected to the PFC, could contribute to this cue set-specific organization of PFC activity. MD neurons responded selectivity for each cue set. Unlike PFC, we could not decode rule-meaning information from the MD, suggesting that the MD exclusively represents task context. Further, our GLM analysis revealed two populations of MD neurons—rate-modulated cells that were negatively coupled to PFC neurons of the opposite context, and temporally-modulated cells that provide a modulatory coupling to PFC neurons that preferred the same context. Therefore, by inhibiting the irrelevant context, MD neurons enhance tuning and selectivity within the PFC. In support of this, transiently suppressing MD neurons reduced PFC tuning strength and increased behavioral inflexibility.

Taken together, our work reveals a critical role for the MD in permitting cognitive flexibility by stabilizing PFC ensembles that are selective for the current sensory context, while suppressing those that are selective for the previous/irrelevant context.

## P151 Long memory in dynamic recurrent networks

### Peter Stratton, Michael Halassa

#### Massachusetts Institute of Technology, Brain and Cognitive Sciences, Cambridge, MA, United States

##### **Correspondence**: Peter Stratton (pstratt@mit.edu)

*BMC Neuroscience* 2018, **19(Suppl 2):**P151

During delay tasks, some neurons in the murine thalamocortical system and hippocampus (‘time cells’) display transient spike responses with timing that is repeated reliably across trials. These transient responses confer a short term memory of the stimulus and context during the delay period, with reliable responses extending to delays of up to 1 s or more. We wondered what neural network structures could facilitate the generation of such dynamic memory patterns. We show that in a simplified formalism of a dynamic recurrently-connected network (DRN), the number of unique dynamic patterns grows exponentially with network size, so that even sparsely firing DRNs of only moderate size can reliably store non-overlapping memories of practically arbitrary duration. Additionally, sparse connectivity allows smaller networks overall (i.e. more neurons but with dramatically reduced connectivity) to generate activity cycles of length comparable to fully connected networks, which is important because as brain networks grow it becomes physically impossible to fully connect them. The DRN formalism emphasises the role in neural function of transient yet repeatable dynamics. Unlike reservoir networks, the connectivity matrix does not need to be finely tuned (random connectivity suffices), and the dynamics implement indefinite (not fading) memory. Activity levels are assumed to be controlled by local inhibition, and gating of input patterns is assumed to be controlled by modulatory signals from thalamus and subcortical structures. In particular, recent experimental evidence suggests that inputs from the MD thalamus convey contextual information and can modulate cortical synaptic strengths. Sparse activity and sparse connectivity, as investigated here, are likely to be critical components in gating of neural signals and the battle against noise-induced degradation of the memory patterns, since sparsity allows weight modulation to have a selective rather than a broad effect on network dynamics. Future work will investigate these mechanisms.

## P152 Stimulus-dependent tuning in cortical area MST of macaques

### Alicia Costalago Meruelo^1^, Stefan Glasauer^1^, Lukas Brostek^1^, Michael J Mustari^2^

#### ^1^Ludwig-Maximilians-Universität München, Dept of Neurology, Munchen, Germany; ^2^University of Washington, Washington Primate Research Center, Seattle, WA, United States

##### **Correspondence**: Alicia Costalago Meruelo (alicia.costalagomeruelo@lrz.uni-muenchen.de)

*BMC Neuroscience* 2018, **19(Suppl 2):**P152

A fundamental goal of neuroscience is the characterization of the relationship between stimuli and neural response. Classical stimuli, such as ramps and sinusoids, have been used because they are straight-forward to analyse and often easy to generate with the equipment available. Although such stimuli are responsible for much of what we know about the tuning properties of sensory neurons, they often do not allow a complete characterization of neural response. In the present work, we recorded extracellular potentials from single neurons in cortical area MST in head-restrained awake monkeys (Macaca mulatta) while presenting classical visual motion stimuli (sinusoids and ramps) and a more naturalistic unpredictable stimulus motion with a band-limited Gaussian white noise (GWN) velocity profile. Visual stimuli were projected on a screen directly in front of the monkey, which was rewarded for following the stimuli by smooth pursuit eye movements. Data was collected from preferred directions eliciting maximal spiking activity. From the neural recordings during GWN stimulus motion we constructed probabilistic multi-dimensional neural tuning functions dependent on retinal and extra-retinal variables such as image and eye velocity and position by an information-theoretic approach [1]. The calculated tuning functions show a gain-field like behaviour with the probability of a spike depending on two or more different explanatory variables. Gain fields have been found in various areas of the brain describing how neurons combine information from more than one source. We then used these tuning functions to predict neural responses to classical sinusoidal and ramp stimuli and compared the predictions to measured responses. The tuning functions derived by the information-theoretic analysis of GWN stimuli where insufficient to fully predict the neural responses to sinusoidal or ramp stimuli in MST.

Our preliminary results indicate that there is a significant difference in the spiking behaviour of individual neurons in the MST between a GWN stimulus in the preferred direction and sinusoidal or ramp stimuli. The differences could be due to tuning specific for a certain class of responses, to missing variables in the characterisation of tuning functions, or to some aspect related to predictability of the signal. Missing variables are one possible explanation, since the amount of data collected usually only allows for 2D tuning functions, while neurons often show tuning to more than two explanatory variables (e.g., retinal slip, eye velocity, and image acceleration). However, it is well-known that the smooth pursuit system contains a predictive mechanism that in case of predictable stimuli allows zero-latency tracking despite the considerable onset delay in the smooth pursuit response. Based on our preliminary results we hypothesize that the found differences in neural responses between predictable and unpredictable reflect the action of this prediction mechanism.

**Acknowledgement**

German Ministry of Research and Education Grant 01GQ1508.

**Reference**

1. Brostek L, Eggert T, Ono S. An Information-Theoretic Approach for Evaluating Probabilistic Tuning Functions of Single Neurons. *Front Comput*. *Neurosci*. 2011, 5, 15.

## P153 Validation and performance of effective network inference using multivariate transfer entropy with IDTxl

### Leonardo Novelli^1^, Patricia Wollstadt^2^, Pedro A.M. Mediano^3^, Joseph Lizier^1^, Michael Wibral^2^

#### ^1^The University of Sydney, Centre for Complex Systems, Sydney, Australia; ^2^Goethe University Frankfurt, MEG Unit, Brain Imaging Centre, Frankfurt am Main, Germany; ^3^Imperial College London, Department of Computing, London, United Kingdom

##### **Correspondence**: Leonardo Novelli (lnov6504@uni.sydney.edu.au)

*BMC Neuroscience* 2018, **19(Suppl 2):**P153

IDTxl is a new open source toolbox for effective network inference from multivariate time series using information theory, available from Github at http://github.com/pwollstadt/IDTxl. The primary application area for IDTxl is the analysis of brain imaging data (import tools for common neuroscience formats, e.g. FieldTrip, are included); however, the toolkit is generic to analysing multivariate time-series data from any discipline and complex system. For each target node in a network, IDTxl employs a greedy iterative algorithm to find the set of parent nodes and delays which maximise the multivariate transfer entropy. Rigorous statistical controls (based on comparison to null distributions from time series surrogates) are used to gate parent selection and to provide automatic stopping conditions for the inference. We validated the IDTxl Python toolkit on different effective network inference tasks, using synthetic datasets where the underlying connectivity and the dynamics are known. We tested random networks of increasing size (10 to 100 nodes) and for an increasing number of time-series observations (100 to 10000 samples). We evaluated the effective network inference against the underlying structural networks in terms of precision, recall, and specificity in the classification of links. In the absence of hidden nodes, we expected the effective network to reflect the structural network. Given the generality of the toolkit, we chose two dynamical models of broad applicability: a vector autoregressive (VAR) process and a coupled logistic maps (CLM) process; both are widely used in computational neuroscience, macroeconomics, population dynamics, and chaotic systems research. We used a linear Gaussian estimator (i.e.Granger causality) for transfer entropy measurements in the VAR process and a nonlinear model-free estimator (Kraskov-Stoegbauer-Grassberger) for the CLM process. Our results showed that, for both types of dynamics, the performance of the inference increased with the number of samples and decreased with the size of the network, as expected. For a smaller number of samples, the recall was the most affected performance measure, while the precision and specificity were always close to maximal. For our choice of parameters, 10000 samples were enough to achieve nearly perfect network inference (> 95% according to all performance measures) in both the VAR and CLM processes, regardless of the size of the network. Decreasing the threshold for statistical significance in accepting a link lead to higher precision and lower recall, as expected. Since we imposed a single coupling delay between each pair of processes (chosen at random between 1 and 5 discrete time steps), we further validated the performance of the algorithm in identifying the correct delays. Once again, 10000 samples were enough to achieve nearly optimal performance, regardless of the size of the network. We emphasise the significant improvement in network size and number of samples analysed in this study, with 100 nodes/10000 samples being an order of magnitude larger than what has been previously demonstrated, bringing larger neural experiments into scope. Nonetheless, analysing large networks with 10000 samples and using the model-free estimators is computationally demanding; therefore, we exploited the compatibility of IDTxl with parallel and GPU computing on high-performance clusters.

## P154 Generative models on accelerated neuromorphic hardware

### Akos Ferenc Kungl^1^, Karlheinz Meier^1^, Sebastian Schmitt^1^, Johann Klahn^1^, Paul Muller^1^, Andreas Baumbach^1^, Dominik Dold^1^, Alexander Kugele^1^, Eric Muller^2^, Christoph Koke^1^, Mitja Kleider^1^, Christian Mauch^1^, Oliver Breitwieser^1^, Maurice Guttler^1^, Dan Husmann^1^, Kai Husmann^1^, Andreas Hartel^1^, Vitali Karasenko^1^, Andreas Grubl^1^, Johannes Schemmel^1^, Mihai A. Petrovici^3^

#### ^1^Heidelberg University, Kirchhoff Institute for Physics, Heidelberg, Germany; ^2^Kirchhoff Institute for Physics, Heidelberg University—Department for Physics and Astronomy, Germany; ^3^Heidelberg University & University Bern, Kirchhoff Institute for Physics & Department of Physiology, Switzerland

##### **Correspondence**: Akos Ferenc Kungl (fkungl@kip.uni-heidelberg.de)

*BMC Neuroscience* 2018, **19(Suppl 2):**P154

Owing to the rapid development of neuromorphic technologies, the emulation of large neuronal networks has recently become feasible [1]. These novel hardware platforms offer advantages compared to classical simulation, such as low power consumption and increased speed, at the cost of limited flexibility and reduced control over the dynamics. A particular scenario in which acceleration plays an important role is Bayesian inference by probabilistic sampling. As an answer to experimental data suggesting that such sampling is realized in the brain at a neuronal level [2, 3], circuit-level theories of sampling with biological neurons have been recently developed [4, 5]. In this work, we present a sampling-network implementation with leaky integrate-and-fire neurons [4] driven by the activity of a decorrelation network [6] on the BrainScaleS neuromorphic platform [7]. The underlying theory allows the training of a population of neurons to approximate target distributions that are either specified explicitly or defined implicitly by data samples. The BrainScaleS system is a spiking, mixed-signal, neuromorphic device running at a speed-up of 104compared to biological real time. The decorrelation network is a deterministic spiking network consisting of self-activating inhibitory neurons with random recurrent connections. By inducing negative correlations in its output spike trains, it counters (positive) shared-input correlations that appear when functional neurons in the sampling network receive random connections from noise-generating sources. This combination of a functional and a noise-generating network allows an implementation that is completely contained in the hardware and can fully benefit from its accelerated dynamics. We used the implemented spiking sampling network to learn various target distributions and perform inference in the associated probability spaces. For example, when trained on a subset of the MNIST dataset, we achieved an accuracy of 93.3 + 0.6–1.3% at a processing rate of 20,000 images per second. As generative models of the learned distribution, our networks were able to complete partially covered patterns and also generate new data without external input (“dreaming’’). Training was done based on the wake-sleep algorithm, with the hardware in the loop: parameter updates were calculated on the host computer using the spiking activity on the neuromorphic device. The ability to train such networks despite a lack of precise parameter control is of particular interest for new nanoelectronic architectures currently under development that make use of analog—therefore inherently imprecise—elements (Fig. 1).

**Fig. 1:**
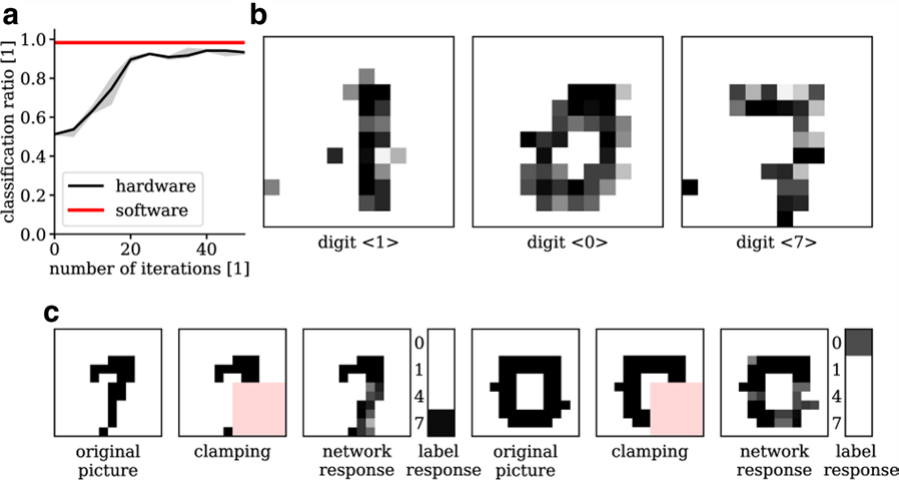
Classification, inference and dreaming of handwritten digits: A Following the transfer of the model from software to hardware, the iterative training recovered a classification rate close to the reference performance level of the ideal software implementation. B Examples of digits generated by the spiking network on the hardware. C The network was able to complete and correctly classify partially occluded digits

**References**

1. S. Furber, *Journal of Neural Engineering* 2016, 13, 051001.
2. P. Berkes, G. Orban, M. Lengyel, and J. Fiser, *Science* 2011, 331, 83.
3. G. Orban, P. Berkes, J. Fiser, and M. Lengyel, *Neuron* 2016, 92, 530.
4. M. A. Petrovici, J. Bill, I. Bytschok, J. Schemmel, and K. Meier, *Physical Review* 2016, 94, 042312.
5. A. Kutschireiter, S. C. Surace, H. Sprekeler, and J.-P. Pfister, *Scientific reports* 2017, 7, 8722.
6. J. Jordan, M. A. Petrovici, O. Breitwieser, J. Schemmel, K. Meier, M. Diesmann, and T. Tetzlaff, *arXiv* preprint arXiv 2017, 1710.04931.
7. J. Schemmel, D. Bruderle, A. Grubl, M. Hock, K. Meier, and S. Millner, in Circuits and systems (ISCAS), *proceedings IEEE*, 1947–1950.
8. Y. LeCun, L. Bottou, Y. Bengio, and P. Haffner, *Proceedings of the IEEE* 1998, 86, 2278.

## P155 Modeling rhythmic control of brain sequential dynamics

### Roberto Latorre^1^, Pablo Varona^1^, Mikhail I. Rabinovich^2^

#### ^1^Universidad Autónoma Madrid, Ingeniería Informática, Madrid, Spain; ^2^University of California, San Diego, BioCircuits Institute, La Jolla, CA, United States

##### **Correspondence**: Pablo Varona (pablo.varona@uam.es)

*BMC Neuroscience* 2018, **19(Suppl 2):**P155

Brain hierarchical networks cooperate and compete in wide variety of spatial and temporal scales producing robust sequential dynamics. Many anatomical and functional motifs emerge dynamically among different brain elements depending on the interconnections in the network and the intrinsic oscillations of the nodes, which can correspond to different levels of the neural hierarchy, from single neurons to large neural ensembles with coherent activity. From this perspective, we build here a winnerless-competition heteroclinic network of oscillatory nodes to study how an endogenous or external periodic frequency can control the switching among different subprocesses relying on the coherence and coordination of the network sequential activity. We relate this dynamical switching to attention processes and cognitive/behavioral brain functions. The network dynamics is modeled with a basic rate-phase motif model based on an adaptation of the generalized Lotka-Volterra model (GLV) [1, 2]. Such dissipative models are convenient approaches able to describe two key aspects of cognitive dynamics: sequential transient behavior and the intrinsic oscillatory nature of its constituent elements. Taking into account the observed metastable informational patterns in experimental recordings, the proposed basic dynamical model helps to understand different informational phenomena that can be related to behavioral and cognitive activity in the framework of the same structural heteroclinic network. In particular, we show that rhythmic signals in a heteroclinic motif network can effectively produce a wide variety of coordinated sequential activations with key computational properties such as spectrum control, dynamical filtering, information modality binding and encoding enhancement. In the view of our results, we propose a complementary view on brain rhythms from the perspective of their interaction with neural sequential dynamics involved in a wide variety of information processing tasks. Results of our simulations show that multifunctional motif networks with sequential activations entrained by external rhythms, described by a simple frequency and amplitude, can evolve through distinct dynamical states. These states can be related to different brain functions and characterized by the broadness of their frequency spectrum, their level of regularity and the specific features of the sequential activations generated in a heteroclinic network. These results also suggest the analysis of sequential activity in novel protocols that use rhythmic input using, for instance, transcranial stimulation or evoked potentials to relate neural activity to cognitive functions, and their associated pathologies [3–5]. As far as we know, this is the first time such an approach is proposed and can provide insight for the design of novel experimental paradigms with rhythmic transcranial or sensory stimulation.

**Acknowledgement**

This work was funded by MINECO/FEDER DPI2015-65833-P, NSF CCF-1640227, ONR N00014310205 and N00014-13-1-0678.

**References**

1. Rabinovich MI, Simmons AN, Varona P. Dynamical bridge between brain and mind. *Trends Cogn Sci*. 2015;19:453–461.
2. Rabinovich M, Tristan I, Varona P. Neural Dynamics of Attentional Cross-Modality Control. *PLoS One* 2013;8:e64406.
3. Fernandez-Vargas J, Pfaff HU, Rodriguez FB, Varona P. Assisted closed-loop optimization of SSVEP-BCI efficiency. *Front Neural Circuits*. 2013;7:Article 27.
4. Rabinovich MI, Varona P. Consciousness as Sequential Dynamics, Robustness, and Mental Disorders. *JAMA Psychiatry*. 2017;74:771–2.
5. Romei V et al. Causal evidence that intrinsic beta-frequency is relevant for enhanced signal propagation in the motor system as shown through rhythmic TMS. *Neuroimage* 2016; 126:120–30.

## P156 An excitation/inhibition ratio impacts on organization of neural connectivity and information transfer

### Motohiro Ogura, Jihoon Park, Yuji Kawai, Minoru Asada

#### Osaka University, Suita, Osaka, Japan

##### **Correspondence**: Motohiro Ogura (motohiro.ogura@ams.eng.osaka-u.ac.jp)

*BMC Neuroscience* 2018, **19(Suppl 2):**P156

Unusual behaviors in autism spectrum disorder have been supposed due to the elevation of an excitation/inhibition (E/I) ratio of neurons [1]. It has been hypothesized that this E/I imbalance causes atypical neural connectivity in autistic brains [2]. However, how the E/I balance affects neural connectivity remains unclear. The purpose of this study is to understand relationship among the E/I ratio, the connectivity, and information transfer based on the Izhikevich’s spiking neural network model [3]. Our model consists of two neuron groups, each of which has 1000 excitatory or inhibitory neurons. An excitatory neuron has 100 connections to others in the same group and three connections to neurons in the other group. An inhibitory neuron is connected to 100 excitatory neurons in the same group. Inter and intra connections are updated according to the spike-timing-dependent plasticity rule [4]. We conducted experiments with different E/I ratios, and evaluated inter connectivity and transfer entropy. Fig. 1 shows the averaged weights of interconnections and the transfer entropy. Here, we defined group1 as a neuron group with a higher value of weights to the other group than the other, for each trial. We tested the model with each E/I ratio 10 times. Our results show that when the E/I ratio was close to 8.2:1.8, asymmetric connections were organized between groups (Fig. 1A, B). We also found that the transfer entropy from group 1–2 was higher than the opposite transfer entropy (Fig. 1C and D) which was among zero (Fig. 1 D). Figure 1. Panel A and B show connection weight from group 1–2 and from group 2–1, respectively. Panel C and D show the transfer entropy from group 1–2 and from group 2–1, respectively. Our simulation demonstrated that the typical E/I ratio (around 8:2) caused asymmetric intergroup connections and the higher transfer entropy. We suppose that this bias for the asymmetric connectivity and directionality of information transfer might be a basis of functional organization of a brain. This may imply atypical connectivity in autistic brains might originate from the E/I imbalance, which leads to atypical information processing.

**Fig. 1:**
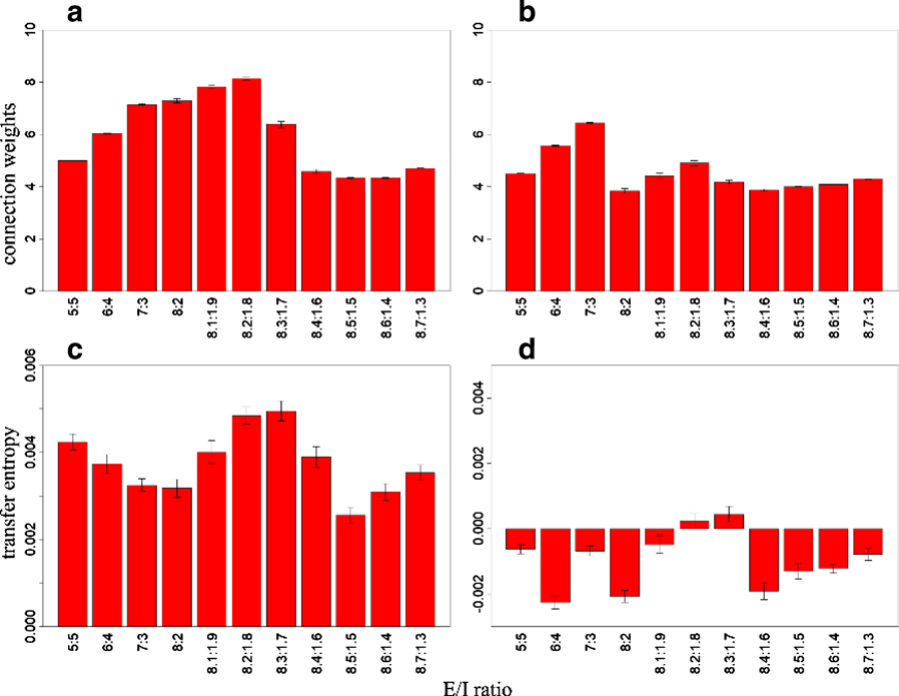
Averaged weights of interconnections and the transfer entropy

**Acknowledgements**

This work was supported by JST CREST Grant Number JPMJCR17A4, Japan.

**References**

1. Yizhar O, et al. Neocortical excitation/inhibition balance in information processing and social dysfunction. *Nature* 2011, 477, 7363, 171.
2. Supekar K, et al. Brain Hyperconnectivity in Children with Autism and its Links to Social Deficits. *Cell Reports* 2013, 5, 738–747
3. Izhikevich, EM. Simple model of spiking neurons. *IEEE Transactions on neural networks* 2033, 14(6), 1569–1572.
4. Izhikevich, EM. Polychronization: computation with spikes. *Neural computation* 2006, 18(2), 245–282.

## P157 Intrinsically bursting neurons enlarge timescales of fluctuations in firing rates

### Tomohiro Miki, Yuji Kawai, Jihoon Park, Minoru Asada

#### Osaka University, Suita, Osaka, Japan

##### **Correspondence**: Tomohiro Miki (tomohiro.miki@ams.eng.osaka-u.ac.jp)

*BMC Neuroscience* 2018, **19(Suppl 2):**P157

Timescales of fluctuations in single-neuron spiking activity are reported to vary depending on cortical areas in a resting-state macaque brain, implying that the timescales contribute to areal functional specialization [1]. However, it remains unclear what intrinsic neural properties cause the various timescales. The purpose of this study is to identify the key parameter of a spiking neural network model to determine the timescale. We focus on the intrinsically bursting (IB) neurons that have a shorter refractory period than the regular spiking (RS) neurons. The IB neurons, if injected dc current, fire burst of spikes, in contrast, the RS neurons fire a few spikes with short interspike period, i.e. refractory period. We evaluated the timescales in neural networks consisting of one thousand Izhikevich’s spiking neurons [2], where the ratios of the number of the IB neurons to that of the RS ones were varied. The timescale*T*was evaluated using the decay of the spike-count autocorrelation*R*for pairs of time bins (currently, 150 ms) based on the equation (1) in Fig. 1, which shows the timescales averaged over all neurons in networks with various ratios of the number of the IB neurons to that of the RS ones. When the timescale becomes large, neurons tend to maintain the number of spikes per bin. Only within the range of the ratio, from 0.95 to 1.35, the exponential decays of the autocorrelation, which were observed in macaque brain [1], were observed. The median of the timescale for 10 trials became large as increase in the number of the IB neurons. This trend indicates that the IB neurons contributes to the maintenance of their firing rates rather than the RS neurons. We speculate that difference of refractory period between RS and IB neuron lead this result. As we mentioned above, refractory period of RS neuron is larger than IB neuron (i.e. the time to next spike is long), therefore, fluctuation of firing rate of RS neuron within each bin is larger than IB neuron.

**Fig. 1:**
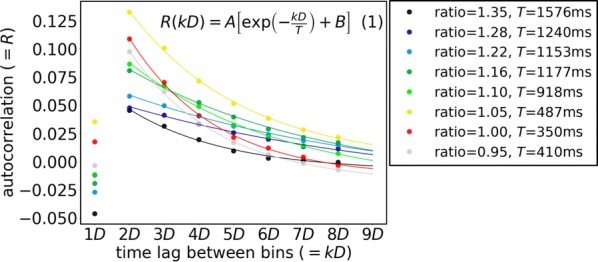
Spike-count autocorrelation was computed with time bins (D = 150 ms) for each ratio of the number of the IB neurons to that of the RS ones. Each color corresponds to the ratio. The timescale T is defined as an exponential decay of the autocorrelation R with a coefficient A and an offset B. Solid lines show the exponential fit as a function of time lag kD between bins. The value of the timescale is the median for 10 trials

**Future issues** The current model had random connectivity, although the existing computational study figured out that the biologically realistic connectivity, e.g., layer-specific connectivity, also contributes to reproduce the various timescales [3]. We plan to investigate how these factors, i.e., the intrinsic properties of an individual neuron and connectivity between neurons have impact on the timescales. Furthermore, how the various timescales yield functional differentiation is an interesting question.

**Acknowledgement**

This work was supported by JST CREST Grant Number JPMJCR17A4, Japan.

**References**

1. Murray AM, et al. A hierarchy of intrinsic timescales across primate cortex. *Nature Neuroscience* 2014,.17, 1661–1663.
2. Izhikevich EM. Simple Model of Spiking Neurons. *IEEE Trans*. *Neural Netw*. *Learn*. *Syst*.2003, 14(6), 1569–1572.
3. Schmidt M, et al. Full-density multi-scale account of structure and dynamics of macaque visual cortex. *arXiv preprint arXiv* 2016, 1511.09364v4.

## P158 Acetylcholine modulation in a biophysical model of cortical neuron

### Vinícius Cordeiro^1^, Parviz Ghaderi^2^, Sareh Rostami^2^, Rodrigo F. O. Pena^1^, Renan O. Shimoura^1^, Antônio C. Roque^1^, Mir Shahram Safari^2^

#### ^1^University of São Paulo, Department of Physics, Ribeirão Preto, Brazil; ^2^Shahid Beheshti University of Medical Science, Neuroscience Research Center, Tehran, Iran, Islamic Republic of Iran

##### **Correspondence**: Vinícius Cordeiro (vinicius.lima.cordeiro@usp.br)

*BMC Neuroscience* 2018, **19(Suppl 2):**P158

Cholinergic inputs from the basal forebrain modulate various visual cortex functions, including visual discrimination, contrast response function, orientation tuning, signal to noise ratio (SNR), and plasticity. Acetylcholine (ACh) has been known to exert its modulatory action via two distinct acetylcholine receptors (AChR), namely nicotinic ACh receptors (nAChRs) and muscarinic ACh receptors (mAChRs) [1]. To understand the mechanisms underlying cholinergic neuromodulation in the primary visual cortex (V1), we developed a biophysical model of V1 neurons incorporating both nicotinic and muscarinic neuromodulatory effects. The model was implemented in NEURON and NetPyNe using Python [2]. The cholinergic mAChRs modulation was modeled as the inactivation ofImandIKCachannels, and nAChRs as an extra ohmic current [3]. The external stimulus was given byIext(t) = log10(C + 1)cos(θ − θ0) [4], which enables simulation of grating bars with contrast levelCand orientationθ, whereθ0is the neuron’s preferred orientation. An extra synaptic background input was injected into the neuron to mimicin-vivolike activity. To study how ACh modulates orientation tuning, first we estimated the orientation selectivity index (OSI) [4] both with and without ACh. The response curves and the measured OSI were compared to our own experimental data. To determine the influence of ACh on the SNR, we computed the coherence function between the membrane voltage time series and the external synaptic input. Finally, to study the influence of ACh modulation in synaptic communication and information flow we computed the Granger causality between coupled neurons. Our results show that synaptic background is fundamental to increase the orientation selectivity in neurons with mAChRs and also nAChRs increases the information capacity of the cell. The comparisons between the response curves generated by the neuron model and experimental response curves showed good agreement.

**Acknowledgements**

This work was produced as part of the activities of FAPESP Research, Disseminations and Innovation Center for Neuromathematics (Grant 2013/07699-0, S. Paulo Research Foundation). VLC, RFOP and ROS are recipients of the respective FAPESP scholarships: 2017/05874-0, 2013/25667-8, and 2017/07688-9. ACR is partially supported by the CNPq fellowship Grant 306251/2014-0. RFOP and ACR are also part of the IRTG 1740/TRP 2015/50122-0, funded by DFG/FAPESP.Part of this work was done during the VII Latin American School on Computational Neuroscience (LASCON).

**References**

1. Lucas-Meunier E, Fossier P, Baux G, Amar M. Cholinergic modulation of the cortical neuronal network.. *Pflügers Arch* 2003, 446, 17–29.
2. Hines M, Davison AP, Muller E:NEURON and Python. *Front Neuroinform* 2009, 3:1.
3. Li G, Cleland TA. A two-layer biophysical model of cholinergic neuromodulation in olfactory bulb. *J Neurosci* 2013, 33, 3037–3058.
4. Gonzalo Cogno S, Mato G. The effect of synaptic plasticity on orientation selectivity in a balanced model of primary visual cortex. *Front Neural Circuits* 2015, 9, 42.

## P159 Anesthesia modifies subthreshold critical slowing in a stochastic Hodgkin-Huxley neuron exposed to inhibitory synaptic noise

### Alex Bukoski^1^, D Alistair Steyn-Ross^2^, Ashley Pickett^3^, Moira L Steyn-Ross^2^

#### ^1^University of Missouri, Columbia, MO, United States; ^2^University of Waikato, School of Engineering, Hamilton, New Zealand; ^3^Auburn University, College of Veterinary Medicine, Auburn, AL, United States

##### **Correspondence**: Alex Bukoski (bukoskia@missouri.edu)

*BMC Neuroscience* 2018, **19(Suppl 2):**P159

Critical slowing-down has been demonstrated in both computational models [1, 2] and experimental preparations [3] as neurons transition from subthreshold quiescence to supra-threshold spike formation. In single neurons, this nonlinear increase in neuronal sensitivity—characterized by growth in amplitude simultaneous with decay in frequency of soma voltage perturbations—is known to obey power-law scaling in the region of the bifurcation that defines spike formation. We investigate the behavior of a stochastic type-I Hodgkin-Huxley point neuron exposed to inhibitory synaptic activity mediated byγ-aminobutyric acid (GABA) type-A receptors as it is driven toward threshold from below by an excitatory input current. The model includes the effects of both intrinsic ion-channel noise as well as inhibitory synaptic noise modeled as multiple Poisson-distributed impulse trains with saturating response functions. The influence of anesthesia is included by reducing the inhibitory synaptic decay rate and is intended to model the primary effect of the GABAergic agent propofol. We find that for a given distance from spiking threshold, increasing anesthetic effect is associated with augmented signatures of critical slowing: fluctuation amplitudes and correlation times grow as spectral power is increasingly focused at 0 Hz. Additionally, anesthesia significantly modifies the inverse square root power-law scaling for variance and correlation time divergences anticipated for deterministic saddle-node bifurcations in such a way that their observability is reduced as anesthetic effect is increased. Compared to the case of no synaptic input, application of linear multivariate Ornstein–Uhlenbeck analysis to the model equations reveals this effect to be the consequence of an additional slow eigenvalue that effectively swamps those of the underlying point neuron unless the distance to spiking threshold is sufficiently small. If subthreshold voltage fluctuations are communicated from neuron to neuron by electrical gap junctions, subthreshold dynamics may play an important role in determining neuronal behavior and thus overall cortical dynamics. Anesthesia is predicted to significantly alter these subthreshold dynamics and this may contribute to the overall effect of anesthesia on the cortex.

**References**

1. Steyn-Ross DA, Steyn-Ross ML, Wilson MT, Sleigh JW. White-noise susceptibility and critical slowing in neurons near spiking threshold. *Phys Rev E* 2006, 74(5), 051920.
2. Bukoski A, Steyn-Ross DA, Steyn-Ross ML. Channel-noise-induced critical slowing in the subthreshold Hodgkin-Huxley neuron. *Phys Rev* E 2015, 91(3), 032708.
3. Meisel C, Klaus A, Kuehn C, Plenz D. Critical slowing down governs the transition to neuron spiking. *PLoS Comput Biol* 2015, 11(2), e1004097.

## P160 Identifying “influential seizers” in a network model of focal epilepsy

### Christian Fink, Joe Emerson, Momi Afelin

#### Wesleyan University, Physics and Neuroscience, Delaware, OH, United States

##### **Correspondence**: Christian Fink (cgfink@owu.edu)

*BMC Neuroscience* 2018, **19(Suppl 2):**P160

We present a network measure for identifying brain regions that most effectively spread epileptic seizures (i.e., “influential seizers”). Using the “Epileptor” model recently proposed by Jirsa et. al. (“On the Nature of Seizure Dynamics,” BRAIN, 2014), we simulate seizure spread on the macaque connectome. We first show that the model’s results accord well with clinical data, with regions in the temporal lobe most likely to initiate severe seizures, followed by regions in the frontal lobe. We then present a centrality measure that uses network structure alone to accurately identify influential seizers, without running dynamical simulations. These results suggest that baseline brain connectivity predisposes particular regions to be more seizure-prone than others, even without pathological network reorganization. Our results also hint at improved, less invasive neurosurgical procedures to treat patients with focal epilepsy.

## P161 Rich dynamical repertoire in the balanced state

### David Dahmen, Lukas Deutz, Moritz Helias

#### Jülich Research Centre, Institute of Neuroscience and Medicine (INM-6), Juelich, Germany

##### **Correspondence**: David Dahmen (d.dahmen@fz-juelich.de)

*BMC Neuroscience* 2018, **19(Suppl 2):**P161

Experimental evidence for cortical networks operating in the balanced state is overwhelming [1, 2, 3]. In this state, strong recurrent inhibition yields almost vanishing correlations in the input to neurons [4, 5]. The balanced state, however, only restricts average correlations in the network due to the large convergence of connections. Here we show that balanced networks can show a rich correlation structure between individual neurons that is explained by the effective connectivity of the network [6]. The latter is determined by the anatomical connections and the sensitivity of neurons to inputs. Large heterogeneity in effective connections causes nearly unstable linearized dynamics in various directions of the high-dimensional space of all neurons, leading to multiple-neuron responses with largely different time courses [7]. As a consequence distributions of correlations across neurons become broad, but approximately centered around zero. A large dispersion of correlations, as for example obtained from recordings in macaque motor cortex, can therefore be used as an indicator of a rich dynamical repertoire, which is hidden from macroscopic brain signals, but essential for high performance in such concepts as reservoir computing [8, 9].

**Acknowledgement**

Supported by HGF young investigator’s group VH-NG-1028, Helmholtz portfolio theme SMHB, and EU Grants 604102 and 720270 (Human Brain Project, HBP).

**References**

1. Okun M, Lampl I. Instantaneous correlation of excitation and inhibition during ongoing and sensory-evoked activities. *Nat*. *Neurosci*. 2008, 11:535.
2. Ecker AS, Berens P, Keliris GA, Bethge M, Logothetis NK: Decorrelated Neuronal Firing in Cortical Microcircuits. *Science* 2010, 327:584–587.
3. Dehghani N, Peyrache A, Telenczuk B, Le Van Quyen M, Halgren E, Cash SE, Hatsopoulos NG, Destexhe A: Dynamic Balance of Excitation and Inhibition in Human and Monkey Neocortex. *Scientific Reports* 2016, 6:23176.
4. Renart A, De La Rocha J, Bartho P, Hollender L, Parga N, Reyes A, Harris KD: The asynchronous State in Cortical Circuits. *Science* 2010, 327:587–590.
5. Tetzlaff T, Helias M, Einevoll G, Diesmann M: Decorrelation of neural-network activity by inhibitory feedback. *PLOS Comput*. *Biol*. 2010, 8(8):e1002596.
6. Dahmen D, Diesmann M, Helias M: Distributions of covariances as a window into the operational regime of neuronal networks. *arXiv* 2016, 1605.04153 [cond-mat.dis-nn].
7. Dahmen D, Grün S, Diesmann M, Helias M: Two types of criticality in the brain. *arXiv* 2017, 1711.10930 [cond-mat.dis-nn].
8. Maass W, Natschläger T, Markram H: Real-time computing without stable states: a new framework for neural computation based on perturbation. *Neural Comput*. 2002. 14(11) 2531–2560.
9. Jaeger H, Haas H: Harnessing nonlinearity: predicting chaotic systems and saving energy in wireless communication. *Science* 2004, 304(5667) 78–80.

## P163 Prefrontal oscillations bias pathways for thought and action

### Jason Sherfey^1^, Joachim Hass^2^, Salva Ardid^3^, Michael Hasselmo^1^, Nancy Kopell^3^

#### ^1^Boston University, Psychological and Brain Sciences, Boston, MA, United States; ^2^Central Institute of Mental Health, BCCN Heidelberg-Mannheim, Mannheim, Germany; ^3^Boston University, Mathematics and Statistics, Boston, MA, United States

##### **Correspondence**: Jason Sherfey (sherfey@bu.edu)

*BMC Neuroscience* 2018, **19(Suppl 2):**P163

The prefrontal cortex (PFC) flexibly encodes task-relevant representations and outputs biases to mediate higher cognitive functions like working memory and rule-based action. The relevant neural ensembles undergo task-related changes in oscillatory dynamics at beta- and gamma frequencies. However, the impact of those changes on target networks and their functional significance for cognition are poorly understood. In this work, we used computational modeling to explore these issues. First, we characterized the network response of a biophysically detailed model of the deep PFC output layer driven by a single input. We show that strong feedback inhibition causes the PFC to generate internal oscillations in the beta/gamma range and to prefer external oscillations at slightly higher frequencies. Importantly, we show that the fastest oscillation frequency that can be relayed by the output network maximizes local inhibition and is equal to a frequency even higher than that of the preferred external oscillation; we call this phenomenon population frequency resonance. Functionally, adaptive cognition requires dynamic mechanisms that can flexibly route signals in different ways using the same underlying neural circuitry. Changes in oscillatory synchronization across rate-coding populations of neurons in PFC have been implicated in a variety of cognitive tasks that require flexible routing. Using a version of our model that includes multiple inputs from rate-coding populations in different dynamical regimes (asynchronous activity and periodic activity with variable synchrony and frequency), we show that the dynamical states of input populations can exhibit a stronger influence over downstream competition than their activity levels (firing rates). Specifically, when multiple inputs from parallel or convergent pathways drive target populations connected to shared interneurons, these dynamics bias competition in favor of the most frequency-resonant input. Essentially, the output population with the shortest period between activations tends to be the dominant driver of local inhibition that suppresses all populations connected to the same interneurons. This form of biased competition, mediated by oscillations, increases with input synchrony and enables an output population driven by a weaker frequency-resonant input to suppress lower-frequency competing responses to stronger inputs. Furthermore, the frequency-resonant bias can be amplified to produce winner-take-all selection by plasticity of recurrent connections that strengthen output responses (e.g., across repeated trials of task). Our model predicts that the experimentally-observed PFC beta and gamma oscillations could leverage frequency-resonance to bias responses in the output layer, and that task-related modulation of oscillatory synchronization could govern the flexible routing of signals in service of cognitive processes like output gating from a working memory buffer and the selection of rule-based actions.

## P164 From single neurons to perception: Examining the basis for sensory deficits in autism

### Rashid Williams-Garcia^1^, G. Bard Ermentrout^2^, Nathan Urban^3^

#### ^1^University of Pittsburgh, Department of Neurobiology & Department of Mathematics, Pittsburgh, PA, United States; ^2^University of Pittsburgh, Department of Mathematics, Pittsburgh, PA, United States; ^3^University of Pittsburgh, Department of Neurobiology, Pittsburgh, PA, United States

##### **Correspondence**: Rashid Williams-Garcia (rwgarcia@pitt.edu)

*BMC Neuroscience* 2018, **19(Suppl 2):**P164

Sensory deficits, such as hyper- and hyposensitivity, as well as sensation avoidance and seeking behaviors, are frequently associated with autism spectrum disorders (ASDs). Quantitative differences in the properties and responses of individual sensory neurons—and their networks—compared to their neurotypical counterparts, potentially drive these deficits. Empirical studies suggest that neural networks from autistic individuals and animal models feature altered neuronal excitability, connectivity, and stimulus response variability. The precise link between these alterations and the behavioral symptoms, though as yet unknown, is key to understanding ASDs. I will present some recent progress in examining this link using a computational approach. Individual neurons and their local interactions are simulated to examine the relationship between spike train correlations and variability, neuronal excitability, synaptic strength, and spike frequency adaptation (SFA). Our findings indicate that impaired SFA and weakened synaptic strengths increase spike train variability and neuronal excitability, a combination which might have consequences for sensory processing.

## P165 Cortical information integration with critical subnetworks: Large capacity, high accuracy, and rapid detection

### Maik Schünemann, Udo Ernst, Nergis Tomen

#### University of Bremen, Institute for Theoretical Physics, Bremen, Germany

##### **Correspondence**: Maik Schünemann (maikschuenemann@gmail.com)

*BMC Neuroscience* 2018, **19(Suppl 2):**P165

Experimental findings and theoretical considerations indicate that cortical networks operate in a critical state between fully ordered and chaotic dynamics which is characterized by collective neuronal bursting (avalanches) occurring on a wide range of spatial and temporal scales. At this state, a number of information-theoretic measures are maximized, suggesting a high significance of critical dynamics for cortical function. However, concrete proposals for how criticality can practically be used in sensory processing are rare and abstract.

In this contribution we present a theory of information integration in heterogeneous cortical networks receiving heterogeneous external inputs. Our central idea is that we do not require the whole network of size **N** to be in a critical state: Instead, we define **N** subnetworks by connecting all **N** units inside each subnetwork with recurrent couplings at a critical coupling strength **alpha** crit(Fig. 1(a), yellow regions). Now a critical dynamics only emerges if an external input co-activates units belonging to one of the subnetworks (Fig. 1(b), inset). If we interpret the external input as evidence about the presence of particular feature combinations in a scene (a “figure”), the critical dynamics can be used efficiently for rapid integration of information that ‘belongs together’, thus supporting cortical functions such as texture segmentation and figure-ground segregation. Starting with the analytically tractable Eurich-Herrmann-Ernst model [1], we extend the derivation of avalanche statistics from a global homogeneous coupling to a general class of heterogeneous, non-negative coupling matrices. In particular we show that this model is equivalent to a group action of translations on the **N**-torus. The avalanche statistics can be derived from the volume of the corresponding regions on the torus using ergodicity. This mathematical connection paves the way for analytical investigations of the effects of different connectivity schemes on collective network events. To show that criticality in subnetworks can be efficiently used for tasks such as feature integration we investigate the capacity to embed **N** randomly drawn subnetworks of size **N** into a large connectivity matrix of*N*units in total. We find that by introducing inhibitory connections between two units that do not share a common subnetwork it is possible to embed a large number of subnetworks before runaway activity emerges (Fig. 1(b)). Although the inhibitory system is not analytically tractable anymore we can still describe the phase space boundary of the transition to runaway activity (Fig. 1(b), black line) analytically. Over the whole phase space, two-alternative forced choice detection accuracy between external stimuli containing a “figure” and containing only unrelated features is high and requires only a small observation time interval by means of a coincidence detection scheme of synchronous events (“avalanches”).

**Fig. 1:**
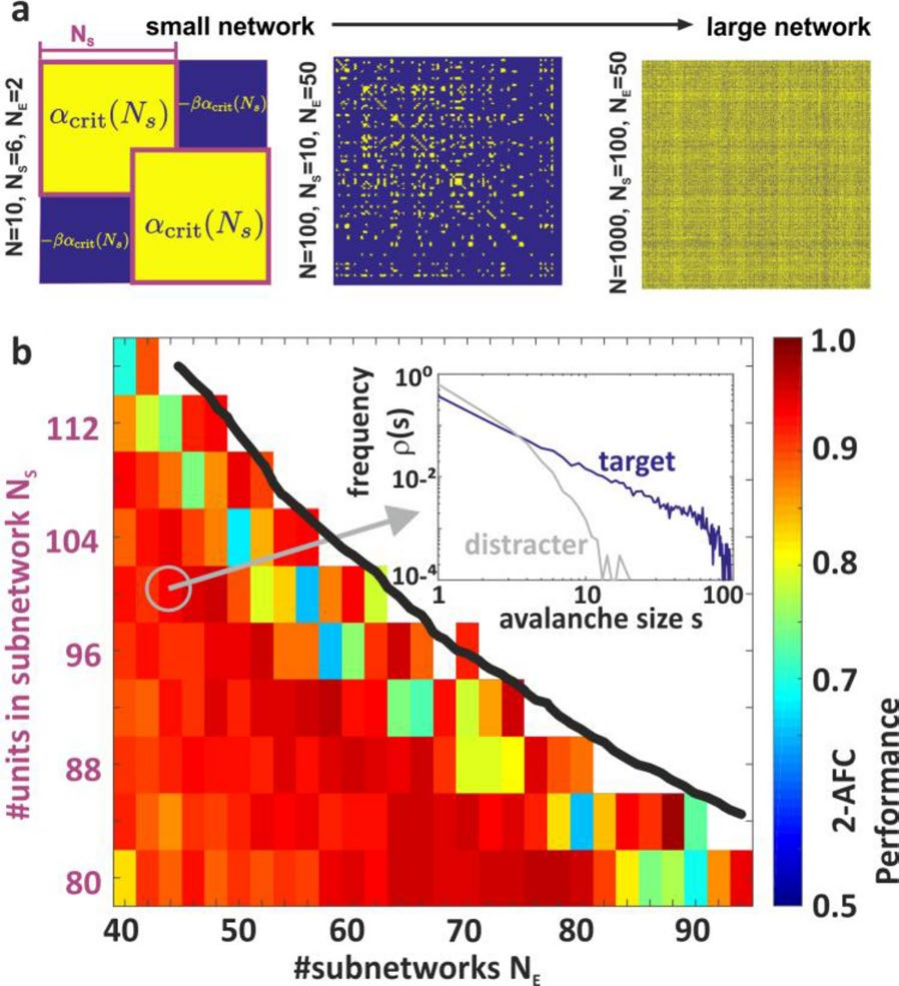
(a) Embedding subnetworks with critical dynamics (yellow) into larger networks. From toy examples to realistic sizes. (b) 2-AFC performance in a “figure-detection” task in dependence on number Ne of embedded subnetworks of size Ns. Inset, target versus distracter avalanche sizes for the parameter combination indicated with the gray circle

**Acknowledgement**

This work was supported by the BMBF (Bernstein Award Udo Ernst, Grant no. 01GQ1106) and by the DFG priority program SPP 1665 (ER 324/3-1).

**Reference**

1. Eurich CW, Herrmann JM, Ernst UA. Finite-size effects of avalanche dynamics. *Physical Review E*. 2002, 66(6):066137.

## P166 Neuroscience gateway: Enabling large scale simulations and data processing and dissemination of neuroscience tools/software

### Amitava Majumdar^1^, Subhashini Sivagnanam^1^, Kenneth Yoshimoto^1^, Nicholas Carnevale^2^

#### ^1^University of California, San Diego, San Diego Supercomputer Center, La Jolla, CA, United States; ^2^Yale University, Neuroscience, New Haven, CT, United States

##### **Correspondence**: Amitava Majumdar (majumdar@sdsc.edu)

*BMC Neuroscience* 2018, **19(Suppl 2):**P166

High performance computing (HPC) resources provided at supercomputer centers worldwide are impacting modeling, simulation, data processing and analytics essential to basic and clinical neurosciences research. The US Brain Initiative, the European Human Brain Project and the recently (Dec 2017) signed International Brain Initiative (Japan, Korea, Europe, US, Australia etc.) all deal with large scale computing and data processing and utilize supercomputing resources. Complex empirically-based models of cells and large scale networks are developed in labs around the world and require HPC for large scale simulations; new experimental methods for brain structure/function generate avalanche of data requiring computationally intensive analysis—all of these for understanding of how the normal brain functions or brain functions related to neurodegenerative diseases. As the scale of both of these increase, HPC is increasingly applied for neuroscience research. The Neuroscience Gateway (NSG http://www.nsgportal.org), funded by the US NSF and NIH, catalyzes computational neuroscience research by lowering or eliminating the administrative and technical barriers that can make it difficult for neuroscience researchers to access and use supercomputer resources for large scale simulations and data processing. It provides free and open access to supercomputers for neuroscientists from anywhere in the world. Supercomputer time is acquired via the peer reviewed allocation process managed by the NSF Extreme Science and Engineering Discovery Environment (XSEDE) project. Since inception in early 2013, it has over 600 registered users (and growing), and provided many tens of millions of core hours on XSEDE HPC resources to neuroscientists, including ~ 10 million supercomputer core hours/year in recent years. NSG architecture (see attached diagram) is designed to be accessed through a simple web portal or programmatically using RESTful services. NSG provides a streamlined environment for uploading models, setting data processing parameters, specifying HPC job parameters, querying running job status, receiving job completion notices, and storing and retrieving output data. The NSG transparently distributes user’s jobs to appropriate XSEDE HPC resources. NSG provides a large number of neuroscience tools, software, pipelines and data processing tools—BluePyOpt (from EU HBP project), Brian, CARLSim4 (GPU based network tool), DynaSim (Matlab tool for dynamical systems), EEGLAB, Freesurfer, Human Neuocortical Neurosolver (modeling tool to interpret EEG/MEG etc. data), MATLAB, MOOSE, NEST, NetePyne, NEURON, Octave, Parameter Search Tool, PGENESIS, PyNN, Python, R, TensorFlow, TVB-Personalized Multimodal Connectome etc. The poster will describe how the NSG architecture (both web based and programmatic REST service) allows implementation of these tools for users and the usage mode of these tools, as well as how NSG interfaces with projects such as the Open Source Brain and its users. In recent years NSG has become a development and dissemination platform for new neuroscience tools and pipelines, as well as a tool for neuroscience education. The poster will describe how NSG is evolving as a result of all of these natural requirements from the neuroscience community (Fig. 1).

**Fig. 1:**
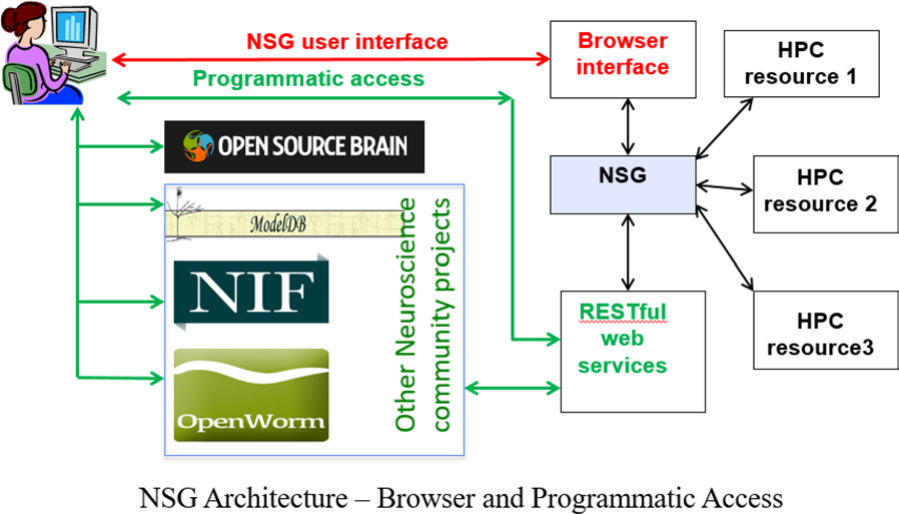
NSG architecture—web portal and programmatic access

## P167 Computational model of the conditional probability of decision-making process as an optimization process

### Nicoladie Tam

#### University of North Texas, Department of Biological Sciences, Denton, TX, United States

##### **Correspondence**: Nicoladie Tam (nicoladie.tam@unt.edu)

*BMC Neuroscience* 2018, **19(Suppl 2):**P167

Computational model of the decision-making process under conflict can be considered as a process of choice making among multiple-choices of conflicting situations. Conflict is defined as choosing only one choice among many other options, when choosing one option could nullify the selection of other options. Such process assumes the decision is a binary choice of “yes” or “no,” based on the Boolean logic of binary logic of “1” or “0.” Yet, the computational underlying the decision-making process may require fuzzy logic of probability rather than Boolean logic of “true” or “false.” Therefore, in order to account for the continuum of probability values which range between 0 and 1 in the underlying computations that comprise of the decision-making process, which can be represented by the continuous range of neural firing rates. Most importantly, in computing the conditional probability that underlies the decision which may not reach the threshold for absolute true or false, the probability of decision may be a better representation of the underlying decision than absolute decision. Towards this goal to compute the conditional probabilities in the decision-making process, the selection of choices can be considered as an optimization process to maximize gains while minimize loses. We propose to use the optimization process for gain-maximization and loss-minimization as the operating principle for making complex decisions. More importantly, in order to account for the social interaction of reciprocity in trust and fairness [1], the decision has to balance between trust-behavior (which is opposite to risk-taking behavior) and fair-behavior (which requires taking into the account of the other interacting person into account). We propose a computational method to take all of the above considerations into account by, not only maximizing the perceived gains while minimizing the perceive losses, but also updating the expectation assumptions in trust-making when the trust is violated in the social interaction. In other words, when expectation of the predicted behavior of the person interacting with does not correspond to the reality in outcome, then an updating of the expectation prediction will be adjusted to correspond to the reality. This adjustment in the prediction of reciprocity behavior would also alter the conditional probability based on the response of the person interacting with. The prediction of the reciprocity behavior is computed by the conditional probability of the product of the risk in trusting the person and the degree of fairness between the two individuals. In order to validate the computational model, we test the hypothesis with decisions made by human subjects playing a social reciprocity game of trust and fairness. The underlying conditional probabilities of risk and fairness can be estimated depending on whether the expectation predictions are updated when the trust is violated. We found that there is a good approximation between the conditional probability and the updating of the expectation prediction.

**Reference**

1. Tam ND: Computational social interaction in reciprocity and empathic behavior as behavioral economics and risk tasking behavior. *BMC Neuroscience* 2017, 18(1), :P103.

## P168 PyRates: A Python framework for rate-based neural simulations

### Richard Gast, Thomas Knoesche, Daniel Rose, Harald Möller, Nikolaus Weiskopf

#### MPI for Human Cognitive and Brain Sciences, Department of Neurophysics, Leipzig, Germany

##### **Correspondence**: Richard Gast (rgast@cbs.mpg.de)

*BMC Neuroscience* 2018, **19(Suppl 2):**P168

In neuroscience, computational modeling has become an important source of insight into brain states and dynamics complementary to classic experiments. This is due to the potential to observe and manipulate variables in the model that are difficult to assess in the living brain. Neural mass models (NMMs) are computationally efficient models for simulating large-scale brain dynamics as observable with neuroimaging techniques such as EEG/MEG or fMRI [1]. They model the dynamic interactions between large, lumped populations of different cell types at the meso- and macroscopic scale. Thereby, a single neural mass reflects the mean-field approximation of the average behavior of a cell population of interest. In this work, we present PyRates, a Python framework that provides the means to build a large variety of different NMMs within a well defined mathematical structure. PyRates is a commitment to open and reproducible computational neuroscience, since each model and functionality within the framework is documented in detail, thoroughly tested and equipped with working examples for the user. To model the neural masses, we provide both an differentio-differential approach for maximal computational efficiency and an integro-differential approach that allows for a flexible implementation of various neurobiological features. These features include plasticity mechanisms, various synaptic and axonal properties as well as different descriptions of the population dynamics. This allows for a highly customizable neural mass design able to approximate a wide range of input–output relationships. PyRates organizes all neural masses of a model in a graph structure optimized for fast information passage and a high degree of parallelization. We show via numerical simulations how PyRates can replicate and extend established NMMs, or build novel NMMs based on the same formal skeleton. To this end, we start out with the thoroughly investigated Jansen-Rit model and replicate key behaviors of the model in PyRates [2]. We then continue by extending the Jansen-Rit model at multiple levels (axonal, synaptic & population properties), thereby demonstrating how biophysical mechanisms originally proposed within specific models can be easily plugged into potentially very different NMMs in PyRates. Finally, we discuss how the framework can be used in combination with various neuroimaging and network analysis tools and conclude that PyRates makes a substantial contribution to open and reproducible neuroscience by providing a unified, fully tested and well documented framework for spatially discrete, rate-based neural simulations (Fig. 1).

**Fig. 1:**
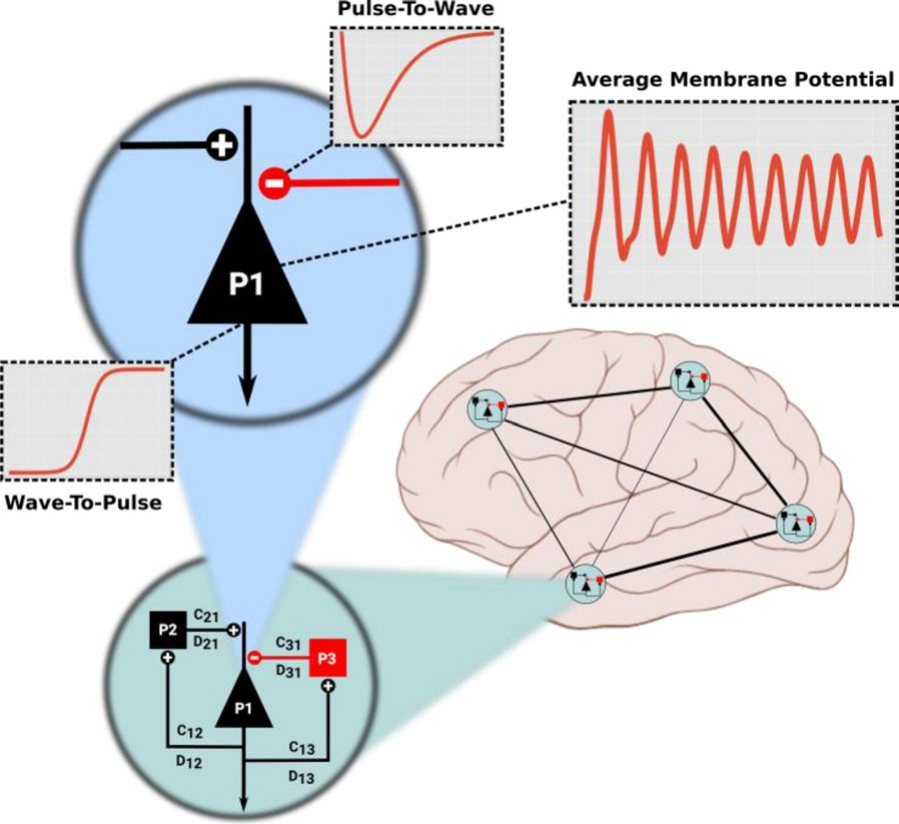
Structure of PyRates. Neural populations (P) are connected within and across micro circuits via connection strengths (C) and delays (D). Connections terminate at synapses that are either excitatory (black) or inhibitory (red). Populations are defined by a) pulse-to-wave transforms that transform incoming, pre-synaptic input into post-synaptic average membrane potential changes and b) wave-to-pulse transforms that transform the average membrane potential back into average firing rates that can in turn be transmitted to other populations

**References**

1. Breakspear M. Dynamic models of large-scale brain activity. *Nature Neuroscience* 2017, 20, 340–352.
2. Jansen BH, Rit VG. Electroencephalogram and visual evoked potential generation in a mathematical model of coupled columns. *Biological Cybernetics* 1995, 73, 357–366.

## P169 A stochastic model of single serotonergic fibers

### Skirmantas Janusonis^1^, Bangalore Manjunath^2^, Nils-Christian Detering^3^

#### ^1^University of California, Santa Barbara, Department of Psychological and Brain Sciences, Santa Barbara, CA, United States; ^2^University of California, Santa Barbara, Department of Electrical and Computer Engineering, Santa Barbara, CA, United States; ^3^University of California, Santa Barbara, Department of Statistics and Applied Probability, Santa Barbara, CA, United States

##### **Correspondence**: Skirmantas Janusonis (janusonis@ucsb.edu)

*BMC Neuroscience* 2018, **19(Suppl 2):**P169

Virtually all neural processes in vertebrate brains are physically embedded in a dense matrix of axons (fibers) that release serotonin (5-HT), a major neurotransmitter. In contrast to point-to-point projections, serotonergic fibers do not have well-defined destinations and instead appear to random-walk through brain regions, producing a meshwork with regionally-specific densities. We have recently proposed that these densities may emerge as a consequence of the stochastic behavior of individual fibers [1]. This process is likely to be fundamental to the brain architecture, but it is also important for the understanding of disorders and conditions associated with altered serotonergic signaling. Changes in serotonergic fiber densities have been reported in Autism Spectrum Disorder [2] and Major Depressive Disorder [3], and they also have been observed after prolonged exposure to MDMA (“Ecstasy”) [4]. Serotonergic fibers in the mouse somatosensory cortex were visualized with fluorescence immunohistochemistry and imaged (in 3D) with confocal laser scanning microscopy (as z-stacks of optical sections, at a resolution of around 150 nm). Individual fibers were manually traced with Fiji ImageJ or automatically segmented in BisQue (http://bioimage.ucsb.edu/bisque). All analyzed fibers were assumed to be realizations of the same stochastic process and were modeled using the von Mises-Fisher (vMF) probability distribution. Long trajectories (more than 500 points) were selected and smoothed to avoid laser-scanning artifacts (with a moving average of 10 points). For each “current” direction of a fiber, the “next” direction was assumed to have the vMF distribution with the parameters μ (a unit-vector representing the “current” direction) and the concentration parameter κ. The fiber κ was calculated using an efficient maximum likelihood estimator [5]. A robust κ estimate (with the mean of around 250) was obtained for the selected brain region and sampling frequency (Fig. 1A). Simulated fibers with the same stochastic process closely resembled the small- and large-scale structure of serotonergic fibers (Fig. 1B). We are currently extending this novel approach to other brain areas and experimentally investigating whether the vMF concentration parameter can predict local serotonergic fiber densities.

**Fig. 1:**
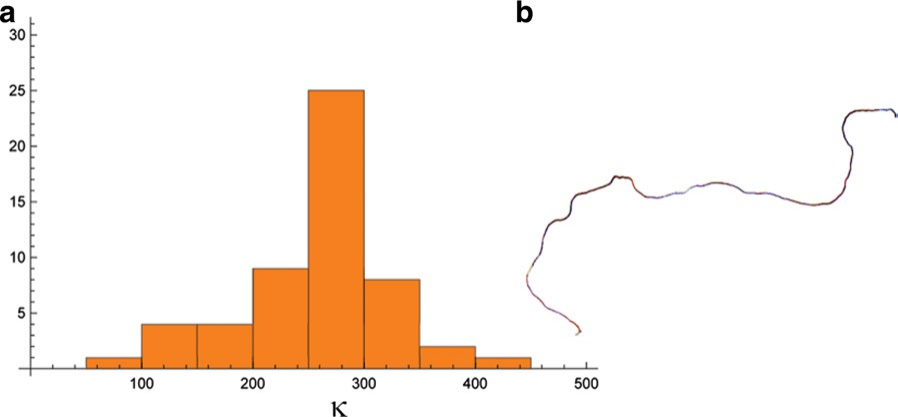
A: The distribution of the estimated κ values of 54 serotonergic fibers in layer IV of the mouse primary somatosensory cortex. B: A simulated fiber with κ = 250 (serotonergic varicosities were not modeled)

**Acknowledgement**

This work was supported by a Challenge Grant of the California NanoSystems Institute. We thank Melissa Hingorani, Kasie Mays, and Riley Demos for assistance with fiber tracing.

**References**

1. Janusonis S. Serotonin in space: Understanding single fibers.*ACS Chem*. *Neurosci*.2017, 8, 893–896.
2. Azmitia EC, Singh JS, Whitaker-Azmitia PM. Increased serotonin axons (immunoreactive to 5-HT transporter) in postmortem brains from young autism donors.*Neuropharmacology* 2011, 60, 1347–1354.
3. Rajkowska G, Mahajan G, Legutko B, et al. Length of axons expressing the serotonin transporter in orbitofrontal cortex is lower with age in depression. *Neuroscience* 2017, 359, 30–39.
4. Vegting Y, Reneman L, Booij J. The effects of Ecstasy on neurotransmitter systems: a review on the findings of molecular imaging studies. *Psychopharmacology* (Berl.) 2016, 233, 3473–3501.
5. Tanabe A, Fukumizu K, Oba S, et al. Parameter estimation for von Mises-Fisher distributions. *Comp*. *Stat*.2007, 22, 145–157.

## P170 Neural model for the recognition of agency and social interaction from abstract stimuli

### Mohammad Hovaidi Ardestani^1^, Martin Giese^2^, Nitin Saini^2^

#### ^1^University Clinic Tübingen, Tübingen, Germany; ^2^Center for Integrative Neuroscience & University Clinic Tübingen, Dept of Cogniitive Neurology, Germany

##### **Correspondence**: Martin Giese (martin.giese@uni-tuebingen.de)

*BMC Neuroscience* 2018, **19(Suppl 2):**P170

**Introduction:** Humans derive spontaneously judgements about agency and social interactions from strongly impoverished stimuli, as impressively demonstrated by the seminal work by Heider and Simmel (1944). The neural circuits that derive such judgements from image sequences are entirely unknown. It has been hypothesized that this visual function is based on high-level cognitive processes, such as probabilistic reasoning. Taking an alternative approach, we show that such functions can be accomplished by relatively elementary neural networks that can be implemented by simple physiologically plausible neural mechanisms, exploiting an appropriately structure hierarchical (deep) neural model of the visual pathway.

**Methods:** Extending classical biologically-inspired models for object and action perception (Riesenhuber & Poggio 1999; Giese & Poggio 2003) by a front-end that exploits deep learning for the construction of low and mid-level feature detectors, we built a hierarchical neural model that reproduces elementary psychophysical results on animacy and social perception from abstract stimuli. The lower hierarchy levels of the model consist of position-variant neural feature detectors that extract orientation and intermediately complex shape features. The next-higher level is formed by shape-selective neurons that are not completely position-invariant, which extract the 2D positions and orientation of moving agents. A second pathway extracts the 2D motion of the moving agents. Exploiting a gain-field network, we compute the relative positions of the moving agents. The top layers of the model combine the mentioned features into more complex high-level features that represent the speed, smoothness of motion and spatial relationships of the moving agents. The highest level of the model consists of neurons that have learned to classify the agency of the motions, and different categories of social interactions.

**Results:** Based on input video sequences, the model successfully reproduces results of Tremoulet and Feldman (2000) on the dependence of perceived animacy on motion parameters, and its dependence on the alignment of motion and body axis (Hernik et al. 2013). In addition, the model correctly classifies four categories of social interactions that have been frequently tested in the psychophysical literature (following, chasing, fighting, guarding) (e.g. Scholl and McCarthy, 2012; McAleer et al., 2011a).

**Conclusion:** Using simple physiologically plausible neural circuits, the model accounts simultaneously for a variety of effects related to animacy and social interaction perception. This leads to interesting predictions about neurons involved in the visual processing of such stimuli.

**Acknowledgement**

This work was supported by: HFSP RGP0036/2016; the European Commission HBP FP7-ICT2013-FET-F/604102 and COGIMON H2020-644727, and the DFG GZ: GI 305/4-1 and KA 1258/15-1.

**References**

1. Heider F, Simmel A. An experimental study of apparent behavior. *The American Journal of Psychology*, 57, 243–259.
2. Riesenhuber M, Poggio T. Hierarchical models of object recognition in cortex. *Nat*. *Neuro* 1999, 2(11), 1019–1025.
3. Giese MA, Poggio T. Neural mechanisms for the recognition of biological movements. *Nat*. *Neuro* 2003, 4(3), 179–192.
4. Tremoulet PD, Feldman J. Perception of animacy from the motion of a single object. *Perception* 2000, 29(8), 943–951.
5. Hernik M, Csibra G. Infants learn enduring functions of novel tools from action demonstrations. *Journal Exp*. *Child Psychol*. 2015, 130, 176–192.
6. Scholl BJ, Gao T. Perceiving Animacy and Intentionality: Visual Processing or Higher-Level Judgment? *Social Perception* 2013, 198–229
7. McAleer M, Mederios MC. Forecasting realized volatility with linear and nonlinear univariate models. *Journal of Economic Surveys* 2010

## P171 Learning oscillatory brain dynamics: van der Pol meets LSTM

### Germán Abrevaya^1^, Aleksandr Aravkin^2^, Guillermo Cecchi^3^, Irina Rish^3^, Silvina Dawson^4^, Pablo Polosecki^3^

#### ^1^Universidad de Buenos Aires & CONICET, Departamento de Física, FCEyN and IFIBA, Buenos Aires, Argentina; ^2^University of Washington, Department of Applied Mathematics, Seattle, WA, United States; ^3^IBM TJ Watson Research Center, Yorktown Heights, United States; ^4^University of Buenos Aires, Departamento de Física, FCEyN, UBA and IFIBA, Buenos Aires, Argentina

##### **Correspondence**: Aleksandr Aravkin (saravkin@uw.edu)

*BMC Neuroscience* 2018, **19(Suppl 2):**P171

Many real-world data sets, especially in biology, are produced by highly multivariate and nonlinear complex dynamical systems. For example, a motivating application for this work is brain imaging, and, more specifically, modeling the whole-brain neural activity of the larval zebrafish from calcium imaging data. Standard vector autoregressive models are limited by their linearity assumptions, and general-purpose, large-scale statistical models such as, for example, LSTM networks, may require large amounts of training data, not always readily available in biological applications, in order to capture complex nonlinear dynamical processes; moreover, such models generally lack interpretability. In this work, we introduce a novel approach for learning a nonlinear differential equation model, which describes calcium dynamics in the brain using both observed voltage-like variables, reflecting voxel activity, and hidden recovery-like variables, reflecting excitability. Namely, we propose a variable-projection optimization approach to estimate the parameters of the multivariate (coupled) van der Pol oscillator, and demonstrate that the proposed model can accurately capture the nonlinear dynamics of the brain data. Furthermore, in order to improve the predictive accuracy when forecasting future brain-activity time series, we propose to use our analytical model as an unlimited source of simulated data for LSTM pretraining, effectively imposing an oscillator prior on LSTM, which improves the predictive performance of both LSTM and our van der Pol models.

## P172 A cross-platform real-time model library to build hybrid neural circuits

### Rodrigo Amaducci, Manuel Reyes-Sanchez, Irene Elices Ocon, Francisco B Rodriguez, Pablo Varona

#### Universidad Autónoma Madrid, Ingeniería Informática, Madrid, Spain

##### **Correspondence**: Pablo Varona (pablo.varona@uam.es)

*BMC Neuroscience* 2018, **19(Suppl 2):**P172

Studying neural systems is a complex task due to their complicated non-linear dynamics, affected by multiple learning and adaptation mechanisms, and because they process information in very different spatial and temporal scales. In addition, the inability to simultaneously observe more than a few signals of the ones involved in these dynamics makes these systems only partially observable. In this context, the traditional stimulus–response paradigm prevents a full characterization of the non-stationary neural activity, influenced by the previous situation and feedback. Closed-loop technology in experimental neuroscience provides novel ways of bidirectional interaction with neural systems, as well as online observation and control, that can largely overcome such difficulties [1]. One example of effective closed-loop implementations are hybrid circuits between living neurons and computational models. In this work we introduce RTHybrid, a cross-platform real-time software model library designed to build hybrid circuits, which is also open-source. To comply with the precise temporal restrictions of data acquisition and activity-dependent stimulation, in the scale of milliseconds or lower, a real-time system is needed [2–5]. RTHybrid is a multiplatform tool, developed to run over two of the currently most common open-source real-time solutions for Linux: Xenomai and Preempt-RT. It has a GUI to facilitate the experimental design and where the hybrid circuit parameters can be chosen to launch the hybrid interaction. Moreover, hybrid protocols can be run from command line loading XML configuration files to implement experiment automation, and uses open-source drivers for National Instruments’ (and several other manufacturers’) DAQ devices. Due to the temporal thresholds established in closed-loop interactions, models, both neural and synaptic, computational cost must be constrained. Spatial and temporal scale differences among the living neurons and the models also present some technical complications that should be addressed. RTHybrid contains a convenient set of neuron and synapse models, as well as automatic calibration algorithms, that meet all the mentioned requirements. We report hybrid circuit functionality and latency measurement validation tests, in CPG circuits. Many researchers and laboratories overlook closed-loop technology despite its benefits due to the difficulties in installation, design and implementation. These handicaps can be surpassed by standardization of real-time software for experimental neuroscience. RTHybrid is a first step in developing a cross-platform and user-friendly real-time multipurpose software tool to implement open and closed-loop experiments, available as open-source software for neuroscientists.

**Acknowledgements**

This work was supported by MINECO/FEDER DPI2015-65833-P, TIN2014-54580-R, TIN2017-84452-R and ONRG grant N62909-14-1-N279

**References**

1. Varona P, Arroyo D, Rodriguez FB, Nowotny T. Online event detection requirements in closed-loop neuroscience. in *Closed*-*Loop Neuroscience*, A. El Hady, Ed. Academic Press, 2016, pp. 81–92.
2. Muniz C, Rodriguez FB, Varona P. *BMC Neurosci*. 2009;10:P49.
3. Biró I, Giugliano M. Front. *Neuroinform*. *Frontiers*; 2015;9:17.
4. Patel YA, George A, Dorval AD, et al. *PLOS Comput*. *Biol*. 2017, 13(5), p. e1005430.
5. Amaducci R, Muñiz C, Reyes-Sanchez M, Rodriguez FB, Varona P. *BMC Neuroscience 2017*, 18 (1), P104.

## P173 Unveiling and characterizing dynamical invariants in central pattern generators

### Irene Elices Ocon^1^, Manuel Reyes-Sanchez^1^, Rodrigo Amaducci^1^, Rafael Levi^2^, Francisco B Rodriguez^1^, Pablo Varona^1^

#### ^1^Universidad Autónoma Madrid, Ingeniería Informática, Madrid, Spain; ^2^University of Southern California, Department of Biological Sciences, Los Angeles, CA, United States

##### **Correspondence**: Irene Elices Ocon (irene.elices@uam.es)

*BMC Neuroscience* 2018, **19(Suppl 2):**P173

Many neural rhythms that present sequential activation of its constituent elements use inhibition as the main mechanism to shape rhythmic activity [1], allowing neurons to express their excitability in specific time windows, and effectively balancing the robustness of the sequence and the flexibility to tune the phases. For example, in cases such as the crustacean pyloric Central Pattern generator (CPG), in addition to keeping the sequence [2, 3], phase adaptations are most likely essential for optimizing the function of the motor plant which moves and processes food. Thus, it is important not only to control the sequence of elements, but also to tune actual phase intervals in which neurons are active within a rhythm.

The variability of the pyloric CPG activity was assessed under different conditions by measuring precise time references such as the first and last spike within bursts. These references were used to define time intervals to quantify the rhythm variability. This analysis demonstrated a large flexibility in several intervals and a strong robustness of the circuit in keeping not only the activation sequences but also specific temporal relationships. In particular, we report the presence of dynamical invariants in the form of strong correlations between specific time intervals [4].

A conductance-based model of the pyloric CPG was used to assess the role of the asymmetric connectivity between the neurons in shaping the rhythm and keeping the LP-PYs-PDs sequences even in irregular bursting regimes [4, 5]. The model does not reproduce the invariants found in the experiments despite of the introduction of variability by setting the model dynamics in a chaotic regime, indicating that the flexibility required to build the invariants is not captured by commonly used conductance-based models.

We also designed and implemented hybrid circuits by connecting neuron models to the living CPG circuit to better understand the origin and relevance of the unveiled dynamical invariants and their role in balancing flexibility and robustness in the rhythm [3, 6, 7]. We show that as a function of the connectivity parameters the dynamical invariants do arise between living and model neurons.

Dynamical invariants as the ones observed in CPGs, underlie rhythm programming and functionality and could be present in other networks, including those related to brain rhythms in vertebrates.

**Acknowledgements**

Funded by MINECO/FEDER DPI2015-65833-P/TIN2014-54580-R/TIN2017-84452-R (http://www.mineco.gob.es/) and ONRG grant N62909-14-1-N279.

**References**

1. Buzsáki G, Watson BO. Dialogues in Clinical Neuroscience 2012 dec; 14(4):345–67.
2. Marder E, Calabrese RL. *Physiol Rev* 1996, 76:687–717.
3. Selverston AI, Rabinovich MI, Abarbanel HDI, Elson R, Szücs A, Pinto RD, Huerta R, Varona P. J Physiol 2000, 94:357–374.
4. Elices I., Arroyo D., Levi R., Rodríguez F.B, Varona P. *BMC Neuroscience 2017*, 18 (Suppl 1):P282 (CNS 2017, Antwerp, Belgium).
5. Elices I, Varona P. *Front Comput Neurosci* 2017, 11:9.
6. Reyes-Sanchez M., Elices I., Amaducci R., Muniz C., Rodriguez F.B., Varona P. *BMC Neuroscience 2017*, 18 (Suppl 1):P281 (CNS 2017, Antwerp, Belgium).
7. Amaducci R., Muñiz C., Reyes-Sanchez M., Rodriguez F.B., Varona P. *BMC Neuroscience 2017*, 18 (Suppl 1):P104 (CNS 2017, Antwerp, Belgium).

## P174 Point process-based dynamic functional connectivity with source-reconstructed EEG data

### Katharina Glomb^1^, David Pascucci^2^, Sebastien Tourbier^1^, Margherita Carboni^3^, Maria Rubega^4^, Serge Vulliemoz^3^, Gijs Plomp^2^, Patric Hagmann^1^

#### ^1^CHUV, Department of Radiology, Lausanne, Switzerland; ^2^University of Fribourg, Department of Psychology, Fribourg, Switzerland; ^3^University Hospital of Geneva & University of Geneva, Department of Fundamental Neurosciences, Geneva, Switzerland; ^4^University of Geneva, Department of Fundamental Neurosciences, Geneva, Switzerland

##### **Correspondence**: Katharina Glomb (katharina.glomb@upf.edu)

*BMC Neuroscience* 2018, **19(Suppl 2):**P174

“Ignition” [1] refers to the ability of local neural events to propagate through a network and alter its global configuration. Ignition has been observed on virtually all scales of spatial and temporal resolution. Deco et al. [2] developed a method which uses a point process [3] to define spontaneous events on the ultraslow timescale of fMRI. We extend their approach to EEG in order to investigate ignition dynamics on the millisecond timescale. We combine the temporal resolution of high-density EEG, EEG source imaging, fMRI and diffusion tensor imaging (DTI). In particular, we introduce a dynamic functional connectivity (FC) measure based on the idea of ignition to investigate network dynamics in response to coherent dot kinematograms (RDK) in 19 healthy subjects engaged in a motion discrimination task. EEG and MRI (T1- and T2-weighted, DTI) data were acquired for each subject on separate sessions. A distributed linear inverse solution (LAURA) (Cartool software [4]), was used to estimate source activity from EEG epochs time-locked to the RDK onset (− 500 to 1000 ms). Subject-specific source reconstructions were informed by individual T1 images [5]. DTI data were used to obtain structural connectomes (SC). Source waveforms were extracted from 14 regions of interest (ROI) functionally defined with an fMRI motion localizer [6]. We first focus on the alpha band (8–15 Hz), one of the predominant frequencies of brain rhythms and neuronal communication [7]. We obtain band-limited power (BLP) via Hilbert transform and define a point process for each ROI: We take as “events” the time points at which the BLP crosses a threshold (1 standard deviation above the mean) from below. FC is computed based on the assumption that two ROIs presenting events within a certain delay d ∈]0,D] are functionally connected. The maximum delay D = 25 ms was derived from the maximum average fiber length between any pair of ROIs. Furthermore, connections not supported by the SC are removed [8]. The spatiotemporal profile of network activity was evaluated by computing the overall inflow (events within the network that precede an event in a given ROI) and outflow (events within the network that follow an event in a given ROI) for each ROI over time. This reveals an evolution in time from early local dynamics in occipital areas toward later global interactions reaching regions of the parietal and prefrontal cortex. An additional peak around 200 ms may reflect the recurrent processing of motion signals and the shift from functionally specialized areas (e.g., middle-temporal cortex/V5) into integrative networks and decision-making circuits [10, 11]. These preliminary results show that dynamic FC informed by local point processes can be used to capture important spatiotemporal information about perceptual processing, promising the investigation of ignition dynamics from a network perspective on a finer temporal scale.

**References**

1. Moutard C, Dehaene S, Malach R. Spontaneous fluctuations and non-linear ignitions: two dynamic faces of cortical recurrent loops. *Neuron* 2015, 88, 1, 194–206.
2. Deco G, et al. Novel intrinsic ignition method measuring local–global integration characterizes wakefulness and deep sleep. *Eneuro* 2017, 4, 5, ENEURO-0106.
3. Tagliazucchi E, et al. Criticality in large-scale brain fMRI dynamics unveiled by a novel point process analysis. *Frontiers in physiology* 2012, 3, 15.
4. Brunet D, Murray MM, Michel CM. Spatiotemporal analysis of multichannel EEG: CARTOOL. *Computational Intelligence and Neuroscience* 2011, 2.
5. de Peralta M, Grave R, et al. Electrical neuroimaging based on biophysical constraints. *Neuroimage* 2004, 21, 2, 527–539.
6. Berman RA., et al. Cortical networks subserving pursuit and saccadic eye movements in humans: an FMRI study. *Human brain mapping* 1999, 8, 4, 209–225.
7. Fries P. A mechanism for cognitive dynamics: neuronal communication through neuronal coherence. *Trends in cognitive sciences* 2005, 9, 10, 474–480.
8. Fries P. Rhythms for cognition: communication through coherence. *Neuron* 2015, 88, 1, 220–235.
9. Griffa A et al. Transient networks of spatio-temporal connectivity map communication pathways in brain functional systems. *Neuroimage* 2017, 155, 490–502.
10. Mercier M, et al. Motion direction tuning in human visual cortex. *European Journal of Neuroscience* 2009, 29, 2, 424–434.
11. Wang, X. Decision making in recurrent neuronal circuits. *Neuron* 2009, 60, 2, 215–234.

## P175 Modeling the spatial inhomogeneous degradation of nitric oxide shows a key role of anatomically localized NO production

### William Haselden, Ravi Kedarasetti, Patrick Drew

#### Pennsylvania State University, Engineering Science and Mechanics, State College, PA, United States

##### **Correspondence**: William Haselden (william.alkenakou@gmail.com)

*BMC Neuroscience* 2018, **19(Suppl 2):**P175

The interaction between neural activity and the hemodynamic response is known as neurovascular coupling (NVC). Proper NVC is a factor in maintaining brain health and can occur through multiple signaling pathways that are targets of ongoing research. Nitric oxide (NO) is a potent vasodilator [2, 6, 7] and neural activity modulator [1, 3, 4, 5, 8] that can freely diffuse across cell membranes and carry signals between neurons and vessels. NO released from neurons vasodilates arteries before being degraded by its interaction with hemoglobin (Hb) in the blood. The dynamics of NO-mediated NVC is dependent on the location and magnitude of NO production and degradation as well as its diffusion through tissue. Physiological NO concentrations are lower bound by production rates that cause dilation of the smooth muscle and upper bound by its toxic inhibition of cytochrome c oxidase (CcO). Using a computational model of NO diffusion we show that in order to generate physiologically plausible dilations without toxic inhibition of CcO, proximal production of NO is required. Additionally we show that vessel size is an important factor in determining arteriole sensitivity to NO and can account for larger dilations observed in smaller arteries as well as its dysregulation in diseased states. We also investigate how vasodilation can affect the concentration of NO in brain tissue.

**References**

1. Cudeiro J et al. Actions of Compounds Manipulating the Nitric Oxide System in the Cat Primary Visual Cortex. *Journal of Physiology* 2004, 504(2), 467–78.
2. Forstermann UA, Mulsch E, Bohme et al. Stimulation of Soluble Guanylate Cyclase by an Acetylcholine-Induced Endothelium-Derived Factor from Rabbit and Canine Arteries. *Circulation Research* 1986, 58(4): 531–38.
3. Garthwaite, J. Concepts of Neural Nitric Oxide-Mediated Transmission. *European Journal of Neuroscience* 2008, 27(11), 2783–2802.
4. Kano T, et al. Effects of Nitric Oxide Synthase Gene Knockout on Neurotransmitter Release in Vivo. *Neuroscience* 1998, 86(3): 695–99.
5. Kara P, Friedlander MJ. Arginine Analogs Modify Signal Detection by Neurons in the Visual Cortex. *J Neurosci* 1999, 19(13): 5528–48.
6. Moncada SRM, Palmer, Higgs EA. Nitric Oxide: Physiology, Pathophysiology, and Pharmacology. *Pharmacological Reviews* 1991, 43(2): 109–42.
7. Rapoport RM, Draznin MB, Murad F. Endothelium-Dependent Relaxation in Rat Aorta May Be Mediated through Cyclic GMP-Dependent Protein Phosphorylation. *Nature* 1983, 306(5939): 174–76.
8. Smith SL, Otis TS. Persistent Changes in Spontaneous Firing of Purkinje Neurons Triggered by the Nitric Oxide Signaling Cascade. *Journal of Neuroscience* 2003, 23(2): 367–72.

## P176 A Bayesian, biophysical framework for spike sorting

### Kevin Lin, Patrick Greene

#### University of Arizona, Department of Applied Mathematics, Tucson, AZ, United States

##### **Correspondence**: Kevin Lin (klin@math.arizona.edu)

*BMC Neuroscience* 2018, **19(Suppl 2):**P176

Extracellular electrical recordings are a common tool in experiments involving awake, behaving animals. Using such recordings to detect neural correlates of behavior often requires*spike sorting*, i.e., labeling each spike by the (putative) identity of the cell that generated it. A common method is to detect spikes by thresholding, then perform spike-sorting via, e.g., clustering waveform feature vectors or template-matching on the waveforms [1]. Though such methods can be relatively fast, they have a number of known limitations, including (i) difficulty sorting spikes that overlap in time, (ii) a bias toward cells with larger spikes, and (iii) difficulty in systematically quantifying the uncertainty in spike assignments [2]. We propose a physics-based approach to spike detection and sorting within a Bayesian statistical framework designed to address these issues. As in all Bayesian methods, the goal is to find the model parameters that optimize the posterior probability given the observed data. In spike sorting, these model parameters would include spike-to-cell assignments and spike waveforms associated to each cell [2]. Our point of departure from previous methods is the discovery, due to Mechler et. al., that data from n-trode (e.g., tetrode) recordings can be used to “triangulate” the source of spikes because the contacts are at slightly different distances to signal sources [3, 4]. Their method relies on a dipole model of spike-to-probe signal transduction. Using this idea, we augment cell IDs by source location, dipole orientation and amplitude, and spike times That is, we construct a computational model for how a small number of current dipoles can account for the observed data, and optimize the model parameters to fit the data. Mathematically, this takes a form similar to a gaussian mixture model (GMM), but one in which every data sample is represented as a sum of waveforms from some subset of the mixture components. This allows us to take into account overlapping spikes and smaller spikes near the noise floor. We test our algorithm using simulated data from a separate extracellular recording and noise model that we have constructed, allowing us to determine sorting and localization accuracy with respect to a known ground truth. We find that our algorithm is usually able to localize neurons to within approximately 30 um of their true position, and achieve sorting accuracy rates roughly 20 percentage points better than a vanilla GMM-based clustering method that uses the PCA components of the waveform. However, in situations where localization cannot be done accurately, the algorithm becomes less effective (Fig. 1).

**Fig. 1:**
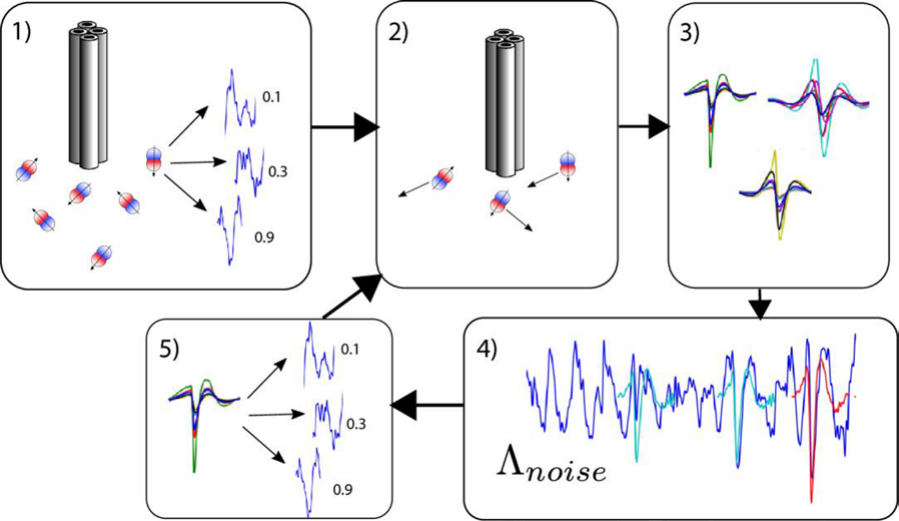
Algorithm overview: (1) From initial positions, weight data samples by the probability of being produced by a dipole at each position, using the forward model. (2) Use these weighted samples to update the dipole and position for each neuron. Estimate each neuron’s spiking probability from the number of spikes assigned to it. (3) Calculate the waveforms generated by these dipoles. (4) Subtract off waveforms from the signal to estimate the noise covariance. (5) Update the data sample weights using the waveforms, and return to step 2)

**References**

1. Rey, H.G., et al. Past, present and future of spike sorting techniques. *Brain Res*. *Bull*. 2015, 119: 106–117.
2. Lewicki, M.S. A review of methods for spike sorting: the detection and classification of neural action potentials. *Network: Comput*. *Neural Syst*.1998, 9: R53-R78.
3. Mechler, F. and Victor, J. Dipole characterization of single neurons from their extracellular action potentials. *J*. *Comput*. *Neurosci*. 2012, 32: 73–100, 2012.
4. Mechler, F. et al. Three-dimensional localization of neurons in cortical tetrode recordings. *J*. *Neurophysiol*. 2011 106: 828–848.

## P177 A detailed model of the hippocampal formation for the generation of Sharp-Wave Ripples and Theta-nested Gamma oscillations

### Amelie Aussel^1^, Radu Ranta^1^, Laure Buhry^1^, Louise Tyvaert^2^, Patrick Henaff^1^

#### ^1^Université de Lorraine, CRAN UMR 7039, Nancy, France; ^2^University Hospital (CHU) Nancy, Nancy, France

##### **Correspondence**: Amelie Aussel (amelie.aussel@loria.fr)

*BMC Neuroscience* 2018, **19(Suppl 2):**P177

The hippocampus can exhibit different oscillatory rhythms within the sleep–wake cycle, each of them being involved in cognitive processes. For example, theta-nested gamma oscillations produced during wakefulness are associated with spatial navigation and working memory tasks, whereas Sharp-Wave-Ripple (SWR) complexes produced during slow-wave sleep play an important role in memory consolidation. Different factors might be involved in the transitions between these rhythms, such as a change in the functional connectivity within the hippocampus, the presence of some rhythm regulator persistent firing neurons or even the entries from the afferent structures. Some neuromodulators, such as Acetylcholine (ACh), whose concentration is higher during wakefulness than sleep, were proposed by different authors for explaining the connectivity variations [1][2], as well as influencing the persistent firing of neurons through CAN channels [3][4]. But though we understand the influence of ACh on individual cells for different receptor types and locations [5], its quantitative effects on the whole hippocampal network remain unclear. In this context, we have built a computational model of the hippocampal formation considering the varying concentration of ACh as well as different input signals. Our model uses point neural models (single-compartment) but having realistic dynamics (conductance-based Hodgkin-Huxley neurons, including CAN channels). Based on [1], the network functional connectivity was also changed between wakefulness and slow-wave sleep by varying the weights of some synaptic connections. Moreover, the stimulation entry of the network was derived from real sEEG measurements recorded during wake/sleep in the prefrontal cortex (projecting on the entorhinal cortex) of epileptic patients from University Hospital of Nancy, France. The output of the model was simulated at different levels (spiking dynamics and field potentials). In order to obtain a reasonable approximation of the LFP of the whole population, as measured by a macroscopic electrode placed within the network, the microscopic anatomy of the neurons was approximated by a dipole, while the macroscopic anatomy of the hippocampal structure was reproduced by positioning and connecting the neurons in an anatomically realistic manner. This output was compared with in vivo sEEG signals from the human hippocampus. Our main finding is that such a model can indeed reproduce both theta-nested gamma oscillations and SWR complexes in humans by changing the level of ACh, with but little influence of the input stimulus. The network functional connectivity seems to determine the high frequency component of the rhythms, whereas individual neurons’ CAN channel conductance influence its low frequency component (Fig. 1).

**Fig. 1:**
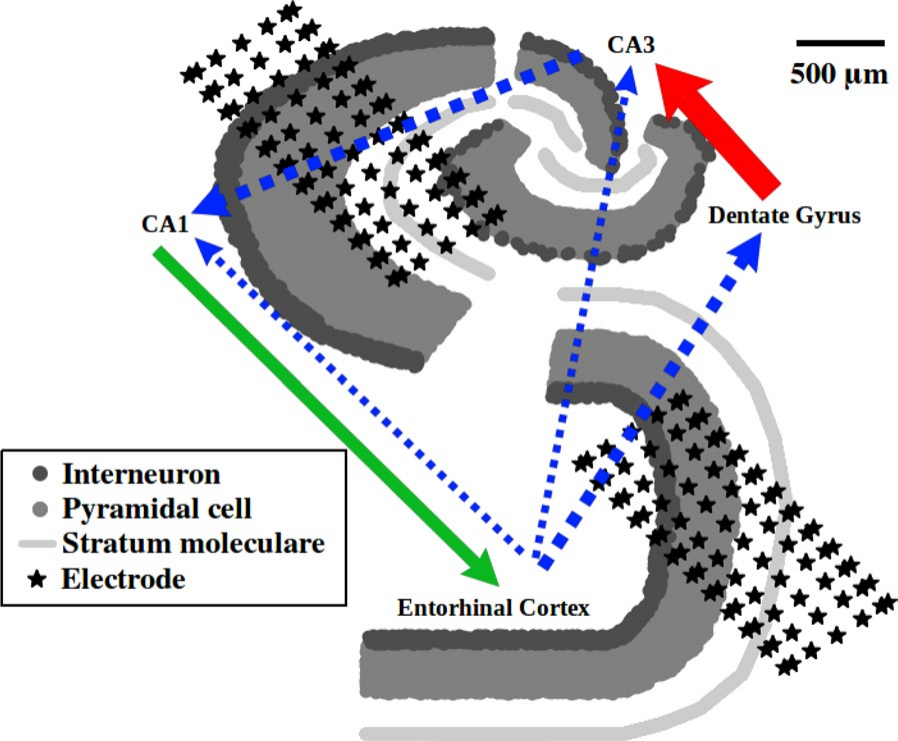
Topology of the entorhinal cortex and the hippocampus used in the model. The arrows represent the functional connectivity between the different regions (thicker arrow indicate more numerous synaptic connections) : the red arrow indicates a connection that is enhanced by ACh, blue dotted arrows indicate a connection that is reduced by ACh, and the green arrow indicates a connection that is not changed by ACh

**References**

1. Hasselmo ME. Neuromodulation:acetylcholine and memory consolidation. *Trends Cogn Sci* 1999, 3(9):351–359.
2. Platt B, Riedel G. The cholinergic system, EEG and sleep. *Behav Brain Res* 2011, 221(2):499–504.
3. Jochems A, Yoshida M. Persistent firing supported by an intrinsic cellular mechanism in hippocampal CA3 pyramidal cells. *Eur*. *J*. *Neurosci*. 2013, 38(2):2250–2259.
4. Giovannini F, Knauer B, Yoshida M, Buhry L. The CAN-in network: A biologically inspired model for self-sustained theta oscillations and memory maintenance in the hippocampus. *Hippocampus* 2017, 27(4):450–463.
5. Drever BD, Riedel G, Platt B. The cholinergic system and hippocampal plasticity. *Behav Brain Res* 2011, 221(2):505–514.

## P178 A mechanistic model explains auditory evoked responses as a reflection of network properties of the entire auditory cortex

### Artur Matysiak^1^, Aida Hajizadeh^1^, Nina Härtwich^1^, Reinhard König^1^, Patrick May^2^

#### ^1^Leibniz Institute for Neurobiology, Special Lab for Non-Invasive Brain Imaging, Magdeburg, Germany; ^2^Lancaster University, Department of Psychology, Lancaster, United Kingdom

##### **Correspondence**: Artur Matysiak (amatysia@lin-magdeburg.de)

*BMC Neuroscience* 2018, **19(Suppl 2):**P178

Magnetoencephalography (MEG) is a widely used non-invasive brain imaging technique whose high temporal resolution makes it ideal for exploring the operation of the auditory system. Individual auditory stimuli elicit a series of peaks and troughs in the event-related magnetic field (ERF), where the most prominent peak, the N1 m, shows sensitivity to nearly every stimulus property that has been examined. The peaks and troughs of the ERF function as landmarks as they can be identified in the ERF of most subjects, albeit they vary greatly from subject to subject. Despite improvements in localization methods—and although the general biophysics of MEG generation is well known—we still have a poor understanding of how ERFs are generated and what they signify. According to one view, the response is the linear sum of separable components, each generated by a spatially defined generator with a well-defined information processing function. However, it has proven difficult to perform component separation in a reliable way and to map components to anatomical structure. Similarly, it seems unlikely that purely local events in, for example, primary fields could represent the full intracortical counterpart of ERFs, which emerge as a superposition of activity across larger swathes of cortex. Thus, cortical activations generating ERFs should have both spatial and temporal dynamics. Here, we provide a mechanistic view on how AC responds to sound. Our starting point is a previous model encapsulating the anatomical structure of AC with its multiple core, belt, and parabelt fields. Taking an analytical approach, we find solutions to the AC system dynamics at the cost of a simplified description. This includes simplified presynaptic plasticity and assumes that the state variables inhabit the linear portion of the spiking-rate non-linearity. The linearization of the spiking rate together with assumptions of symmetry of the weight matrices allows us to transform the coupled state equations into decoupled equations which are analytically solvable and provide a description of the system dynamics in terms of so called normal modes. We show how the ERF response originates from a mixture of these dynamical elements, and how these elements directly depend on the anatomical structure as expressed in the weight matrices. There is also a non-dynamical modulation of the signal which accounts for the topography of the primary currents and therefore includes the effects of connection type (i.e., feedforward vs. feedback) as well as the orientation of the primary currents and their distance from the MEG sensor. In our account, each peak and trough of the ERF is not due to a dedicated response generator but, rather, arises out of the network properties of the entire AC. This analysis also generates several predictions for teasing out whether the large variations of the ERF across individual subjects is due to subject-specific topography of the cortical surface, to subject-specific cortical dynamics, or to a mixture of these effects. Finally, our results point to the interplay between synaptic plasticity, tonotopic representation, and the analysis of time information in cortex forming a richer tapestry of interactions.

## P179 Noisy deep networks with short-term plasticity make similar errors as mice in a detection of change task

### Jiaqi Shang^1^, Brian Hu^2^, Shawn Olsen^2^, Stefan Mihalas^2^, Doug Ollerenshaw^2^, Marina Garrett^2^, Justin Kiggins^2^, Peter Groblewski^2^

#### ^1^Northwestern University, Northwestern University, Evanston, IL, United States; ^2^Allen Institute for Brain Science, Modelling, Analysis and Theory, Seattle, WA, United States

##### **Correspondence**: Brian Hu (brianh@alleninstitute.org)

*BMC Neuroscience* 2018, **19(Suppl 2):**P179

The ability to detect changes in an environment is fundamental for survival in a dynamic world. However, the neural mechanisms underlying this ability are not well understood. We developed a new behavioral paradigm that systematically measures the neural activity in the visual cortex of awake, behaving mice during a change detection task. During the task, the mice view a sequence of eight natural images separated by blank screens and reports if the current image is different from the last image presented. Mice are able to learn the task and generalize well to untrained image sets. Preliminary results showed a robust behavioral asymmetry across animals and image sets. Specifically, the response probability matrix (Fig. 1, panel a) shows that for most image pairs A and B, the transition from A to B is not the same as B to A. One hypothesis for this behavioral asymmetry is that differences in contrast at spatial frequencies mice are most sensitive to cause short-term depression on specific pathways. To test this hypothesis, and to understand the overall underlying mechanism that gives rise to a decision during the task, we built a computational model that is trained on the same task. First, the model uses a 4-layer convolutional neural network (CNN) trained on CIFAR-10 image dataset to embed the natural images used in the experiment into a low-dimensional (84) input for the decision module. Gaussian noise is added to each layer of the network to simulate biological noise in the system. Then, synaptic depression is added to the CNN output by multiplying it with a synaptic efficacy function that includes both usage and recovery rates. Lastly, the decision module uses a simple network with one hidden layer to perform a binary classification and generate outputs that correspond to go or no-go behavioral decisions. The model learns the task, as shown by the transition matrix (Fig. 1, Panel b) that has lower response probabilities along the diagonal where the input images is a repetition of the last image. We are able to generally reproduce the behavioral asymmetry observed in behavioral experiments (compare Fig. 1, panels a and b). Interestingly, for our model to match behavioral data, we had to put a strong penalty on missed detections. We also find that models without synaptic depression are unable to learn the task and do not show the behavioral asymmetry observed in the experiments. We believe that short-term depression can be used by the model as a regularizer that allows the large space of images to be separated into much lower dimensions. We will investigate whether the model learned on training sets can generalize to novel images sets, as mice are capable of doing. Finally, we will study how recurrent neural networks, which have a memory module, can be used to unravel the mechanism behind this task, and more generally to detect change.

**Fig. 1:**
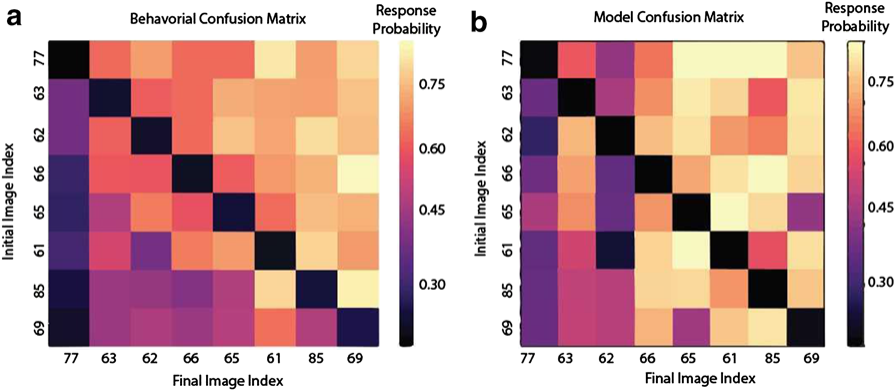
Quantification of behavior during a change detection task. Mice are presented a sequence of grayscale natural images, each for 250 ms. Between each image, mice are shown a gray screen for 500 ms. Animals are trained to respond when the current image they observe is different from the last image they saw. (a) Confusion matrix averaged over multiple mice, where rows show the initial image index and columns show the final image index after the change. Warmer colors indicate higher response probabilities. (b) Confusion matrix for the model, with the same image ordering as for the mice

## P180 Statistical properties of strengths of structural and functional connectivity

### Xiao Gao, Peter Robinson

#### The University of Sydney, School of Physics, Sydney, Australia

##### **Correspondence**: Xiao Gao (demi.gao@sydney.edu.au)

*BMC Neuroscience* 2018, **19(Suppl 2):**P180

To better understand how brain processes inputs and performs tasks rapidly, how it maintains stability while preventing the spread of undesirable activity such as seizures, how it develops, and how it responds to damage have provided much of the motivation for the current brain research. The brain connectivity is often quantified via connectivity matrices (CMs). In particular, anatomical CMs (aCMs) summarize the known anatomical connectivity between brain regions, functional CMs (fCMs) are determined from the correlation of activity in brain regions using low-frequency functional magnetic resonance imaging (fMRI), and effective CMs (eCMs) quantify the neural effect of one region to another. Previously, a method based on neural field theory (NFT) for computing eCMs from the experimental fCMs was developed which has successfully detected the underestimated strengths of connections in the anatomical images. However, we do not yet fully understand the statistical properties of connectivity, e.g., some of the functional connections appear to be negative even when anatomical connections are all positive. In this study, a theory aim to explain the observed statistical properties of strengths of brain connectivity is expanded using NFT. The theory shows, first, how the observed lognormal distribution of overall connection strengths, as well as the lognormal distribution of connection strengths at each fixed Euclidean distance between brain regions can emerge from the approximately exponential fall-off with distance of axonal connectivity; second, how observed negative connection strengths are incorporated in the derived eCMs; and third, how to better interrelate the structural connectivity to the functional connectivity. The theory is verified using NKI-Rockland fMRI dataset.

**Acknowledgements**

This work was supported by the Australian Research Council Center of Excellence for Integrative Brain Function (ARC Grant CE140100007), ARC Laureate Fellowship Grant FL1401000225, and ARC Federation Fellowship Grant FF0883155.

## P181 Plasticity of information coding by cerebellar Purkinje cells during sensorimotor learning

### Sungho Hong^1^, Erik De Schutter^1^, Akshay Markanday^2^, Ayaka Usui^3^, Peter Thier^2^

#### ^1^Okinawa Institute of Science and Technology, Computational Neuroscience Unit, Okinawa, Japan; ^2^University of Tübingen, Hertie Institute for Clinical Brain Research, Department of Cognitive Neurology, Tübingen, Germany; ^3^Okinawa Institute of Science and Technology, Quantum Systems Unit, Okinawa, Japan

##### **Correspondence**: Sungho Hong (shhong@oist.jp)

*BMC Neuroscience* 2018, **19(Suppl 2):**P181

The cerebellum is a brain region best known for its role in sensorimotor coordination and is involved in rapid learning of precise movements. In classical theories on cerebellar motor learning, the most important role is played by the Purkinje cells (PC), which fire a burst called a complex spike (CS) from a climbing fiber input that delivers an error feedback signal, and the CS triggers intracellular processes inducing plasticity in PC coding of the sensorimotor information, and therefore leading to learning [1]. However, a precise role of CSs still remains as a question since experimental evidence shows that the CS frequency does not reflect the size of a motor error, therefore contradicting this view [2]. Here we address this question by analyzing simultaneous recordings of eye movements and PC firings in the oculomotor vermis of three rhesus monkeys (M. Mulatta), which performed saccadic adaptation tasks. Here we used the cross-axis adaptation paradigm where the visual target sequentially jumped twice in two different directions separated by 90º, causing a substantial retinal slip and therefore forcing an animal to learn complex movements to reduce the error [3]. In data sets selected by recording quality and sensitivity of simple spike firing to eye movements, we found that the timing of CSs represented sensory signals, the onsets of target movements. Therefore, the CS firing rate significantly increased within a narrow (~ 100 ms) time window related to the target onset, but the overall spike count during each trial is preserved between adaptation and non-adaptation sessions ([CS rate in adaptation]/[CS rate in pre-/post-adaptation] = 1.00 ± 0.02, mean ± SEM), suggesting that sensory stimuli strongly affect timing of CS firing. On the other hand, simple spike (SS) activity is better explained by the motor context [4–6], and showed movement direction-dependent changes that lasted until post-adaptation sessions. However, these changes could not be explained by simply rotating the directional tuning of the single PC SS activity, in the same way as the eye movements adapted, but rather involved more complex changes in SS bursts and pauses [7]. We suggest that those direction-dependent plasticity in encoding of eye movement by individual PCs reshape the population-wide response during adaptation.

**References**

1. Ito M. Cerebellar circuitry as a neuronal machine. *Prog Neurobiol*. 2006, 78, 272–303.
2. Prsa M, Thier P. The role of the cerebellum in saccadic adaptation as a window into neural mechanisms of motor learning. *Eur J Neurosci*. 2011, 33, 2114–28.
3. Noto CT, Watanabe S, Fuchs AF. Characteristics of simian adaptation fields produced by behavioral changes in saccade size and direction. *J Neurophys*. 1999, 81, 2798–813.
4. Thier P, Dicke PW, Haas R, Barash S. Encoding of movement time by populations of cerebellar Purkinje cells. *Nature*. 2000, 405, 72–6.
5. Herzfeld DJ, Kojima Y, Soetedjo R, Shadmehr R. Encoding of action by the Purkinje cells of the cerebellum. *Nature*. 2015, 526, 439–42.
6. Hong S, Negrello M, Junker M, Smilgin A, Thier P, De Schutter E. Multiplexed coding by cerebellar Purkinje neurons. *Elife*. 2016, 5, 1234.
7. Catz N, Dicke PW, Thier P. Cerebellar-dependent motor learning is based on pruning a Purkinje cell population response. *Proc Natl Acad Sci USA*. 2008, 105, 7309–14.

## P182 A systematic comparison of neural morphology representations in the context of cell type discrimination

### Sophie Laturnus, Ziwei Huang, Philipp Berens

#### Institute of Ophthalmic Research, Neural Data Science for Vision Research, Tuebingen, Germany

##### **Correspondence**: Sophie Laturnus (sophie.laturnus@uni-tuebingen.de)

*BMC Neuroscience* 2018, **19(Suppl 2):**P182

The morphology of neurons is typically considered a defining feature of neural cell types. For example, 14 types of bipolar cells can be discriminated in the mouse retina based on their morphology [1–3], leading to a classification in good agreement with genetic and physiological data [4–5]. Similarly, many retinal ganglion cell types can be discriminated based morphological properties [6]. Given recent advances in automatic reconstruction and crowd-based tracing techniques, the amount of available data is rapidly increasing (see e.g. www.neuromorpho.org). However, machine learning methods to automatically classify neurons based on their morphology often rely on fairly simple representations. Typically, these methods consider the neurite density in three dimensions or low dimensional projections thereof or convert neural morphoplogies into expert-defined summary statistics. The similarity between two neurons can then be measured using simply the euclidean distance in these feature spaces. However, such representations may discard much of the fine-grained information contained in neural morphologies. Here we systematically compare different feature sets based on morphometric statistics or neurite density maps, with techniques based in graph theory and the recently proposed persistence [6]. Using regularized logistic regression we investigate which morphological representations allow reliable discrimination of neural types within meaningfully defined cell classes. To this end, we analyze datasets from retinal bipolar cells and ganglion cells as well as interneurons from the primary visual cortex.

In this context, we introduce *MorphoPy*, a Python toolbox that bundles the state-of-the-art methods for representation and analysis of neural morphologies (https://github.com/berenslab/MorphoPy).

**References**

1. Helmstaedter M, Briggman KL, Turaga SC, et al. Connectomic reconstruction of the inner plexiform layer in the mouse retina. *Nature* 2013, 500, 7461
2. Kim JS, Greene MJ, Zlateski A, et al. Space–time wiring specificity supports direction selectivity in the retina. *Nature* 2014, 509, 7500.
3. Greene MJ, Jinseop SK, Seung HS. Analogous convergence of sustained and transient inputs in parallel on and off pathways for retinal motion computation. *Cell reports* 2016, 14(8), 1892–1900.
4. Shekhar K, Lapan SW, Whitney IE, et al. Comprehensive classification of retinal bipolar neurons by single-cell transcriptomics. *Cell 2016*, 166(5), 1308–1323.
5. Franke K, Berens P, Schubert T, et al. Inhibition decorrelates visual feature representations in the inner retina. *Nature* 2017, 542, 439.
6. Sümbül U, Song S, McCulloch K, et al. A genetic and computational approach to structurally classify neuronal types. *Nature Communications* 2014, 5, 3512.

## P183 Online accurate spike sorting for hundreds of channels

### Baptiste Lefebvre, Olivier Marre, Pierre Yger

#### Institut De La Vision, Computational Neuroscience, Paris, France

##### **Correspondence**: Pierre Yger (pierre.yger@inserm.fr)

*BMC Neuroscience* 2018, **19(Suppl 2):**P183

Understanding how assemblies of neurons encode information requires recording of large populations of cells in the brain. In recent years, multi-electrode arrays and large silicon probes have been developed to record simultaneously from thousands of electrodes packed with a high density. To tackle the fact that these new devices challenge the classical way to perform spike sorting, we recently developed a fast and accurate spike sorting algorithm (available as an open source software, called SpyKING CIRCUS), validated both with in vivo and in vitroground truth experiments. The software, performing a smart clustering of the spike waveforms followed by a greedy template-matching reconstruction of the signal, is able to scale to up to 4225 channels in parallel, solving the problem of temporally overlapping spikes. It thus appears as a general solution to sort, offline, spikes from large-scale extracellular recordings. In this work, we implemented this algorithm in an “online” mode, sorting spikes in real time while the data are acquired, to allow closed-loop experiments for high density electrophysiology. To achieve such a goal, we built a robust architecture for distributed asynchronous computations and we proposed a modified algorithm that is composed of two concurrent processes running continuously: (1) “a template-finding” process to extract the cell templates (i.e. the pattern of activity evoked over many electrodes when one neuron fires an action potential) over the recent time course; (2) a “template-matching” process where the templates are matched onto the raw data to identify the spikes. Templates are updated online with a density-based clustering algorithm adapted for data streams, to keep track of drifts over time. A key advantage of our implementation is to be parallelized over a computing cluster to use optimally the computing resources: all the different processing steps of the algorithms (whitening, filtering, spike detection, template identification and fit) can be distributed according to the computational needs.Our software is therefore a promising solution for future closed-loop experiments involving recordings with hundreds of electrodes.

## P184 Time step sensitivity in large scale compartmental models of the neocortex

### Joshua Crone^1^, David Boothe^1^, Alfred Yu^2^, Kelvin Oie^2^, Piotr Franaszczuk^2^

^1^U.S. Army Research Laboratory, Computational and Information Sciences Directorate, Aberdeen Proving Ground, MD, United States; ^2^U.S. Army Research Laboratory, Human Research and Engineering Directorate, Aberdeen Proving Ground, MD, United States

#### **Correspondence**: Piotr Franaszczuk (pfranasz@gmail.com)

##### *BMC Neuroscience* 2018, **19(Suppl 2):**P184

Identifying an appropriate time step is among the first tasks when numerically solving ordinary differential equations. Often times, it is not possible to determine the optimal time step a priori. This is especially true in complex neuronal simulations, where the discrete nature of spikes and high degree of connectivity between neurons leads to a complex system of ordinary differential equations. For these systems, the time step must be selected based on empirical observation rather than from theoretical deduction. Yet it is crucial that appropriate time steps are determined before trusting the results from a neuronal simulation, as integration errors can have unpredictable consequences on the model’s dynamics. As we show in this work, further complicating the process of determining the optimal time step is the poor transferability in the empirically obtained time steps when scaling up to larger neuronal networks. We show that the time step is not only dependent on the intrinsic properties of the neuron, but are also strongly dependent on the size and connectivity of the network. To explore time step sensitivity, we use a biophysically detailed model of the neocortex adapted from Traub et al. [1] which contains 14 neuron types with 50 to 137 compartments each. Simulations are carried out using the GEneral NEural SImulation System (GENESIS) [2]. Neurons are organized into microcolumns containing up to 21 neurons. These microcolumns are repeated to create models of three system sizes with 65, 1040, and 16640 neurons. The number of synaptic connections for the three system sizes are 6790, 1756170, and 449308275, respectively. To simulate resting neuronal activity, neurons are randomly driven with independent Poisson distributed excitatory postsynaptic potentials with an average firing rate between 1 and 10 Hz. For each system size, the connectivity probabilities and random Poisson inputs are held fixed and simulations are carried out using the exponential Euler integration method with three time step sizes of 50, 5, and 0.5 µs. Average spiking rates for excitatory pyramidal neurons in layer 2/3 (P23RS) and layer 5 (P5IB) and inhibitory neurons in layer 2/3 (I23LTS) and layer 5 (I5LTS) for each time step size are shown in Fig. 1. For the smallest system size with 65 neurons, we do not observe any significant effect of due to altering the time step, suggesting that 50 µs is adequate. However, when increasing the system size, model behavior changes from being dominated by excitatory activity to being dominated by inhibitory activity. Therefore, we are unable to extrapolate the time step convergence study results from the small, 65 neuron model to the large neuronal networks, despite being made up of identical neurons with the same intrinsic properties. Further work is ongoing to elucidate why smaller time steps are required for larger system systems, but early evidence suggest it is due stacking of input spikes, which becomes more frequent as the number of neurons and synaptic connections increases. Work is also underway to compare the results with implicit integration methods.

**Fig. 1:**
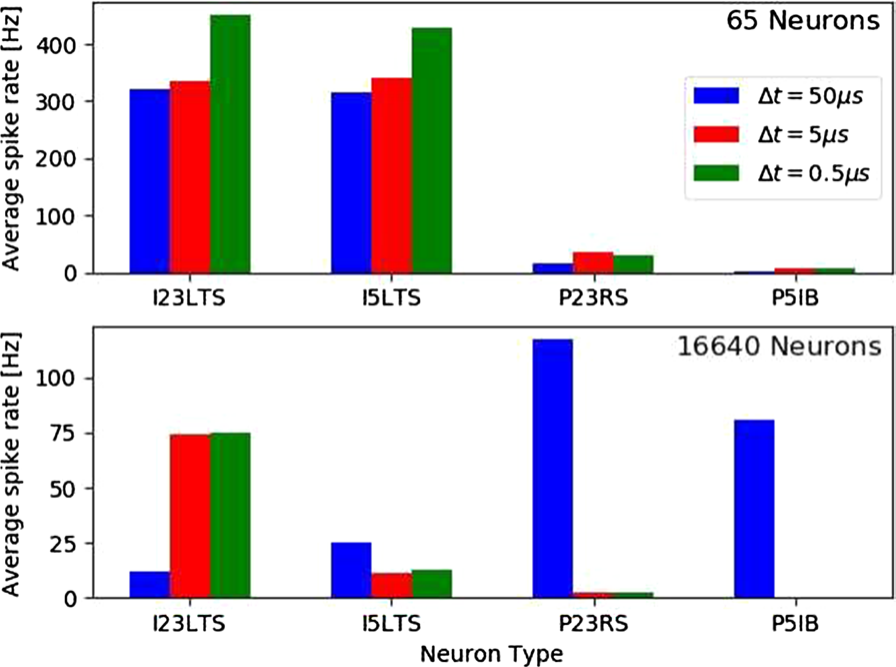
Average spiking rates for excitatory pyramidal neurons in layer 2/3 (P23RS) and layer 5 (P5IB) and inhibitory neurons in layer 2/3 (I23LTS) and layer 5 (I5LTS) for the three time step sizes used in this study

**References**

1. Traub RD, Contreras D, Cunningham MO. Single-column thalamocortical network model exhibiting gamma oscillations, sleep spindles, and epileptogenic bursts. *Journal of Neurophysiology* 2005, 93(4), 2194–2232.
2. Bower JM, Beeman D. *The Book of GENESIS*. USA:NY, Springer-Verlag.

## P185 Electrical coupling of perisomatic and distal apical regions of a layer 5 pyramidal neuron compartmental model

### Melvin Felton^1^, Alfred Yu^2^, David Boothe^1^, Kelvin Oie^2^, Piotr Franaszczuk^2^

#### ^1^U.S. Army Research Laboratory, Computational and Information Sciences Directorate, Aberdeen Proving Ground, MD, United States; ^2^U.S. Army Research Laboratory, Human Research and Engineering Directorate, Aberdeen Proving Ground, MD, MD, United States

##### **Correspondence**: Piotr Franaszczuk (pfranasz@gmail.com)

*BMC Neuroscience* 2018, **19(Suppl 2):**P185

Perisomatic and distal apical regions of layer 5 pyramidal neurons are viewed as two distinct “zones” that mediate action potential initiation. Electrical coupling of these two zones plays a functional role in the associative processing attributed to these neurons because it allows them to detect coincident input to their perisomatic and distal apical regions [1]. However, while it is known that layer 5 pyramidal neurons have inhomogeneous distributions of voltage-dependent frequency preferences that are established by a variety of ionic conductances [2, 3], it is not well known what role subthreshold electrical coupling between perisomatic and distal apical regions could play in multi-frequency coupling in the brain. To assess the subthreshold interaction between perisomatic and distal apical zones, we characterized the resonance properties of a biophysically-realistic compartmental model of a neocortical layer 5 pyramidal neuron. Consistent with recently published theoretical and empirical findings, our model was configured to have a “hot zone” in distal apical dendrite and apical tuft where both high- and low-threshold Ca2+ ionic conductances had densities 1–2 orders of magnitude higher than anywhere else in the apical dendrite [4]. We simulated injection of “chirp” currents with linearly increasing frequency to calculate the transfer impedance between the soma and distal apical dendrite/tuft, and a dimensionless term we introduce called resonance quality. Transfer resonance analysis revealed that changes in subthreshold electrical coupling were found to modulate the transfer resonant frequency of signals transmitted from distal apical dendrite and apical tuft to the soma, which would impact the frequencies that individual neurons are expected to respond to and reinforce. We used the insights from transfer resonance analysis to demonstrate phase-locking of somatic frequency preference for distal apical input to a somatic modulating signal. Specifically, we simulated injection of a slow sinusoidal modulating signal into the soma while simultaneously simulating injection of multiple sine waves, each at a different frequency determined from transfer resonance analysis, into distal apical compartments. Our results demonstrate a form of phase modulation of power where the amplitude of the faster signals transmitted from distal apical compartments to the soma is varied as a function of the phase of the slower somatic modulating signal. This type of process may underlie phase-amplitude coupling observed in EEG data.

**References**

1. Larkum ME A cellular mechanism for cortical associations: an organizing principle for the cerebral cortex. *Trends Neurosci*. 2013, 36(3), 141–151. 10.1016/j.tins.2012.11.006
2. Hutcheon B, Yarom Y. Resonance, oscillation and the intrinsic frequency preferences of neurons. *Trends Neurosci*. 2000 23(5), 216–222.
3. Zhuchkova E, Remme MW, Schreiber S. Somatic versus dendritic resonance: differential filtering of inputs through non-uniform distributions of active conductances. *PLoS One* 2013. 8(11):e78908. 10.1371/journal.pone.0078908
4. Hay E, Hill S, Schürmann F, et al. Models of neocortical layer 5b pyramidal cells capturing a wide range of dendritic and perisomatic active properties. *PLoS Comput Biol* 2011. 7(7):e1002107. 10.1371/journal.pcbi.1002107

## P186 Impact of small world connectivity on a multi-region model of cerebral cortex

### David Boothe^1^, Alfred Yu^2^, Kelvin Oie^2^, Piotr Franaszczuk^2^

#### ^1^U.S. Army Research Laboratory, Computational and Information Sciences Directorate, Aberdeen Proving Ground, MD, United States; ^2^U.S. Army Research Laboratory, Human Research and Engineering Directorate, Aberdeen Proving Ground, MD, MD, United States

##### **Correspondence**: David Boothe (david.l.boothe7.civ@mail.mil)

*BMC Neuroscience* 2018, **19(Suppl 2):**P186

How constrained is brain activity by underlying neuronal connectivity? Here we apply concepts from network science to understand the role connectivity plays in generation of large scale brain dynamics [1]. We simulate a network of multiple cortical brain regions each spatially distinct and connected through cortico–cortico white matter tracts. We vary connectivity between simulated patches and measure the small worldness of network connectivity. Additionally, we explore how physiologically relevant factors like synaptic conductance, neuronal origin, and neurotransmitter type interact with ‘who is connected to who’ network graphs to change large scale brain dynamics [2]. We use a modified version of the Traub cortical model [3] implemented in the GEneral NEural SImulation System (GENESIS) [4] simulation framework on high performance computing resources. The model includes three neurotransmitters (AMPA, NMDA and GABA), 12 neuron types, modeled using 15 voltage gated channels, within 50 to 137 compartments. This level of biophysical fidelity allows one to observe changes in the local field potential (LFP) as an emergent property of the physics of the simulation. In order to keep overall levels of neuronal activity sparse, and to emphasize the role of cortical connectivity on network behavior simulated neurons were tuned to eliminate intrinsic activity. Activity levels in individual patches were manipulated by changes in local connectivity probability and the prevalence of Poisson distributed noise inputs. Initial simulations indicate that in a network of four patches (of about 1000 neurons each) that long range connectivity can significantly alter model behavior by shifting the LFP from 1/f like background activity to large scale oscillations. We expand our model to include multiple cortical regions and focus our analysis on generation of large scale brain dynamics hypothesized to be strongly impacted by cortical connectivity such as: 1, the generation of 1/f in the power spectrum of the local field potential (LFP), 2, the relationship between local and global oscillatory behavior in the LFP, and 3, multiscale dynamics from spikes to changes in the LFP.

**References**

1. Watts D, and Strogatz S. *Nature* 1998, 393, 440–442.
2. Hilgetag C and Goulas A. *Brain Struct Funct* 2016, 221, 2361–2366
3. Traub, et. al. *J Neurophysiol* 2016, 93(4), 2194–232
4. Bower JM, Beeman D. *The book of GENESIS: a workbook of tutorials for the General Neural Simulation System*. 1994. Springer, New York.

## P187 Transcranial direct current stimulation (tDCS) is impacted by neuronal morphology and spatial configuration

### Alfred Yu^1^, David Boothe^2^, Kelvin Oie^1^, Piotr Franaszczuk^1^

#### ^1^U.S. Army Research Laboratory, Human Research and Engineering Directorate, Aberdeen Proving Ground, MD, United States; ^2^U.S. Army Research Laboratory, Computational and Information Sciences Directorate, Aberdeen Proving Ground, MD, United States

##### **Correspondence**: David Boothe (david.l.boothe7.civ@mail.mil)

*BMC Neuroscience* 2018, **19(Suppl 2):**P187

We investigated the widely-held belief that transcranial direct current stimulation (tDCS) raises or lowers the probability of neuronal action potential generation [1]. We used GENESIS to simulate a network of neurons with fully realized spatial geometry, including realistic dendritic arborization. We show that taking into account individual neuronal morphology produces variation in stimulation outcomes with consequences on both spiking and network level activity, including oscillatory activity as observed in LFP and EEG. We implemented the hypothesized tDCS mechanism initially with an input current injected directly into the soma where spike generation is initiated. However, it is known that current densities can vary greatly as a function of the spatial relationship between the stimulating electrodes and the region being stimulated [2] and as a function of the spatial morphology of the cells in questions [3]. We extended the somatic stimulation model to a whole-cell stimulation model where stimulation arrives at every compartment with current amplitudes scaled according to compartment orientation and surface area. We found that despite similar characteristics in the single-neuron case (e.g. probability of firing as a result of a spike train into the soma), network-level activity is greatly impacted by whole-cell stimulation. Specifically, we observe a greater range of variability in both firing rate and power spectral density in response to stimulation. In addition, we show that the network-level effects of tDCS are dependent on the orientation of the main trunk of the axon relative to the electric field, highlighting its important role in action potential initiation and propagation. These results could be used for predictive modeling of the functional outcomes of tDCS in specific regions, extending existing approaches of modeling current density by additionally incorporating information about the orientation of the cortical surface. Spatial orientation-dependent variation in efficacy could be particularly important to account for in regions with high variability in spatial extent across individuals.

**References**

1. Nitsche MA, Paulus W. Excitability changes induced in the human motor cortex by weak transcranial direct current stimulation. *J*. *Physio*. 2000, 15, 527, 633–639
2. Datta A, Truong D, Minhas P, et al. Inter-Individual Variation during Transcranial Direct Current Stimulation and Normalization of Dose Using MRI-Derived Computational Models. *Front*. *Psychiatry* 2012, 3, 91.
3. Rahman SA, Marcu S, Shapiro CM, et al. Spectral modulation attenuates molecular, endocrine, and neurobehavioral disruption induced by nocturnal light exposure. *American Journal of Physiology* 2011.

## P188 Simulating extracellular signatures of action potentials using single compartment neurons and geometrical filtering

### Harry Tran, Steven Le Cam, Valérie Louis Dorr, Radu Ranta

#### Université de Lorraine, CRAN UMR 7039, Nancy, France

##### **Correspondence**: Harry Tran (harry.tran@univ-lorraine.fr)

*BMC Neuroscience* 2018, **19(Suppl 2):**P188

Simulating extracellular recordings of single/multiple neurons or of complete populations is an important and challenging task both for understanding the nature of extracellular field potentials (LFP) at different scales and for validating signal processing tools (e.g. despiking/spike sorting). State of the art models [1], based on neuron models having multiple active or passive compartments, show that the extracellular signatures of both synaptic sources and membrane sources during action potentials depend on the geometry of the neurons and on the position of the measuring electrode with respect to this geometry. The simulation methodology, based on the NEURON environment [2] and the LFPy python package [3], could require a high computational burden for large neuronal populations [4]. We propose a new method to simulate the extracellular images of action potentials. Our method takes into account the geometry of the neuron and the position of the electrode, but only requires to model the dynamics of a single compartment. Different shapes are next obtained using a filter based on the geometrical properties of the recording setup, with an overall much smaller computational burden.

More precisely, we start from the classical assertion that at every time instant t, the potential recorded by the electrode is a weighted sum of the membrane currents of all compartments, the weights depending on the medium conductivity (assumed constant) and the geometry (relative position of the compartments and the electrode). The basic idea of our method is to model the membrane current sources active during the action potential as a moving dipole, oriented parallel to the axon and moving with a certain speed along it. In this case, one can show that this weighted sum is equivalent to a convolution between a geometry-conductivity based filter and the membrane currents of a single active compartment. Our model of extracellular spikes is thus generated by a single-compartment Hodgkin-Huxley neuron, convolved with a filter, analytically computed using the shape of the considered neuron. We have tested our model by comparing it with the NEURON/LFPy simulation of a neuron having the same structure (same shape and number of active compartments), in our case a simple ball-stick model (see Fig. 1). The resulting extracellular spikes showed a very good correlation between the two models (mean value above 0.9). Moreover, their shapes were also close to experimental extracellular recordings reporting different spike shapes, depending on the type of neuron (inhibitory/excitatory) and on the position of the electrode with respect to the soma or to the axon [5].

**Fig. 1:**
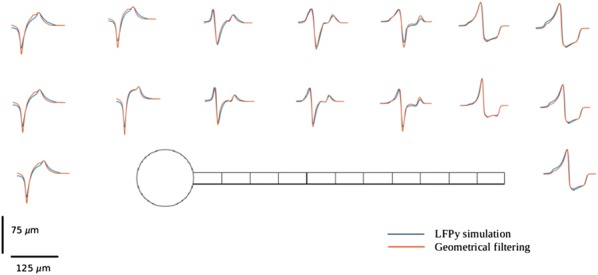
Normalized amplitude, position-dependent extracellular action potentials waveforms. Each waveform position corresponds to an electrode position. The ball-and-Stick neuron is stimulated with an IClamp stimulation on the soma. Diameters of the soma and axon are scaled up for visualization purposes

**References**

1. Einevoll GT, et al. Modelling and analysis of local field potentials for studying the function of cortical circuits. *Nat*. *Rev*. *Neuroscience* 2013, 14(11), 770.
2. Hines ML, Carnevale NT. The NEURON simulation environment. *Neural Comput*. 1997, 9(6), 1179–1209.
3. Lindén H, et al. LFPy: a tool for biophysical simulation of extracellular potentials generated by detailed model neurons. *Frontiers in Neuroinformatics* 2014, 7, 41.
4. Camunas-Mesa LA, Quian-Quiroga R. A detailed and Fast Model of Extracellular Recordings. *Neural Comput*. 2013, 25(5), 1191–1212.
5. Robbins AA, et al. Short duration waveforms recorded extracellularly from freely moving rats are representative of axonal activity. *Frontiers in Neural Circuits* 2013, 7, 181.

## P189 Learning the payoffs and costs of actions

### Moritz Moeller, Rafal Bogacz

#### University of Oxford, Nuffield Department of Clinical Neurosciences, Oxford, United Kingdom

##### **Correspondence**: Moritz Moeller (moritz.moeller@ndcn.ox.ac.uk)

*BMC Neuroscience* 2018, **19(Suppl 2):**P189

The basal ganglia is a subcortical structure common to all vertebrates, which receives input from many cortical areas, and routes its output mainly to the thalamus. Both habit learning and action selection are associated with that part of the brain; however, not much is known about concrete implementation of these functionalities. A novel, integrated theory of learning and action selection, proposed by Collins and Frank (2014), provides a mechanistic description of how the basal ganglia works. That theory differs from conventional reinforcement learning in one critical aspect: decisions are not based on the learned value of an action, but rather on payoff and cost of the action, which are both learned distinctly, and the motivational state. According to this theory, the motivational state balances two behavioural strategies: minimization of cost on the one hand, and maximization of payoff on the other. The theory also maps remarkably well on the relevant physiological features: the basal ganglia circuit includes two main pathways—the action-facilitating Go pathway, and the action-inhibiting NoGo pathway—which are differently modulated by dopamine. Payoffs and costs might be represented by activity of neurons in those pathways, and the motivational state by tonic activity of dopaminergic neurons, which inhibits the NoGo pathway and amplifies the Go pathway. However, there remains a difficult question: how does the basal ganglia acquire its separate knowledge of payoff and cost of actions? We propose a learning mechanism capable of learning the mean payoffs and costs of actions. We designed this learning mechanism to infer the payoffs and costs solely from prediction errors, which encode the surprise about the outcomes of actions, and are thought to be the dominant feedback signal in the basal ganglia. The learning rules we propose formalize the idea that, to learn payoff and cost, positive reinforcement should mainly strengthen the Go pathway, while negative reinforcement should mainly strengthen the NoGo pathway. We demonstrate the learning mechanism’s capability to learn payoffs and costs both numerically and—in the case of purely stochastic or purely deterministic rewards—also analytically. Furthermore, our model accounts for experimental results concerning the effects of dopamine depletion on willingness to exert effort. In an experiment by Salamone et al. [1], rats could choose between pressing a lever to obtain a food pellet, and obtaining free lab chow. Normal animals were willing to press the lever for pellets, but after dopamine depletion, they preferred costless lab chow. Our model accounts for this behaviour both qualitatively and quantitatively: under dopamine depletion, the activity of NoGo neurons is enhanced. Hence, the cost of lever-pressing is over-weighted and the costly action is supressed. Previous work showed that the model also accounts for the effect of dopaminergic manipulations on decision making under risk, and that the proposed learning rules are consistent with known synaptic plasticity and the properties of dopaminergic receptors in the basal ganglia.

**Reference**

1. Salamone JD, Steinpreis RE, McCullough LD, et al. Haloperidol and nucleus accumbens dopamine depletion suppress lever pressing for food but increase free food consumption in a novel food choice procedure. *Psychopharmacology* 1991, 104(4), 515–521.

## P190 A network of intrinsic oscillators can drive forward locomotion in* C. elegans*

### Erick Olivares, Eduardo Izquierdo, Randall Beer

#### Indiana University, Cognitive Science Program, School of Informatics and Computing, Bloomington, IN, United States

##### **Correspondence**: Erick Olivares (erickolivaresb@gmail.com)

*BMC Neuroscience* 2018, **19(Suppl 2):**P190

While central pattern generators (CPGs) are involved in animal locomotion from leech to humans, their role in*C*. *elegans*is still unclear. With detailed behavioral, biomechanical, physiological, and neuroanatomical information available, the question of whether it is possible for a chain of CPGs to drive forward locomotion can be answered through a brain-body-environment computational model.*C*. *elegans*locomotes in an undulatory fashion, generating thrust by propagating dorsoventral bends along its body. Three hypotheses for its locomotion have been postulated: (a) Stretch-receptor feedback driven mechanism for generating and propagating oscillations; (b) A CPG in the head for generating oscillations with stretch-receptor feedback propagating the wave down the body; and (c) A chain of multiple network CPGs in coordination along the body. Existing neuromechanical models of forward locomotion have demonstrated the feasibility of the first two hypotheses. In this work, we build a neuromechanical model to test the third hypothesis. In recent work, we identified a repeating subcircuit in the Ventral Nerve Cord (VNC) that could intrinsically reproduce the neural activity associated with forward locomotion during freely-moving behavior. In the current study, we integrate multiple VNC neural units along the length of the worm’s body (Fig. 1A), interconnected via the set of chemical and electrical synapses obtained from the connectome dataset, and we embed the nervous system in a model of the worm’s musculature, which in turn drives a 2D physical model of the body in an agar environment (Fig. 1B). We use an evolutionary optimization algorithm to set the unknown physiological parameters of neurons, synapses, and neuromuscular junctions so as to match the mean velocity observed in worms moving on agar. We performed 160 evolutionary runs using different random seeds and consistently found electrophysiological configurations that reproduced realistic control of forward movement. The evolved solutions demonstrate for the first time that the VNC can not only generate intrinsic oscillations, but that those oscillations can propagate throughout the VNC to drive forward locomotion in the absence of stretch-receptor feedback. We filtered evolved solutions to those that matched experimental characterizations of the kinematics of forward locomotion and the effect of neural ablations on movement. We selected one representative model to understand in detail its neuromechanical machinery and generate testable experimental hypothesis. We found that a subset of 6 of the 15 connections in the model were necessary and sufficient to drive locomotion (Fig. 1A, C). Oscillations were generated independently in each unit in a subcircuit comprising neurons AS, DA, and DB. Muscle activity is driven mainly by DB and VB neurons while neurons DD and VA can be completely removed without compromising locomotion. A difference in phase between the oscillations of adjacent subunits is controlled by a single electrical synapse between VB and DB neurons. Interestingly, the difference in phase occurs through from the anterior to the posterior unit, even though the exchange of information in the gap junction is bidirectional. Although the mechanism used by the selected model was representative of the majority of the solutions in the ensemble, analysis of the rest of the solutions revealed a set of alternative hypotheses for both generating and propagating the oscillations.

**Fig. 1:**
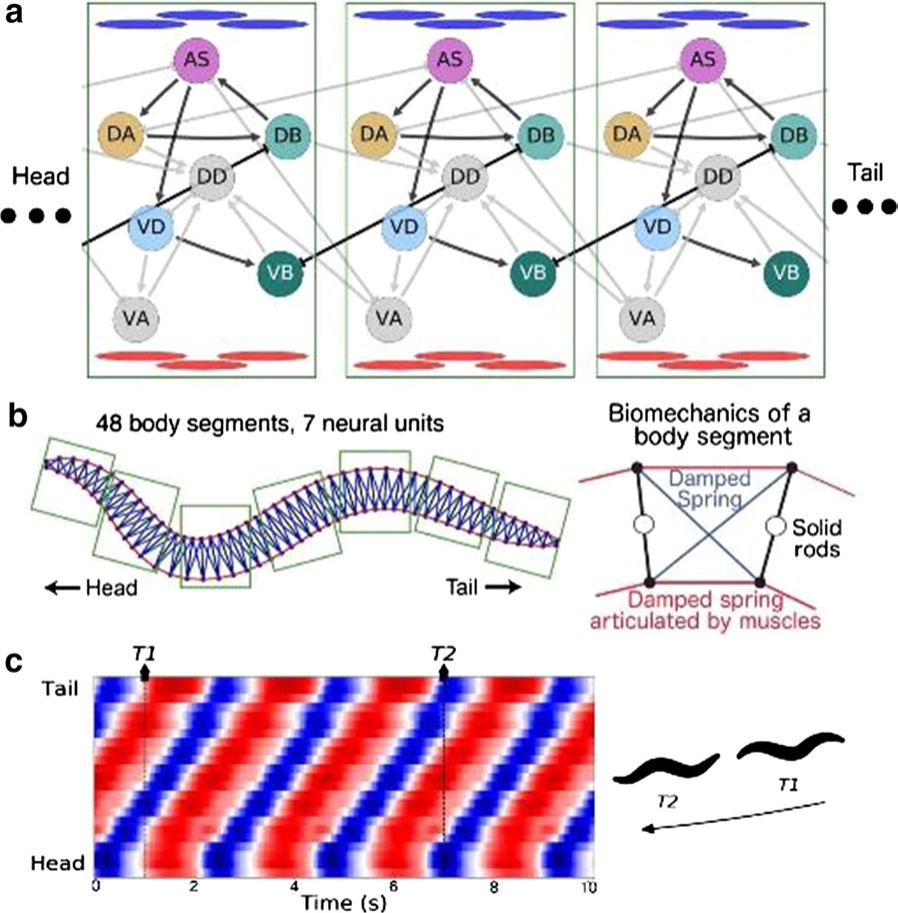
[A] Segmental model of C. elegans VNC, green boxes delimits neural units. Connection present in the minimal configuration of the selected model in black, dorsal and ventral muscles in blue and red respectively. [B] 2D C. elegans Mechanical model and detail of biomechanical segment. The green boxes represent the body area innervated by a neural unit. [C] Forward locomotion in the minimal configuration model showing dorsoventral bending waves along the worm body (vertical axis) and time (horizontal axis) resembling those observed in forward crawling worms. Worm posture is depicted at times T1 and T2

## P191 Computational validation of a closed loop neuromorphic controller for ventilatory control

### Ricardo Siu^1^, James Abbas^2^, Brian Hillen^1^, Sylvie Renaud^3^, Ranu Jung^1^

#### ^1^Florida International University, Biomedical Engineering, Miami, FL, United States; ^2^Arizona State University, School of Biological and Health Systems Engineering, Tempe, AZ, United States; ^3^Université de Bordeaux, IMS Laboratoire – Bordeaux INP, Talence, France

##### **Correspondence**: Ricardo Siu (rsiu001@gmail.com)

*BMC Neuroscience* 2018, **19(Suppl 2):**P191

Respiration supplies oxygen to maintain metabolic processes while simultaneously removing carbon dioxide. It can be impaired due to several pathologies such as medullary stroke or spinal cord injury. Individuals with such impairments often rely on mechanical ventilation, which can lead to diaphragm atrophy and alveolar damage. An alternative to mechanical ventilation, respiratory pacing, utilizes electrical stimulation of the phrenic nerve to cause diaphragmatic contraction or direct stimulation of the diaphragm muscle. These systems facilitate ventilation and thus respiration in a more physiological manner and thus avoid many of the drawbacks associated with mechanical ventilation. Since stimulation parameters are pre-selected and are kept fixed outside of a clinical setting, the systems have to be manually tuned and may not account for changes in respiratory conditions that can lead to insufficient ventilation. We aimed to develop a neuromorphic closed-loop adaptive controller capable of accounting for mechanical, muscular, and metabolic changes to maintain adequate ventilation on a breath-by-breath basis. The closed-loop adaptive controller has a pattern generator (PG)/pattern shaper (PS) architecture that produces a periodic set of adaptive stimulation parameters for diaphragmatic pacing to obtain a desired breath-by-breath ventilatory profile while maintaining normocapnia. The PG is a biomimetic model of the respiratory central pattern generator (rCPG) that responds to changes in arterial CO2. The output of the PG is transformed into a volume profile by a biomechanical chest and diaphragm model. This volume profile and breath duration are set as the ideal target and sent to the PS. The PS consists of an artificial neural network with time-shifted activation profiles. The instantaneous error signal between the measured breath volume and the desired breath volume profile modifies this network to shape the output to the stimulator. As the controller adapts, a stimulation profile develops that can elicit a breath volume profile that matches the desired breath volume profile in both shape and duration. A computational model of rat ventilatory biomechanics paired with a three-compartment model of CO2dynamics was developed in LabVIEW and used to assess performance of the PG/PS controller. Simulations compared a fixed-PG/adaptive PS system against a closed-loop adaptive PG controller. CO2production rate was varied from 2 to 30 mM/s to simulate changes in metabolic function and to assess the working range of CO2production over which the PG could maintain normocapnia. The closed-loop PG/PS could maintain normocapnia with an increased range of 4 to 26 mM/s as compared to a 9 to 13 mM/s seen with the fixed PG/adaptive PS controller. Restrictive respiratory disease was modeled to validate the controller under a pathological state. Inspiratory root mean square error (iRMSE) remained below 10% (iRMSE of 4.47 ± 0.41%) for all trials and PaCO2remained within normocapnic value of 44.9 ± 3.0 mmHg.

The adaptive capabilities of the controller enabled it to maintain normocapnia over a broad range of CO2production rates, thus demonstrating that the system can automatically adjust ventilation to meet the metabolic demands of an individual. Additionally, simulations also suggest that the controller can adapt to changes in the physiological state of the user (Fig. 1).

**Fig. 1:**
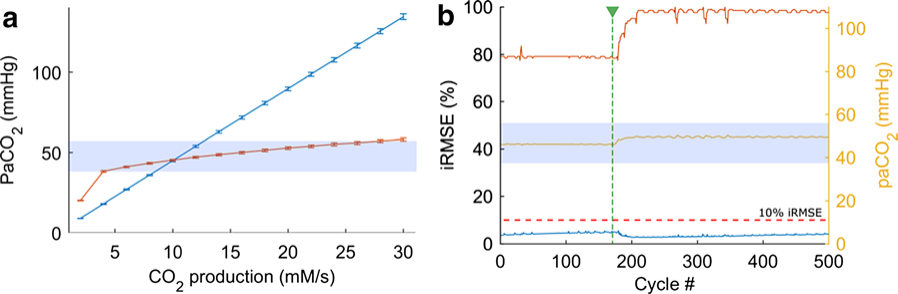
PG/PS controller performance across a range of CO2 production rates and physiological conditions. (A) PaCO2 response for an open-loop PG/adaptive PS (blue line) and closed-loop adaptive PG/adaptive PS (orange line). The closed-loop PG/PS could maintain normocapnia (blue shade) within the CO2 production rates tested for all conditions. (B) PG/PS performance for a restrictive pulmonary lung disease model for a CO2 production rate of 14 mM/s. CO2 production rate was increased to 14 mM/s after three minutes (vertical green dashed line). The PG responds by increasing minute ventilation (orange line) to account for the change in PaCO2v (yellow), thus maintaining normocapnia (blue shade). iRMSE (blue line) remained below 10% (red dashed line) throughout the trial showing that regardless of a changing PG, the PS could elicit a breath volume that could match the desired volume profile

**Acknowledgements**

Supported by National Institutes of Health (R01-NS086088).

## P192 Modeling the altered function of canonical feedback inhibitory circuits in chronic epilepsy

### Christian Klos^1^, Leonie Pothmann^2^, Oihane Horno^3^, Oliver Braganza^2^, Heinz Beck^2^, Raoul-Martin Memmesheimer^1^

#### ^1^University of Bonn, Neural Network Dynamics and Computation, Institute of Genetics, Bonn, Germany; ^2^University of Bonn, Laboratory for Experimental Epileptology and Cognition Research, Department of Epileptology, Bonn, Germany; ^3^Champalimaud Center for the Unknown, Cortical Circuits Laboratory and Theoretical Neuroscience Laboratory, Lisbon, Portugal

##### **Correspondence**: Christian Klos (cklos@uni-bonn.de)

*BMC Neuroscience* 2018, **19(Suppl 2):**P192

Canonical feedback inhibitory motifs play a key role in controlling and structuring the activity of principal cell ensembles. Despite the importance of these motifs and evidence for altered inhibition in chronic epilepsy, changes in the function of canonical feedback inhibition in chronic epilepsy have not been investigated. On the basis of experimental results from the hippocampal region CA1 in the pilocarpine model of chronic epilepsy, we use theoretical modeling to examine how the functional properties of the canonical feedback circuit motifs are changed and how this influences the filtering of signals from CA3. The experiments indicate that intrinsic excitability is reduced in both basket and pyramidal cells. Furthermore, the response of basket cells to stimuli mimicking feedback excitation exhibits reduced initial excitation as well as reduced synaptic depression of the synapse between pyramidal and basket cells. Finally, the initial feedback inhibition to pyramidal cells is strongly reduced. Here we show that simple, biologically plausible neuron and synapse models for basket cells and for the pyramidal cell-to-basket cell-synapses match the data on basket cell responses for suitably chosen parameters. We use this to quantify and interpret the experimentally observed pathological changes. To visualize our results, we project the reproduction error nonlinearly onto the relevant model parameters. This shows a qualitative difference between the parameter sets characterizing the synapses on healthy and pathological basket cells. A similar approach can be employed for the pyramidal cells and the basket cell-to-pyramidal cell synapses. We combine the obtained models for the basket and pyramidal cells and their synapses to models of the complete feedback circuit motif in CA1 for healthy and epileptic animals. Probing them with inputs from CA3, we find that the entirety of the changes in the feedback motif leads to increased activity of the pyramidal cells in the epileptic case especially in case of steep rises of the signal, which are typical for the initial phase of epileptic bursts. This suggests that the changes in CA1 during development of epilepsy promote the transmission of epileptic bursts from CA3 to other parts of the brain.

**Acknowledgements**

This work was supported by the German Federal Ministry for Education and Research BMBF through the Bernstein Network (Bernstein Award 2014), the Deutsche Forschungsgemeinschaft (SFB 1089), the BONFOR program of the University of Bonn Medical Center, and the ERANET Neuron grant ‘EpiNet’.

## P193 Investigating impact of synaptic inputs in seizure models

### Cengiz Gunay, Reuben Massaquoi

#### Georgia Gwinnett College, School of Science and Technology, Lawrenceville, GA, United States

##### **Correspondence**: Cengiz Gunay (cengique@users.sf.net)

*BMC Neuroscience* 2018, **19(Suppl 2):**P193

Recent advancements in high-throughput analysis of brain connectivity have been revealing. However, without knowledge of neuronal dynamics, the connectome alone provides insufficient understanding of nervous system functionality and disorders. In particular, dysfunction of neuronal ion channels can cause major central nervous system disorders such as epilepsy that affects about 1% of the global population. Role of ion channels in electroshock-induced seizures can be investigated using the slamdance mutant fruitfly. However, developmental compensation in mutants prevents relating channel activity to behavioral outcomes. Channel effects were successfully measured in isolation previously using a computational neuron model. This model only represented a single motoneuron without considering contributions from other neurons. The recent availability of electron micrograph connectivity from the fly larva allows reconstructing a more detailed anatomical morphology of this motoneuron, which includes the surrounding circuit. We propose to reconstruct this circuit on the computer to investigate effects of synaptic inputs on seizure activity via simulation.

## P194 Fundamental neuromechanical components of robust forward locomotion in* C. Elegans*

### Carter Johnson, Timothy Lewis, Robert Guy

#### University of California, Davis, Department of Applied Mathematics, Davis, CA, United States

##### **Correspondence**: Carter Johnson (caljohnson@ucdavis.edu)

*BMC Neuroscience* 2018, **19(Suppl 2):**P194

Animal behavior is generated by neural activity as well as sensory feedback and the biomechanical constraints of the body in an environment. Locomotion of C. elegans is an ideal model to study these neuromechanical interactions, due to its well-described connectome and limited behavioral repertoire including forward crawling or swimming. The motor circuit responsible for forward locomotion in C. elegans consists of a chain of local modules driven by a single command neuron (White et al. 1976). C. elegans swims forward by undulation, underlain by a wave of curvature traveling down the body. There is substantial evidence that suggests coordination of the local neuromechanical modules arises via proprioceptive coupling (Wen et al. 2012). It is also known that C. elegans can change its curvature waveform in media of varying viscosities to match the loads imposed by the environment (Berri et al. 2009; Boyle et al. 2012; Fang-Yen et al. 2010). The exact mechanisms generating oscillatory activity and coordinating these oscillations to produce appropriate locomotive behavior is a subject of ongoing. To examine the fundamental components necessary for robust oscillations yielding forward locomotion, we construct a minimal model for neuromuscular control of C. elegans based on the model of Boyle et al., 2012. The model consists of a chain of neuromechanical modules connected by both mechanical coupling and proprioceptive feedback, a schematic of which is shown in Fig. 1. We characterize the parameter dependence of the activity in a single module and therefore provide insight into the basic mechanism generating oscillatory behavior. We then couple two to twelve such oscillating modules together, and examine the relative roles of local, mechanical coupling and long-range, proprioceptive coupling in producing coordinated behavior. We use this model to make testable predictions of swimming speed of C. elegans in different environments, and analyze how the strength of neural feedback from body mechanics, the strength of mechanical coupling, and the role of directional, proprioceptive coupling give rise to the adaptive swimming gaits.

**Fig. 1:**
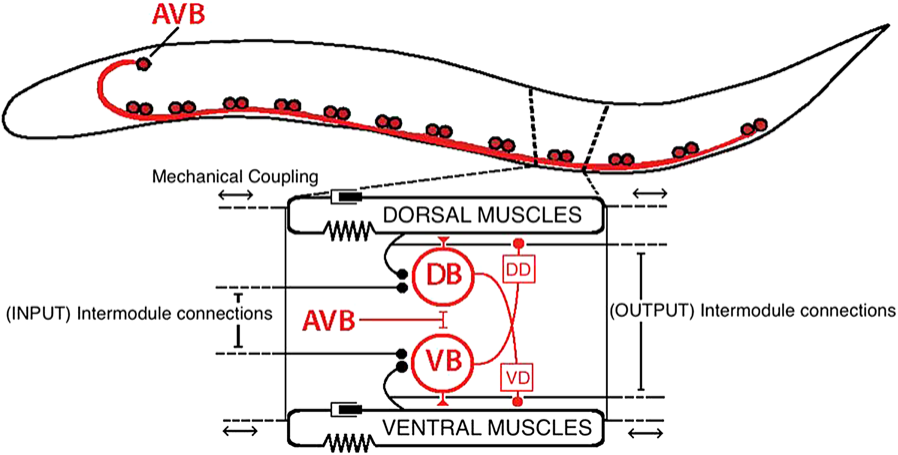
Schematic of the neuromechanical model of C. elegans forward locomotion. The AVB command neuron drives all twelve neuromechanical modules, which are connected together via both mechanical coupling and intermod- ular (proprioceptive) coupling. Each module consists of two B-class neurons, which exhibit ipsilateral excitation and contralateral inhibition of body wall muscles. The contralateral inhibition is relayed through the D-class neurons. The viscoelastic body wall muscles are modeled as springs and dashpots in parallel, with an additional active tension from neural excitation. The local proprioception is modeled as ipsilateral inhibition of the B-class neurons by the muscles, and the long-range proprioception modeled as ipsilateral inhibition of the B-class neurons by muscles in anterior modules (INPUT label). The muscles in each module also exhibit ipsilateral inhibition of B-class neurons in posterior modules (OUTPUT label), and are mechanically coupled to ipsilateral muscles in neighboring modules

**References**

1. White, J.G., Southgate, E., Thomson, J.N., and Brenner, S. The structure of the ventral nerve cord of Caenorhabditis elegans. *Philos*. *Trans*. *R*. *Soc*. *Lond*. *B Biol*. *Sci*.1976, 275, 327348.
2. Wen Q, Po MD, Hulme E, Chen S, Liu X, Kwok SW, Gershow M, Leifer AM, Butler V, Fang- Yen C et al.: Proprioceptive coupling within motor neurons drives C. elegans forward locomotion. *Neuron* 2012, 76:750–761.
3. Berri S, Boyle JH, Tassieri M, Hope IA, Cohen N: Forward locomotion of the nematode C. elegans is achieved through modulation of a single gait. *HFSP J* 2009, 3:186–193.
4. Boyle JH, Berri S, Cohen N: Gait modulation in C. elegans: an integrated neuromechanical model. *Front Comput Neurosci* 2012, 6:10.
5. Fang-Yen C, Wyart M, Xie J, Kawai R, Kodger T, Chen S, Wen Q, Samuel ADT: Biomechanical analysis of gait adaptation in the nematode Caenorhabditis elegans. *Proc Natl Acad Sci USA* 2010, 107:20323–20328.

## P195 A reservoir computing model of motor learning with parallel cortical and basal ganglia pathways

### Ryan Pyle, Robert Rosenbaum

#### University of Notre Dame, Applied and Computational Mathematics and Statistics, South Bend, IN, United States

##### **Correspondence**: Ryan Pyle (ryanpyle1@gmail.com)

*BMC Neuroscience* 2018, **19(Suppl 2):**P195

Reservoir computing has been proposed as a model of how the brain learns and generates motor output. Most learning rules used for reservoir computing, including the popular First Order Reduced and Controlled Errors (FORCE) method, are fully supervised. These can learn intricate motor tasks and produce internal dynamics strikingly similar to those of motor cortical neurons, but rely on biologically unrealistic learning rules. As models of biological motor learning, such algorithms could only learn to “copy’’ the output generated in another area of the brain. Moreover, they can only be applied to tasks where the mapping from reservoir output to motor action and its inverse are known explicitly. How are novel motor outputs learned by biological neural networks? Biological motor learning is controlled at least in part by dopamine-modulated reinforcement learning in the basal ganglia. Other, more realistic learning rules based on reinforcement learning algorithms for reservoir computing have been proposed, but are often derived for simplified, discrete tasks in contrast to the intricate dynamics that characterize real motor responses. In addition, we find that they fail to converge on some relatively simple tasks. We bridge these two approaches to develop a biologically realistic learning rule for reservoir computing, Supervised Transfer of Rewarded Exploration (SUPERTREX), that models the interaction between reinforcement and supervised learning observed in mammals and songbirds. Through various learning tasks and simulations, we show that SUPERTREX performs as well as or better than existing learning algorithms for reservoir computing, is more biologically realistic, and is applicable to a larger class of motor learning tasks. Finally, we show that SUPERTREX can reproduce findings that relate Parkinson’s disease and its treatments to motor learning.

## P196 Dynamic features of neural responses to triplet-streaming simulated by integrate-and-fire networks of core auditory cortex

### Aarati Mahat, Rodica Curtu

#### University of Iowa, Department of Mathemathics, Iowa City, IA, United States

##### **Correspondence**: Rodica Curtu (rodica-curtu@uiowa.edu)

*BMC Neuroscience* 2018, **19(Suppl 2):**P196

**Introduction:** Past decades of auditory research have identified several acoustic features that influence perceptual organization of sound, in particular, the frequency of tones and the rate of presentation [1–2]. One class of stimuli that have been intensively studied are sequences of tones that alternate in frequency. They are typically presented in patterns of repeating doublets ABAB…or repeating triplets ABA_ABA_ where the symbol *“*−” stands for a gap of silence between triplets repeats. The duration of each tone or silence is typically tens to hundreds of milliseconds, and listeners hearing the sequence perceive either one auditory object (“stream integration”) or two separate auditory objects (“stream segregation”). Animal studies have characterized single- and multi- unit neural activity and event-related local field potentials while systematically varying frequency separation between tones (DF) or the presentation rate (PR). They found that theBtone responses in doublets were differentially suppressed with increasing PR and that the B tones responses in triplets decreased with larger DF [1–2]. However, the neural mechanisms underlying these animal data have yet to be explained. In this study we built an integrate-and-fire network model of core auditory cortex (AC) that accurately reproduced the experimental results from [1–2]. We then extended the model to account for basic spectro-temporal features of electrocorticography (ECoG) recordings from the posteriomedial part of the Heschl’s gyrus (HGPM; cortical area equivalent to the AC of monkeys), obtained from humans listening to sequences of triplets ABA_.

**Results:** A large network of voltage-dependent leaky integrate-and-fire neurons (3600 excitatory, 900 inhibitory) was constructed to simulate neural activity from layers 3/4 of AC during streaming of doublets and triplets. Parameters describing synaptic and membrane properties were based on experimental data from early studies of AC [3–4]. Network structure assumed spatially-dependent probability of connections and tonotopical organization. Subpopulations of neurons were tuned to different frequencies along the tonotopic map. In-silico recordings were performed during the presentation of long sequences of triplets and/or doublets. The network’s output was derived with two types of measurements in mind: spiking activity of individual neurons and/or local populations of neurons, and local field potentials [5–6]. The network spiking neural activity reproduced reliably data reports from [1–2], including dependence of responses to the B tone in triplets ABA_ on stimulus parameter DF. Approximations of average evoked potentials (AEPs) from ECoG signals recorded at four depth contacts placed over human HGPM during auditory streaming of triplets were also obtained. The model accounted for features of HGPM activity such as short-latency large-amplitude responses and robust isomorphic representation of acoustic stimulus properties, including onsets and offsets of individual tones within the triplet.

**Acknowledgement**

This work received support from NSF-CRCNS 1515678.

**References**

1. Micheyl C, Tian B, Carylon R, et al.. Perceptual Organization of Tone Sequences in the Auditory Cortex of Awake Macaques. *Neuron* 2005, 48, 139–148.
2. Fishman YI, Reser DH, Arezzo JC, et al. Neural correlates of auditory stream segregation in primary auditory cortex of the awake monkey. *Hear Res* 2001, 151, 167–187.
3. Schiff ML, Reyes AD, Characterization of thalamocortical responses of regular-spiking and fast-spiking neurons of the mouse auditory cortex in vitro and in silico. *JNeurophys* 2012, 107, 1476–1488
4. Levy RB, Reyes AD. Spatial Profile of Excitatory and Inhibitory Synaptic Connectivity in Mouse Primary Auditory Cortex. *JNeurosci* 2012, 32, 5609–5619.
5. Beeman D. A modeling study of cortical waves in primary auditory cortex. *BMC Neurosci* 2013, 15 (1), 32
6. Cavallari S, Panzer S, Mazzoni A. Comparison of the dynamics of neural interactions between current-based and conductance-based integrate-and-fire recurrent networks. *Frontiers in Neural Circuits* 2014, 8, 1–23.

## P197 Reduction of conductance-based neuron models for neuromodulation studies

### Tomas Van Pottelbergh, Rodolphe Sepulchre

#### University of Cambridge, Department of Engineering, Cambridge, United Kingdom

##### **Correspondence**: Tomas Van Pottelbergh (tmjv2@cam.ac.uk)

*BMC Neuroscience* 2018, **19(Suppl 2):**P197

The recently introduced multi-quadratic integrate-and-fire (MQIF) [1, 8] is an economic integrate-and-fire model that captures important modulation properties such as a robust transition between tonic firing and bursting or between distinct excitability types. Such neuronal properties are critical to retain in neuromodulation studies of network models such as [5] or [6] as the neuromodulation not only affects the connectivity but also—and sometimes, predominantly—the excitability properties of the neurons. We revisit the classical method of equivalent potentials [4] with the goal of reducing detailed conductance-based models to the MQIF model. Our method relies on the simulation of the equivalent potentials during a representative behaviour of the model. To model the fast-slow dynamics, we use a least squares fitting to express each equivalent potential as a linear combination of a constant, fast, and slow membrane potential. The role of ultraslow variables is subsequently modelled through the modulation of the fast-slow MQIF parameters and the applied current. We illustrate our method on two different conductance-based models from the literature: the Connor-Stevens model [2, 3] and*Aplysia*R15 model [7]. The reduced integrate-and-fire models are shown to exhibit behaviours close to those of the original models. Most importantly, modulation between different excitability types in the Connor-Stevens model and between tonic spiking and bursting in the*Aplysia*R15 model is shown to be captured well by the key parameters of the MQIF model, which determine the fast and slow points of balance [8].

**Acknowledgements**

T.V.P. received a fees scholarship from the Engineering and Physical Sciences Research Council (https://www.epsrc.ac.uk) under Grant 1611337. Both T.V.P. and R.S. were supported by the European Research Council (https://erc.europa.eu) under the Advanced ERC Grant Agreement 670645.

**References**

1. Drion G, Franci A, Dethier J, Sepulchre R: Dynamic Input Conductances Shape Neuronal Spiking.*eNeuro* 2015, 2(1):ENEURO.0031-14.2015.
2. Connor JA, Stevens C F: Prediction of repetitive firing behaviour from voltage clamp data on an isolated neurone soma.*The Journal of Physiology* 1971, 213(1):31–53.
3. Connor JA, Walter D, McKnown R: Neural repetitive firing: modifications of the Hodgkin-Huxley axon suggested by experimental results from crustacean axons.*Biophysical Journal* 1977, 18(1):81–102.
4. Kepler TB, Abbott LF, Marder E: Reduction of conductance-based neuron models.*Biological Cybernetics* 1992, 66(5):381–387.
5. Lee SH, Dan Y: Neuromodulation of Brain States.*Neuron* 2012, 76(1):209–22.
6. Marder E, Bucher D: Understanding Circuit Dynamics Using the Stomatogastric Nervous System of Lobsters and Crabs.*Annual Review of Physiology* 2007, 69(1):291–316.
7. Rinzel J, Lee YS: Dissection of a model for neuronal parabolic bursting.*Journal of Mathematical Biology* 1987, 25(6):653–675.
8. Van Pottelbergh T, Drion G, Sepulchre R: Robust Modulation of Integrate-and-Fire Models.*Neural Computation* 2018, in press.

## P198 System identification of neuronal dynamics

### Thiago Burghi, Rodolphe Sepulchre

#### University of Cambridge, Department of Engineering, Cambridge, United Kingdom

##### **Correspondence**: Thiago Burghi (tbb29@cam.ac.uk)

*BMC Neuroscience* 2018, **19(Suppl 2):**P198

The voltage clamp experiment, pioneered by Hodgkin and Huxley, is still a widely applied technique for the identification of detailed neuronal models [1]. In system theoretic terms, the success of the voltage clamp experiment rests on the fact that the inverse dynamics of neuronal models are stable and almost linear whereas the direct dynamics from applied current to voltage are unstable and nonlinear. In any conductance based model, the inverse system, from voltage to total current, has an architecture reminiscent of a parallel one layer continuous-time artificial neural network. It is amenable to estimation using standard techniques from the field of systems identification [2]. In this work, we explore this specific architecture by proposing a simple technique based on convex optimization to automatically identify the dynamics of a single compartment neuron model from membrane potential and applied current traces only. Little knowledge of the underlying ion channel structure and kinetics is assumed. As an initial model, we use a large set of simple parallel branches composed of static nonlinearities and linear time-invariant filters, chosen so as to span a large space of dynamic behaviours. We then use L1 regularization in our cost function to ensure that a sparse solution to the optimization problem is found, keeping in our model only those few branches which capture the excitable properties of the neuron at the right amplitude and frequency ranges [3]. This provides a systematic way of modulating the complexity of an identified neuron model, while preserving information about its intrinsic timescales (a computationally relevant feature of single neuron models, as argued in [4]). To illustrate our approach, we estimate the dynamics of conductance-based models from the literature using voltage and applied current data obtained numerically. Advantages of applying this approach in the study of neuronal systems beyond single compartment neurons are also discussed.

**Acknowledgements**

The research leading to these results has received funding from the Brazilian higher education funding agency CAPES and the European Research Council under the Advanced ERC Grant Agreement Switchlet n.670645.

**References**

1. Huys QJM, Ahrens MB, Paninski L: Efficient Estimation of Detailed Single-Neuron Models. *Journal of Neurophysiology* 2006 96:2, 872–890.
2. Ljung L. *Systems Identification: Theory for the user*. Prentice Hall PTR, 1999.
3. Drion G, O’Leary T, Dethier J, Franci A, Sepulchre R: Neuronal behaviors: A control perspective, 2015 *54th IEEE Conference on Decision and Control* (*CDC*), Osaka, 2015, pp. 1923–1944.
4. Gjorgjieva J, Drion G, Marder E: Computational implications of biophysical diversity and multiple timescales in neurons and synapses for circuit performance. *Current Opinion in Neurobiology*, 2016, 37:44–52.

## P199 Neuromorphic hyperpolarized bursting

### Luka Ribar, Rodolphe Sepulchre

#### University of Cambridge, Department of Engineering, Cambridge, United Kingdom

##### **Correspondence**: Luka Ribar (lr368@cam.ac.uk)

*BMC Neuroscience* 2018, **19(Suppl 2):**P199

Neurons possess two elementary modes of firing activity: tonic spiking where neurons periodically elicit single action potentials, and burst-firing, where sequences of high-frequency spikes are followed by quiescent periods. In addition to controlling the frequency and amplitude of the spiking activity, an essential modulation mechanism that can appear in neurons is the transition between these two firing regimes. This behavioral switch has been observed in subthalamic nucleus neurons [1] through the phenomena of *hyperpolarized*-*induced bursting*, whereby a hyperpolarizing injected current can induce a transition from tonic spiking activity to burst firing; this transition has important medical consequences with regards to the development of Parkinson’s disease. From a neural processing standpoint, such a switch can provide a fast mechanism for controlling the network oscillations, providing means for rapid switches in brain states [2]. Our aim is to develop an analysis and synthesis method for realizing neurons whose behavior can be precisely modulated and that are able to experience these fast switches in firing activity. We use a recently introduced design architecture [3] to illustrate how hyperpolarized bursting can be robustly implemented in a neuromorphic circuit. The architecture consists of a parallel interconnection of a membrane capacitor with elementary I-V elements in the form of first-order filters in series with sigmoidal I-V elements. These elements provide local positive or negative conductance acting in a well-defined timescale set by the first-order filters, and thus mimic the effect of the activation and inactivation of ionic currents in conductance-based models. The elements are easily realizable in hardware in sub-threshold MOSFET architecture, and lead to a powerful design and analysis approach by utilizing the model’s fast, slow and ultra-slow I-V curves that are shaped through the gains of the feedback elements. The modulation of the bursting and spiking activity is then fully captured through the synthesis of the I-V curves in each timescale, mimicking the effect of different neuromodulators in the physiological neurons. The main novelty with respect to our previous work is that hyperpolarization-induced bursting strongly relies on the inactivation of T-type calcium channels. The effect of these channels has a direct correspondence to a slow negative conductance element in our model, and the inactivation effect can be easily added to our previous design architecture. By keeping the same feedback structure as in the physiological model, we retain the same robust switch in behavior that does not require parameter fine-tuning, but relies on the well-defined modulation through I-V curve shaping. This simple mechanism allows us to modulate the input–output neural behavior through the hyperpolarization and depolarization of the neuron, opening the possibilities for novel studies of neural network behavior, where robust local behavioral switches can induce qualitative changes in the network oscillations.

**References**

1. Beurrier, C, et al. Subthalamic nucleus neurons switch from single-spike activity to burst-firing mode. *Journal of Neuroscience* 1999, 19(2), 599–609.
2. Dethier D, et al. Cellular control of localized brain states. In preparation.
3. Sepulchre R. Bursting through interconnection of excitable circuits. *Biomedical Circuits and Systems Conference* (BioCAS), 2017 IEEE. IEEE, 2017.

**Publisher’s Note**

Springer Nature remains neutral with regard to jurisdictional claims in published maps and institutional affiliations.

